# A Ni-Mediated Cross-Coupling Approach to Deuterated ^18^F- Fluoromethylated (Hetero)arenes

**DOI:** 10.1021/jacs.6c09649

**Published:** 2026-07-03

**Authors:** Maddison Lovell, Sebastiano Ortalli, Raquel Sánchez-Bento, Matthew Tredwell, Joseph Ford, Véronique Gouverneur

**Affiliations:** † Department of Chemistry, Chemistry Research Laboratory, 6396 University of Oxford, Mansfield Road, Oxford OX1 3TA, U.K.; ‡ MSD, Midland Road, London NW1 1AT, U.K.; § Wales Research and Diagnostic PET Imaging Centre, Cardiff University, University Hospital of Wales, Heath Park, Cardiff CF14 4XN, U.K.; ∥ School of Chemistry, 2112 Cardiff University, Main Building, Park Place, Cardiff CF10 3AT, U.K.

## Abstract

Herein, we report a Ni-mediated ^18^F,D_2_-monofluoromethylation of (hetero)­arenes from (pseudo)­halide coupling partners. This new approach offers key advantages compared to traditional radiochemistry based on S_N_2-type reactivity with [^18^F]­fluoride. These include increased precursor stability and availability, obviating bespoke precursor syntheses, broad functional group tolerance, and the late-stage introduction of deuterium and fluorine-18 in a single step, all features facilitating early access to PET ligands for discovery campaigns. This novel protocol is applied to over 30 diverse substrates, including complex bioactive molecules, and is amenable to scale-up via a semiautomated protocol on a commercial platform, illustrated by isolation of a ^18^F,D_2_-labeled PARP radiotracer.

## Graphical abstract

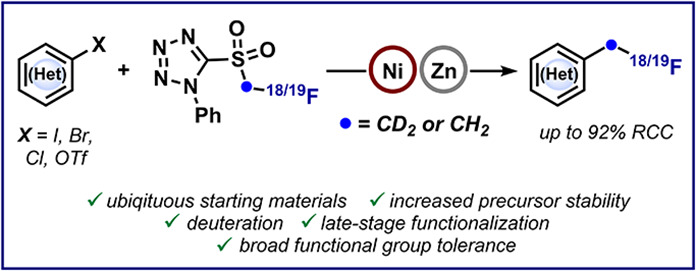

Positron emission tomography (PET) is a highly sensitive, non-invasive imaging technique used widely for clinical diagnosis and staging of numerous diseases.^1^ Furthermore, insights from PET imaging can support the development of novel pharmaceuticals, e.g. with biodistribution and dosing studies.^2^ Fluorine-18 has prevailed as the radionuclide of choice for (pre)­clinical PET imaging due to its advantageous nuclear decay properties (*t*_1/2_ = 109.8 min, 97% β^+^ decay, 635 keV positron energy), enabling the preparation of elaborate radiotracers and acquisition of high-resolution images.^3^ The recent advance in total-body PET imaging and the emerging technology of theranostics has attracted growing attention to ^18^F-radiochemistry.^4,5^ Despite such progress, the mild and selective synthesis of ^18^F-labeled radiotracers remains a significant bottleneck for PET imaging with novel ligands.^6^ Even with substantial advances in fluorine chemistry over the past two decades, translation of these methodologies to ^18^F-radiochemistry is challenging for several reasons, e.g. the requirement for specialist laboratories and equipment, stoichiometry, radiolysis, and handling constraints.^6–8^

For these reasons, radiochemists often apply well-established radiolabeling strategies, such as the nucleophilic displacement of reactive leaving groups with [^18^F]­fluoride (Figure 1).^9,10^ As benzylic positions are primed for such reactivity, ^18^F-fluoromethyl substituents ([^18^F]­CH_2_F) have become prevalent on (hetero)­arenes.^9,10^ Indeed, the fluoromethyl group has been identified as a bioisostere of numerous common functional groups, e.g. thiomethyl and hydroxymethyl, and its incorporation within bioactive molecules has been associated with improved physiochemical properties, e.g. lipophilicity and membrane permeability, rendering it attractive in (radio)­pharmaceutical design.^11^

**Figure 1:**
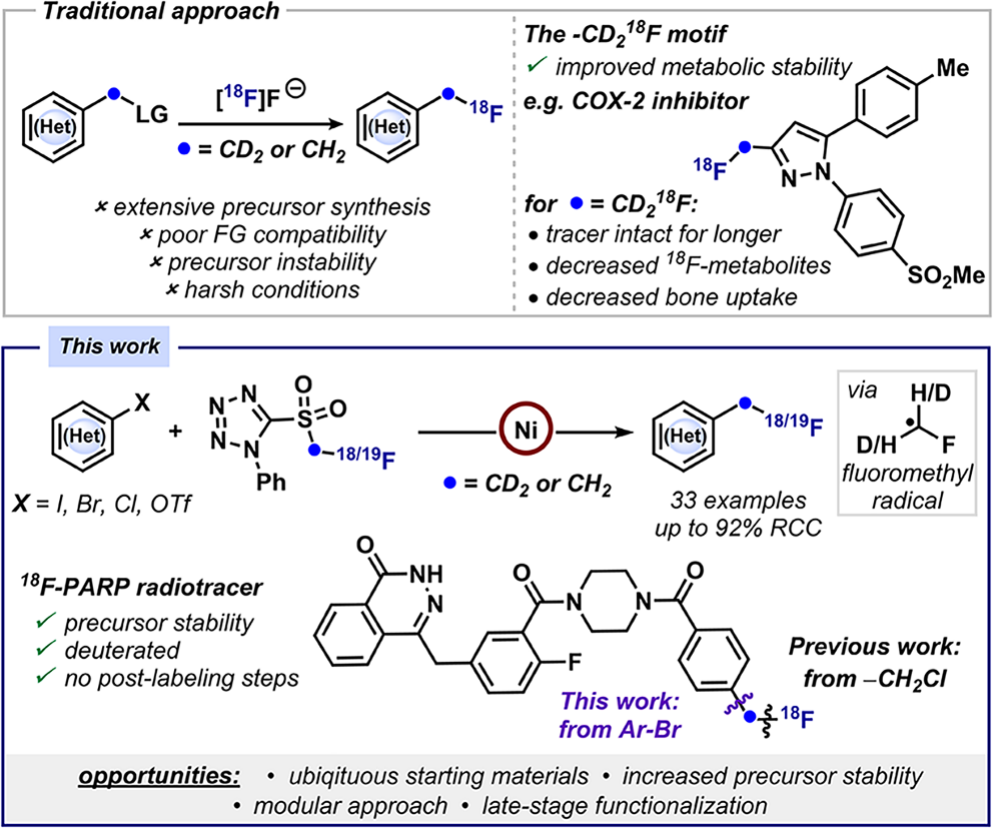
Nucleophilic S_N_2 displacement to ^18^F-fluoromethyl compounds (LG = leaving group), the CD_2_^18^F motif, and this work.

Some radiotracers bearing [^18^F]­CH_2_F are however susceptible to *in vivo* degradation via radiodefluorination.^12–18^ This reduces the concentration of intact radiotracer and produces radiometabolites, often free [^18^F]­fluoride, contributing to background signal due to off-target binding, particularly in bone tissue.^12–17^ To address this, radiochemists have exploited deuteration to increase stability of the ^18^F-radiolabel while maintaining biological activity.^19–25^ For example, Pietzsch and co-workers investigated a ^18^F­(D_2_)-fluoromethylated COX-2 inhibitor, reporting a more favorable metabolic fate for the deuterated isotopolog (Figure 1).^21^ Current radiosynthetic approaches to ^18^F,D_2_-fluoromethylated (hetero)­arenes demand multistep syntheses of bespoke, deuterated labeling precursors bearing preinstalled leaving groups.^21–23^ In addition to synthetic challenges arising from late-stage introduction of reactive groups, careful planning is required to maintain the isotopic integrity of the deuterated precursor in downstream manipulations. Introduction of deuterium often requires harsh reagents, e.g. LiAlD_4_, posing functional group compatibility issues and often demanding early-stage deuterium incorporation.^21–23^ Precursors bearing reactive leaving groups may also suffer from stability issues.^14–17^ For example, Sutherland and co-workers reported the preparation of a ^18^F-labeled poly­(ADP-ribose) polymerase (PARP) radiotracer from a chloromethyl precursor with [^18^F]­fluoride.^16^ However, precursor instability to self-oligomerization between the chloromethyl group and the free phthalazinone N–H necessitated N–H protection and a two-step radiofluorination-deprotection sequence.^16^ Furthermore, Diliën and co-workers recently reported a benzylic bromide precursor to a novel GABA transporter 1 radiotracer that underwent degradation under various storage conditions, and hence preparation of fresh precursor was required for radiolabeling.^17^

With these (radio)­synthetic challenges in mind, a more appealing strategy involves the cross-coupling of ubiquitous aryl halide substrates with an easy-to-produce, radiolabeled and deuterated ^18^F,D_2_-fluoromethylating reagent. While a single report from Suzuki and co-workers describes a Pd-mediated cross-coupling of aryl pinacol boronic esters with [^18^F]­bromofluoromethane, extension to deuteration was not reported.^26^ Challenges associated with volatile radioactive reagents (bp (CH_2_FBr) = 19 °C) have discouraged adoption of this radiochemistry for PET imaging.

In our design plan, we envisaged the ^18^F,D_2_-fluoromethyl radical as a suitable reactive species to engage in a Ni-catalyzed cross-coupling with readily available aryl halides. The cross-coupling of unlabeled (fluoro)­alkyl radicals is documented,^27–31^ however for fluoromethylation is limited to use of volatile, ozone-depleting reagents or preformed air/moisture sensitive organozinc species, both of which prohibit application to ^18^F-radiochemistry.^27–30^ We began our studies considering the structural features of an ideal fluoromethyl radical-releasing reagent, with key characteristics for successful translation to ^18^F-radiochemistry in mind, e.g. accessibility in ^18^F and D_2_-labeled form, non-volatility, lack of ozone-depletion potential, and uncharged for facile reverse-phase HPLC purification. Encouraged by work from our group and others, we selected fluoromethyl sulfones as reagents satisfying these criteria.^30–38^ A library of ^19^F-fluoromethyl sulfones was selected for radical generation via either photocatalysis or reduction with metals, typically zinc or manganese,^39,40^ based on redox properties (*E*_Red_ (V vs SCE): **1**, −2.00 V; **2**, −1.77 V; **3**, −1.42 V; **4**, −1.32 V; **5**, −0.86 V). While photocatalytic methods were unsuccessful, preliminary screening revealed sulfones **2**, **3** and **4** as suitable candidates for the cross-coupling with aryl iodide **6** (1.5 equiv) using a zinc reductant (Tables S1–S10). The use of 4.0 equiv of zinc with dimethyl-2-imidazolidinone (DMI) as the solvent was important for reaction efficiency (Tables S2 and S4). The ligand system was also investigated (Tables S3 and S5), with a bulky tridentate ligand, tri-^*t*^Bu-terpy, in a 1:1.5 Ni:L ratio proving beneficial, yielding the desired benzylic fluoride **7** from **3** in 53% yield (Scheme 1). Under these conditions, sulfones **2** and **4** were less reactive, suggesting there is an optimal, intermediate reduction potential of the reagent. Pleasingly, when reagent [D_2_]**3** was trialed under these conditions, [D_2_]**7** was obtained in 52% yield and in high deuterium incorporation (97%) (Scheme 1, Figure S9).

**Scheme 1:**
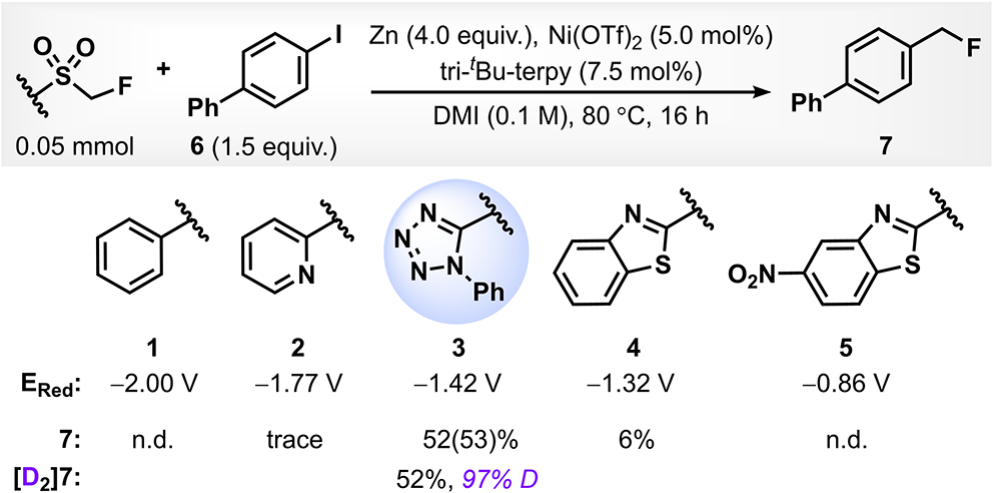
Comparison of Fluoromethyl Sulfone Reagents[Fn s1fn1]

a
*E*_red_ (V vs SCE). Yields and deuterium incorporation determined by ^19^F NMR spectroscopy. Isolated yield in parentheses, n.d. = not detected.

Next, we focused on the radiosynthesis of ^18^F-labeled fluoromethyl sulfone reagents from halomethyl sulfide precursors. Sulfone **2** was rapidly discarded due to challenging precursor preparation, so sulfones **3** and **4** were brought forward for radiochemical experiments (see SI). Sulfone [^18^F]**3** was readily accessed from the requisite bromide or iodide precursors, which were synthesized from commercially available, non-ozone depleting dibromo- and diiodomethane, both also commercially available in deuterated form. Subjecting these precursors to an automated two-step radiofluorination-oxidation protocol afforded [^18^F]**3** in a non-decay corrected (n.d.c.) activity yield (AY) of up to 1.80 GBq (5 ± 2% _(*n*=15)_), from 20 GBq starting activity (Table S14).^33–38^ Similarly, [^18^F,D_2_]**3** was accessed from the corresponding dideutero bromomethyl sulfide precursor in a n.d.c. AY of 2.32 GBq (6%) and a molar activity (*A*_m_) of 109 GBq μmol^–1^ from 40 GBq starting activity (Table S14–S15). With these results in hand, the Ni-mediated reaction of [^18^F]**3** and **6** was examined. Initially we found the use of zinc powder limited reproducibility (Table 1, entry 2), likely due to the heterogeneous nature of the reaction and an increased sensitivity to reliable stirring over the short reaction time scale.^41^ To overcome this, the use of zinc nanopowder (40–60 nm avg. particle size vs powder, 595–841 μm) proved beneficial, forming [^18^F]**7** in 79% radiochemical conversion (RCC) determined by radio-HPLC analysis (Table 1, entry 1). Sulfone [^18^F]**4** was also a competent reagent, albeit to a lesser extent than [^18^F]**3** (Table 1, entry 3 and Scheme 1). Pleasingly, the desired deuterated product [^18^F,D_2_]**7** was obtained in 81% RCC when [^18^F,D_2_]**3** was used (Table 1, entry 4). In line with non-radioactive screening, Mn was not a competent reductant (Table 1, entry 5 and Table S8). The reaction benefitted from increasing the temperature from 80 to 110 °C (Table 1, entry 6). Alternative tridentate ligands such as terpyridine (terpy) proved effective (Table 1, entry 7), and alternative solvents, such as *N*,*N*-dimethyl formamide (DMF) and *N*,*N*-dimethylacetamide (DMA) gave inconsistent results (Table 1, entries 8 and 9). Notably, varying the coupling partner to an aryl bromide, chloride and triflate still enabled ^18^F-fluoromethylation in 72%, 43% and 23% RCC, respectively (Table 1, entries 10–12). The extent of deuterium incorporation was also determined in a carrier-added experiment, whereby [D_2_]**3** was spiked into a reaction of [^18^F,D_2_]**3** and **6** under our optimized conditions (Table 1, entry 1). After radioactive decay, analysis of the crude reaction mixture by quantitative ^19^F NMR spectroscopy revealed 97% deuteration of [^18^F,D_2_]**7**, an identical value to that obtained from the cross-coupling of [D_2_]**3** and **6** (Figures S9 and 10).

**Table 1:**
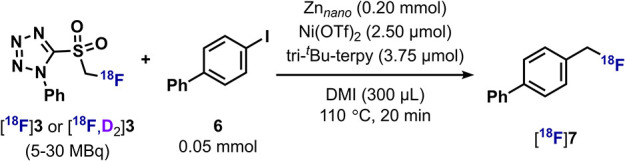
Reaction Optimization^a^

Entry	Deviation	RCC (%)
1	none	79 ± 11_(*n*=13)_
2	Zn powder	43 ± 30_(*n*=3)_
3	[^18^F]**4** as reagent	31 ± 13_(*n*=6)_
4	[^18^F,D_2_]**3** as reagent	81 ± 4_(*n*=2)_
5	Mn	0_(*n*=1)_
6	80 °C	17 ± 10_(*n*=2)_
7	terpy	74 ± 10_(*n*=2)_
8	DMF	43 ± 29_(*n*=2)_
9	DMA	42 ± 34_(*n*=2)_
10	ArBr	72 ± 2_(*n*=2)_
11	ArCl	43 ± 12_(*n*=3)_
12	ArOTf	23 ± 2_(*n*=2)_

a RCC determined by radio-HPLC of crude reaction mixtures. Zn_*nano*_ = zinc nanopowder (40–60 nm avg. particle).

With optimized reaction conditions in hand, we investigated the reaction scope (Scheme 2). First, the protocol was applied using non-radioactive **3** to access ^19^F-fluoromethylated reference compounds in synthetically useful yields (**7**, **8**, **10**–**12**, **16**–**18**, **24**, **27**, **28**, **36**; 41–62%). As such, the synthesis of reference compounds required for evaluation of novel radioligands is straightforward, as both reference standard and radiolabeled compounds are conveniently accessible from the same (hetero)­aryl iodide starting material. In addition to model substrate **7**, arenes substituted at the *para*-position with various functional groups were well tolerated, including ester (e.g., [^18^F]**8**), nitrile, (e.g., [^18^F]**9**), ketone (e.g., [^18^F]**10**), amide (e.g., [^18^F]**11**), trifluoromethyl (e.g., [^18^F]**12**) and sulfone (e.g., [^18^F]**13**) functionality. A sterically hindered 2-iodo-1,1′-biphenyl precursor was amenable to radiolabeling, as well as naphthyl-derived substrates ([^18^F]**15**–[^18^F]**16**). Electron-rich and electron-deficient substrates were competent reaction partners ([^18^F]**17**, [^18^F]**18**). Notably, a primary tosylate, which could serve as a handle for conjugation, remained intact throughout the reaction ([^18^F]**19**), showcasing the orthogonality of this cross-coupling protocol with respect to the traditional S_N_2 radiolabeling approach with [^18^F]­fluoride. More complex *para*-substituted aryl bromides performed well ([^18^F]**20**–[^18^F]**22**). A substrate featuring an electron-deficient alkene, known to be a proficient acceptor of the fluoromethyl radical,^42^ was also well tolerated ([^18^F]**23**), highlighting the chemoselectivity of our technology.

**Scheme 2:**
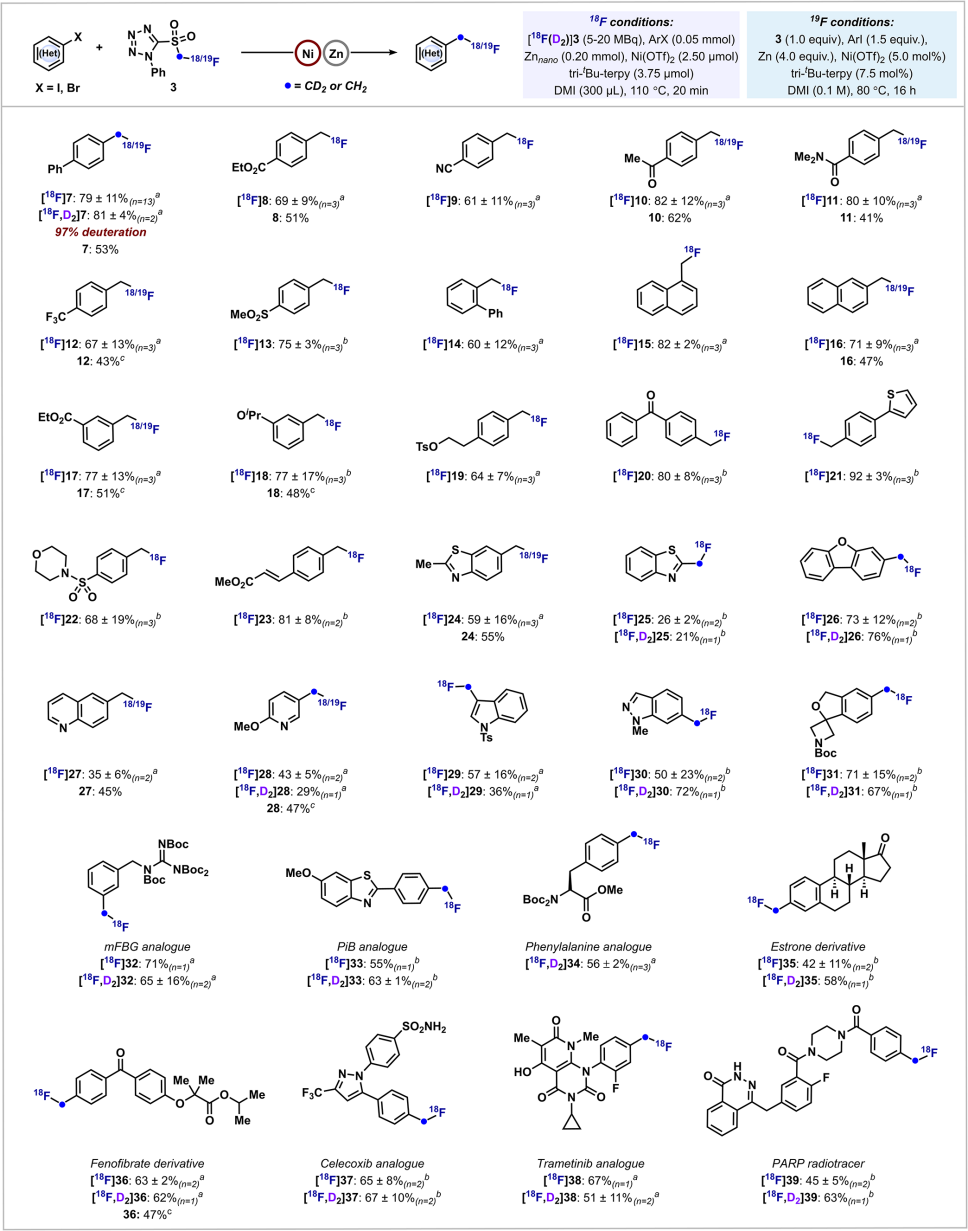
Substrate Scope^a^

a RCC determined by radio-HPLC. Zn_*nano*_ = zinc nanopowder (40–60 nm avg. particle size). ^*a*^From ArI. ^*b*^From ArBr. ^*c*^Determined by quantitative ^19^F NMR spectroscopy.

Reactivity further extended to a broad range of medicinally relevant hetero/spirocyclic substrates ([^18^F]**24**-[^18^F]**31**, RCCs 21%–72%). Radiotracer analogues of [^18^F]­mFBG and [^11^C]­PiB, used clinically for imaging the norepinephrine transporter^43^ and β-amyloid plaques,^44^ respectively, were prepared in good RCC ([^18^F,D_2_]**32**, [^18^F,D_2_]**33**). Analogues of phenylalanine ([^18^F,D_2_]**34**), estrone ([^18^F,D_2_]**35**), fenofibrate ([^18^F,D_2_]**36**) celecoxib ([^18^F,D_2_]**37**), trametinib ([^18^F,D_2_]**38**) and a PARP radiotracer ([^18^F,D_2_]**39**) were also within reach. Notably, [^18^F,D_2_]**37**, [^18^F,D_2_]**38** and [^18^F,D_2_]**39** contain nucleophilic functionalities, otherwise incompatible with electrophilic ^18^F-fluoromethyl reagents (e.g., [^18^F]­CH_2_FBr) and reactive benzylic radiolabeling precursors. This also presents the first example of ^18^F,D_2_-labeling of the celecoxib scaffold without modification or protection of the nucleophilic sulfonamide moiety.

Finally, to demonstrate the utility of this method to prepare deuterated ^18^F-radiotracers for preclinical imaging studies, reaction automation is a key consideration. A semiautomated radiosynthesis protocol was developed using a TRASIS AllinOne radiosynthesizer and applied to [^18^F,D_2_]**39**. This PARP radiotracer was selected because direct radiofluorination of an unstable chloromethyl radiolabeling precursor had been prohibitive, in addition to the reported *in vivo* radiodefluorination of [^18^F]**39**.^16^ In contrast, our technology gave access to [^18^F,D_2_]**39** from a stable aryl bromide and in an AY of up to 260 MBq (9% n.d.c. from [^18^F,D_2_]**3**, overall 1% n.d.c. from 40 GBq [^18^F]­fluoride) (Scheme 3). *A*_m_ reached 71 GBq μmol^–1^ and [^18^F,D_2_]**39** was obtained in high radiochemical purity (RCP) of >99%. While routine adoption of this method may warrant further development, this proof-of-concept result comprises the first report of a deuterated isotopolog of this radiotracer, and demonstrates this method can produce sufficient quantities of radiotracer for preclinical imaging studies.

**Scheme 3:**
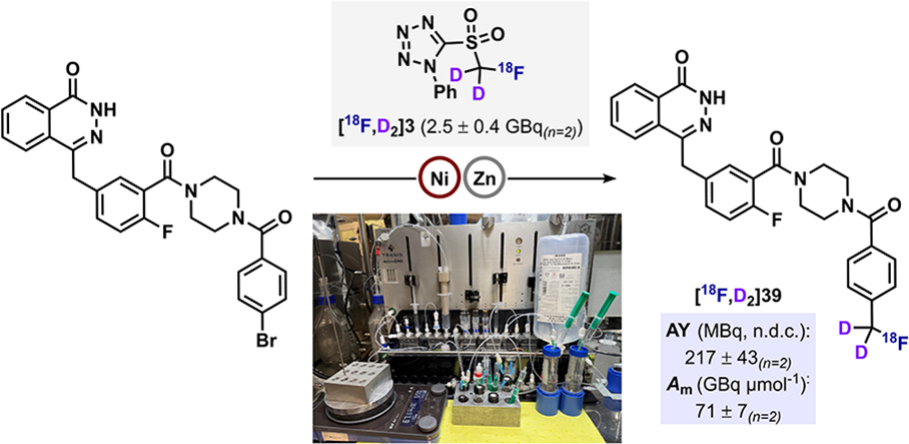
Scale-up and Isolation of PARP Radiotracer [^**18**^F,D_**2**_]­39^a^

a Conditions: aryl bromide (0.05 mmol), Zn_*nano*_ (0.20 mmol), Ni­(OTf)_2_ (2.50 μmol), tri-^*t*^Bu-terpy (3.75 μmol), 110 °C, 15 min, DMI. AY (n.d.c.) = Activity yield, non-decay corrected. *A*_m_ = molar activity, decay-corrected to the end-of-synthesis.

In conclusion, we have developed a novel radiochemical cross-coupling technology to address challenges reported by several groups in preparing novel ^18^F-fluoromethylated PET radiotracers, in particular deuterated variants. Our method obviates the time-consuming and complex syntheses of bespoke deuterated precursors bearing reactive benzylic leaving groups. This precursor class is known to suffer from poor stability, presenting a roadblock in evaluating these compounds as PET tracers. Specifically, we demonstrate that bench-stable (hetero)­aryl halides are easily converted into a broad range of complex and biologically relevant ^18^F,D_2_-fluoromethyl containing (hetero)­arenes from a common labeled reagent. This strategy not only provides such compounds for PET imaging but is also directly applicable to the preparation of ^19^F-reference compounds. Moreover, we anticipate the expedient access to deuterated PET tracer analogues will encourage radiochemists to consider ^18^F-fluoromethylated targets that may have otherwise been overlooked due to issues of *in vivo* radiodefluorination or precursor synthesis and instability.^12–25^

## Supplementary Material



## Notes

Preparation of starting materials and reference compounds; radiochemistry details; radio-HPLC traces; NMR spectra (PDF)

## Abbreviations

*A*_m_: molar activity
AY: activity yield
HPLC: high-performance liquid chromatography
n.d.c.: non-decay corrected
RCC: radiochemical conversion, as determined by radio-HPLC analysis of the crude reaction mixture
