# Supplementary material for: A Ni-Mediated Cross-Coupling Approach to Deuterated 18F- Fluoromethylated (Hetero)arenes
Source: J Am Chem Soc. 2026 Jul 3;148(27):28049–54. doi: 10.1021/jacs.6c09649 (PMC13383636; doi:10.1021/jacs.6c09649)
Supplement: Supplementary file 1 [file ja6c09649_si_001.pdf]

Supporting information for

A Ni-Mediated Cross-Coupling Approach to Deuterated ^{18}F-Fluoromethylated (Hetero)arenes

Maddison Lovell[†], Sebastiano Ortalli[†], Raquel Sánchez-Bento[‖], Matthew Tredwell[#,¶], Joseph Ford[†,*] and Véronique Gouverneur[†,*]

[†]Department of Chemistry, Chemistry Research Laboratory, University of Oxford, Mansfield Road, Oxford OX1 3TA, U.K.

[‖]MSD, Midland Road, London NW1 1AT, U.K.

[#]Wales Research and Diagnostic PET Imaging Centre, Cardiff University, University Hospital of Wales, Heath Park, Cardiff CF14 4XN, U.K.

[¶]School of Chemistry, Cardiff University, Main Building, Park Place, Cardiff, CF10 3AT, U.K.

* Correspondence should be addressed to joseph.ford@chem.ox.ac.uk and veronique.gouverneur@chem.ox.ac.uk

Contents

General Information **S3**

Non-Radioactive Experiments **S4**

Reaction Optimization S4

Synthesis of Starting Materials S11

Synthesis of Reference Compounds S23

Synthesis of [D_2]**1** S41

Cyclic Voltammetry S43

Radiochemistry **S46**

General Experimental Details S46

Screening of ^{18}F-Fluoromethyl Sulfone Reagent Precursor S49

Automated ^{18}F-Fluoromethyl Sulfone Reagent Radiosynthesis S50

Automated Radiosynthesis of [^{18}F(D_2)]**3** S50

Calculation of Molar Activity of [^{18}F,D_2]**3** S53

Automated Radiosynthesis of [^{18}F]**4** S55

Reaction Optimization S56

Determination of Deuterium Incorporation S59

Substrate Scope Limitations S62

Semi-Automated Radiosynthesis of [^{18}F(D_2)]**39** S63

Calculation of Molar Activity of [^{18}F,D_2]**39** S66

Determination of Residual Nickel Content in Isolated [^{18}F,D_2]**39** S68

Radio and UV HPLC Trace Overlays S69

References **S104**

NMR Spectra **S108**

General Information

Unless otherwise stated, all reactions were conducted under an atmosphere of nitrogen with anhydrous solvents using standard Schlenk techniques. Glassware was flame-dried and allowed to cool to room temperature under vacuum. Cooling of reaction mixtures to 0 °C was achieved using an ice-water bath. Cooling of reaction mixtures to -78 °C was achieved using an acetone-dry ice bath. Weighing was performed with a 4 or 5 decimal place balance. If activated before use, zinc was stirred in aqueous HCl (1 M) for 2 hours, followed by filtration and washing with H_2O, then EtOH, and Et_2O. The zinc powder was then dried under vacuum for 18 h. Anhydrous solvents were purified by filtration through dried alumina columns using the University of Oxford internal solvent drying system (Innovative Technology Inc. PS-400-7). Thin-layer chromatography (TLC) was performed on Merck silica gel 60 F_{254} pre-coated aluminium backed TLC sheets with visualization under a UV lamp (λ_{max} = 254 nm) and/or by staining with potassium permanganate. Flash column chromatography was performed using Merck silica gel (60, particle size 0.040–0.063 mm). All NMR spectra were recorded on Bruker AVIIIHD 400, Bruker NEO 400, AVIIIHD 500, Bruker AVIII 600, or Bruker NEO 600. All reported ^{1}H and ^{13}C chemical shifts (δ_H, δ_C) are referenced to the residual signal of deuterated solvents ($CDCl_3$: δ_H = 7.26 ppm, δ_C = 77.16 ppm; C_6D_6: δ_H = 7.16 ppm, δ_C = 128.06 ppm; ^{19}F chemical shifts (δ_F) are referenced externally to $CFCl_3$ (δ_F = 0.0 ppm). Chemical shifts (δ) are reported in parts per million (ppm) to the nearest 0.01 ppm for ^{1}H NMR, and 0.1 ppm for ^{13}C and ^{19}F NMR. Coupling constants (J) are reported in Hertz (Hz) and rounded to the nearest 0.1 Hz. Unless otherwise stated, ^{13}C spectra are ^{1}H decoupled and reported coupling constants for ^{13}C spectra correspond to ^{19}F–^{13}C heteronuclear coupling. Multiplicities are reported as followings: s (singlet), d (doublet), t (triplet), q (quartet), pent. (pentet), sext. (sextet), m (multiplet), br (broad signal). NMR spectra were processed with MestReNova 15.0.0. Infrared spectra were recorded as the neat compound (neat) or as an evaporated solution (thin layer film) using a Bruker Tensor 27 FT-IR spectrometer. Absorptions are reported in wavenumber (cm^{-1}). Melting points of solids were measured on either a Griffin apparatus or a Stuart SMP20 apparatus and are uncorrected. High resolution mass spectra (HRMS) were recorded on Thermo Exactive mass spectrometer, for electrospray ionization (ESI), or an Agilent 7200 Accurate Mass Q-TOF GC-MS connected to a 7890 GC system, for electron ionization (GC-EI). m/z values are reported in Daltons (Da) and high-resolution values are calculated to four decimal places from the molecular formula. Samples for mass spectroscopy were prepared in 1 mg/mL solution in MeCN or MeOH (HRMS-ESI). Compound names were generated by PerkinElmer ChemDraw Professional 23.0.1.10.

[^{18}F]Fluoride was produced in an IBA Cyclon 18/9 in PETIC (UK), or in a GE MINITrace Qilin cyclotron in OxIME (UK), using the ^{18}O(p,n)^{18}F reaction. All experiments were performed on Trasis AllinOne radiosynthesizer. All isolated activity yields are non-decay corrected (n.d.c.). All molar activities are decay corrected to the end of synthesis (EOS), unless stated otherwise.

Non-Radioactive Experiments

Reaction Optimization

General procedures for the optimization studies

To an oven-dried 1.75 mL glass vial equipped with a magnetic stir bar (see below for details regarding reaction set-up) were added fluoromethyl sulfone reagent (**1**–**5**, 0.050 mmol, 1.0 equiv.), aryl iodide (0.075 mmol, 1.5 equiv.), activated zinc powder, $NiOTf_2$ and ligand. Anhydrous DMI (given concentration) was then added and the reaction mixture was closed under air. The vial was wrapped with Parafilm and stirred at 80 °C, at 650 rpm, in an aluminium heating block for 16 h. The reaction mixture was cooled to room temperature, 4-fluoroanisole (internal standard, 10 µL, 0.088 mmol) was added and the reaction mixture was diluted with $CDCl_3$, filtered through cotton wool, and analyzed by quantitative ^{19}F NMR.

Reaction set-up:

A 1.75 mL screw-neck glass vial (11 mm diameter × 36 mm height) with plastic screw cap lid and a magnetic stir bar was used (pictured right).

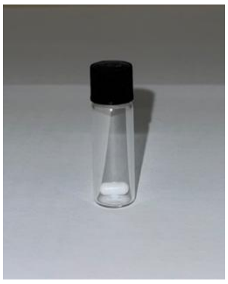

Due to the heterogenous nature of the reaction, efficient stirring was found to be crucial for reproducibility.

For optimal results, the magnetic stir bar should span most of the vial diameter; for example, 8 x 3 mm stir bars were found to be effective in this set-up.

Table S1. Sulfone screen.

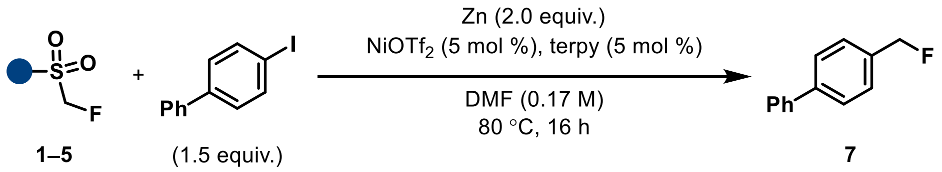

Reagent	E_{Red} (V vs SCE)	Sulfone remaining (%)	7 (%)
**1**	−2.00	89	0
**2**	−1.77	85	0
**3**	−1.42	5	18
**4**	−1.32	45	0
**5**	−0.86	43	0

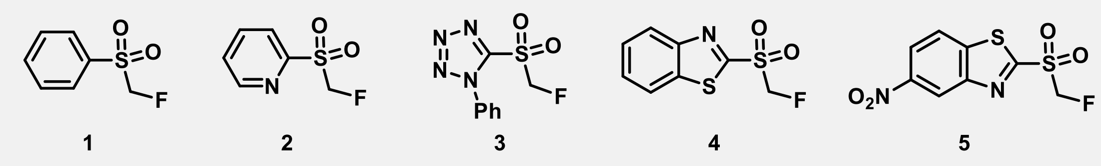

**terpy = 2,2′:6′,2″-terpyridine. E_{Red} = reduction potential. SCE = Saturated Calomel Electrode.*

Table S2. Zinc equivalents screen.

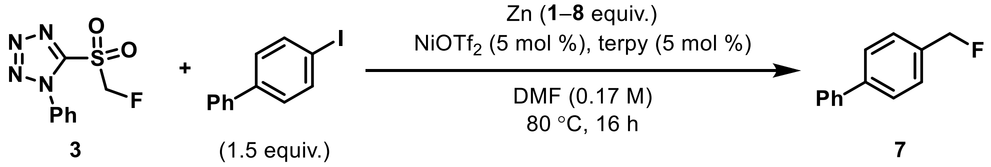

Zn (equiv.)	3 (%)	7 (%)
1.0	27	14
2.0	5	18
4.0	0	21
8.0	0	13

Table S3. Nickel and ligand loading screen.

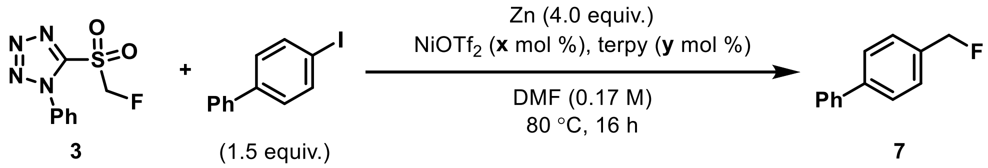

Ni (x, mol%)	terpy (y, mol%)	Ni:L	3 (%)	7 (%)
5	5	1:1	0	21
5	7.5	1:1.5	0	28
5	15	1:3	0	26
10	10	1:1	0	26
10	15	1:1.5	0	22

Table S4. Solvent screen.

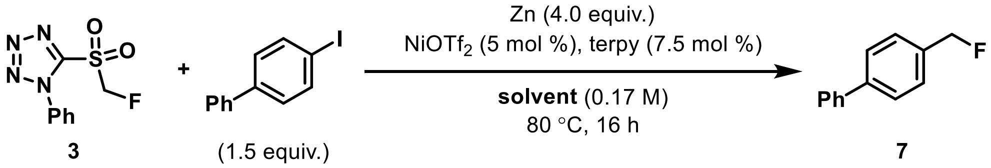

Solvent	3 (%)	7 (%)
DMF	0	28
DMA	0	32
DMI	0	32
NMP	0	20
MeCN	50	0
Toluene	94	0
DMSO	trace	20
1,4-dioxane	91	0

Table S5. Ligand screen

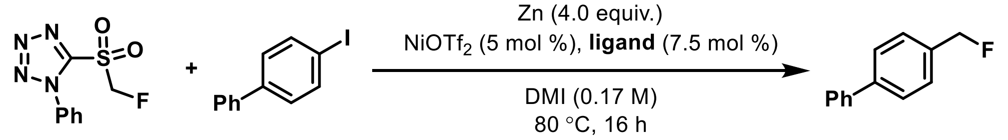

3 (1.5 equiv.) 7

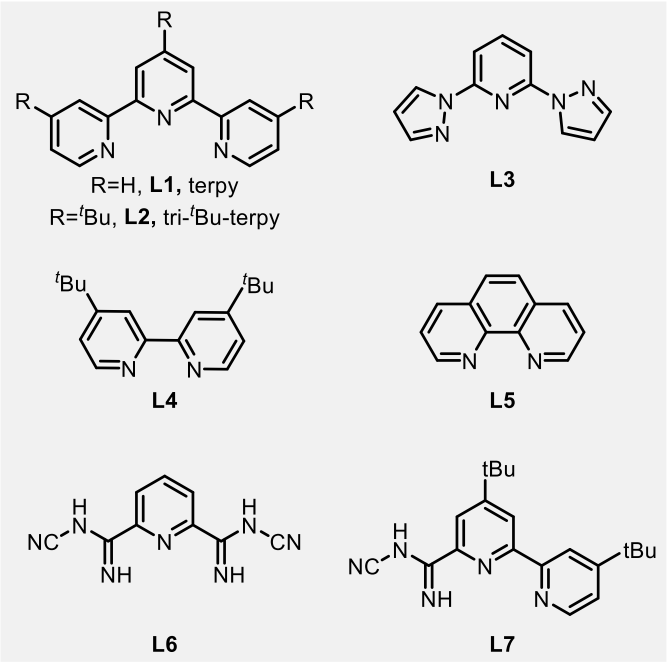

Ligand	3 (%)	7 (%)
**L1**	0	32
**L2**	0	45
**L2***	0	37
**L3**	5	trace
**L4**	15	trace
**L5**	11	0
**L6**	3	5
**L7**	25	6

**Using DMA as solvent.*

Table S6. Nickel source screen.

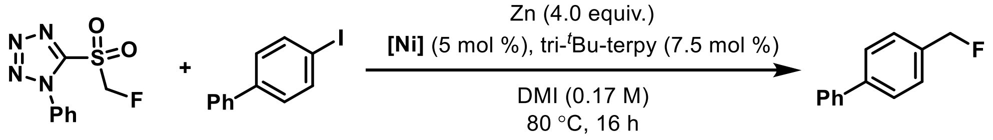

3 (1.5 equiv.) 7

[Ni]	3 (%)	7 (%)
$Ni(OTf)_2$	0	45
NiI_2	13	26
$Ni(acac)_2$	0	45
Ni(COD)(^{t}Bu-BQ)	0	40
$NiBr_2 \cdot DME$	0	47
$NiBr_2$	0	39

Table S7. Temperature screen.

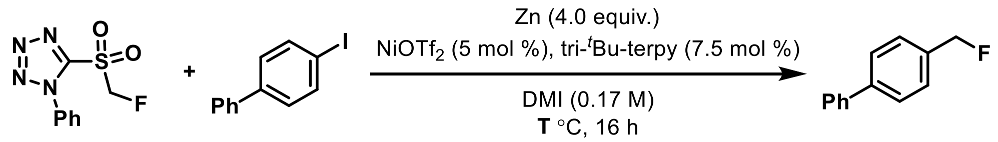

3 (1.5 equiv.) 7

T (°C)	3 (%)	7 (%)
r.t.	97	0
50	41	2
80	0	45
100	0	36

Table S8. Reductant screen.

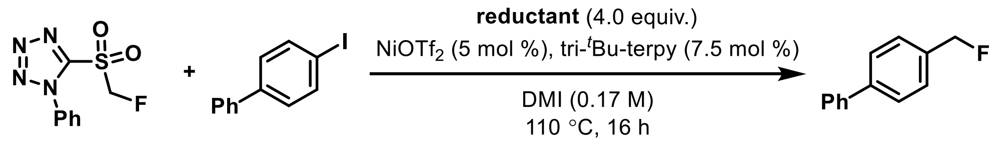

3 (1.5 equiv.) 7

Reductant	3 (%)	7 (%)
Zn	0	45
Zn*	0	45
Zn_{nano}	0	47
Mn	83	0
TDAE	58	0

Zn = zinc powder unactivated.* Zn_{nano} *= zinc nanopowder. TDAE = tetrakis(dimethylamino)ethylene.*

Table S9. Concentration screen.

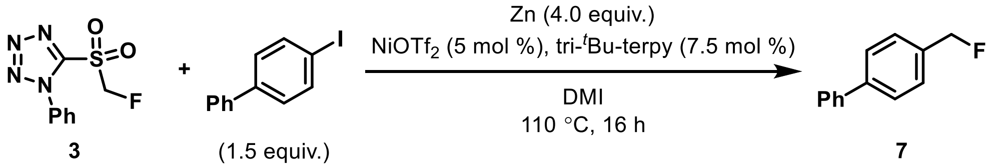

Concentration (M)	3 (%)	7 (%)
0.05	0	trace
0.067	0	46
0.1	0	52(53)
0.17	0	45
0.33	0	22
0.5	0	19

Isolated yield in parentheses, performed on 0.30 mmol scale.

Table S10. Re-evaluation of sulfones **1–5** under optimised conditions.

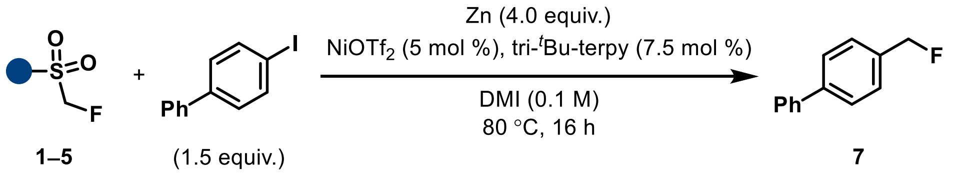

Reagent	E_{Red} (V vs SCE)	Sulfone remaining (%)	7 (%)
**1**	−2.00	89	0
**2**	−1.77	85	trace
**3**	−1.42	0	52(53)
**4**	−1.32	55	6
**5**	−0.86	76	0

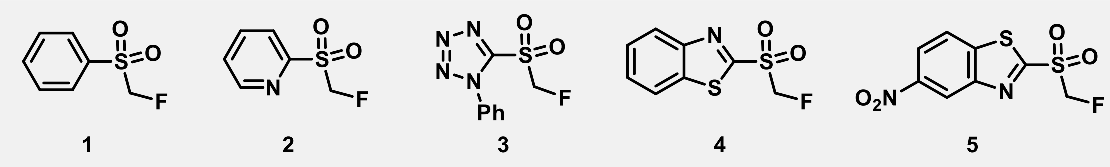

Isolated yield in parentheses, performed on 0.30 mmol scale.

Table S11. Control reactions.

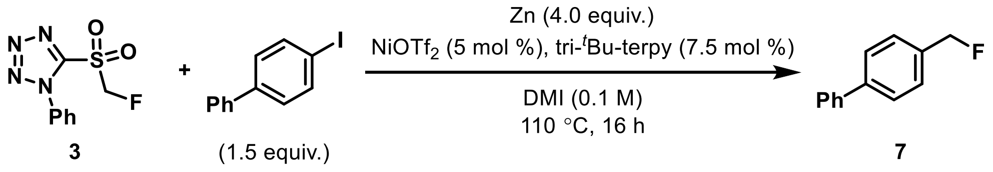

Deviation	3 (%)	7 (%)
**Under N_2 atmosphere**	0	49
**w/o Zn**	82	0
**w/o Ni**	23	0
**w/o ArI**	0	0
**w/o Ni/ligand or ArI**	0	0
**w/o Zn and ArI**	79	0
**w/o Zn and Ni**	91	0

w/o Ni specifies without $NiOTf_2$ and tri-^{t}Bu-terpy.

Table S12. Radical trapping experiments.

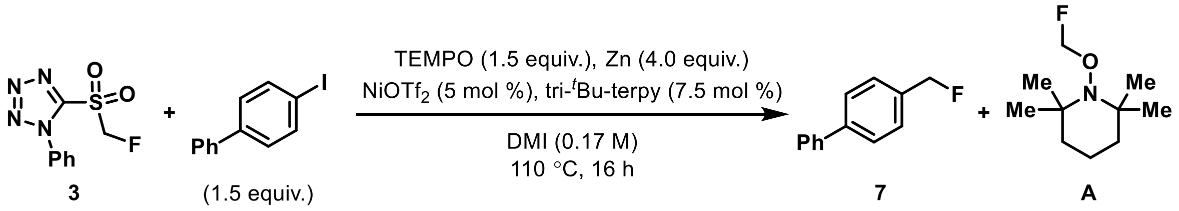

Deviation	3 (%)	7 (%)	A (%)
**–**	81	0	n.d
**No ArI, no Ni/L**	86	0	Detected by HRMS*
**No ArI**	83	0	n.d

**HRMS (ESI) m/z calculated for $C_{10}H_{21}FNO$ ($[M+H]^+$): 190.1602, found 190.1593. n.d. = not detected.*

Synthesis of Starting Materials

The following were purchased from commercial suppliers (ABCR, Acros Organics, Apollo Scientific, BLDpharm, Combi-Blocks, Fluorochem, Key Organics, Sigma Aldrich, TCI, Thermo Fisher Scientific) and used as received, without further purification: fluoroiodomethane, thiophenol, pyridine-2-thiol, 1-phenyl-1*H*-tetrazole-5-thiol, benzo[*d*]thiazole-2-thiol, 5-nitrobenzo[*d*]thiazole-2-thiol, diiodomethane, dibromomethane, dibromomethane-d_2 , [1,1'-biphenyl]-4-ol, 2-(4-iodophenyl)ethan-1-ol, 4-iodo-1,1'-biphenyl, 4-bromo-1,1'-biphenyl, 4-chloro-1,1'-biphenyl, ethyl 4-iodobenzoate, 4-iodobenzonitrile, 1-(4-iodophenyl)ethan-1-one, 4-iodo-*N*,*N*-dimethylbenzamide, 1-bromo-4-(methylsulfonyl)benzene, 2-iodo-1,1'-biphenyl, 1-iodonaphthalene, 2-iodonaphthalene, ethyl 3-iodobenzoate, 1-bromo-3-isopropoxybenzene, (4-bromophenyl)(phenyl)methanone, methyl (*E*)-3-(4-bromophenyl)acrylate, 2-(4-bromophenyl)thiophene, 4-((4-bromophenyl)sulfonyl)morpholine, 2-bromobenzo[*d*]thiazole, 6-iodo-2-methylbenzo[*d*]thiazole, 3-bromodibenzo[*b,d*]furan, 6-iodoquinoline, 5-iodo-2-methoxypyridine, 3-iodo-1-tosyl-1*H*-indole, 6-bromo-1-methyl-1*H*-indazole, *tert*-butyl 5'-bromo-3'*H*-spiro[azetidine-3,1'-isobenzofuran]-1-carboxylate, 3-iodobenzylamine hydrochloride, *N*,*N*'-bis(*tert*-butoxycarboyl)-*N*''-triflylguanidine, (4-bromophenyl)boronic acid, 2-bromo-6-methoxybenzo[*d*]thiazole, methyl (*R*)-2-((*tert*-butoxycarbonyl)amino)-3-(4-iodophenyl)propanoate, estrone, fenofibrate, 4-hydrazineylbenzenesulfonamide hydrochloride, 1-(4-bromophenyl)-4,4,4-trifluorobutane-1,3-dione, 3-cyclopropyl-1-(2-fluoro-4-iodophenyl)-5-hydroxy-6,8-dimethylpyrido[2,3-*d*]pyrimidine-2,4,7(1*H*,3*H*,8*H*)-trione, *tert*-butyl piperazine-1-carboxylate, methyl 2-(5-methoxy-2-methyl-1*H*-indol-3-yl)acetate 2-bromobenzoic acid, 2-(3-bromophenyl)ethan-1-amine, *N*-(4-iodophenyl)-5-nitrofuran-2-carboxamide, 7-bromo-5*H*-pyrido[4,3-*b*]indole, 2-(4-bromophenyl)pyridine, 3,5-dibromobenzonitrile, 2-ethynylpyridine, 2-bromothiazole-4-carbaldehyde, 3-fluoro-5-iodobenzonitrile.

General Procedure 1 (GP1):

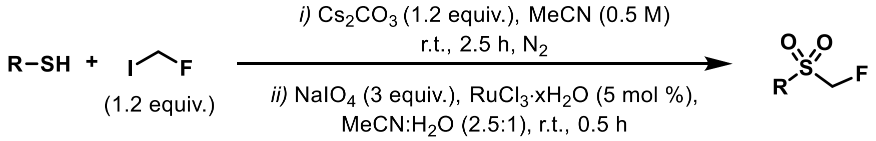

<u>Step 1</u>: The following is a literature procedure.[1] To a flame-dried round bottom flask equipped with a magnetic stir bar were added thiol (1.0 equiv.) and Cs_2CO_3 (1.2 equiv.) The flask was evacuated and backfilled with nitrogen three times prior to the addition of anhydrous MeCN (0.5 M). Fluoroiodomethane (1.2 equiv.) was added dropwise, and the reaction mixture was stirred under nitrogen at room temperature for 2.5 h. Upon completion, the reaction was quenched by the addition of H_2O, and extracted with Et_2O twice. The combined organic layers were dried over anhydrous Na_2SO_4, filtered, and concentrated under the flow of nitrogen gas. The crude product was used directly in the next step, assuming quantitative conversion.

<u>Step 2</u>: The following is an adapted literature procedure.[2,3] The crude product from step 1 was suspended in MeCN (0.4 M) and $NaIO_4$ (3.0 equiv.), $RuCl_3{\cdot}xH_2O$ (5.0 mol%) and H_2O (1.0 M) were added. The reaction mixture

was stirred at room temperature under air for 0.5 h. Upon completion, the reaction mixture was filtered through a pad of silica, washing with EtOAc. The filtrate was washed with brine, dried over anhydrous Na_2SO_4, filtered and concentrated *in vacuo*. The crude product was purified by silica gel column chromatography.

((Fluoromethyl)sulfonyl)benzene (1)

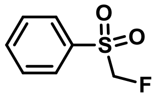

The title compound was prepared according to **GP1** using thiophenol (102 µL, 1.00 mmol). Purification by silica gel column chromatography (5% EtOAc in pentane) afforded the desired product as a white solid (89 mg, 0.511 mmol, 51%). **^{1}H NMR** (600 MHz, $CDCl_3$) δ 7.99 – 7.95 (m, 2H), 7.76 – 7.70 (m, 1H), 7.64 – 7.59 (m, 2H), 5.13 (d, *J* = 47.0 Hz, 2H); **^{19}F NMR** (377 MHz, $CDCl_3$) δ −210.67 (t, *J* = 47.3 Hz); **^{13}C NMR** (151 MHz, $CDCl_3$) δ 136.1, 135.0, 129.7, 129.2, 92.1 (d, *J* = 220.4 Hz); **HRMS** (GC-EI) *m/z* calculated for $C_7H_7O_2SF$ ($[M]^+$): 174.0145, found 174.0149. All spectroscopic data were in accordance with the literature.[4]

2-((Fluoromethyl)sulfonyl)pyridine (2)

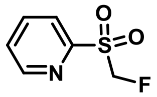

The title compound was prepared according to **GP1** using pyridine-2-thiol (222 mg, 2.00 mmol). Purification by silica gel column chromatography (25% EtOAc in pentane) afforded the desired product as a white solid (154 mg, 0.879 mmol, 44%). **^{1}H NMR** (400 MHz, $CDCl_3$) δ 8.80 (ddd, *J* = 4.7, 1.7, 0.9 Hz, 1H), 8.18 (dt, *J* = 7.9, 1.1 Hz, 1H), 8.03 (td, *J* = 7.8, 1.7 Hz, 1H), 7.63 (ddd, *J* = 7.7, 4.7, 1.2 Hz, 1H), 5.54 (d, *J* = 47.0 Hz, 2H); **^{19}F NMR** (377 MHz, $CDCl_3$) δ −213.57 (t, *J* = 47.0 Hz); **^{13}C NMR** (101 MHz, $CDCl_3$) δ 154.6, 150.8, 138.6, 128.3, 124.1, 88.7 (d, *J* = 219.9 Hz); **HRMS** (ESI) *m/z* calculated for $C_6H_7NO_2SF$ ($[M+H]^+$): 176.0176, found 176.0173. All spectroscopic data were in accordance with the literature.[2]

5-((Fluoromethyl)sulfonyl)-1-phenyl-1*H*-tetrazole (3)

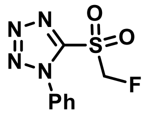

The title compound was prepared according to **GP1** using 1-phenyl-1*H*-tetrazole-5-thiol (356 mg, 2.00 mmol). Purification by silica gel column chromatography (10 to 30% EtOAc in pentane) afforded the desired product as a white solid (251 mg, 1.04 mmol, 52%). **^{1}H NMR** (400 MHz, $CDCl_3$) δ 7.70 – 7.61 (m, 5H), 5.75 (d, *J* = 46.3 Hz, 2H); **^{19}F NMR** (377 MHz, $CDCl_3$) δ −208.89 (t, *J* = 46.3 Hz); **^{13}C NMR** (126 MHz, $CDCl_3$) δ 151.8, 132.8, 132.0, 130.0, 125.3, 91.8 (d, *J* = 227.6 Hz); **HRMS** (ESI) *m/z* calculated for $C_8H_8N_4O_2SF$ ($[M+H]^+$): 243.0347, found 243.0348. All spectroscopic data were in accordance with the literature.[3]

2-((Fluoromethyl)sulfonyl)benzo[*d*]thiazole (4)

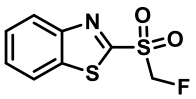

The title compound was prepared according to **GP1** using benzo[*d*]thiazole-2-thiol (334 mg, 2.00 mmol). Purification by silica gel column chromatography (20 to 30% EtOAc in pentane) afforded the desired product as a white solid (315 mg, 1.36 mmol, 68%). **^{1}H NMR** (400 MHz, $CDCl_3$) δ 8.28 – 8.26 (m, 1H), 8.07 – 8.04 (m, 1H), 7.70 – 7.62 (m, 2H), 5.59 (d, *J* = 46.8 Hz, 2H); **^{19}F NMR** (377 MHz, $CDCl_3$) δ −210.83 (t, *J* = 46.9 Hz); **^{13}C NMR** (101 MHz, $CDCl_3$) δ 162.4, 152.9, 137.5, 128.7, 128.1, 126.0, 122.6, 90.8 (d, *J* = 223.5 Hz); **HRMS** (ESI) *m/z* calculated for $C_8H_7NO_2S_2F$ ($[M+H]^+$): 231.9897, found 231.9898. All spectroscopic data were in accordance with the literature.[3]

2-((Fluoromethyl)sulfonyl)-5-nitrobenzo[*d*]thiazole (5)

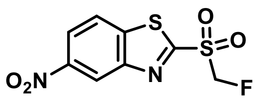

The title compound was prepared according to **GP1** using 5-nitrobenzo[*d*]thiazole-2-thiol (424 mg, 2.00 mmol). Purification by silica gel column chromatography (10 to 40% EtOAc in pentane) afforded the desired product as a white solid (173 mg, 0.626 mmol, 31%). **^{1}H NMR** (600 MHz, $CDCl_3$) δ 9.12 (d, *J* = 2.1 Hz, 1H), 8.51 (dd, *J* = 8.9, 2.2, 1H), 8.23 (d, *J* = 9.0 Hz, 1H), 5.65 (d, *J* = 46.7 Hz, 2H); **^{19}F NMR** (377 MHz, $CDCl_3$) δ −210.82 (t, *J* = 46.7 Hz); **^{13}C NMR** (151 MHz, $CDCl_3$) δ 166.8, 152.4, 148.0, 142.9, 123.6, 122.8, 121.5, 90.6 (d, *J* = 224.5 Hz); **HRMS** (ESI) *m/z* calculated for $C_8H_5FN_2O_4S_2K$ ($[M+K]^+$): 314.9306, found 314.9305; **IR** (neat) 2923, 1513, 1465, 1342, 1157, 1074, 945, 915, 826, 764, 742, 726, 704; **m.p.** 177–178 °C.

5-((Iodomethyl)thio)-1-phenyl-1*H*-tetrazole (s1)

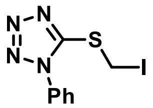

To a flame-dried round bottom flask equipped with a magnetic stir bar was added Cs_2CO_3 (782 mg, 2.40 mmol, 1.2 equiv.) The flask was evacuated and backfilled with nitrogen three times prior to the addition of anhydrous MeCN (20 mL) and diiodomethane (3.23 mL, 40.0 mmol, 20 equiv.). The flask was then cooled to 0 °C and a solution of 1-phenyl-1*H*-tetrazole-5-thiol (356 mg, 2.00 mmol, 1.0 equiv.) in MeCN (6 mL) was added dropwise over 10 minutes whilst stirring at the same temperature. The reaction mixture was then stirred under nitrogen at 0 °C for a further 0.5 h. The reaction was quenched by the addition of H_2O (10 mL), and extracted with Et_2O (3 x 20 mL). The combined organic layers were dried over anhydrous Na_2SO_4, filtered, and concentrated *in vacuo*. Purification by silica gel column chromatography (5 to 30% EtOAc in pentane) afforded the desired product as a yellow solid (292 mg, 0.918 mmol, 46%). **^{1}H NMR** (400 MHz, $CDCl_3$) δ 7.66 – 7.45 (m, 5H), 4.81 (s, 2H); **^{13}C NMR** (151 MHz, $CDCl_3$) δ 152.9, 133.3, 130.7, 130.1, 124.0, −7.1; **HRMS** (ESI) *m/z* calculated for $C_8H_7IN_4SNa$ ($[M+Na]^+$): 340.9328, found 340.9321. All spectroscopic data were in accordance with the literature.[5]

5-((Bromomethyl)thio)-1-phenyl-1*H*-tetrazole (s2)

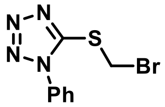

To a flame-dried round bottom flask equipped with a magnetic stir bar was added Cs_2CO_3 (782 mg, 2.40 mmol, 1.2 equiv.) The flask was evacuated and backfilled with nitrogen three times prior to the addition of anhydrous MeCN (30 mL) and dibromomethane (5.6 mL, 80.0 mmol, 40 equiv.). The flask was then cooled to 0 °C and a solution of 1-phenyl-1*H*-tetrazole-5-thiol (356 mg, 2.00 mmol, 1.0 equiv.) in MeCN (10 mL) was added dropwise *via* syringe pump over 3 h, whilst keeping the reaction mixture at 0 °C. The reaction mixture was then stirred under nitrogen at 0 °C for a further hour. The reaction was quenched by the addition of H_2O (10 mL), and extracted with Et_2O (3 x 30 mL). The combined organic layers were dried over anhydrous Na_2SO_4, filtered, and concentrated *in vacuo*. Purification by silica gel column chromatography (20% EtOAc in pentane) afforded the desired product as a white solid (116 mg, 0.428 mmol, 21%). **^{1}H NMR** (600 MHz, $CDCl_3$) δ 7.62 – 7.53 (m, 5H), 5.17 (s, 2H); **^{13}C NMR** (151 MHz, $CDCl_3$) δ 151.8, 133.3, 130.7, 130.2, 124.1, 29.1; **HRMS** (ESI) *m/z* calculated for $C_8H_8BrN_4S$ ($[M+H]^+$): 270.9648, found 270.9643; **IR** (neat) 3043, 1499, 1462, 1421, 1248, 1211, 992, 761, 715, 684; **m.p.** 85–86 °C.

5-((Bromomethyl-*d*$_2$)thio)-1-phenyl-1*H*-tetrazole ([D$_2$]s2)

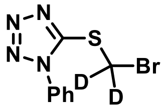

To a round bottom flask equipped with a magnetic stir bar were added 1-phenyl-1*H*-tetrazole-5-thiol (178 mg, 1.00 mmol, 1.0 equiv.) and K_2CO_3 (691 mg, 5.00 mmol, 5.0 equiv.) under air. Acetone (10 mL) and dibromomethane-*d*$_2$ (700 µL, 9.97 mmol, 10 equiv.) were added sequentially and the reaction mixture was heated to reflux whilst stirring for 2 h. The reaction mixture was cooled to room temperature, diluted with H_2O (30 mL) and extracted with Et_2O (3 x 30 mL). The combined organic layers were dried over anhydrous Na_2SO_4, filtered, and concentrated *in vacuo*. Purification by silica gel column chromatography (20% EtOAc in pentane) afforded the desired product as a white solid (76 mg, 0.278 mmol, 28%). **^{1}H NMR** (400 MHz, $CDCl_3$) δ 7.62 – 7.53 (m, 5H); **^{13}C NMR** (101 MHz, $CDCl_3$) δ 151.8, 133.2, 130.7, 130.1, 124.1, 28.9 (p, J = 25.7 Hz); **HRMS** (ESI) *m/z* calculated for $C_8H_6D_2BrN_4S$ ($[M+H]^+$): 272.9773, found 272.9767; **IR** (neat) 2360, 1499, 1421, 1387, 1248, 916, 761, 657; **m.p.** 82–84 °C.

((1-Phenyl-1*H*-tetrazol-5-yl)thio)methyl 4-methylbenzenesulfonate (s3)

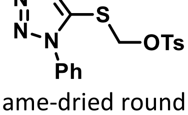

The following is a literature procedure.[6] To a flame-dried round bottom flask equipped with a magnetic stir bar were added AgOTs (163 mg, 0.585 mmol, 1.3 equiv) and 5-((iodomethyl)thio)-1-phenyl-1*H*-tetrazole **s1** (143 mg, 0.449 mmol, 1.0 equiv.). The flask was evacuated and backfilled with nitrogen three times prior to the addition of anhydrous MeCN (3 mL). The reaction mixture was then stirred under nitrogen at room temperature for 16

h. The reaction mixture was then filtered through a pad of celite and washed with MeCN and the filtrate was concentrated *in vacuo*. Purification by silica gel column chromatography (30 to 40% EtOAc in pentane) afforded the desired product as a white solid (135 mg, 0.372 mmol, 83%). **^{1}H NMR** (600 MHz, $CDCl_3$) δ 7.76 (d, J = 8.4 Hz, 2H), 7.58 – 7.55 (m, 3H), 7.47 – 7.44 (m, 2H), 7.32 (d, J = 8.0 Hz, 2H), 5.89 (s, 2H), 2.44 (s, 3H); **^{13}C NMR** (151 MHz, $CDCl_3$) δ 151.2, 145.8, 133.2, 132.9, 130.7, 130.1, 130.1, 128.2, 124.1, 69.9, 21.9; **HRMS** (ESI) *m/z* calculated for $C_{15}H_{14}N_4O_3S_2Na$ ($[M+Na]^+$): 385.0400, found 385.0392; **IR** (neat) 1499, 1365, 1175, 1094, 951, 817, 756, 703; **m.p.** 93–94 °C.

2-((Iodomethyl)sulfonyl)benzo[*d*]thiazole (s4)

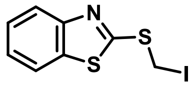

To a flame-dried round bottom flask equipped with a magnetic stir bar was added Cs_2CO_3 (391 mg, 1.20 mmol, 1.2 equiv). The flask was evacuated and backfilled with nitrogen three times prior to the addition of anhydrous MeCN (10 mL) and diiodomethane (1.61 mL, 19.9 mmol, 20 equiv). A solution of 2-benzo[*d*]thiazole-2-thiol (167 mg, 1.00 mmol, 1.0 equiv) in anhydrous MeCN (5 mL) was then added dropwise at 0 °C under nitrogen over 10 minutes. The reaction mixture was then stirred at 0 °C for a further 30 minutes and then diluted with H_2O (10 mL) and brine (5 mL). The aqueous layer was extracted with Et_2O (3 x 20 mL), and the combined organic layers were dried over anhydrous Na_2SO_4, filtered, and concentrated *in vacuo*. Purification by silica gel column chromatography (0 to 3% EtOAc in pentane) afforded the desired product as a yellow solid (48 mg, 0.156 mmol, 16%). **^{1}H NMR** (400 MHz, $CDCl_3$) δ 7.98 (d, J = 8.1 Hz, 1H), 7.82 (d, J = 7.9 Hz, 1H), 7.52 – 7.45 (m, 1H), 7.41 – 7.31 (m, 1H), 4.90 (s, 2H); **^{13}C NMR** (101 MHz, $CDCl_3$) δ 163.7, 153.1, 135.6, 126.5, 125.0, 122.4, 121.4, −3.5; **HRMS** (ESI) *m/z* calculated for $C_8H_7INS_2$ ($[M+H]^+$): 307.9059, found 307.9043; **IR** (neat) 2920, 1456, 1425, 1261, 1136, 1021, 798, 757, 725; **m.p.** 70–73 °C.

[1,1'-Biphenyl]-4-yl trifluoromethanesulfonate (s5)

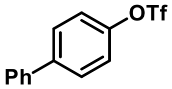

Synthesized according to a literature procedure.[7] All spectroscopic data were in accordance with the literature.[7]

1-Iodo-3-isopropoxybenzene (s6)

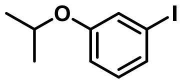

Synthesized according to a literature procedure.[8] All spectroscopic data were in accordance with the literature.[8]

4-Iodophenethyl 4-methylbenzenesulfonate (s7)

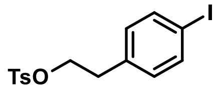

Synthesized according to a literature procedure.[9] All spectroscopic data were in accordance with the literature.[10]

Methyl (*R*)-2-(bis(*tert*-butoxycarbonyl)amino)-3-(4-iodophenyl)propanoate (s8)

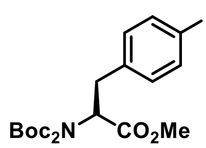

Synthesized according to a literature procedure.[11] All spectroscopic data were in accordance with the literature.[11]

***N*',*N*''-Bis(*tert*-butoxycarbonyl)-*N*-(3-iodobenzyl)guanidine (s9)**

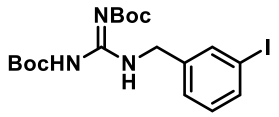

The following is an adapted literature procedure.[12] To a stirring solution of 3-iodobenzylamine hydrochloride (539 mg, 2.00 mmol, 1.0 equiv.) in CH_2Cl_2 (19 mL) was added triethylamine (1.39 mL, 9.97 mmol, 5.0 equiv.). After stirring at room temperature for 5 minutes, *N*,*N*′-bis(*tert*-butoxycarboyl)-*N*′′-triflylguanidine (783 mg, 2.00 mmol, 1.0 equiv.) was added and the reaction mixture was stirred at room temperature for 30 minutes. The reaction mixture was diluted with H_2O (20 mL) and extracted with CH_2Cl_2 (3 x 20 mL). The combined organic layers were washed with H_2O (2 x 20 mL) and brine (20 mL), were dried over anhydrous Na_2SO_4, filtered, and concentrated *in vacuo* affording the desired product as a white solid (919 mg, 1.93 mmol, 97%). **^{1}H NMR** (400 MHz, $CDCl_3$) δ 11.53 (s, 1H), 8.58 (t, J = 5.4 Hz, 1H), 7.66 (m, 1H), 7.62 (dt, J = 7.7, 1.4 Hz, 1H), 7.29 – 7.26 (m, 1H), 7.07 (t, J = 7.8 Hz, 1H), 4.57 (d, J = 5.4 Hz, 2H), 1.51 (s, 9H), 1.48 (s, 9H); **^{13}C NMR** (126 MHz, $CDCl_3$) δ 163.7, 156.3, 153.3, 139.9, 137.1, 136.8, 130.6, 127.2, 94.7, 83.5, 79.6, 44.2, 28.4, 28.2; **HRMS** (ESI) *m/z* calculated for $C_{18}H_{26}IN_3O_4Na$ ($[M+Na]^+$): 498.0860, found 498.0844. All spectroscopic data were in accordance with the literature.[12]

***N*,*N*',*N*''-Tetra-*tert*-butyloxycarbonyl-*N*-(3-iodobenzyl)guanidine (s10)**

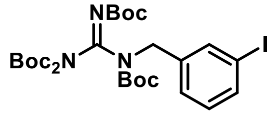

Synthesized according to a literature procedure from *N*',*N*''-bis(*tert*-butoxycarbonyl)-*N*-(3-iodobenzyl)guanidine **s9**.[12] All spectroscopic data were in accordance with the literature.[12]

(8*R*,9*S*,13*S*,14*S*)-13-Methyl-17-oxo-7,8,9,11,12,13,14,15,16,17-decahydro-6*H*-cyclopenta[*a*]phenanthren-3-yl trifluoromethanesulfonate (s11)

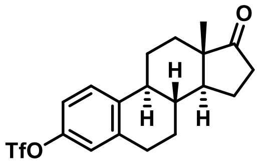

Synthesized according to a literature procedure.[13] All spectroscopic data were in accordance with the literature.[13]

(8*R*,9*S*,13*S*,14*S*)-13-Methyl-3-(4,4,5,5-tetramethyl-1,3,2-dioxaborolan-2-yl)-6,7,8,9,11,12,13,14,15,16-decahydro-17*H*-cyclopenta[*a*]phenanthren-17-one (s12)

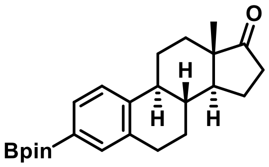

The following is an adapted literature procedure.[14] To a flame-dried Schlenk flask equipped with a stir bar were added estrone trifluoromethanesulfonate **s11** (1.006 g, 2.50 mmol, 1.0 equiv), Pd(dppf)Cl_2•CH_2Cl_2 (102 mg, 0.125 mmol, 0.050 equiv.), B_2pin_2 (698 mg, 2.75 mmol, 1.1 equiv.) and KOAc (736 mg, 7.50 mmol, 3.0 equiv.). The flask was evacuated and backfilled with nitrogen three times prior to the addition of anhydrous 1,4-dioxane (10 mL). The reaction mixture was heated to 100 °C whilst stirring under nitrogen for 12 h. The reaction mixture was then cooled to room temperature, diluted with EtOAc, filtered through a pad of celite and Na_2SO_4 and the filtrate was concentrated *in vacuo*. Purification by silica gel column chromatography (0 to 10% EtOAc in pentane) afforded the desired product as a white solid (804 mg, 2.11 mmol, 84%). **^{1}H NMR** (400 MHz, $CDCl_3$) δ 7.60 (d, J = 7.8 Hz, 1H), 7.57 (s, 1H), 7.32 (d, J = 7.7 Hz, 1H), 2.97 – 2.89 (m, 2H), 2.57 – 2.40 (m, 2H), 2.33 (td, J = 10.9, 4.2 Hz, 1H), 2.24 – 1.88 (m, 4H), 1.73 – 1.39 (m, 6H), 1.34 (s, 12H), 0.91 (s, 3H); **^{13}C NMR** (101 MHz, $CDCl_3$) δ 221.0, 143.3, 136.0, 135.7, 132.3, 124.9, 83.8, 50.7, 48.1, 44.9, 38.2, 36.0, 31.7, 29.3, 26.6, 25.7, 25.0, 24.9, 21.7, 14.0. *Note:* C(Ar)-B was not observed; **HRMS** (ESI) *m/z* calculated for $C_{24}H_{33}BO_3Na$ ($[M+Na]^+$): 403.2415, found 403.2404. All spectroscopic data were in accordance with the literature.[14]

(8*R*,9*S*,13*S*,14*S*)-3-Bromo-13-methyl-6,7,8,9,11,12,13,14,15,16-decahydro-17*H*-cyclopenta[*a*]phenanthren-17-one (s13)

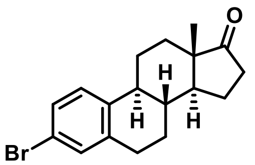

Synthesized according to a literature procedure from estrone boronic acid pinacol ester **s12**.[15] All spectroscopic data were in accordance with the literature.[15]

Isopropyl 2-methyl-2-(4-(4-(4,4,5,5-tetramethyl-1,3,2-dioxaborolan-2-yl)benzoyl)phenoxy)propanoate (s14)

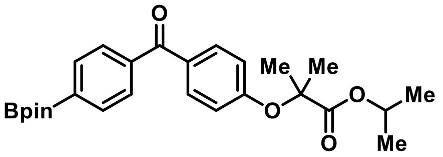

Synthesized according to a literature procedure.[16] All spectroscopic data were in accordance with the literature.[17]

(4-(4-((1-Isopropoxy-2-methyl-1-oxopropan-2-yl)oxy)benzoyl)phenyl)boronic acid (s15)

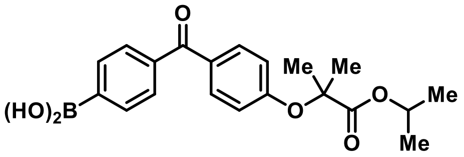

Synthesized according to a literature procedure from fenofibrate boronic acid pinacol ester **s14**.[17] All spectroscopic data were in accordance with the literature.[18]

Isopropyl 2-(4-(4-iodobenzoyl)phenoxy)-2-methylpropanoate (s16)

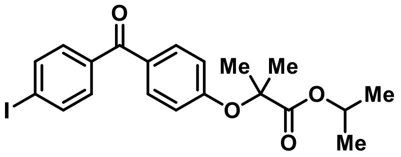

Synthesized according to a literature procedure from fenofibrate boronic acid **s15**.[19] All spectroscopic data were in accordance with the literature.[19]

4-(5-(4-Bromophenyl)-3-(trifluoromethyl)-1*H*-pyrazol-1-yl)benzenesulfonamide (s17)

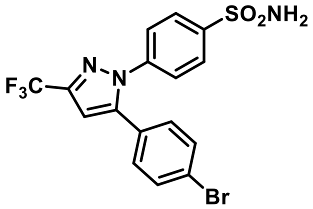

Synthesized according to a literature procedure.[20] All spectroscopic data were in accordance with the literature.[20]

2-(4-Bromophenyl)-6-methoxybenzo[*d*]thiazole (s18)

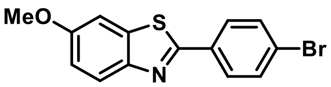

Synthesized according to a literature procedure.[21] All spectroscopic data were in accordance with the literature.[21]

tert-Butyl 4-(4-bromobenzoyl)piperazine-1-carboxylate (s19)

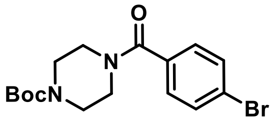

The following is an adapted literature procedure.[22] 4-Bromobenzoic acid (603 mg, 3.00 mmol, 1.0 equiv.), *tert*-butyl piperazine-1-carboxylate (559 mg, 3.00 mmol, 1.0 equiv.), EDCI•HCl (748 mg, 3.90 mmol, 1.3 equiv.) and DMAP (220 mg, 1.80 mmol, 0.60 equiv.) were added to a flame-dried flask. The flask was evacuated and backfilled with nitrogen three times prior to the addition of anhydrous CH_2Cl_2 (30 mL). The reaction mixture was stirred under nitrogen at room temperature for 15 h. The reaction mixture was then diluted with H_2O (30 mL) and extracted with extracted with EtOAc (2 x 30 mL). The combined organic layers were dried over anhydrous Na_2SO_4, filtered, and concentrated *in vacuo*. Purification by silica gel column chromatography (10 to 20% EtOAc in pentane) afforded the desired product as a white solid (1.02 g, 2.76 mmol, 92%). **^{1}H NMR** (600 MHz, $CDCl_3$) δ 7.58 – 7.52 (m, 2H), 7.30 – 7.26 (m, 2H), 3.90 – 3.19 (m, 8H), 1.46 (s, 9H); **^{13}C NMR** (151 MHz, $CDCl_3$) δ 169.7, 154.7, 134.4, 132.0, 128.9, 124.4, 80.6, 47.7, 43.8, 42.3, 28.5; **HRMS** (ESI) *m/z* calculated for $C_{16}H_{22}BrN_2O_3$ ($[M+H]^+$): 369.0808, found 369.0802. All spectroscopic data were in accordance with the literature.[23]

(4-Bromophenyl)(piperazin-1-yl)methanone (s20)

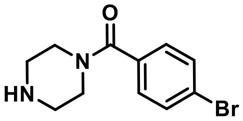

The following is an adapted literature procedure.[24] To a stirring solution of *tert*-butyl 4-(4-bromobenzoyl)piperazine-1-carboxylate **s19** (554 mg, 1.50 mmol, 1.0 equiv.) in THF (3 mL) at room temperature was added 4 M HCl in 1,4-dioxane (3.75 mL, 15.0 mmol, 10 equiv.). The reaction mixture was stirred at room temperature for 12 h, followed by concentration under the flow of nitrogen gas. The residue was dissolved in CH_2Cl_2 (15 mL) and washed with a 10% aqueous K_2CO_3 solution (2 x 15 mL) and then H_2O (15 mL). The organic layer was dried over anhydrous Na_2SO_4, filtered, and concentrated *in vacuo*, affording the desired product as a white solid (400 mg, 1.49 mmol, 99%). **^{1}H NMR** (600 MHz, DMSO-d_6) δ 7.67 – 7.61 (m, 2H), 7.36 – 7.31 (m, 2H), 3.52 (br s, 2H), 3.21 (br s, 2H), 2.72 (br, s, 2H), 2.63 (br, s, 2H); **^{13}C NMR** (151 MHz, DMSO-d_6) δ 167.9, 135.3, 131.4, 129.1, 122.6, 66.3, 48.5 (br), 45.6 (br d, J = 68.6 Hz), 42.8 (br); **HRMS** (ESI) *m/z* calculated for $C_{11}H_{14}BrN_2O$ ($[M+H]^+$): 269.0284, found 269.0278. All spectroscopic data were in accordance with the literature.[24]

2-Fluoro-5-((4-oxo-3,4-dihydrophthalazin-1-yl)methyl)benzoic acid (s21)

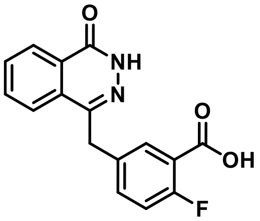

Synthesized according to a literature procedure.[25] All spectroscopic data were in accordance with the literature.[25]

4-(3-(4-(4-Bromobenzoyl)piperazine-1-carbonyl)-4-fluorobenzyl)phthalazin-1(2*H*)-one (s22)

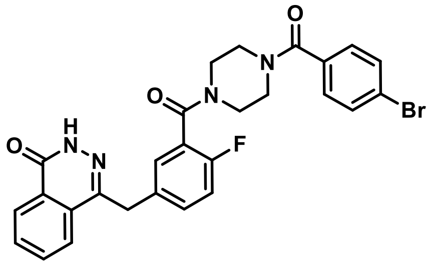

The following is a literature procedure.[25] To a stirring solution of 2-fluoro-5-((4-oxo-3,4-dihydrophthalazin-1-yl)methyl)benzoic acid **s21** (233 mg, 0.781 mmol, 1.0 equiv.) in DMF (17 mL) were added Et_3N (174 µL, 1.25 mmol, 1.6 equiv.) and HBTU (326 mg, 0.86 mmol, 1.1 equiv.). The reaction mixture was stirred under nitrogen at room temperature for 1 h, followed by the addition of (4-bromophenyl)(piperazin-1-yl)methanone **s20** (210 mg, 0.780 mmol, 1.0 equiv.). The reaction mixture was stirred whilst heating to 50 °C for 85 h. Upon completion, H_2O (17 mL) was added and the mixture was stirred at 50 °C for 2 h. The flask was cooled to 0°C, a further portion of H_2O (25 mL) was added and the resulting precipitate was collected by vacuum filtration, whilst washing with H_2O (4 x 30 mL). The crude product was purified by silica gel column chromatography (1 to 6% MeOH in CH_2Cl_2) affording the desired product as a yellow solid (300 mg, 0.546 mmol, 70%). **^{1}H NMR** (600 MHz, $CDCl_3$) δ 11.01 (s, 1H), 8.51 – 8.43 (m, 1H), 7.81 – 7.73 (m, 2H), 7.74 – 7.68 (m, 1H), 7.60 – 7.51 (m, 2H), 7.37 – 7.30 (m, 2H), 7.28 (br d, *J* = 8.0 Hz, 2H), 7.03 (br s, 1H), 4.29 (s, 2H), 4.04 – 3.14 (br m, 8H); **^{19}F NMR** (565 MHz, $CDCl_3$) δ −117.59 – −117.76 (m); **^{13}C NMR** (151 MHz, $CDCl_3$) δ 169.7, 165.3, 160.7, 157.1 (d, *J* = 247.4 Hz), 145.6, 134.6 (d, *J* = 3.3 Hz), 134.0, 133.8, 132.1, 132.0 (d, *J* = 8.1 Hz), 131.8, 129.7, 129.4 (d, *J* = 3.5 Hz), 129.0, 128.5, 127.3 ,125.1, 124.7, 123.7 (d, *J* = 17.9 Hz), 116.3 (d, *J* = 22.1 Hz), 47.1 (br), 42.3 (br), 37.8; **HRMS** (ESI) *m/z* calculated for $C_{27}H_{23}BrFN_4O_3$ ([M+H]$^+$): 549.0932, found 549.0909. All spectroscopic data were in accordance with the literature.[25]

3-Bromo-5-(pyridin-2-ylethynyl)benzonitrile (s23)

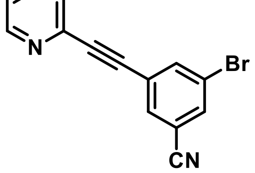

Synthesized according to a literature procedure.[12] All spectroscopic data were in accordance with the literature.[12]

2-Bromo-4-ethynylthiazole (s24)

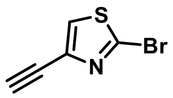

Synthesized according to a literature procedure.[26] All spectroscopic data were in accordance with the literature.[26]

3-((2-Bromothiazol-4-yl)ethynyl)-5-fluorobenzonitrile (s25)

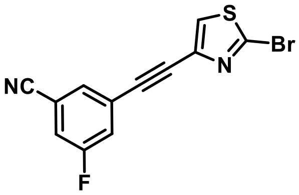

The following is a literature procedure.[27] To a flame dried Schlenk flask equipped with a stir bar were added 3-fluoro-5-iodobenzonitrile (687 mg, 2.78 mmol, 3.0 equiv.), 2-bromo-4-ethynylthiazole **s24** (174 mg, 0.925 mmol, 1.0 equiv.), $Pd(PPh_3)_2Cl_2$ (13 mg, 0.019, 0.020 equiv.) and copper(I) iodide (7.0 mg, 0.0368 mmol, 0.040 equiv.). The flask was evacuated and backfilled with nitrogen three times prior to the addition of anhydrous triethylamine (20 mL). The reaction mixture was stirred at room temperature for 4 h. Upon completion the reaction was quenched by the addition of H_2O (20 mL) and brine (20 mL). The aqueous layer was extracted with CH_2Cl_2 (3 x 40 mL), and the combined organic layers were dried over anhydrous Na_2SO_4, filtered, and concentrated *in vacuo*. The crude product was purified by silica gel column chromatography (0 to 5% Et_2O in pentane). The obtained solid was further purified by recrystallization from CH_2Cl_2/pentane, affording the desired product as a white solid (150 mg, 0.488 mmol, 53%). **^{1}H NMR** (400 MHz, $CDCl_3$) δ 7.62 (t, J = 1.3 Hz, 1H), 7.54 (s, 1H), 7.47 (ddd, J = 8.7, 2.6, 1.3 Hz, 1H), 7.36 (ddd, J = 7.9, 2.6, 1.4 Hz, 1H); **^{19}F NMR** (377 MHz, $CDCl_3$) δ −108.73 (t, J = 8.2 Hz); **^{13}C NMR** (151 MHz, $CDCl_3$) δ 162.1 (d, J = 251.8 Hz), 136.8, 136.5, 131.4, 127.8, 125.9 (d, J = 9.9 Hz), 123.4 (d, J = 22.9 Hz), 119.7 (d, J = 24.7 Hz), 116.9 (d, J = 3.4 Hz), 114.6 (d, J = 10.3 Hz), 86.4 (d, J = 3.5 Hz), 85.5; **HRMS** (ESI) *m/z* calculated for $C_{12}H_5BrFN_2S$ ($[M+H]^+$): 306.9335, found 306.9349. All spectroscopic data were in accordance with the literature.[28]

*Attempted radiolabeling precursor synthesis towards [^{18}F]**2***

Attempts to prepare radiolabeling precursors to [^{18}F]**2** were not successful. This was not pursued further because sulfones [^{18}F]**3** and [^{18}F]**4** were successfully prepared (see S50–S55) which was sufficient to investigate the role of reagent design in the radiochemical setting.

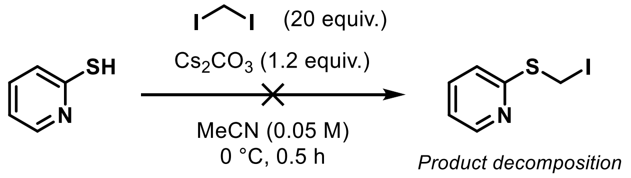

To a flame-dried round bottom flask equipped with a magnetic stir bar was added Cs_2CO_3 (391 mg, 1.20 mmol, 1.2 equiv.). The flask was evacuated and backfilled with nitrogen three times prior to the addition of anhydrous MeCN (8mL) and diiodomethane (1.61 mL, 20.0 mmol, 20 equiv.). The flask was then cooled to 0 °C and pyridine-2-thiol (111 mg, 1.00 mmol, 1.0 equiv.) in MeCN (5 mL) was added dropwise over 10 minutes whilst stirring. The reaction mixture was then stirred under nitrogen at 0 °C for a further 0.5 h. The reaction was quenched by the addition of H_2O (10 mL), and extracted with Et_2O (3 x 20 mL). The combined organic layers were dried over anhydrous Na_2SO_4, filtered, and concentrated *in vacuo*. The crude product was purified by silica gel column chromatography (5 to 10% EtOAc in pentane). Product decomposition was observed upon concentration.

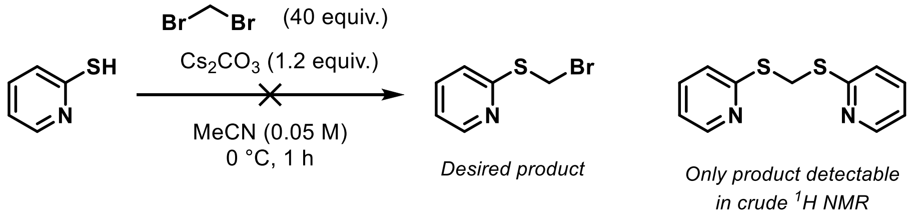

To a flame-dried round bottom flask equipped with a magnetic stir bar was added Cs_2CO_3 (391 mg, 1.20 mmol, 1.2 equiv.). The flask was evacuated and backfilled with nitrogen three times prior to the addition of anhydrous MeCN (15 mL) and dibromomethane (2.80 mL, 40.0 mmol, 40 equiv.). The flask was then cooled to 0 °C and pyridine-2-thiol (111 mg, 1.00 mmol, 1.0 equiv.) in MeCN (5 mL) was added dropwise over 15 minutes whilst stirring. The reaction mixture was then stirred under nitrogen at 0 °C for a further 20 minutes. The reaction was quenched by the addition of H_2O (10 mL), and extracted with Et_2O (3 x 20 mL). The combined organic layers were dried over anhydrous Na_2SO_4, filtered, and concentrated *in vacuo*. The ^{1}H NMR of the crude mixture did not indicate desired product formation.

Synthesis of Reference Compounds

The following aryl halides were purchased from commercial suppliers (ABCR, Acros Organics, Apollo Scientific, BLDpharm, Combi-Blocks, Fluorochem, Key Organics, Sigma Aldrich, TCI, Thermo Fisher Scientific) and used as received, without further purification: 4-(hydroxymethyl)benzonitrile, 1-(bromomethyl)-4-(trifluoromethyl)benzene, 1-(bromomethyl)-4-(methylsulfonyl)benzene, 2-(bromomethyl)-1,1'-biphenyl, naphthalen-1-ylmethanol, (3-(ethoxycarbonyl)phenyl)boronic acid, 3-(hydroxymethyl)phenol, 2-(4-(hydroxymethyl)phenyl)acetate, phenyl(p-tolyl)methanone, 4-(thiophen-2-yl)benzaldehyde, morpholine, 4-(bromomethyl)benzene-1-sulfonyl chloride, benzo[*d*]thiazol-2-ylmethanol, dibenzo[*b,d*]furan-3-ylboronic acid, (6-methoxypyridin-3-yl)methanol, fluoroacetic acid, *N*-hydroxyphthalimide, (1-methyl-1*H*-indazol-6-yl)methanol, 6-methoxybenzo[*d*]thiazol-2-amine, ethyl trifluoroacetate, 4-(4,4,5,5-tetramethyl-1,3,2-dioxaborolan-2-yl)benzaldehyde.

General Procedure 2 (GP2):

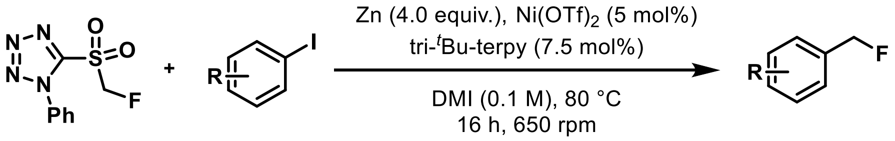

To an oven-dried 7.0 mL glass vial equipped with a magnetic stir bar (see below for details regarding reaction set-up) were added 5-((fluoromethyl)sulfonyl)-1-phenyl-1*H*-tetrazole **3** (1.0 equiv.), aryl iodide (1.5 equiv.), zinc powder (4.0 equiv.), $Ni(OTf)_2$ (5 mol%), tri-^{t}Bu-terpy (7.5 mol%) and DMI (0.1 M). The reaction mixture was stirred whilst heating to 80 °C for 16 h. Upon completion, the reaction was cooled to room temperature, diluted with EtOAc, and washed with a 10% aqueous LiCl solution thrice. The organic layer was then dried over anhydrous Na_2SO_4, filtered and concentrated *in vacuo*. The crude product was purified by silica gel column chromatography.

__Reaction set-up:__

A 7.0 mL screw-neck glass vial (17 mm diameter × 60 mm height) with plastic screw cap lid, and a magnetic stir bar was used (pictured right).

Due to the heterogenous nature of the reaction, efficient stirring was found to be crucial for reproducibility.

For optimal results, the magnetic stir bar should span most of the vial diameter; for example, 15 x 4.5 mm or 15 x 6 mm stir bars were found to be effective in this set-up.

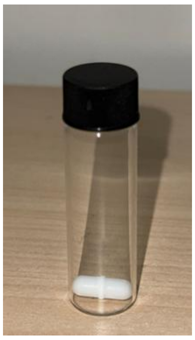

General Procedure 3 (GP3):

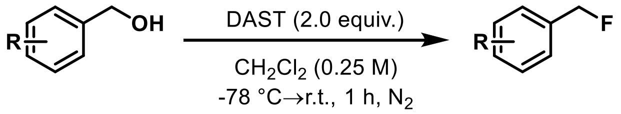

The following is a literature procedure.[29] To a flame-dried round bottom flask equipped with a magnetic stir bar was added alcohol (1.0 equiv.). The flask was evacuated and backfilled with nitrogen three times prior to the addition of anhydrous CH_2Cl_2 (0.25 M). The flask was cooled to −78 °C and diethylaminosulfur trifluoride (DAST, 2.0 equiv.) was added dropwise. The reaction mixture was then stirred at room temperature under nitrogen for 1 hour. Upon completion, the reaction was quenched by the addition of a saturated aqueous solution of $NaHCO_3$, and extracted with Et_2O thrice. The combined organic layers were dried over anhydrous Na_2SO_4, filtered, and concentrated *in vacuo*. The crude product was purified by silica gel column chromatography.

General Procedure 4 (GP4):

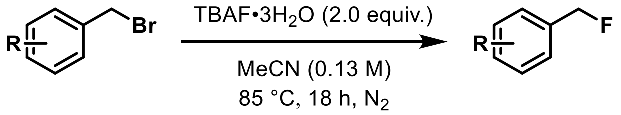

The following is a literature procedure.[30] To a flame-dried round bottom flask equipped with a magnetic stir bar were added benzyl bromide, if solid (1.0 equiv.), and TBAF·$3H_2O$ (2.0 equiv.). The flask was evacuated and backfilled with nitrogen three times prior to the addition of anhydrous MeCN (0.13 M), and benzyl bromide, if liquid (1.0 equiv.). The reaction mixture was then stirred at 85 °C for 18 h under nitrogen. The reaction mixture was cooled to room temperature, concentrated *in vacuo*, and the crude product was purified by silica gel column chromatography.

General Procedure 5 (GP5):

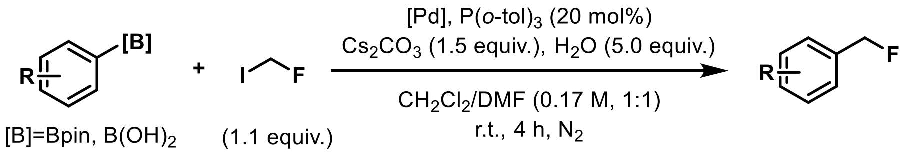

The following is adapted from a literature procedure.[13] To a flame-dried Schlenk flask equipped with a magnetic stir bar were added [Pd] (conditions A: 5 mol% Pd_2dba_3, conditions B: 10 mol% Pd(dppf)Cl_2), P(*o*-tol)$_3$ (20 mol%), Cs_2CO_3 (1.5 equiv.) and aryl boronic acid or pinacol ester (1.0 equiv.) The flask was evacuated and backfilled with nitrogen three times prior to the addition of CH_2Cl_2 (0.3 M), DMF (0.3 M), and H_2O (5.0 equiv.). The reaction mixture was degassed by sparging with nitrogen for 10 min. Fluoroiodomethane (1.1 equiv.) was added and the reaction mixture was stirred at room temperature for 4 h. The reaction mixture was then filtered through a pad of celite and washed with Et_2O. The filtrate was washed with a 10% aqueous LiCl solution twice, dried over anhydrous Na_2SO_4, filtered, and concentrated *in vacuo*. The crude product was purified by silica gel column chromatography.

5-((Fluoromethyl)thio)-1-phenyl-1*H*-tetrazole (s26)

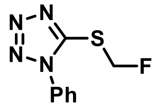

Synthesized according to **GP1-step 1**, using 1-phenyl-1*H*-tetrazole-5-thiol (178 mg, 1.00 mmol, 1.0 equiv.), affording the desired product as a white solid (197 mg, 0.937 mmol, 94%). **^{1}H NMR** (600 MHz, $CDCl_3$) δ 7.68 – 7.48 (m, 5H), 6.21 (d, *J* = 50.0 Hz, 2H); **^{19}F NMR** (377 MHz, $CDCl_3$) δ −188.36 (t, *J* = 50.0 Hz); **^{13}C NMR** (151 MHz, $CDCl_3$) δ 151.5 (d, *J* = 3.0 Hz), 133.3, 130.8, 130.1, 124.3, 84.0 (d, *J* = 226.9 Hz); **HRMS** (ESI) *m/z* calculated for $C_8H_8FN_4S$ ($[M+H]^+$): 211.0448, found 211.0444. All spectroscopic data were in accordance with the literature.[31]

4-(Fluoromethyl)-1,1'-biphenyl (7)

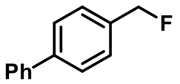

Synthesized according to **GP2** using 4-iodo-1,1'-biphenyl (126 mg, 0.450 mmol). Purification by silica gel column chromatography (100% pentane) afforded the desired product as a white solid (30 mg, 0.159 mmol, 53%). **^{1}H NMR** (400 MHz, $CDCl_3$) δ 7.66 – 7.57 (m, 4H), 7.50 – 7.42 (m, 4H), 7.40 – 7.33 (m, 1H), 5.43 (d, *J* = 47.9 Hz, 2H); **^{19}F NMR** (377 MHz, $CDCl_3$) δ −206.29 (t, *J* = 48.1 Hz); **^{13}C NMR** (101 MHz, $CDCl_3$) δ 141.9 (d, *J* = 3.2 Hz), 140.7 (d, *J* = 1.3 Hz), 135.3 (d, *J* = 17.1 Hz), 129.0, 128.2 (d, *J* = 5.7 Hz), 127.7, 127.5 (d, *J* = 1.5 Hz), 127.3, 84.5 (d, *J* = 166.1 Hz); **HRMS** (ESI) *m/z* calculated for $C_{13}H_{12}F$ ($[M+H]^+$): 187.0918, found 187.0941. All spectroscopic data were in accordance with the literature.[32]

4-(Fluoromethyl-*d*$_2$)-1,1'-biphenyl ([D$_2$]7)

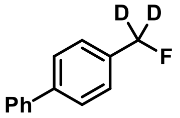

Synthesized according to a literature procedure.[33] All spectroscopic data were in accordance with the literature.[33]

Ethyl 4-(fluoromethyl)benzoate (8)

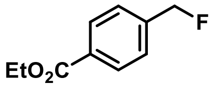

Synthesized according to **GP2**, using ethyl 4-iodobenzoate (124 mg, 0.450 mmol). Purification by silica gel column chromatography (4% EtOAc in pentane) afforded the desired product as a colorless oil (28 mg, 0.154 mmol, 51%). **^{1}H NMR** (400 MHz, $CDCl_3$) δ 8.07 (d, *J* = 7.9 Hz, 2H), 7.43 (d, *J* = 7.8 Hz, 2H), 5.45 (d, *J* = 47.2 Hz, 2H), 4.39 (q, *J* = 7.1 Hz, 2H), 1.40 (t, *J* = 7.1 Hz, 3H); **^{19}F NMR** (377 MHz, $CDCl_3$) δ −212.65 (t, *J* = 47.2 Hz); **^{13}C NMR** (101 MHz, $CDCl_3$) δ 166.4, 141.2 (d, *J* = 17.4 Hz), 130.8 (d, *J* = 2.2 Hz), 130.0, 126.7 (d, *J* = 6.6 Hz), 83.9 (d, *J* = 168.5 Hz), 61.2, 14.5; **HRMS** (ESI) *m/z* calculated for $C_{10}H_{12}FO_2$ ($[M+H]^+$): 183.0816, found 183.0810. All spectroscopic data were in accordance with the literature.[34]

4-(Fluoromethyl)benzonitrile (9)

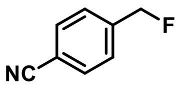

Synthesized according to **GP3**. All spectroscopic data were in accordance with the literature.[29]

1-(4-(Fluoromethyl)phenyl)ethan-1-one (10)

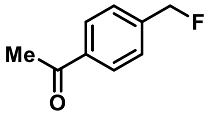

Synthesized according to **GP2** using 1-(4-iodophenyl)ethan-1-one (111 mg, 0.450 mmol). Purification by silica gel chromatography (1 to 6% EtOAc in pentane) afforded the desired product as a yellow oil (28 mg, 0.185 mmol, 62%). **^{1}H NMR** (400 MHz, $CDCl_3$) *δ* 7.98 (dd, *J* = 8.5, 1.2 Hz, 2H), 7.46 (ddd, *J* = 8.0, 1.4, 0.7 Hz, 2H), 5.45 (d, *J* = 47.2 Hz, 2H), 2.62 (s, 3H); **^{19}F NMR** (376 MHz, $CDCl_3$) *δ* −213.13 (t, *J* = 47.0 Hz); **^{13}C NMR** (101 MHz, $CDCl_3$) *δ* 197.6, 141.4 (d, *J* = 17.4 Hz), 137.2 (d, *J* = 2.7 Hz), 128.6, 126.8 (d, *J* = 6.6 Hz), 83.7 (d, *J* = 168.6 Hz), 26.7; **HRMS** (ESI) *m/z* calculated for $C_9H_{10}FO$ ($[M+H]^+$): 153.0710, found 153.0709. All spectroscopic data were in accordance with the literature.[34]

4-(Fluoromethyl)-*N,N*-dimethylbenzamide (11)

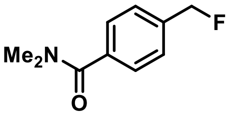

Synthesized according to **GP2** using 4-iodo-*N*,*N*-dimethylbenzamide (124 mg, 0.450 mmol). Purification by silica gel column chromatography (0 to 50% EtOAc in pentane) afforded the desired product as an orange oil (23 mg, 0.124 mmol, 41%). **^{1}H NMR** (400 MHz, $CDCl_3$) *δ* 7.45 (d, *J* = 7.9 Hz, 2H), 7.41 (d, *J* = 7.8 Hz, 2H), 5.40 (d, *J* = 47.6 Hz, 2H), 3.12 (s, 3H), 2.98 (s, 3H); **^{19}F NMR** (377 MHz, $CDCl_3$) *δ* −209.46 (t, *J* = 47.6 Hz); **^{13}C NMR** (151 MHz, $CDCl_3$) *δ* 171.3, 137.7 (d, *J* = 17.1 Hz), 136.9 (d, *J* = 2.9 Hz), 127.5, 127.3 (d, *J* = 6.4 Hz), 84.2 (d, *J* = 167.2 Hz), 39.7, 35.5; **HRMS** (ESI) *m/z* calculated for $C_{10}H_{13}FNO$ ($[M+H]^+$): 182.0976, found 182.0974. All spectroscopic data were in accordance with the literature.[13]

1-(Fluoromethyl)-4-(trifluoromethyl)benzene (12)

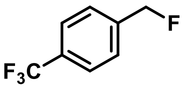

Synthesized according to a literature proceudre.[29] All spectroscopic data were in accordance with the literature.[29]

A second reaction was performed according to **GP2**, using 1-iodo-4-(trifluoromethyl)benzene (66 µL, 0.450 mmol). 4-Fluoroanisole (internal standard, 30 µL, 0.264 mmol) was added and the reaction mixture was diluted with $CDCl_3$, filtered through cotton wool, and analyzed by quantitative ^{19}F NMR, giving an NMR yield of 43%.

1-(Fluoromethyl)-4-(methylsulfonyl)benzene (13)

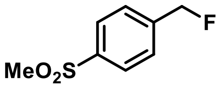

Synthesized according to a literature procedure.[35] All spectroscopic data were in accordance with the literature.[35]

2-(Fluoromethyl)-1,1'-biphenyl (14)

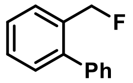

Synthesized according to **GP4**. All spectroscopic data were in accordance with the literature.[30]

1-(Fluoromethyl)naphthalene (15)

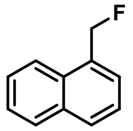

Synthesized according to **GP3** using naphthalen-1-ylmethanol (158 mg, 1.00 mmol). Purification by silica gel chromatography (100% pentane) afforded the desired product as a white solid (55 mg, 0.343 mmol, 34%). **^{1}H NMR** (400 MHz, $CDCl_3$) δ 8.08 (d, J = 8.1 Hz, 1H), 7.91 – 7.88 (m, 2H), 7.61 – 7.45 (m, 4H), 5.86 (d, J = 48.0 Hz, 2H); **^{19}F NMR** (377 MHz, $CDCl_3$) δ −206.23 (t, J = 48.0 Hz); **^{13}C NMR** (126 MHz, $CDCl_3$) δ 133.7, 131.8 (d, J = 15.3 Hz), 131.4 (d, J = 1.9 Hz), 129.9 (d, J = 3.4 Hz), 128.7, 126.9 (d, J = 8.5 Hz), 126.7, 126.1, 125.2 (d, J = 1.9 Hz), 123.6, 83.3 (d, J = 165.6 Hz); **HRMS** (ESI) m/z calculated for $C_{11}H_9F$ ($[M]^+$): 160.0683, found 160.0680. All spectroscopic data were in accordance with the literature.[32]

2-(Fluoromethyl)naphthalene (16)

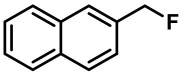

Synthesized according to **GP2** using 2-iodonaphthalene (114 mg, 0.450 mmol). Purification by silica gel chromatography (100% pentane) afforded the desired product as a white solid (23 mg, 0.140 mmol, 47%). **^{1}H NMR** (400 MHz, $CDCl_3$) δ 7.94 – 7.80 (m, 4H), 7.56 – 7.44 (m, 3H), 5.55 (d, J = 47.7 Hz, 2H); **^{19}F NMR** (377 MHz, $CDCl_3$) δ −206.82 (t, J = 48.0 Hz); **^{13}C NMR** (151 MHz, $CDCl_3$) δ 133.8 (d, J = 17.0 Hz), 133.5 (d, J = 1.8 Hz), 133.3, 128.6, 128.3 (d, J = 1.8 Hz), 127.9 (d, J = 1.8 Hz), 126.9 (d, J = 7.3 Hz), 126.6 (d, J = 10.9 Hz), 125.1, 125.1, 84.9 (d, J = 166.5 Hz); **HRMS** (ESI) m/z calculated for $C_{11}H_9F$ ($[M]^+$): 160.0683, found 160.0684. All spectroscopic data were in accordance with the literature.[36]

Ethyl 3-(fluoromethyl)benzoate (17)

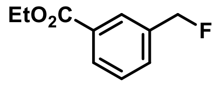

Synthesized according to **GP5** (conditions A), using (3-(ethoxycarbonyl)phenyl)boronic acid (194 mg, 1.00 mmol). Purification by silica gel column chromatography (0 to 2% EtOAc in pentane) afforded the desired product as a light-yellow oil (105 mg, 0.576 mmol, 58%). **^{1}H NMR** (400 MHz, $CDCl_3$) δ 8.05 – 8.03 (m, 2H), 7.59 – 7.56 (m, 1H), 7.48 (t, J = 7.8 Hz, 1H), 5.43 (d, J = 47.5 Hz, 2H), 4.39 (q, J = 7.2 Hz, 2H), 1.40 (t, J = 7.1 Hz, 3H); **^{19}F NMR** (377 MHz, $CDCl_3$) δ −208.87 (t, J = 47.6 Hz); **^{13}C NMR** (101 MHz, $CDCl_3$) δ 166.3, 136.7 (d, J = 17.5 Hz), 131.8 (d, J = 5.8 Hz), 131.1, 130.0 (d, J = 2.3 Hz), 128.9, 128.5 (d, J = 5.9 Hz), 84.1 (d, J = 167.1 Hz), 61.3, 14.5; **HRMS** (ESI) *m/z* calculated for $C_{10}H_{12}FO_2$ ([M+H]$^+$): 183.0816, found 183.0807. All spectroscopic data were in accordance with the literature.[34]

A second reaction was performed according to **GP2**, using ethyl 3-iodobenzoate (76 µL, 0.450 mmol). 4-Fluoroanisole (internal standard, 30 µL, 0.264 mmol) was added and the reaction mixture was diluted with $CDCl_3$, filtered through cotton wool, and analyzed by quantitative ^{19}F NMR, giving an NMR yield of 51%.

(3-Isopropoxyphenyl)methanol (s27)

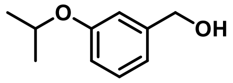

Synthesized according to a literature procedure.[37] All spectroscopic data were in accordance with the literature.[37]

1-(Fluoromethyl)-3-isopropoxybenzene (18)

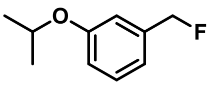

Synthesized according to **GP3** using (3-isopropoxyphenyl)methanol **s27** (100 mg, 0.602 mmol). Purification by silica gel column chromatography (0 to 1% Et_2O in pentane) afforded the desired product as an orange liquid (30 mg, 0.178 mmol, 30%). **^{1}H NMR** (500 MHz, $CDCl_3$) δ 7.30 – 7.27 (m, 1H), 6.93 – 6.91 (m, 2H), 6.89 – 6.87 (m, 1H), 5.34 (d, J = 47.7 Hz, 2H), 4.57 (hept, J = 6.1 Hz, 1H), 1.34 (d, J = 6.0 Hz, 6H); **^{19}F NMR** (471 MHz, $CDCl_3$) δ −207.66 (t, J = 47.7 Hz); **^{13}C NMR** (126 MHz, $CDCl_3$) δ 158.3, 137.9 (d, J = 17.2 Hz), 129.8, 119.5 (d, J = 5.7 Hz), 116.3 (d, J = 2.6 Hz), 114.9 (d, J = 6.1 Hz), 84.7 (d, J = 166.9 Hz), 70.1, 22.2; **HRMS** (GC-EI) *m/z* calculated for $C_{10}H_{13}FO$ ([M]$^+$): 168.0945, found 168.0949; **IR** (neat) 2979, 2360, 1489, 1375, 1266, 1116, 968, 785, 694.

A second reaction was performed according to **GP2**, using 1-iodo-3-isopropoxybenzene (118 mg, 0.450 mmol). 4-Fluoroanisole (internal standard, 30 µL, 0.264 mmol) was added and the reaction mixture was diluted with $CDCl_3$, filtered through cotton wool, and analyzed by quantitative ^{19}F NMR, giving an NMR yield of 48%.

Methyl 2-(4-(fluoromethyl)phenyl)acetate (s28)

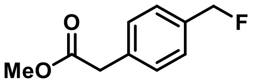

Synthesized according to **GP3** using methyl 2-(4-(hydroxymethyl)phenyl)acetate (496 mg, 2.75 mmol). Purification by silica gel column chromatography (10% Et_2O in pentane) afforded the desired product as a colorless oil (319 mg, 1.75 mmol, 64%). **^{1}H NMR** (400 MHz, $CDCl_3$) δ 7.38 – 7.28 (m, 4H), 5.36 (d, J = 47.7 Hz, 2H), 3.70 (s, 3H), 3.65 (s, 2H); **^{19}F NMR** (377 MHz, $CDCl_3$) δ −206.63 (t, J = 47.8 Hz); **^{13}C NMR** (101 MHz, $CDCl_3$) δ 171.9, 135.2 (d, J = 17.3 Hz), 134.7 (d, J = 3.2 Hz), 129.7, 127.9 (d, J = 5.6 Hz), 84.4 (d, J = 166.1 Hz), 52.2, 41.0; **HRMS** (GC-EI) *m/z* calculated for $C_{10}H_{11}FO_2$ ($[M]^+$): 182.0738, found 182.0742. All spectroscopic data were in accordance with the literature.[38]

2-(4-(Fluoromethyl)phenyl)ethan-1-ol (s29)

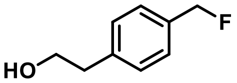

Synthesized exactly as reported in the literature using methyl 2-(4-(fluoromethyl)phenyl)acetate **s28**.[39] All spectroscopic data were in accordance with the literature.[39]

4-(Fluoromethyl)phenethyl 4-methylbenzenesulfonate (19)

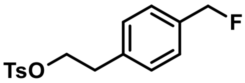

To a flame-dried round bottom flask equipped with a magnetic stir bar was added 2-(4-(fluoromethyl)phenyl)ethan-1-ol **s29** (111 mg, 0.720 mmol, 1.0 equiv.) in dry CH_2Cl_2 (4.5 mL) under nitrogen. Triethylamine (200 µL, 1.44 mmol, 2.0 equiv.), tosyl chloride (164 mg, 0.860 mmol, 1.2 equiv.) and DMAP (4.4 mg, 0.0360 mmol, 0.050 equiv.) were added, and the reaction mixture was stirred under nitrogen at room temperature for 9 h. The reaction was diluted with H_2O (15 mL), and the aqueous layer was extracted with CH_2Cl_2 (3 x 10 mL). The combined organic layers were dried over anhydrous Na_2SO_4, filtered, and concentrated under a flow of nitrogen gas. Purification by silica gel column chromatography (10% Et_2O in pentane) afforded the desired product as a white solid (319 mg, 1.75 mmol, 64%). **^{1}H NMR** (500 MHz, $CDCl_3$) δ 7.69 (d, J = 8.1 Hz, 2H), 7.32 – 7.25 (m, 4H), 7.16 (d, J = 7.6 Hz, 2H), 5.36 (d, J = 47.9 Hz, 2H), 4.24 (t, J = 6.9 Hz, 2H), 2.99 (t, J = 6.8 Hz, 2H), 2.45 (s, 3H); **^{19}F NMR** (471 MHz, $CDCl_3$) δ −205.82 (t, J = 47.9 Hz); **^{13}C NMR** (126 MHz, $CDCl_3$) δ 144.8, 137.1 (d, J = 2.9 Hz), 135.0 (d, J = 17.2 Hz), 133.0, 129.9, 129.3, 128.1 (d, J = 5.6 Hz), 127.9, 84.4 (d, J = 165.6 Hz), 70.5, 35.2, 21.7; **HRMS** (ESI) *m/z* calculated for $C_{16}H_{17}FO_3SNa$ ($[M+Na]^+$): 331.0775, found 331.0761; **IR** (neat) 1381, 1188, 1013, 946, 705; **m.p.** 53–55 °C.

(4-(Fluoromethyl)phenyl)(phenyl)methanone (20)

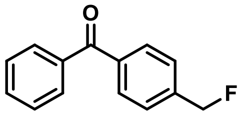

Synthesized according to a literature procedure.[40] All spectroscopic data were in accordance with the literature.[40]

(4-(Thiophen-2-yl)phenyl)methanol (s30)

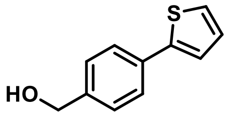

To a flame-dried round bottom flask equipped with a magnetic stir bar was added 4-(thiophen-2-yl)benzaldehyde (188 mg, 1.00 mmol, 1.0 equiv.). The flask was evacuated and backfilled with nitrogen three times prior to the addition of anhydrous CH_2Cl_2 (5 mL) and MeOH (5 mL). The flask was cooled to 0 °C and $NaBH_4$ (45 mg, 1.19 mmol, 1.2 equiv.) was added. The reaction mixture was then stirred at room temperature under nitrogen for 1.5 h. The reaction was quenched by the addition of aqueous HCl (1 M, 10 mL) and extracted with CH_2Cl_2 (3 x 10 mL). The combined organic layers were dried over anhydrous Na_2SO_4, filtered, and concentrated *in vacuo*. Purification by silica gel column chromatography (20% EtOAc in pentane) afforded the desired product as a white solid (133 mg, 0.699 mmol, 70%). **^{1}H NMR** (600 MHz, $CDCl_3$) δ 7.65 – 7.58 (m, 2H), 7.42 – 7.36 (m, 2H), 7.32 (dd, *J* = 3.6, 1.2 Hz, 1H), 7.28 (dd, *J* = 5.1, 1.1 Hz, 1H), 7.08 (dd, *J* = 5.1, 3.6 Hz, 1H), 4.71 (s, 2H); **^{13}C NMR** (151 MHz, $CDCl_3$) δ 144.2, 140.2, 134.0, 128.2, 127.7, 126.3, 125.0, 123.3, 65.2; **HRMS** (ESI) *m/z* calculated for $C_{11}H_{10}OSNa$ ($[M+Na]^+$): 213.0345, found 213.0343. All spectroscopic data were in accordance with the literature.[41]

2-(4-(Bromomethyl)phenyl)thiophene (s31)

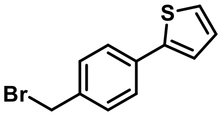

Synthesized according to a literature procedure from (4-(thiophen-2-yl)phenyl)methanol **s30**.[41] All spectroscopic data were in accordance with the literature.[41]

2-(4-(Fluoromethyl)phenyl)thiophene (21)

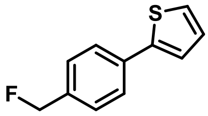

Synthesized according to **GP4** using 2-(4-(bromomethyl)phenyl)thiophene **s31** (52 mg, 0.205 mmol). Purification by silica gel column chromatography (10% CH_2Cl_2 in pentane) afforded the desired product as a white solid (38 mg, 0.198 mmol, 97%). **^{1}H NMR** (500 MHz, $CDCl_3$) δ 7.65 (dd, *J* = 8.3, 1.3 Hz, 2H), 7.40 (dd, *J* = 8.4, 2.0 Hz, 2H), 7.34 (dd, *J* = 3.6, 1.1 Hz, 1H), 7.30 (dd, *J* = 5.1, 1.2 Hz, 1H), 7.10 (dd, *J* = 5.1, 3.6 Hz, 1H), 5.39 (d, *J* = 47.9 Hz, 2H); **^{19}F NMR** (470 MHz, $CDCl_3$) δ −206.44 (t, *J* = 47.7 Hz); **^{13}C NMR** (126 MHz, $CDCl_3$) δ 143.9 (d, *J* = 2.1 Hz), 135.4 (d, *J* = 16.9 Hz), 135.0 (d, *J* = 3.3 Hz), 128.3 (d, *J* = 5.9 Hz), 128.2, 126.2 (d, *J* = 1.8 Hz), 125.3, 123.6, 84.4 (d, *J* = 166.2 Hz); **HRMS** (GC-EI) *m/z* calculated for $C_{11}H_9FS$ ($[M]^+$): 192.0404, found 192.0415. All spectroscopic data were in accordance with the literature.[42]

4-((4-(Bromomethyl)phenyl)sulfonyl)morpholine (s32)

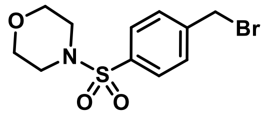

Synthesized according to a literature procedure.[43] All spectroscopic data were in accordance with the literature.[43]

4-((4-(Fluoromethyl)phenyl)sulfonyl)morpholine (22)

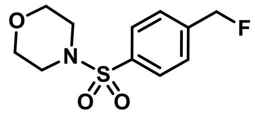

Synthesized according to **GP4** using 4-((4-(bromomethyl)phenyl)sulfonyl)morpholine **s32** (241 mg, 0.753 mmol). Purification by silica gel column chromatography (15 to 20% EtOAc in pentane) afforded the desired product as a white solid (177 mg, 0.683 mmol, 91%). **^{1}H NMR** (500 MHz, $CDCl_3$) δ 7.79 – 7.77 (m, 2H), 7.55 – 7.53 (m, 2H), 5.48 (d, J = 47.0 Hz, 2H), 3.74 – 3.72 (m, 4H), 3.01 – 2.99 (m, 4H); **^{19}F NMR** (470 MHz, $CDCl_3$) δ −214.54 (t, J = 47.2 Hz); **^{13}C NMR** (126 MHz, $CDCl_3$) δ 141.8 (d, J = 17.7 Hz), 135.3 (d, J = 2.4 Hz), 128.3, 127.2 (d, J = 6.7 Hz), 83.3 (d, J = 169.9 Hz), 66.2, 46.1; **HRMS** (ESI) *m/z* calculated for $C_{11}H_{15}FNO_3S$ ([M+H]$^+$): 260.0751, found 260.0746; **IR** (neat) 2918, 2851, 2361, 1346, 1332, 1265, 1164, 1114, 940, 813, 738; **m.p.** 124–127 °C.

Methyl (*E*)-3-(4-(4,4,5,5-tetramethyl-1,3,2-dioxaborolan-2-yl)phenyl)acrylate (s33)

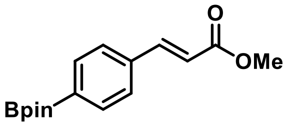

Synthesized according to a literature procedure.[44] All spectroscopic data were in accordance with the literature.[44]

Methyl (*E*)-3-(4-(fluoromethyl)phenyl)acrylate (23)

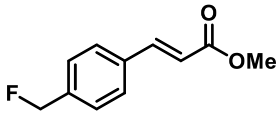

Synthesized according to adapted **GP5** (conditions A), using methyl (*E*)-3-(4-(4,4,5,5-tetramethyl-1,3,2-dioxaborolan-2-yl)phenyl)acrylate **s33** (144 mg, 0.500 mmol). Purification by silica gel column chromatography (1% acetone in pentane) afforded the desired product as a pale-yellow solid (62 mg, 0.319 mmol, 64%). **^{1}H NMR** (600 MHz, $CDCl_3$) δ 7.71 (d, J = 16.0 Hz, 1H), 7.56 (dd, J = 8.1, 1.4 Hz, 2H), 7.42 – 7.40 (m, 2H), 6.48 (dd, J = 16.0, 0.7 Hz, 1H), 5.41 (d, J = 47.5 Hz, 2H), 3.83 (s, 3H); **^{19}F NMR** (565 MHz, $CDCl_3$) δ −209.72 (t, J = 47.5 Hz); **^{13}C NMR** (151 MHz, $CDCl_3$) δ 167.4, 144.2 (d, J = 1.2 Hz), 138.5 (d, J = 17.0 Hz), 134.8 (d, J = 2.9 Hz), 128.4, 127.8 (d, J = 6.1 Hz), 118.5, 84.0 (d, J = 167.6 Hz), 51.9; **HRMS** (GC-EI) *m/z* calculated for $C_{11}H_{11}FO_2$ ([M]$^+$): 194.0738 found 194.0751; **IR** (neat) 2361, 1710, 1637, 1321, 1174, 971, 835, 791; **m.p.** 57–59 °C.

6-(Fluoromethyl)-2-methylbenzo[*d*]thiazole (24)

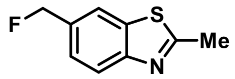

Synthesized according to **GP2** using 5-iodo-2-methylbenzo[*d*]thiazole (83 mg, 0.300 mmol). Purification by silica gel column chromatography (5 to 10% acetone in hexanes) afforded the desired product as a white solid (20 mg, 0.109 mmol, 55%). **^{1}H NMR** (500 MHz, $CDCl_3$) δ 7.96 (d, J = 8.3 Hz, 1H), 7.86 (t, J = 2.0 Hz, 1H), 7.45 (dt, J = 8.4, 1.7 Hz, 1H), 5.49 (d, J = 47.9 Hz, 2H), 2.85 (s, 3H); **^{19}F NMR** (471 MHz, $CDCl_3$) δ −204.64 (t, J = 47.8 Hz); **^{13}C NMR** (126 MHz, $CDCl_3$) δ 168.3, 153.6 (d, J = 2.7 Hz), 136.0, 133.0 (d, J = 17.2 Hz), 125.8 (d, J = 5.1 Hz), 122.6, 120.8 (d, J = 6.8 Hz), 84.6 (d, J = 167.2 Hz), 20.3; **HRMS** (ESI) *m/z* calculated for C_9H_9FNS ([M+H]$^+$): 182.0434, found 182.0432; **IR** (neat) 1523, 1458, 1418, 1253, 1164, 963, 881, 818, 644; **m.p.** 40−41 °C.

2-(Fluoromethyl)benzo[*d*]thiazole (25)

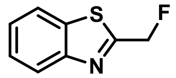

Synthesized according to **GP3** using benzo[*d*]thiazol-2-ylmethanol (99 mg, 0.599 mmol). Purification by silica gel column chromatography (0 to 2% EtOAc in pentane) afforded the desired product as a yellow solid (65 mg, 0.389 mmol, 65%). **^{1}H NMR** (500 MHz, $CDCl_3$) δ 8.05 (d, J = 8.2 Hz, 1H), 7.94 (dd, J = 8.0, 1.2 Hz, 1H), 7.52 (ddd, J = 8.3, 7.1, 1.3 Hz, 1H), 7.44 (td, J = 7.7, 1.2 Hz, 1H), 5.76 (d, J = 46.7 Hz, 2H); **^{19}F NMR** (471 MHz, $CDCl_3$) δ −213.78 (t, J = 46.8 Hz); **^{13}C NMR** (126 MHz, $CDCl_3$) δ 166.0 (d, J = 24.5 Hz), 152.8, 135.0, 126.5, 125.7, 123.4, 121.9, 81.4 (d, J = 170.7 Hz); **HRMS** (ESI) *m/z* calculated for C_8H_7FSN ([M+H]$^+$): 168.0278, found 168.0275. All spectroscopic data were in accordance with the literature.[45]

3-(Fluoromethyl)dibenzo[*b,d*]furan (26)

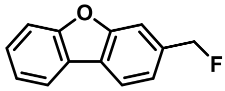

Synthesized according to adapted **GP5** (conditions A), using dibenzo[*b,d*]furan-3-ylboronic acid (212 mg, 1.00 mmol). Purification by silica gel column chromatography (100% pentane) afforded the desired product as a white solid (45 mg, 0.225 mmol, 23%). **^{1}H NMR** (600 MHz, $CDCl_3$) δ 7.97 (d, J = 7.8 Hz, 2H), 7.66 – 7.56 (m, 2H), 7.48 (ddd, J = 8.3, 7.2, 1.3 Hz, 1H), 7.42 – 7.33 (m, 2H), 5.54 (d, J = 47.8 Hz, 2H); **^{19}F NMR** (377 MHz, $CDCl_3$) δ −204.91 (t, J = 47.9 Hz); **^{13}C NMR** (151 MHz, $CDCl_3$) δ 156.8, 156.3, 135.6 (d, J = 17.2 Hz), 127.6, 124.8 (d, J = 2.3 Hz), 123.9, 123.0, 122.3 (d, J = 5.8 Hz), 120.9, 120.9, 111.9, 111.0 (d, J = 6.5 Hz), 84.8 (d, J = 167.3 Hz); **HRMS** (GC-EI) *m/z* calculated for $C_{13}H_9FO$ ([M]$^+$): 200.0632, found 200.0639. All spectroscopic data were in accordance with the literature.[42]

6-(Fluoromethyl)quinoline (27)

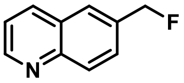

Synthesized according to **GP2** using 6-iodoquinoline (115 mg, 0.450 mmol). Purification by silica gel column chromatography (15 to 20% EtOAc in pentane) afforded the desired product as a green oil (22 mg, 0.136 mmol, 45%). **^{1}H NMR** (400 MHz, $CDCl_3$) δ 8.95 (dd, J = 4.3, 1.7 Hz, 1H), 8.18 (d, J = 8.2 Hz, 1H), 8.14 (d, J = 8.7 Hz, 1H), 7.82 (m, 1H), 7.74 – 7.65 (m, 1H), 7.44 (dd, J = 8.3, 4.2 Hz, 1H), 5.57 (d, J = 47.3 Hz, 2H); **^{19}F NMR** (377 MHz, $CDCl_3$) δ −209.16 (t, J = 47.2 Hz); **^{13}C NMR** (101 MHz, $CDCl_3$) δ 150.9, 148.1, 136.2, 134.6 (d, J = 17.2 Hz), 130.0, 128.3 (d, J = 4.8 Hz), 128.0, 126.2 (d, J = 7.9 Hz), 121.6, 84.1 (d, J = 168.0 Hz); **HRMS** (ESI) *m/z* calculated for $C_{10}H_8FN$ ($[M]^+$): 161.0635, found 161.0634. All spectroscopic data were in accordance with the literature.[46]

5-(Fluoromethyl)-2-methoxypyridine (28)

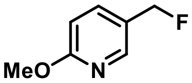

Synthesized according to **GP3** using (6-methoxypyridin-3-yl)methanol (150 µL, 1.25 mmol). Purification by silica gel column chromatography (10% EtOAc in pentane) afforded the desired product as a yellow liquid (76 mg, 0.538 mmol, 43%). **^{1}H NMR** (600 MHz, $CDCl_3$) δ 8.18 (t, J = 2.9 Hz, 1H), 7.63 (ddd, J = 8.5, 2.3, 1.5 Hz, 1H), 6.78 (d, J = 8.5 Hz, 1H), 5.30 (d, J = 48.5 Hz, 2H), 3.96 (s, 3H); **^{19}F NMR** (565 MHz, $CDCl_3$) δ −201.74 (t, J = 48.5 Hz); **^{13}C NMR** (151 MHz, $CDCl_3$) δ 164.8 (d, J = 2.6 Hz), 147.1 (d, J = 6.3 Hz), 139.3 (d, J = 3.4 Hz), 124.6 (d, J = 18.1 Hz), 111.3, 82.3 (d, J = 165.5 Hz), 53.8; **HRMS** (ESI) *m/z* calculated for C_7H_9FNO ($[M+H]^+$): 142.0663, found 142.0658; **IR** (thin layer film) 2917, 2361, 1613, 1499, 1292, 1018, 917, 832, 762, 736, 693.

A second reaction was performed according to **GP2**, using 5-iodo-2-methoxypyridine (57 µL, 0.450 mmol). 4-Fluoroanisole (internal standard, 30 µL, 0.264 mmol) was added and the reaction mixture was diluted with $CDCl_3$, filtered through cotton wool, and analyzed by quantitative ^{19}F NMR, giving an NMR yield of 47%.

1,3-Dioxoisoindolin-2-yl 2-fluoroacetate (s34)

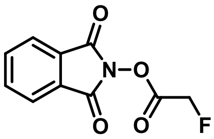

Caution: fluoroacetic acid is highly toxic if in contact with skin or eyes and fatal if swallowed, and must be handled with great caution, full COSHH assessment required prior to use.

To a flame-dried round bottom flask equipped with a magnetic stir bar were added fluoroacetic acid (300 µL, 5.27 mmol, 1.0 equiv.) and anhydrous CH_2Cl_2 (25 mL). The flask was cooled to 0 °C and SO_2Cl_2 (1.92 mL, 26.3 mmol, 5.0 equiv.) was added dropwise under nitrogen. The reaction mixture was then heated to 30 °C whilst stirring for 30 minutes. The flask was then cooled to 0 °C and a solution of *N*-hydroxyphthalimide (859 mg, 5.27 mmol, 1.0 equiv.) and pyridine (1.28 mL, 15.8 mmol, 3.0 equiv.) in CH_2Cl_2 (25 mL) was added. The reaction mixture was stirred at room temperature under nitrogen for 15 h. Upon completion, the reaction was

concentrated under the flow of nitrogen gas. The crude product was purified by silica gel column chromatography (20% EtOAc in pentane). The resulting solid was redissolved in a minimal volume of CH_2Cl_2. Pentane was then added and the resulting solution was kept at −20 °C until precipitation occurred. The precipitate was then collected by filtration, washed with additional ice-cold pentane and dried, affording the desired product as a white solid (852 mg, 3.82 mmol, 72% yield). **^{1}H NMR** (400 MHz, $CDCl_3$) δ 7.92 (dd, J = 5.5, 3.1 Hz, 2H), 7.83 (dd, J = 5.5, 3.1 Hz, 2H), 5.28 (d, J = 46.2 Hz, 2H); **^{19}F NMR** (377 MHz, $CDCl_3$) δ −234.07 (t, J = 46.2 Hz); **^{13}C NMR** (101 MHz, $CDCl_3$) δ 164.4 (d, J = 23.1 Hz), 161.4, 135.2, 128.8, 124.4, 76.0 (d, J = 186.2 Hz); **HRMS** (ESI) *m/z* calculated for $C_{10}H_6FNO_4Na$ ($[M+Na]^+$): 246.0173, found 246.0165. Spectroscopic data were in accordance with the literature.[47]

***Note**: compound **s34** was concentrated in plastic vessels and stored at −20 °C to prevent degradation. After precipitation, the final product was dried under the flow of nitrogen gas and then in vacuo for 5 minutes to prevent degradation observed upon prolonged periods of drying by rotary evaporation.*

3-(Fluoromethyl)-1-tosyl-1*H*-indole (29)

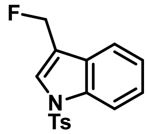

To an oven-dried 4 mL glass vial equipped with a magnetic stir bar were added 3-iodo-1-tosyl-1*H*-indole (60 mg, 0.151 mmol, 1.0 equiv.), 1,3-dioxoisoindolin-2-yl 2-fluoroacetate **s34** (40 mg, 0.179 mmol, 1.2 equiv.), zinc powder (20 mg, 0.306 mmol, 2.0 equiv.), dtbbpy (4.8 mg, 0.0179 mmol, 0.12 equiv.) and $NiBr_2 \cdot DME$ (5.6 mg, 0.0181 mmol, 0.12 equiv.). The vial was sealed, and evacuated and backfilled with nitrogen three times. Anhydrous DMA (190 µL, 0.8 M) was then added, followed by TMSCl (57 µL, 0.449 mmol, 3.0 equiv.). The reaction mixture was then stirred at room temperature for 1 h. Purification of the reaction mixture directly by silica gel column chromatography (10 to 50% EtOAc in pentane) afforded the desired product as a white solid (29 mg, 0.0956 mmol, 63%). **^{1}H NMR** (400 MHz, $CDCl_3$) δ 7.98 (dt, J = 8.3, 0.9 Hz, 1H), 7.78 (d, J = 8.4 Hz, 2H), 7.65 (d, J = 4.7 Hz, 1H), 7.63 – 7.59 (m, 1H), 7.36 (ddd, J = 8.4, 7.3, 1.3 Hz, 1H), 7.31 – 7.27 (m, 1H), 7.24 (d, J = 8.3 Hz, 2H), 5.51 (d, J = 48.3 Hz, 2H), 2.35 (s, 3H); **^{19}F NMR** (377 MHz, $CDCl_3$) δ −207.34 (td, J = 48.3, 4.8 Hz); **^{13}C NMR** (126 MHz, $CDCl_3$) δ 145.4, 135.3 (d, J = 13.7 Hz), 130.2, 129.4, 127.1, 126.0 (d, J = 9.3 Hz), 125.4, 123.7, 119.9, 117.9, 117.7, 113.8, 76.6 (d, J = 163.1 Hz), 21.7; **HRMS** (ESI) *m/z* calculated for $C_{16}H_{14}FNO_2SNa$ ($[M+Na]^+$): 326.0622, found 326.0612. All spectroscopic data were in accordance with the literature.[48]

6-(Fluoromethyl)-1-methyl-1*H*-indazole (30)

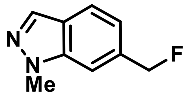

Synthesized according to **GP3** using (1-methyl-1*H*-indazol-6-yl)methanol (243 mg, 1.50 mmol). Purification by silica gel column chromatography (30% EtOAc in pentane) afforded the desired product as a white solid (108 mg, 0.658 mmol, 44%). **^{1}H NMR** (500 MHz, $CDCl_3$) δ 7.98 (d, J = 1.0 Hz, 1H), 7.73 (dt, J = 8.3, 1.0 Hz, 1H), 7.42 (m, 1H), 7.12 (dt, J = 8.4, 1.4 Hz, 1H), 5.52 (d, J = 47.7 Hz, 2H), 4.07 (s, 3H); **^{19}F NMR** (471 MHz, $CDCl_3$) δ −206.48 (t, J

= 47.7 Hz); **^{13}C NMR** (126 MHz, $CDCl_3$) *δ* 139.9, 134.7 (d, *J* = 16.8 Hz), 132.8, 124.1 (d, *J* = 2.1 Hz), 121.5, 119.9 (d, *J* = 5.1 Hz), 107.8 (d, *J* = 8.0 Hz), 84.9 (d, *J* = 167.4 Hz), 35.6; **HRMS** (ESI) *m/z* calculated for $C_9H_9FN_2$ ($[M]^+$): 164.0744, found 164.0743; **IR** (neat) 1376, 1187, 973, 941, 864, 847, 814, 767, 706; **m.p.** 45–47 °C.

***tert*-Butyl 5'-(4,4,5,5-tetramethyl-1,3,2-dioxaborolan-2-yl)-3'*H*-spiro[azetidine-3,1'-isobenzofuran]-1-carboxylate (s35)**

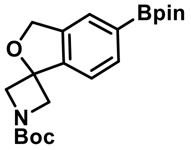

To a flame-dried Schlenk flask equipped with a magnetic stir bar were added *tert*-butyl 5'-bromo-3'*H*-spiro[azetidine-3,1'-isobenzofuran]-1-carboxylate (714 mg, 2.10 mmol, 1.1 equiv.), bis(pinacolato)diboron (508 mg, 2.00 mmol, 1.0 equiv.), KOAc (589 mg, 6.00 mmol, 3.0 equiv.) and Pd(dppf)Cl_2 (44 mg, 0.0601 mmol, 0.030 equiv.). The flask was evacuated and backfilled with nitrogen three times prior to the addition of anhydrous 1,4-dioxane (10 mL, 0.2 M). The reaction mixture was degassed by bubbling with nitrogen for 10 min, and then heated to 80 °C whilst stirring for 14 h. The reaction mixture was then filtered through a pad of celite and washed with CH_2Cl_2. The filtrate was concentrated *in vacuo*, and the crude product was purified by silica gel column chromatography (20 to 30% EtOAc in hexane), affording the desired product as a white solid (669 mg, 1.73 mmol, 87%). **^{1}H NMR** (400 MHz, $CDCl_3$) *δ* 7.83 (dd, *J* = 7.5, 1.0 Hz, 1H), 7.66 (m, 1H), 7.48 (d, *J* = 7.6 Hz, 1H), 5.10 (s, 2H), 4.31 (dd, *J* = 9.3, 1.1 Hz, 2H), 4.13 (dd, *J* = 9.3, 1.1 Hz, 2H), 1.48 (s, 9H), 1.35 (s, 12H); **^{13}C NMR** (126 MHz, $CDCl_3$) *δ* 156.6, 144.8, 138.1, 135.0, 127.2, 120.2, 84.2, 82.7, 80.0, 72.9, 64.2, 28.5, 25.0. *Note:* C(Ar)-B was not observed; **HRMS** (ESI) *m/z* calculated for $C_{21}H_{31}BNO_5$ ($[M+H]^+$): 388.2290, found 388.2279; **IR** (neat) 2360, 1705, 1395, 1364, 1348, 1164, 1142, 1096, 1078, 854; **m.p** 128 – 131 °C.

***tert*-Butyl 5'-(fluoromethyl)-3'*H*-spiro[azetidine-3,1'-isobenzofuran]-1-carboxylate (31)**

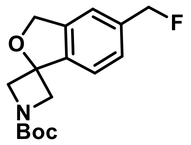

Synthesized according to adapted **GP5** (conditions A), using *tert*-butyl 5' (4,4,5,5 tetramethyl 1,3,2 dioxaborolan-2-yl)-3'*H*-spiro[azetidine-3,1'-isobenzofuran]-1-carboxylate **s35** (387 mg, 1.00 mmol). Purification by silica gel column chromatography (10% acetone in hexanes) afforded the desired product as a colorless oil (117 mg, 0.399 mmol, 40%). **^{1}H NMR** (500 MHz, $CDCl_3$) *δ* 7.49 (d, *J* = 7.7 Hz, 1H), 7.39 – 7.37 (m, 1H), 7.24 – 7.23 (m, 1H), 5.40 (d, *J* = 47.7 Hz, 2H), 5.12 (s, 2H), 4.31 (dd, *J* = 9.2, 1.1 Hz, 2H), 4.13 (dd, *J* = 9.4, 1.1 Hz, 2H), 1.48 (s, 9H); **^{19}F NMR** (470 MHz, $CDCl_3$) *δ* −206.52 (t, *J* = 47.7 Hz); **^{13}C NMR** (126 MHz, $CDCl_3$) *δ* 156.6, 142.4 (d, *J* = 2.9 Hz), 139.4, 136.9 (d, *J* = 16.9 Hz), 127.8 (d, *J* = 5.9 Hz), 121.1, 120.0 (d, *J* = 6.0 Hz), 84.3 (d, *J* = 166.7 Hz), 82.6, 80.0, 72.8, 64.2, 28.5; **HRMS** (ESI) *m/z* calculated for $C_{16}H_{21}FNO_3$ ($[M+H]^+$): 294.1500, found 294.1494; **IR** (neat) 2360, 1701, 1393, 1166, 1095, 1054.

_N_,_N'_,_N''_-tetra-Boc-_N_-(3-(4',4',5',5'-tetramethyl-1,3,2-dioxaborolan-2-yl)benzyl)guanidine (s36)

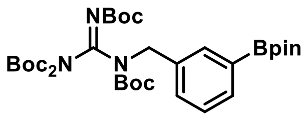

Synthesized according to a literature procedure from **s10**.[49] All spectroscopic data were in accordance with the literature.[49]

_N_,_N'_,_N''_-tetra-Boc-_N_-(3-(fluoromethyl)benzyl)guanidine (32)

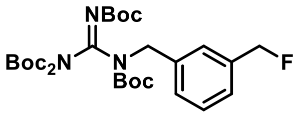

Synthesized according to adapted **GP5** (conditions A), using *N*,*N'*,*N''*-tetra-Boc-*N*-(3-(4',4',5',5'-tetramethyl-1,3,2-dioxaborolan-2-yl)benzyl)guanidine **s36** (203 mg, 0.300 mmol). Purification by silica gel column chromatography (8 to 15% Et_2O in pentane) afforded the desired product as a red oil (79 mg, 0.136 mmol, 45%). **^{1}H NMR** (500 MHz, $CDCl_3$) δ 7.42 – 7.40 (m, 2H), 7.33 – 7.29 (m, 1H), 7.26 – 7.24 (m, 1H), 5.34 (d, *J* = 47.8 Hz, 2H), 5.04 (s, 2H), 1.49 (s, 9H), 1.46 (s, 18H), 1.39 (s, 9H); **^{19}F NMR** (470 MHz, $CDCl_3$) δ -206.93 (t, *J* = 47.8 Hz); **^{13}C NMR** (126 MHz, $CDCl_3$) δ 157.5, 151.3, 147.5, 144.7, 138.2, 136.3 (d, *J* = 17.1 Hz), 128.6 (d, *J* = 1.0 Hz), 128.3 (d, *J* = 2.8 Hz), 127.1 (d, *J* = 5.9 Hz), 126.5 (d, *J* = 5.9 Hz), 84.6 (d, *J* = 166.4 Hz), 84.0, 83.8, 82.7, 82.2, 28.1, 28.0, 28.0; **HRMS** (ESI) *m/z* calculated for $C_{29}H_{44}FN_3O_8Na$ ($[M+Na]^+$): 604.3005, found 604.2980; **IR** (neat) 1728, 1369, 1280, 1255, 1226, 1128, 1104.

2-Amino-5-methoxybenzenethiol (s37)

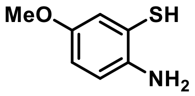

Synthesized according to a literature procedure.[50] All spectroscopic data were in accordance with the literature.[50]

4-(Fluoromethyl)benzaldehyde (s38)

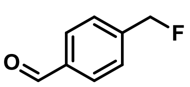

Synthesized according to a literature procedure.[13] All spectroscopic data were in accordance with the literature.[13]

2-(4-(Fluoromethyl)phenyl)-6-methoxybenzo[_d_]thiazole (33)

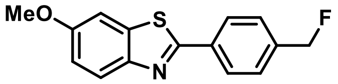

The following is a literature procedure.[51] To an oven-dried microwave vial was added 2-amino-5-methoxybenzenethiol **s37** (68 mg, 0.438 mmol, 1.0 equiv.). The vial was sealed and evacuated and backfilled

with nitrogen three times prior to the addition of a solution of 4-(fluoromethyl)benzaldehyde **s38** (60 mg, 0.434 mmol, 1.0 equiv.) in DMSO (2.6 mL). The reaction mixture was stirred whilst heating to 160 °C for 4 h. The reaction was quenched by the addition of a saturated aqueous NH_4Cl solution (20 mL) and diluted with CH_2Cl_2 (20 mL). The organic layer was washed with a saturated aqueous NH_4Cl solution (2 x 10 mL). The organic layer was dried over anhydrous Na_2SO_4, filtered, and concentrated *in vacuo*. Purification by silica gel column chromatography (7% EtOAc in pentane) afforded the desired product as a white solid (109 mg, 0.399 mmol, 92%). **^{1}H NMR** (500 MHz, $CDCl_3$) δ 8.06 – 8.04 (m, 2H), 7.95 (d, *J* = 8.9 Hz, 1H), 7.46 (dd, *J* = 8.2, 1.8 Hz, 2H), 7.33 (d, *J* = 2.6 Hz, 1H), 7.09 (dd, *J* = 9.0, 2.6 Hz, 1H), 5.43 (d, *J* = 47.6 Hz, 2H), 3.87 (s, 3H); **^{19}F NMR** (377 MHz, $CDCl_3$) δ −210.05 (t, *J* = 47.4 Hz); **^{13}C NMR** (126 MHz, $CDCl_3$) δ 164.9, 158.0, 148.7, 138.7 (d, *J* = 17.1 Hz), 136.6, 134.1 (d, *J* = 2.9 Hz), 127.8 (d, *J* = 6.2 Hz), 127.5, 123.9, 115.9, 104.2, 84.0 (d, *J* = 167.7 Hz), 55.9; **HRMS** (ESI) *m/z* calculated for $C_{15}H_{13}FNOS$ ([M+H]$^+$): 274.0696, found 274.0686; **IR** (neat) 2967, 1602, 1491, 1462, 1435, 1266, 1226, 1059, 966, 946, 836; **m.p.** 142–145 °C.

Methyl (*R*)-2-((*tert*-butoxycarbonyl)amino)-3-(4-(fluoromethyl)phenyl)propanoate (s39)

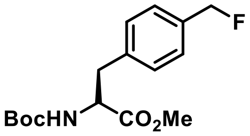

Synthesized according to adapted **GP5** (conditions A), using methyl (*R*)-2-((*tert*-butoxycarbonyl)amino)-3-(4-(4,4,5,5-tetramethyl-1,3,2-dioxaborolan-2-yl)phenyl)propanoate (118 mg, 0.291 mmol). Purification by silica gel column chromatography (0 to 10% acetone in pentane) afforded the desired product as a red oil (85 mg, 0.273 mmol, 94%). **^{1}H NMR** (500 MHz, $CDCl_3$) δ 7.31 (dd, *J* = 8.1, 1.9 Hz, 2H), 7.15 (d, *J* = 7.6 Hz, 2H), 5.34 (d, *J* = 47.9 Hz, 2H), 4.97 (d, *J* = 8.3 Hz, 1H), 4.59 (q, *J* = 6.6 Hz, 1H), 3.71 (s, 3H), 3.16 – 3.04 (m, 2H), 1.41 (s, 9H); **^{19}F NMR** (470 MHz, $CDCl_3$) δ −206.32 (t, *J* = 47.8 Hz); **^{13}C NMR** (126 MHz, $CDCl_3$) δ 172.3, 155.2, 136.9 (d, *J* = 3.2 Hz), 135.1 (d, *J* = 17.0 Hz), 129.7, 128.0 (d, *J* = 5.6 Hz), 84.5 (d, *J* = 166.0 Hz), 80.1, 54.5, 52.4, 38.2, 28.4; **HRMS** (ESI) *m/z* calculated for $C_{16}H_{22}FNO_4Na$ ([M+Na]$^+$): 334.1425, found 334.1422; **IR** (thin layer film) 2919, 1714, 1458, 1426, 1146, 1003, 757, 726.

Methyl (*R*)-2-(bis(*tert*-butoxycarbonyl)amino)-3-(4-(fluoromethyl)phenyl)propanoate (34)

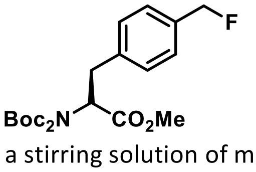

The following is a literature procedure.[11] To a stirring solution of methyl (*R*)-2-((*tert*-butoxycarbonyl)amino)-3-(4-(fluoromethyl)phenyl)propanoate **s39** (248 mg, 0.193 mmol, 1.0 equiv.) and DMAP (24 mg, 0.196 mmol, 1.0 equiv.) in MeCN (2 mL) was added di-*tert*-butyl decarbonate (63 mg, 0.289 mmol, 1.5 equiv.) at room temperature. The reaction mixture was stirred at room temperature under air for 24 h. Upon completion, the reaction mixture was diluted with H_2O (5 mL) and extracted with EtOAc (2 x 5 mL). The combined organic layers were washed with brine (10 mL), dried over anhydrous Na_2SO_4, filtered and concentrated *in vacuo*. Purification by silica gel column chromatography (8 to 10% EtOAc in pentane) afforded the desired product as a colorless oil

(50 mg, 0.122 mmol, 63%). **^{1}H NMR** (400 MHz, $CDCl_3$) *δ* 7.28 (d, *J* = 7.3 Hz, 2H), 7.21 (d, *J* = 7.8 Hz, 2H), 5.31 (d, *J* = 48.0 Hz, 2H), 5.15 (dd, *J* = 10.2, 5.1 Hz, 1H), 3.74 (s, 3H), 3.44 (dd, *J* = 14.0, 5.1 Hz, 1H), 3.22 (dd, *J* = 14.1, 10.2 Hz, 1H), 1.38 (s, 18H); **^{19}F NMR** (377 MHz, $CDCl_3$) δ −205.51 (t, *J* = 48.1 Hz); **^{13}C NMR** (101 MHz, $CDCl_3$) *δ* 170.9, 151.8, 138.6 (d, *J* = 3.1 Hz), 134.6 (d, *J* = 17.1 Hz), 129.9 (d, *J* = 1.5 Hz), 127.9 (d, *J* = 5.7 Hz), 84.5 (d, *J* = 165.7 Hz), 83.2, 59.4 (d, *J* = 1.8 Hz), 52.4, 36.1, 28.0; **HRMS** (ESI) *m/z* calculated for $C_{21}H_{30}FNO_6Na$ ($[M+Na]^+$): 434.1949, found 434.1924; **IR** (neat) 2981, 1748, 1369, 1270, 1224, 1140, 757.

(8*R*,9*S*,13*S*,14*S*)-3-(Fluoromethyl)-13-methyl-6,7,8,9,11,12,13,14,15,16-decahydro-17*H* cyclopenta[*a*]phenanthren-17-one (35)

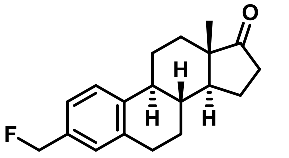

Synthesized according to **GP5** (conditions A) from estrone boronic acid pinacol ester **s12**.[13] All spectroscopic data were in accordance with the literature.[13]

Isopropyl 2-(4-(4-(fluoromethyl)benzoyl)phenoxy)-2-methylpropanoate (36)

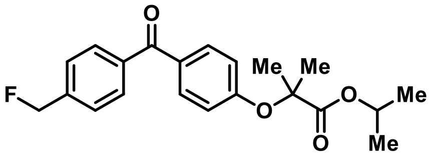

Synthesized according to **GP5** (conditions A), using fenofibrate boronic acid **s15** (370 mg, 0.999 mmol). Purification by silica gel column chromatography (5% EtOAc in pentane) afforded the desired product as an orange oil (37 mg, 0.103 mmol, 10%). **^{1}H NMR** (400 MHz, $CDCl_3$) *δ* 7.76 (dd, *J* = 9.0, 7.0 Hz, 4H), 7.47 (dd, *J* = 8.1, 1.5 Hz, 2H), 6.93 – 6.78 (m, 2H), 5.47 (d, *J* = 47.2 Hz, 2H), 5.09 (hept, *J* = 6.4 Hz, 1H), 1.66 (s, 6H), 1.20 (d, *J* = 6.2 Hz, 6H); **^{19}F NMR** (377 MHz, $CDCl_3$) *δ* −211.78 (t, *J* = 47.3 Hz); **^{13}C NMR** (126 MHz, $CDCl_3$) *δ* 195.2, 173.3, 159.9, 140.3 (d, *J* = 16.6 Hz), 138.5 (d, *J* = 2.7 Hz), 132.2, 130.6, 130.2, 126.8 (d, *J* = 6.4 Hz), 117.4, 84.0 (d, *J* = 168.6 Hz), 79.6, 69.5, 25.5, 21.7; **HRMS** (ESI) *m/z* calculated for $C_{21}H_{24}FO_4$ ($[M+H]^+$): 359.1653, found 359.1642. All spectroscopic data were in accordance with the literature.[19]

A second reaction was performed according to **GP2**, using isopropyl 2-(4-(4-iodobenzoyl)phenoxy)-2-methylpropanoate **s16** (34 mg, 0.0750 mmol). 4-Fluoroanisole (internal standard, 10 µL, 0.0883 mmol) was added and the reaction mixture was diluted with $CDCl_3$, filtered through cotton wool, and analyzed by quantitative ^{19}F NMR, giving an NMR yield of 47%.

4,4,4-Trifluoro-1-(4-(fluoromethyl)phenyl)butane-1,3-dione (s40)

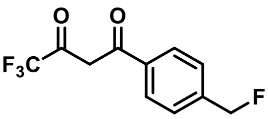

The following is a literature procedure.[52] To a stirring solution of 1-(4-(fluoromethyl)phenyl)ethan-1-one **7** (156 mg, 1.02 mmol, 1.0 equiv.) in anhydrous THF (3.5 mL) under nitrogen at 0 °C was added NaH (60% dispersion in mineral oil, 82 mg, 2.04 mmol, 2.0 equiv.) portion-wise. The resulting mixture was stirred at 0 °C for 30 minutes prior to the addition of ethyl trifluoroacetate (182 µL, 1.53 mmol, 1.5 equiv.). The reaction mixture was stirred at room temperature for 6 h, and was then quenched with ice water (10 mL) and acidified to pH 6 with an aqueous solution of HCl (1 M). The aqueous layer was extracted with EtOAc (3 x 10 mL) and the combined organic layers were dried over anhydrous Na_2SO_4, filtered and concentrated *in vacuo*. The crude product was suspended in hexane and filtered under reduced pressure whilst washing with hexane. The solid was collected and further dried under vacuum, affording the desired product as an orange solid (151 mg, 0.608 mmol, 60%). *Note:* Enol observed as the major tautomer. **^{1}H NMR** (500 MHz, CD_3CN) δ 7.87 (d, J = 7.8 Hz, 2H), 7.44 – 7.42 (m, 2H), 6.01 (s, 1H), 5.42 (d, J = 47.6 Hz, 2H); **^{19}F NMR** (377 MHz, CD_3CN) δ −77.09 (s, 3F), −213.94 (t, J = 48.1 Hz, 1F). **^{13}C NMR** (101 MHz, CD_3CN) δ 187.4, 177.0 (q, J = 36.0 Hz), 144.2 (d, J = 16.9 Hz), 133.8 (d, J = 2.5 Hz), 129.1, 128.4 (d, J = 6.8 Hz), 118.4 (q, J = 282.3 Hz), 94.1 (q, J = 2.1 Hz), 84.6 (d, J = 164.8 Hz); **HRMS** (GC-EI) *m/z* calculated for $C_{11}H_8F_4O_2$ ($[M]^+$): 248.0455, found 248.0473; **IR** (neat) 1620, 1499, 1319, 1186, 1129, 791, 684; **m.p.** 224–227 °C.

4-(5-(4-(Fluoromethyl)phenyl)-3-(trifluoromethyl)-1*H*-pyrazol-1-yl)benzenesulfonamide (37)

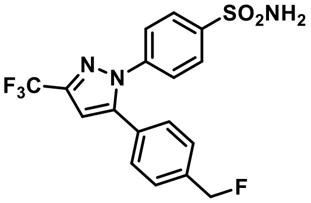

The following is a literature procedure.[53] To a flame-dried round bottom flask equipped with a magnetic stir bar was added 4-hydrazinylbenzenesulfonamide hydrochloride (110 mg, 0.492 mmol, 1.0 equiv.). The flask was evacuated and backfilled with nitrogen three times prior to the addition of a solution of 4,4,4-trifluoro-1-(4-(fluoromethyl)phenyl)butane-1,3-dione **s40** (128 mg, 0.516 mmol, 1.1 equiv.) in EtOH (6.1 mL). The reaction mixture was then heated to reflux whilst stirring under nitrogen for 21 h. The reaction mixture was cooled to room temperature and concentrated *in vacuo*. The crude product was suspended in EtOAc, filtered and the filtrate was concentrated *in vacuo* and purified by silica gel column chromatography (2% MeOH in CH_2Cl_2). The resulting product was repurified by silica gel column chromatography (0 to 4% acetone in pentane), followed by recrystallization from hot toluene. The solid was collected by filtration whilst washing with toluene and dried under vacuum, affording the desired product as a white solid (40 mg, 0.100 mmol, 20%). **^{1}H NMR** (500 MHz, $CDCl_3$) δ 7.92 – 7.90 (m, 2H), 7.48 – 7.45 (m, 2H), 7.40 – 7.38 (m, 2H), 7.27 (d, J = 8.5 Hz, 2H), 6.79 (s, 1H), 5.41 (d, J = 47.3 Hz, 2H), 5.00 (s, 2H); **^{19}F NMR** (470 MHz, $CDCl_3$) δ −62.46 (s, 3F), −210.72 (t, J = 46.9 Hz, 1F); **^{13}C NMR** (126 MHz, $CDCl_3$) δ 144.9, 144.4 (q, J = 38.8 Hz), 142.5, 141.7, 138.0 (d, J = 17.3 Hz), 129.2, 129.0 (d, J = 2.7 Hz),

127.9 (d, J = 6.2 Hz), 127.8, 125.7, 121.1 (q, J = 269.4 Hz), 107.0, 83.8 (d, J = 168.1 Hz); **HRMS** (ESI) m/z calculated for $C_{17}H_{13}F_4N_3O_2SNa$ ($[M+Na]^+$): 422.0557, found 422.0553; **IR** (neat) 1684, 1597, 1411, 1237, 1159, 1129, 1099, 974, 846, 831, 765; **m.p.** 151–153 °C.

3-Cyclopropyl-1-(2-fluoro-4-(fluoromethyl)phenyl)-5-hydroxy-6,8-dimethylpyrido[2,3-*d*]pyrimidine-2,4,7(1*H*,3*H*,8*H*)-trione (38)

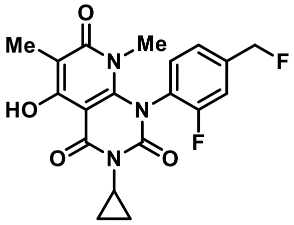

To an oven-dried 4 mL glass vial equipped with a magnetic stir bar were added 3-cyclopropyl-1-(2-fluoro-4-iodophenyl)-5-hydroxy-6,8-dimethylpyrido[2,3-*d*]pyrimidine-2,4,7(1*H*,3*H*,8*H*)-trione (145 mg, 0.300 mmol, 1.0 equiv.), 1,3-dioxoisoindolin-2-yl 2-fluoroacetate **s34** (80 mg, 0.358 mmol, 1.2 equiv.), zinc (39 mg, 0.596 mmol, 2.0 equiv.) and $NiBr_2{\cdot}dtbbpy$ (18 mg, 0.0370 mmol, 0.12 equiv.). The vial was sealed, and evacuated and backfilled with nitrogen three times. Anhydrous DMA (375 µL, 0.8 M) was then added, followed by TMSCl (114 µL, 0.898 mmol, 3.0 equiv.). The reaction mixture was then stirred at room temperature for 1 h. Purification of the reaction mixture directly by silica gel column chromatography (10 to 50% EtOAc in pentane) afforded the desired product as a white solid (61 mg, 0.157 mmol, 52%).^{1}**H NMR** (500 MHz, $CDCl_3$) δ 11.94 (s, 1H), 7.30 (d, J = 10.2 Hz, 1H), 7.24 – 7.20 (m, 2H), 5.44 (d, J = 46.8 Hz, 2H), 2.90 (s, 3H), 2.77 (dq, J = 7.2, 3.6 Hz, 1H), 2.00 (s, 3H), 1.21 – 1.17 (m, 2H), 0.89 – 0.86 (m, 2H); ^{19}**F NMR** (377 MHz, $CDCl_3$) δ −115.91 (m, 1F), −214.51 (t, J = 46.8 Hz, 1F); ^{13}**C NMR** (126 MHz, $CDCl_3$) δ 165.7, 163.8, 160.9, 159.4, 157.4, 149.2 (d, J = 217.1 Hz), 141.3 (dd, J = 18.5, 7.4 Hz), 129.7, 125.8 (dd, J = 12.8, 2.3 Hz), 123.0 (dd, J = 6.9, 3.4 Hz), 115.2 (dd, J = 20.8, 7.4 Hz), 103.5, 90.4, 82.6 (d, J = 171.4 Hz), 34.8, 25.6, 8.6, 8.5, 8.3; **HRMS** (ESI) m/z calculated for $C_{19}H_{17}F_2N_3O_4Na$ ($[M+Na]^+$): 412.1079, found 412.1071; **IR** (neat) 2361, 1727, 1680, 1642, 1489, 1432, 793, 747; **m.p.** 143–145 °C.

***Note**: compound **35** was found to decompose upon aqueous work-up, hence reaction mixture was loaded onto silica column directly.*

4-(4-Fluoro-3-(4-(4-(fluoromethyl)benzoyl)piperazine-1-carbonyl)benzyl)phthalazin-1(2*H*)-one (39)

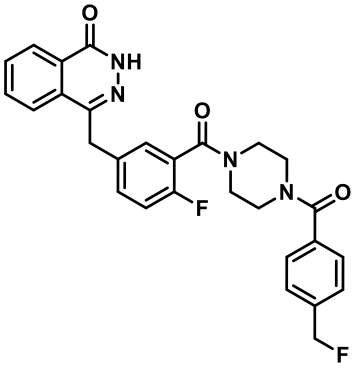

Synthesized according to a literature procedure.[54] All spectroscopic data were in accordance with the literature.[54]

Synthesis of $[D_2]$**1**

5-((Methyl-*d*$_3$)thio)-1-phenyl-1*H*-tetrazole (s41)

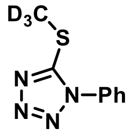

The following is a literature procedure.[55] To a flame-dried round bottom flask equipped with a magnetic stir bar was added NaOH (1.1 g, 26.8 mmol, 2.5 equiv.). The flask was evacuated and backfilled with nitrogen three times prior to the addition of anhydrous EtOH (32 mL). 1-Phenyl-1*H*-tetrazole-5-thiol (1.9 g, 10.7 mmol, 1.0 equiv.) was added under nitrogen, and the reaction mixture was stirred at room temperature for 1 h. CD_3I (1 mL, 16.1 mmol, 1.5 equiv.) was added dropwise, and the reaction mixture was stirred at room temperature for a further 3 h. The reaction mixture was then concentrated *in vacuo*, and H_2O (20 mL) was added. The aqueous layer was extracted with EtOAc (3 x 20 mL), and the combined organic layers were dried over anhydrous Na_2SO_4, filtered, and concentrated *in vacuo*. Purification by silica gel column chromatography (10 to 20% EtOAc in pentane) afforded the desired product as a white solid (2.06 g, 10.6 mmol, 99%). **^{1}H NMR** (500 MHz, $CDCl_3$) δ 7.58 – 7.48 (m, 5H), *>99% deuterium incorporation*; **^{13}C NMR** (126 MHz, $CDCl_3$) δ 155.0, 133.7, 130.2, 129.9, 123.7, 14.9 (hept, J = 22.0 Hz); **HRMS** (ESI) *m/z* calculated for $C_8H_5D_3N_4SNa$ ($[M+Na]^+$): 218.0550, found 218.0543; **IR** (neat) 2360, 1710, 1637, 1383, 1320, 1174, 994, 954, 834, 790; **m.p** 73–77 °C.

5-((Methyl-*d*$_3$)sulfonyl)-1-phenyl-1*H*-tetrazole (s42)

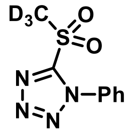

5-((Methyl-*d*$_3$)thio)-1-phenyl-1*H*-tetrazole **s41** (1.0 g, 5.12 mmol, 1.0 equiv.) was suspended in MeCN (13 mL) and $NaIO_4$ (3.3 g, 15.4 mmol, 3.0 equiv.), $RuCl_3 \cdot xH_2O$ (53 mg, 0.256 mmol, 0.050 equiv.) and H_2O (5 mL) were added. The reaction mixture was stirred at room temperature under air for 0.5 h. Upon completion, the reaction mixture was filtered through a pad of silica, washing with EtOAc. The filtrate was washed with brine (30 mL), dried over anhydrous Na_2SO_4, filtered and concentrated *in vacuo*. Purification by silica gel column chromatography (5 to 25% EtOAc in pentane) afforded the desired product as a white solid (1.06 g, 4.65 mmol, 91%). **^{1}H NMR** (400 MHz, $CDCl_3$) δ 7.74 – 7.67 (m, 2H), 7.66 – 7.58 (m, 3H), *>99% deuterium incorporation*; **^{13}C NMR** (151 MHz, $CDCl_3$) δ 154.1, 133.0, 131.6, 129.9, 125.0, 43.3 (hept, J = 21.5 Hz); **HRMS** (ESI) *m/z* calculated for $C_8H_5D_3N_4O_2SNa$ ($[M+Na]^+$): 250.0449, found 250.0445; **IR** (neat) 2360, 1713, 1498, 1335, 1165, 792, 763, 729, 689; **m.p** 75–77 °C.

5-((Fluoromethyl-d_2)sulfonyl)-1-phenyl-1*H*-tetrazole ([D_2]3)

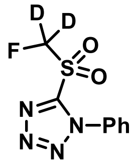

The following is an adapted literature procedure.[56] To a flame-dried round bottom flask equipped with a magnetic stir bar was added 5-((methyl-d_3)sulfonyl)-1-phenyl-1*H*-tetrazole **s42** (625 mg, 2.75 mmol, 1.0 equiv.). The flask was evacuated and backfilled with nitrogen three times prior to the addition of anhydrous THF (5 mL). The reaction mixture was cooled to −78 °C, and *n*BuLi (2.5 M in hexanes, 1.2 mL, 3.03 mmol, 1.1 equiv.) was added dropwise. After stirring for 10 minutes at −78 °C, NFSI (1.04 g, 3.30 mmol, 1.2 equiv.) was added portion-wise as a solid over 20 minutes, and the reaction mixture was stirred whilst warming to room temperature for 18 h. The reaction was then cooled to −78 °C, quenched by the addition of D_2O (5 mL), and stirred at room temperature for 0.5 h. The aqueous layer was extracted with EtOAc (3 x 10 mL) and the combined organic layers were dried over anhydrous Na_2SO_4, filtered, and concentrated *in vacuo*. Purification by silica gel column chromatography (15% EtOAc in pentane) afforded the desired product as a yellow oil (138 mg, 0.565 mmol, 21%). **^{1}H NMR** (500 MHz, $CDCl_3$) δ 7.68 – 7.60 (m, 5H); **^{19}F{^{1}H} NMR** (376 MHz, $CDCl_3$) δ −208.94 (s, d_0, 0.06), −209.48 (t, J = 7.2 Hz, d_1, 2.15), −210.02 (p, J = 6.9 Hz, d_2, 97.79), *99% deuterium incorporation*; **^{13}C{^{1}H,^{19}F} NMR** (126 MHz, $CDCl_3$) δ 151.7, 132.8, 132.0, 130.0, 125.2, 91.2 (p, J = 25.7 Hz); **HRMS** (ESI) *m/z* calculated for $C_8H_6D_2FN_4O_2SNa$ ([M+H]$^+$): 245.0472, found 245.0463; **IR** (neat) 2360, 1497, 1371, 1180, 1117, 980, 790, 769, 689; **m.p** 56–58 °C.

Cyclic Voltammetry

Cyclic Voltammetry was conducted on an IKA ElectraSyn 2.0 using a three-electrode cell configuration. This included a glassy carbon working electrode, a platinum coated copper wire as the counter-electrode and a silver wire as the reference electrode in 3 M KCl solution. 0.15 M solutions of tetrabutylammonium hexafluorophosphate in dry, degassed MeCN were added to the desired compound (15–25 mg), and the resulting solution was also degassed by sparging with nitrogen for 10 min prior to measurements. A scan rate of 100 mV s^{-1} was used. Values were converted from Fc/Fc+ to SCE according to the following formula: $E_{1/2}^{Red}$ vs Ag/Ag^+ = $E_{1/2}^{Red}$ vs SCE + 0.045.

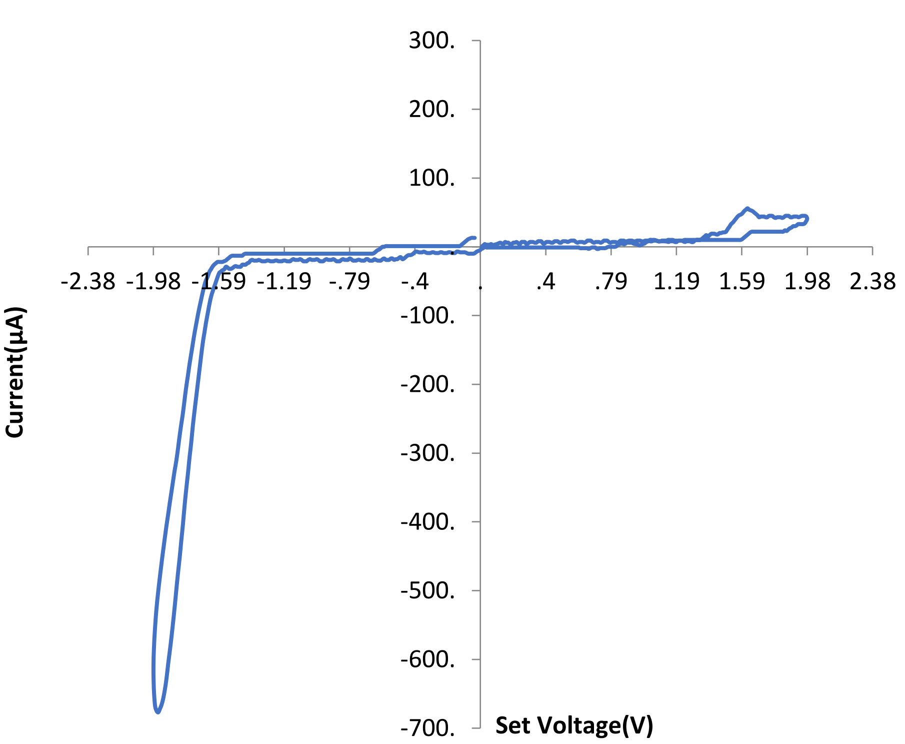

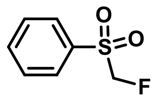

1

$E_{1/2}^{Red}$ vs Ag/Ag^+:

-1.95 V

$E_{1/2}^{Red}$ vs SCE:

-2.00 V

Figure S1. Cyclic voltammogram of compound **1**.

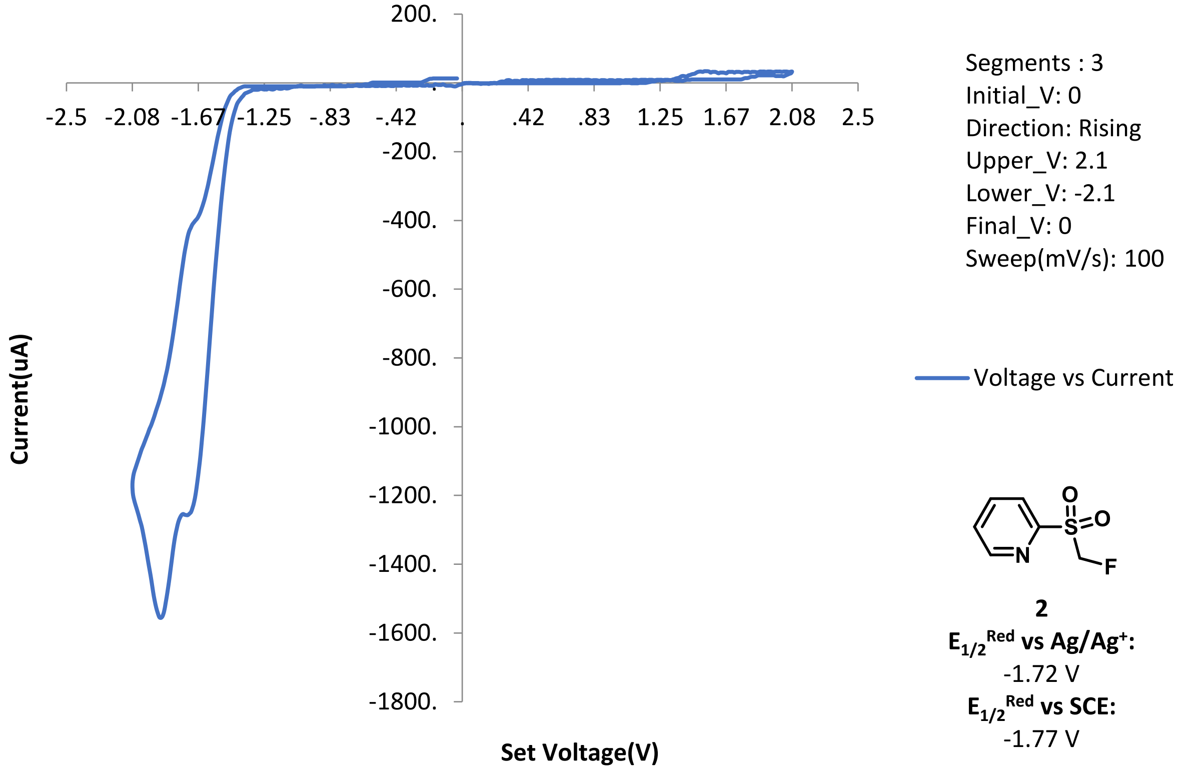

Figure S2. Cyclic voltammogram of compound **2**.

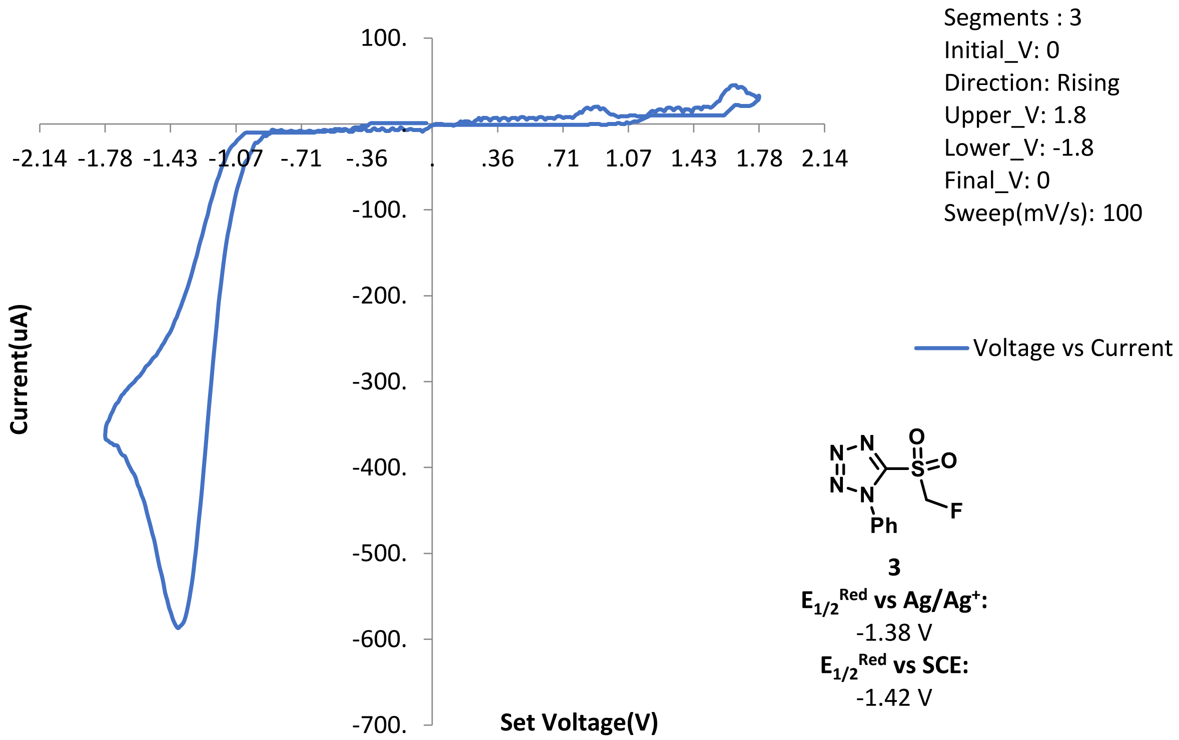

Figure S3. Cyclic voltammogram of compound **3**.

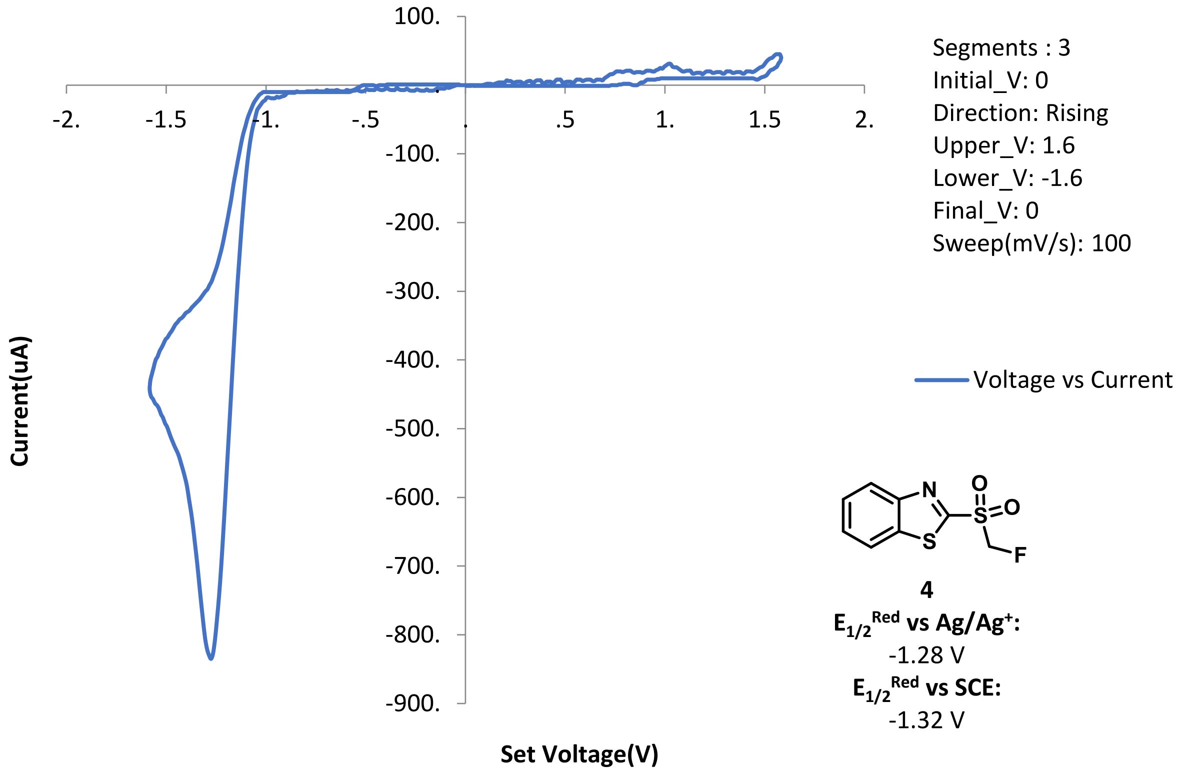

Figure S4. Cyclic voltammogram of **4**.

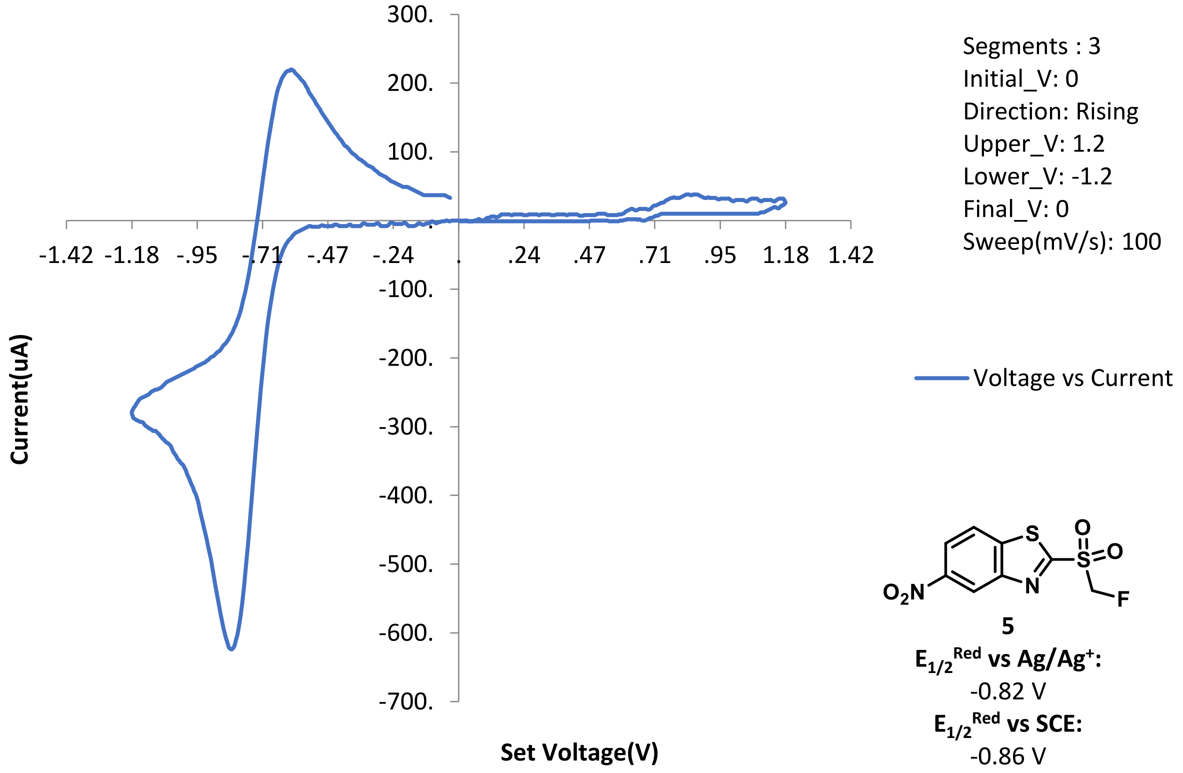

Figure S5. Cyclic voltammogram of compound **5**.

Radiochemistry

General Experimental Details

Caution: *Fluorine-18 undergoes radioactive decay via positron emission, resulting in the release of ionizing gamma radiation upon annihilation, which presents a serious health hazard. All experiments with fluorine-18 were conducted in lead-shielded hot cells equipped with external telemanipulators or using appropriate lead shielding for safe handling.*

Automated radiochemistry experiments

[^{18}F]Fluoride was produced using an IBA Cyclon 18/9 (PETIC, UK) or GE MINITrace Qilin (OxIME, UK) cyclotron using the ^{18}O(*p*,*n*)^{18}F reaction. All experiments were performed with a TRASIS AllinOne radiosynthesizer. Analysis of radiolabelled products for identity, radiochemical purity (RCP) and molar activity (A_m) was achieved using radio-high-performance liquid chromatography (radio-HPLC)- see HPLC conditions for further details. Approximate UV-radio detector offset = 0.1 min.

HPLC conditions

Analytical

Conditions A-B: Agilent 1200 equipped with a UV detector and LabLogic gamma-RAM Model 4 detector

Conditions C: Agilent 1200 equipped with a UV detector and LabLogic FlowRAM with 1” NaI/PMT detector

Conditions D: LabLogic LogiChrom HPLC equipped with a UV detector and 1” NaI/PMT detector

Conditions E: Shimadzu LC-40 equipped with a PDA detector and LabLogic FlowRAM with 1” NaI/PMT detector

Conditions F: Shimadzu LC-40 equipped with a PDA detector and LabLogic FlowRAM with 2” well-type NaI/PMT detector (50 µL flow cell)

Semi-Preparative

Conditions G-H: TRASIS AllInOne integrated HPLC system

***Conditions A**: Analytical*

Flow rate = 1.0 mL/min

Temperature = 25 °C

Wavelength = 220 nm (unless otherwise specified)

Column: Agilent C18 Eclipse Plus 80Å 150 x 4.6 mm LC column

HPLC gradient: water/MeCN

- 0–1 min (25% MeCN) isocratic
- 1–10 min (25% MeCN to 95% MeCN) linear increase
- 10–16 min (95% MeCN) isocratic
- 16–18 min (95% MeCN to 25% MeCN) linear decrease
- 18–20 min (25% MeCN) isocratic

***Conditions B**: Analytical*

Flow rate = 1.0 mL/min

Temperature = 25 °C

Wavelength = 220 nm (unless otherwise specified)

Column: Agilent C18 Eclipse Plus 80Å 150 x 4.6 mm LC column

HPLC gradient: water/MeCN

0–1 min (5% MeCN) isocratic

1–10 min (5% MeCN to 95% MeCN) linear increase

10–16 min (95% MeCN) isocratic

16–18 min (95% MeCN to 5% MeCN) linear decrease

18–20 min (5% MeCN) isocratic

***Conditions C**: Analytical*

Flow rate = 1.0 mL/min

Temperature = 25 °C

Wavelength = 220 nm (unless otherwise specified)

Column: Agilent C18 Eclipse Plus 80Å 150 x 4.6 mm LC column

HPLC gradient: water/MeCN

0–1 min (25% MeCN) isocratic

1–10 min (25% MeCN to 95% MeCN) linear increase

10–16 min (95% MeCN) isocratic

16–18 min (95% MeCN to 25% MeCN) linear decrease

18–20 min (25% MeCN) isocratic

***Conditions D**: Analytical*

Flow rate = 1.0 mL/min

Temperature = 25 °C

Wavelength = 220 nm (unless otherwise specified)

Column: Agilent C18 Eclipse Plus 80Å 150 x 4.6 mm LC column

HPLC gradient: water/MeCN

0–1 min (25% MeCN) isocratic

1–10 min (25% MeCN to 95% MeCN) linear increase

10–16 min (95% MeCN) isocratic

16–18 min (95% MeCN to 25% MeCN) linear decrease

18–20 min (25% MeCN) isocratic

***Conditions E**: Analytical*

Flow rate = 1.0 mL/min

Temperature = 25 °C

Wavelength = 220 nm (unless otherwise specified)

Column: Agilent C18 Eclipse Plus 80Å 150 x 4.6 mm LC column

HPLC gradient: water/MeCN

0–2 min (25% MeCN) isocratic

2–10 min (25% MeCN to 95% MeCN) linear increase

10–15 min (95% MeCN) isocratic

15–17 min (95% MeCN to 25% MeCN) linear decrease

17–20 min (25% MeCN) isocratic

***Conditions F**: Analytical*

Flow rate = 1.0 mL/min

Temperature = 25 °C

Wavelength = 207 nm

Column: Gemini C18 110Å 250 x 4.6 mm LC column

HPLC gradient: water/MeCN

0–2 min (25% MeCN) isocratic

2–15 min (25% MeCN to 65% MeCN) linear increase

15–16.5 min (65% MeCN) isocratic

16.5–18 min (65% MeCN to 25% MeCN) linear decrease

18–20 min (25% MeCN) isocratic

***Conditions G**: Semi-Preparative Purification*

Flow rate = 4.0 mL/min

Temperature = room temperature

Wavelength = 254 nm

Column: Phenomenex Gemini 5 µm 250 x 10 mm LC column

Isocratic: water/MeCN

***Conditions H**: Semi-Preparative Purification*

Flow rate = 3.0 mL/min

Temperature = room temperature

Wavelength = 254 nm

Column: Phenomenex Synergi™ 4 µm Hydro RP 80Å 250 x 10 mm LC column

Isocratic: water/MeCN

Screening of ^{18}F-Fluoromethyl Sulfone Reagent Precursor

Automated [^{18}F]KF Elution and Drying

[^{18}F]Fluoride (5 GBq) in [^{18}O]water was received directly from the cyclotron, into the Trasis AllInOne radiosynthesis platform. The [^{18}F]fluoride was separated from ^{18}O-enriched-water using an anion exchange cartridge (Waters Sep-Pak AccellPlus QMA Carbonate Plus Light Cartridge, activated with H_2O (10 mL) prior to use) and released with a solution of K_{222} (7.5 mg) and K_2CO_3 (1.5 mg) in MeCN/H_2O (0.75 mL, 4:1, v/v). The solution was dried over a period of 20 min by azeotropic drying using dry MeCN (3 x 0.7 mL) under a flow of nitrogen at 110 °C.

Manual ^{18}F-Radiofluorination Reaction Procedure

To a 3 or 5 mL V-vial containing tetrazole sulfide **s1**, **s2**, or **s3** (0.04 mmol), K_2CO_3 (1.5 mg) and K_{222} (7.5 mg) in MeCN (1 mL) was added [^{18}F]KF (5–30 MBq in MeCN (20–50 µL)). The mixture was stirred at 110 °C for 10 min, and then was cooled to room temperature and quenched by addition of a MeCN/H_2O solution (1:1, 300 µL). An aliquot of this mixture was further analyzed by radio-HPLC, conditions A (Table S13). Radiochemical conversion (RCC, %) was determined by radio-HPLC.

Table S13. Screening of the radiofluorination of precursors **s1**, **s2** and **s3** for the radiosynthesis of [^{18}F]**s26**.

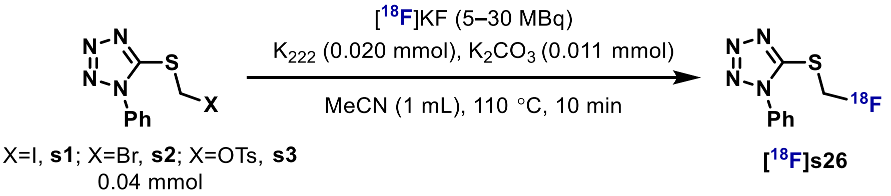

X	Deviation	RCC [^{18}F]S29 (%)
I	–	18 ± 8$_{(n=4)}$
I	w/o K_{222} or K_2CO_3	6$_{(n=1)}$
I	+ AgOTs (0.04 mmol)	7$_{(n=1)}$
Br	–	50 ± 8$_{(n=4)}$
OTs	–	3$_{(n=1)}$

Automated ^{18}F-Fluoromethyl Sulfone Reagent Radiosynthesis

Automated Radiosynthesis of [^{18}F(D$_2$)]**3**

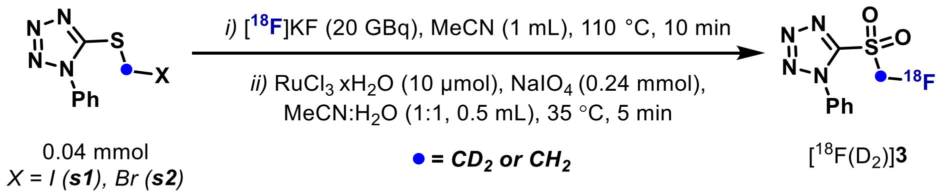

The automated radiosynthesis of [^{18}F(D$_2$)]**3** was carried out using a Trasis AllinOne automated radiosynthesis platform, using a custom cassette set-up (Figure S6). A vial charged with K_2CO_3 (1.5 mg), Kryptofix® 222 (7.5 mg), MeCN (0.6 mL) and H_2O (0.15 mL) was inserted at position 2. A vial charged with 5-((iodomethyl)thio)-1-phenyl-1*H*-tetrazole **s1** (12.7 mg, 0.04 mmol) or 5-((bromomethyl)thio)-1-phenyl-1*H*-tetrazole **s2** (10.8 mg, 0.04 mmol) or 5-((bromomethyl-*d*$_2$)thio)-1-phenyl-1*H*-tetrazole [D$_2$]**s2** (10.9 mg, 0.04 mmol) in anhydrous MeCN (1.0 mL) was inserted at position 9. A vial charged with $RuCl_3$ hydrate (2 mg), $NaIO_4$ (52 mg), H_2O (0.25 mL) and MeCN (0.25 mL) was inserted at position 10. A vial was filled with dry MeCN (approximately 10 mL) and placed at position 8. A vial was filled with dry DMI (approximately 10 mL) and placed at position 17. A water bag was placed in position 14. The HPLC collection vial was filled with H_2O (20 mL) and placed in position 34. A Waters Sep-Pak AccellPlus QMA Carbonate Plus Light Cartridge was pre-conditioned with H_2O prior to use and placed in position 5. A Waters Sep-Pak C18 Plus Short cartridge was activated with EtOH (10 mL) and then H_2O (10 mL) prior to use and was placed in position 33.

[^{18}F]Fluoride (20–40 GBq) in [^{18}O]water was received directly from the cyclotron, into the Trasis AllInOne radiosynthesis platform. The [^{18}F]fluoride was separated from the water by trapping on the QMA cartridge, followed by elution with the solution from the vial in position 2 into the reactor. The [^{18}F]fluoride was then dried azeotropically, and once this was complete, the ^{18}F-fluorination of the precursor (**s1**, **s2**, or [D$_2$]**s2**) proceeded in the same reactor at 110 °C for 10 minutes. The reactor was then cooled and the contents of the vial at position 10 were added to the same reactor. The oxidation reaction proceeded at 35 °C for 5 minutes. The crude reaction mixture was diluted with H_2O and MeCN and transferred to the HPLC injection loop for semi-preparative reverse phase HPLC purification (conditions G, 35:65 MeCN:H_2O). The desired purified product was collected in the vial at position 34. This solution was diluted further with H_2O for reformulation using a C18 Plus cartridge. [^{18}F]**3** was then eluted with DMI (2.0 mL) into a vial. Activity yield data is given (Table S14).

Table S14. Activity yields (AY) for the automated synthesis of [^{18}F(D$_2$)]**3**.

X	$-CH_2X$ or $-CD_2X$	Starting activity (GBq)	AY (n.d.c., GBq)	RCP (%)	% AY
I	H	20	0.367	97	2
I	H	20	0.572	97	3
I	H	20	0.746	>99	4
Br	H	20	0.848	>99	4
Br	H	20	1.23	>99	6
Br	H	20	0.952	>99	5
Br	H	20	1.02	>99	5
Br	H	20	1.10	>99	6
Br	H	20	1.12	>99	6
Br	H	20	0.959	>99	5
Br	H	20	0.555	>99	3
Br	H	20	1.80	>99	9
Br	H	20	0.897	>99	4
Br	H	20	0.610	>99	3
Br	H	20	1.12	>99	6
Br	D	20	1.32	>99	7
Br	D	20	1.06	>99	5
Br*	D	40	2.32	>99	6

*Oxidant loading: $RuCl_3$ hydrate (4 mg), $NaIO_4$ (104 mg), H_2O (1.0 mL)

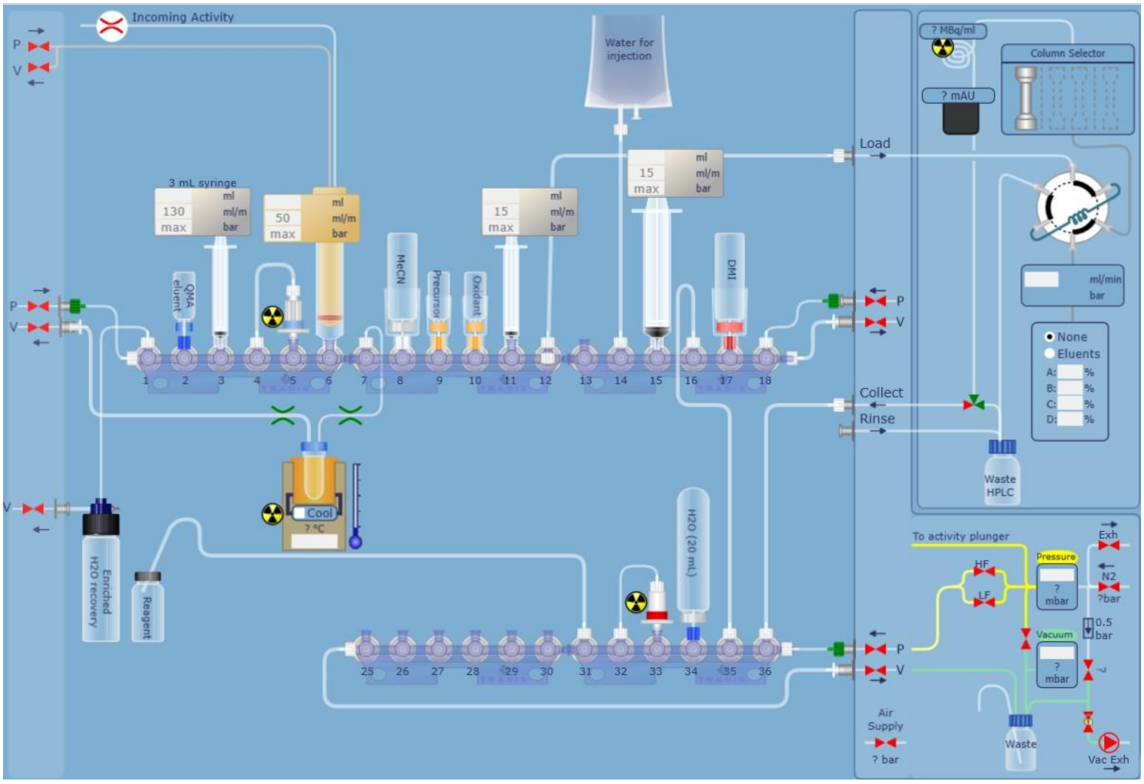

Figure S6. Cassette layout for the automated synthesis of [^{18}F]**3** and [^{18}F]**4** on the Trasis AllInOne platform.

Calculation of Molar Activity of [^{18}F,D_2]**3**

Procedure for the calibration curve of **3**: A calibration curve for authentic reference [D_2]**3** was recorded by preparing samples of a range of concentrations by serial dilution, starting with a solution of [D_2]**3** (7.7 mg) in MeCN (1.0 mL) (Figure S7). These were injected onto an HPLC (10 µL injection volume from a 1.0 mL stock, HPLC conditions F at 214 nm) and the UV response was measured by integrating the peak of interest.

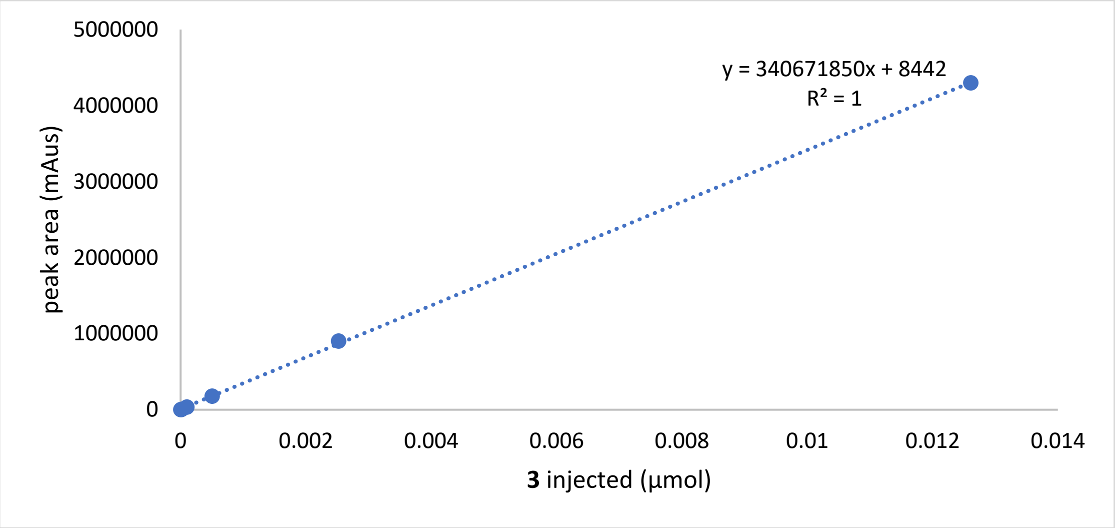

Figure S7. HPLC calibration curve for authentic reference [D_2]**3**.

Table S15. Molar activity calculation of [^{18}F,D_2]**3**.

Measurement	Activity injected (MBq, d.c. EOS)	Peak area (mAU.s)	3 injected (µmol)	A_m (GBq µmol^{-1})
**1**	0.396	1141	3.35×10^{-6}	118.2
**2**	1.02	3712	1.09×10^{-5}	93.6
**3**	1.18	3462	1.02×10^{-5}	115.7
			**Average:**	**109 ± 11**

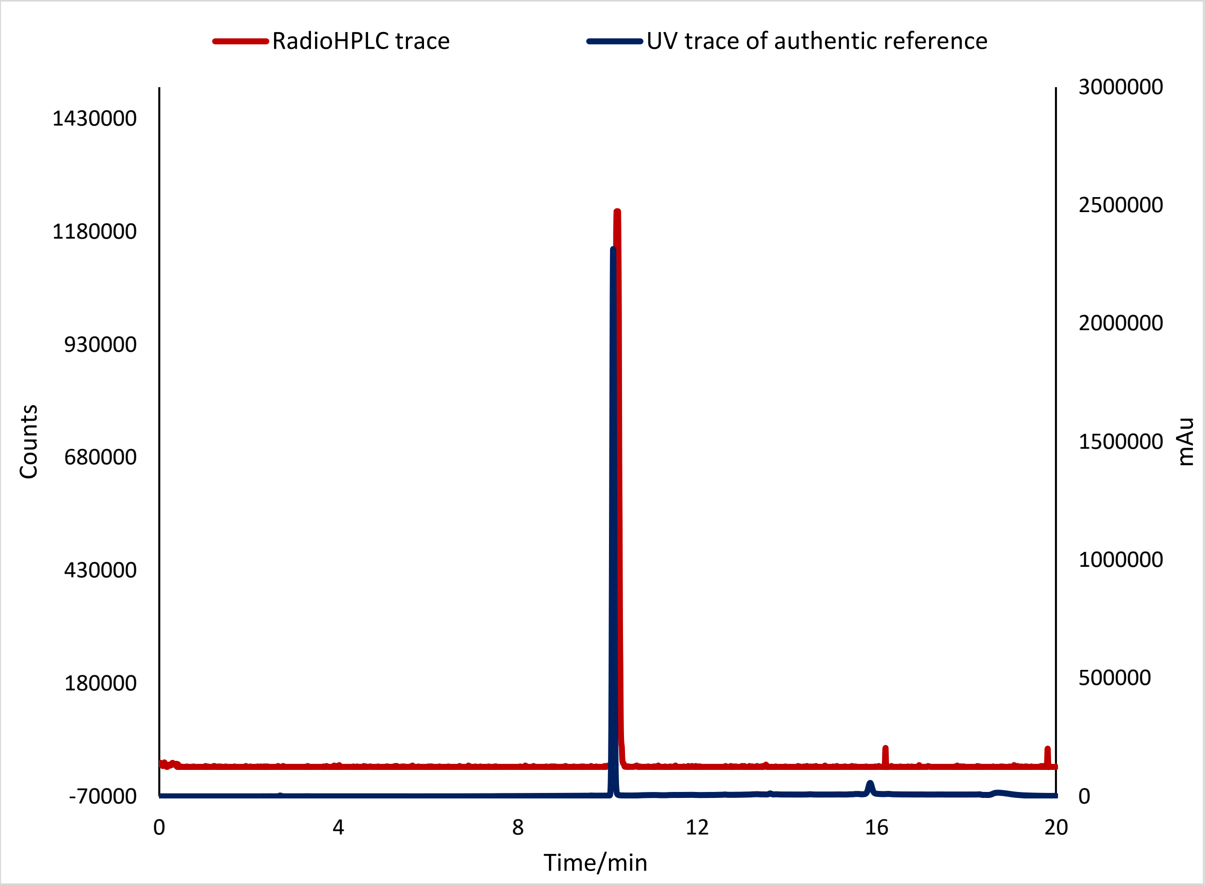

Figure S8. Radio and UV-HPLC trace overlays for isolated [^{18}F,D_2]**3**.

Automated Radiosynthesis of [18F]**4**

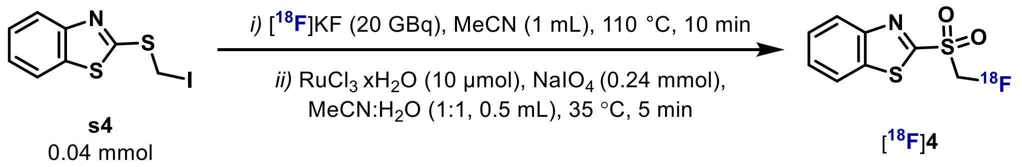

The automated radiosynthesis of [18F]**4** was carried out using a Trasis AllinOne automated radiosynthesis platform, using a custom cassette set-up (Figure S6). A vial charged with K_2CO_3 (1.5 mg), Kryptofix® 222 (7.5 mg), MeCN (0.6 mL) and H_2O (0.15 mL) was inserted at position 2. A vial charged with 2-((iodomethyl)sulfonyl)benzo[*d*]thiazole **s4** (12.3 mg, 0.04 mmol) and MeCN (1.0 mL) was inserted at position 9. A vial charged with $RuCl_3$ hydrate (2 mg), $NaIO_4$ (52 mg), H_2O (0.25 mL) and MeCN (0.25 mL) was inserted at position 10. A vial was filled with dry MeCN (approximately 10 mL) and placed at position 8. A vial was filled with dry DMI (approximately 10 mL) and placed at position 17. A water bag was placed in position 14. The HPLC collection vial was filled with H_2O (20 mL) and placed in position 34. A Waters Sep-Pak AccellPlus QMA Carbonate Plus Light Cartridge was pre-conditioned with H_2O prior to use and placed in position 5. A Waters Sep-Pak C18 Plus Short cartridge was activated with EtOH (10 mL) and then H_2O (10 mL) prior to use and was placed in position 33.

[18F]Fluoride (20 GBq) in [18O]water was received directly from the cyclotron, into the Trasis AllInOne radiosynthesis platform. The [18F]fluoride was separated from the water by trapping on the QMA cartridge, followed by elution with the solution from the vial in position 2 into the reactor. The [18F]fluoride was then dried, and once this was complete, the 18F-fluorination of **s4** proceeded in the same reactor at 110 °C for 10 minutes. The reactor was then cooled and the contents of the vial at position 10 were added to the same reactor. The oxidation reaction proceeded at 35 °C for 5 minutes. The crude reaction mixture was diluted with H_2O and MeCN and transferred to the HPLC injection loop for semi-preparative reverse phase HPLC purification (conditions G, 50:50 MeCN:H_2O). The desired purified product was collected in the vial at position 34. This solution was diluted further with H_2O for reformulation using a C18 Plus cartridge. [18F]**4** was then eluted with DMI (2.0 mL) into a vial, AY = 370 $MBq_{(n=1)}$ (n.d.c.), RCP > 99%.

Note*: unoptimized reformulation procedure caused a decreased isolated activity of [18F]***4***.*

Reaction Optimization

General Procedure for the Cross-Coupling of ^{18}F-Fluoromethyl Sulfones and Aryl Halides

To a 1 mL V-vial (see below for details regarding reaction set-up) containing aryl halide (0.05 mmol), nickel species, ligand, and reductant in anhydrous solvent was added [^{18}F]**3**, [^{18}F,D_2]**3**, or [^{18}F]**4** (5–30 MBq in DMI (20–50 µL)). The mixture was heated whilst stirring at 650 ppm for 20 min, and then cooled to room temperature and quenched by addition of a MeCN/H_2O solution (1:1, 300 µL). An aliquot of this mixture was filtered through a Whatman® Mini-UniPrep® syringeless filter vial and analyzed by radio-HPLC (conditions A-D). RCC (%) was then determined by radio-HPLC.

Reaction set-up

A 1 mL Wheaton V-vial® (13 mm diameter × 44 mm height) with open-top cap fitted with PTFE/silicon septum and equipped with Wheaton magnetic spin-vane for 1 mL V-Vial® was used (pictured right).

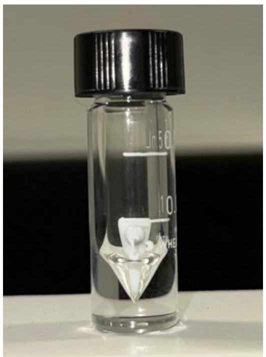

Due to the heterogenous nature of the reaction, efficient stirring was found to be crucial for reproducibility, combined with the use of zinc nanopowder at 650 rpm stirring speed.

Zinc nanopowder was purchased from Sigma Aldrich, 40−60 nm average particle size, ≥99% trace metal basis and was used as received.

Comparison of the use of zinc nanopowder (left) and zinc powder (right):

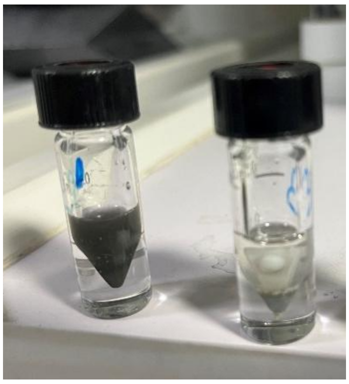

Table S16. Reductant screen. Zn = zinc powder, Zn_{nano} = zinc nanopowder.

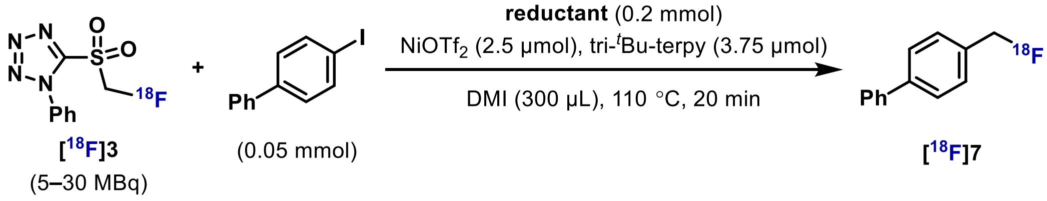

Reductant	RCC [^{18}F]7 (%)
Zn	43 ± 30$_{(n=3)}$
Zn_{nano}	79 ± 11$_{(n=13)}$
Mn	0$_{(n=1)}$

Table S17. Solvent screen.

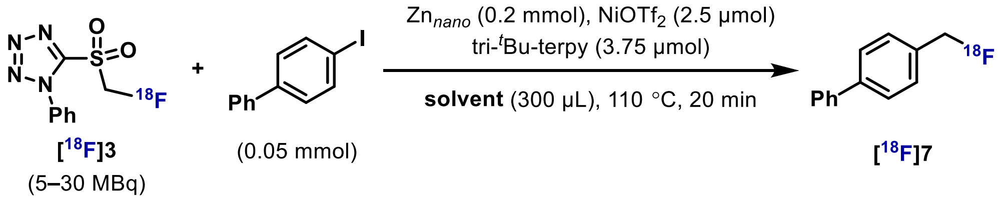

Solvent	RCC [^{18}F]7 (%)
DMI	79 ± 11$_{(n=13)}$
DMF	43 ± 29$_{(n=2)}$
DMA	42 ± 34$_{(n=2)}$
DME	73$_{(n=1)}$
MeCN	58$_{(n=1)}$
Toluene	75$_{(n=1)}$

Table S18. Ligand screen.

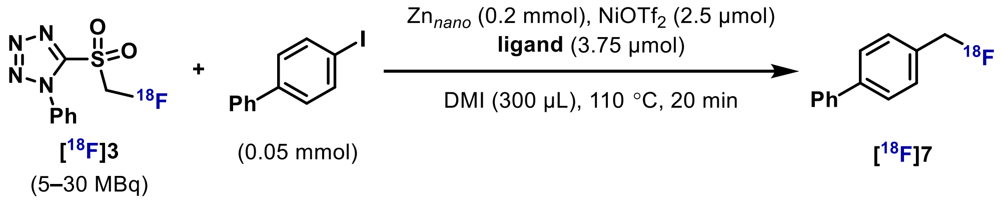

Ligand	RCC [^{18}F]7 (%)
tri-^{t}Bu-terpy	79 ± 11$_{(n=13)}$
terpy	74 ± 10$_{(n=2)}$
dtbbpy	89$_{(n=1)}$
^{t}BuBpyCAM^{CN}	62$_{(n=1)}$

Table S19. Aryl (pseudo)halide screen.

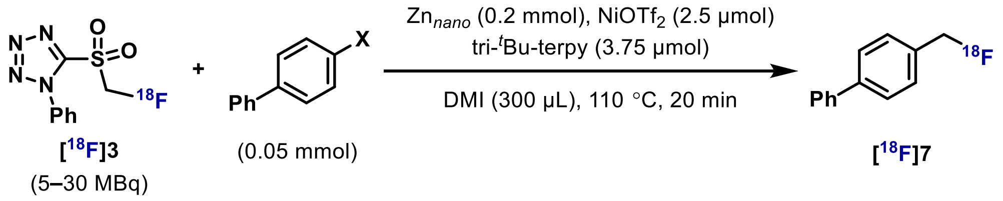

X	RCC [^{18}F]7 (%)
I	79 ± 11$_{(n=13)}$
Br	72 ± 2$_{(n=2)}$
Cl	43 ± 12$_{(n=3)}$
OTf	23 ± 2$_{(n=2)}$

Determination of Deuterium Incorporation

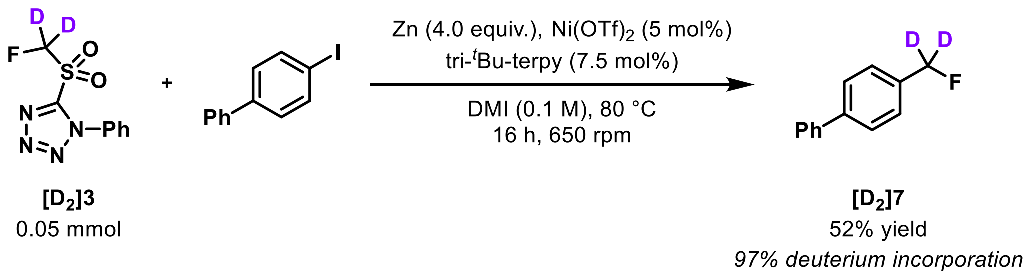

To an oven-dried 1.75 mL glass vial equipped with a magnetic stir bar were added 5-((fluoromethyl-d_2)sulfonyl)-1-phenyl-1*H*-tetrazole [D_2]**3** (12.2 mg, 0.050 mmol, 1.0 equiv.), 4-iodo-1,1'-biphenyl (21 mg, 0.075 mmol, 1.5 equiv.), activated zinc powder (13 mg, 0.20 mmol, 4.0 equiv.), $NiOTf_2$ (0.9 mg, 2.50 µmol, 0.05 equiv.) and tri-^{t}Bu-terpy (1.5 mg, 3.75 µmol, 0.075 equiv.). Anhydrous DMI (0.5 mL) was then added and the reaction mixture was closed under air. The vial was wrapped with Parafilm and stirred at 80 °C, at 650 rpm, in a heating block for 16 h. The reaction mixture was cooled to room temperature, 4-fluoroanisole (internal standard, 10 µL, 0.088 mmol) was added and the reaction mixture was diluted with $CDCl_3$, filtered through cotton wool, and analyzed by quantitative ^{19}F NMR spectroscopy (Figure S9). Yield (52%) and deuterium incorporation (97%) were thus determined.

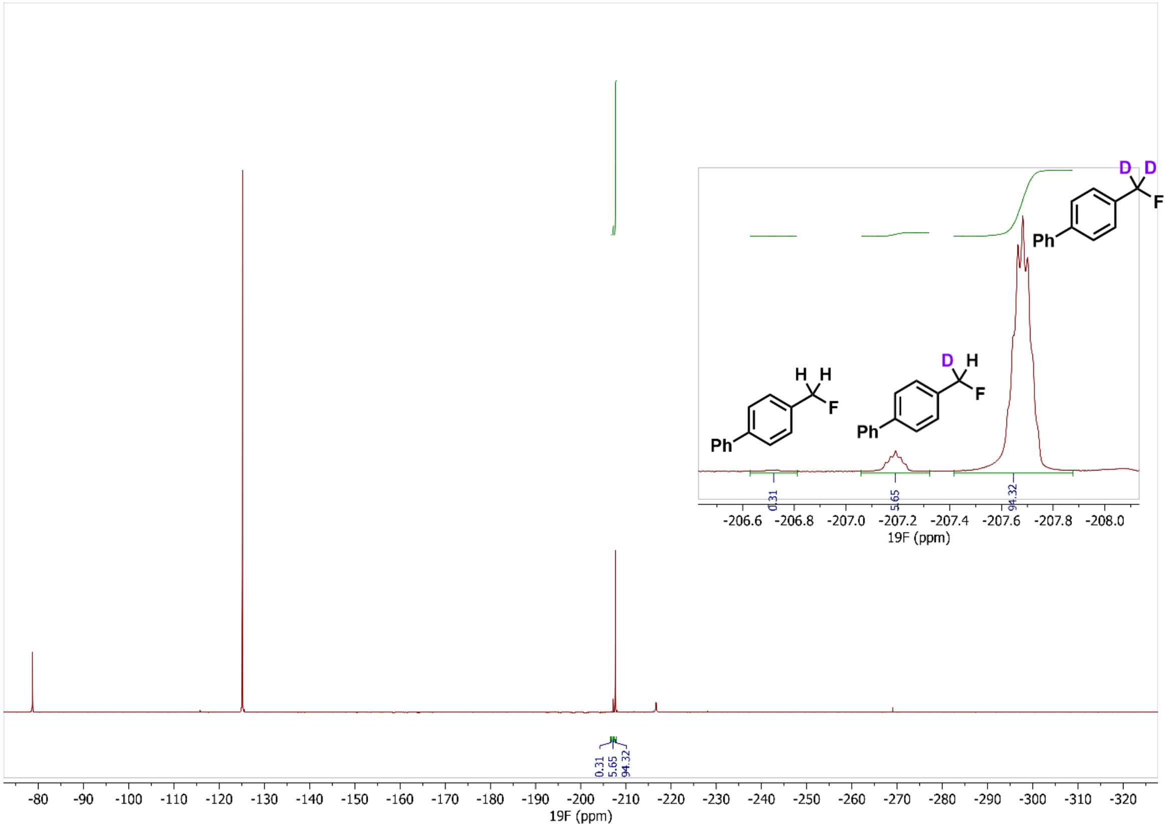

Figure S9. Quantitative ^{19}F{^{1}H} NMR of cross-coupling with [D_2]**3**.

Deuterium incorporation: (5.65 × 0.5) + 94.32 = 97.15%

Carrier-added Reaction

Radio-HPLC could not be used for determination of the deuterium content of the radiolabeled products because of the identical retention times of [^{18}F,D_2]**7**, [^{18}F,D]**7**, and [^{18}F]**7**. Therefore, a carrier-added experiment was performed, based on the assumption that the deuterium incorporation of [^{18}F]**3** is the same as that of [D_2]**3**. After decay, the reaction mixture was analyzed by quantitative ^{19}F{^{1}H} NMR.

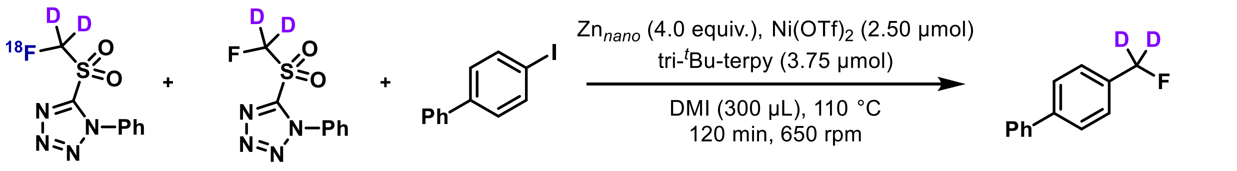

To a vial containing 4-iodo-1,1'-biphenyl (14.0 mg, 0.05 mmol), Ni(OTf)$_2$, (0.9 mg, 2.50 μmol), tri-^{t}Bu-terpy (1.5 mg, 3.75 μmol), zinc nanopowder (13.1 mg, 0.20 mmol) and 5-((fluoromethyl-d_2)sulfonyl)-1-phenyl-1*H*-tetrazole [D_2]**3** (12.2 mg, 0.05 mmol) in DMI (300 μL) was added [^{18}F,D_2]**3** (5–30 MBq in DMI (20–50 μL)). The mixture was heated to 110 °C whilst stirring at 650 rpm for 120 min and then cooled to room temperature and quenched by addition of a MeCN-d_3 (600 μL). This was left to decay, filtered through a filter vial and analyzed by quantitative ^{19}F{^{1}H} NMR (Figure S10).

***Note**: an increased reaction duration of 120 minutes was used to maximise conversion in presence of ^{19}F-carrier, to form sufficient quantities of [D_2]**3** for NMR analysis.*

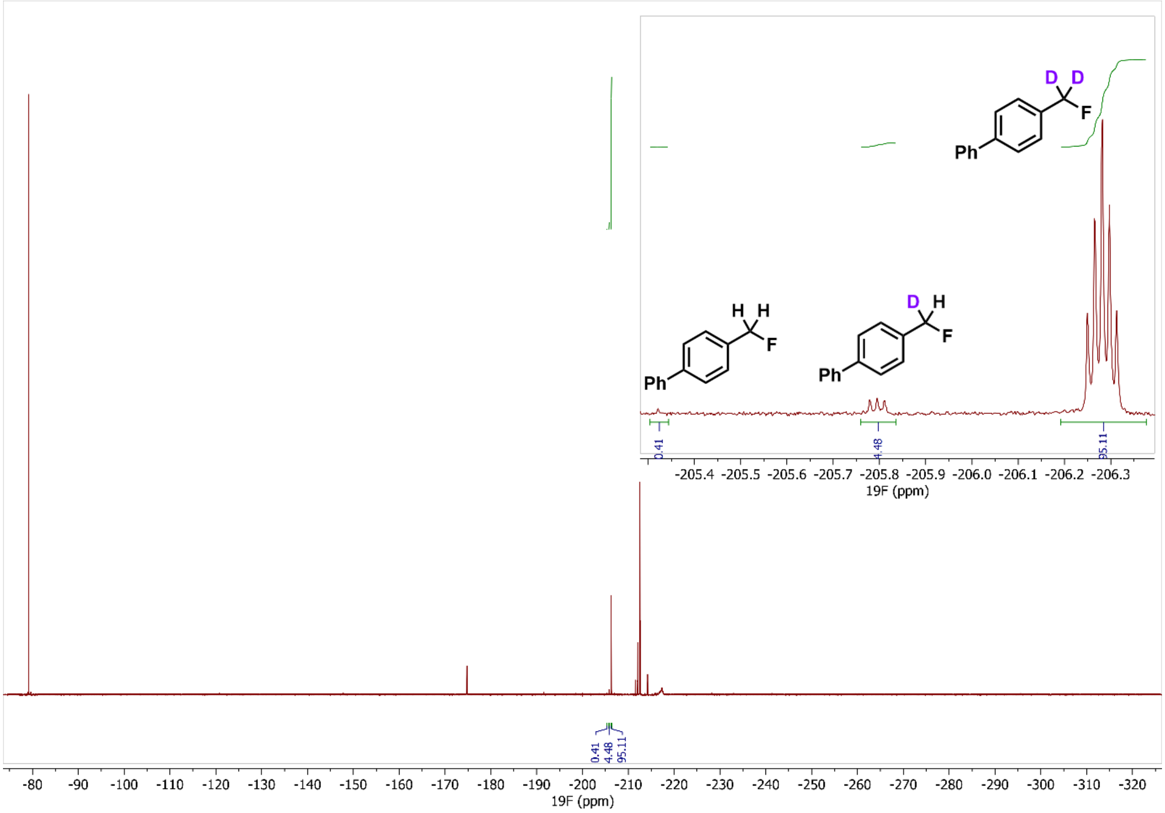

Figure S10. Quantitative $^{19}F\{^{1}H\}$ NMR of carrier added experiment.

Deuterium incorporation: (4.48 × 0.5) + 95.11 = 97.35%

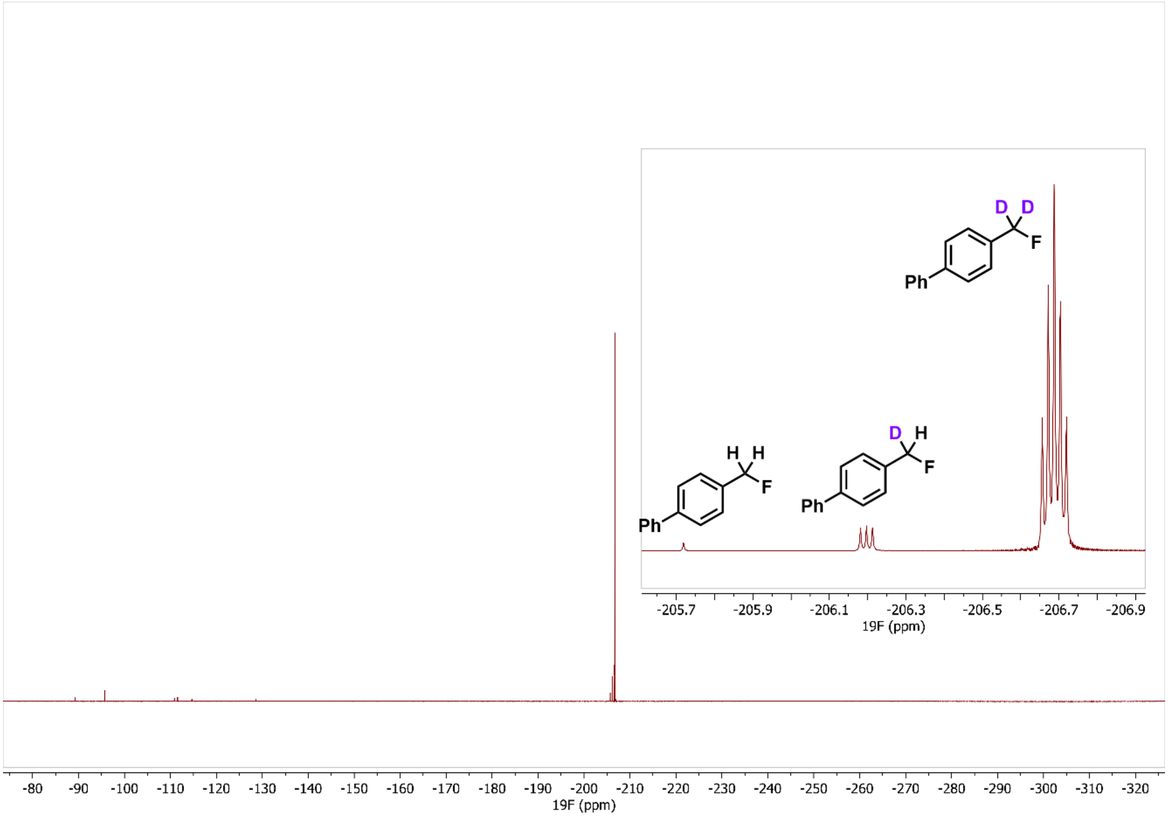

Figure S11. $^{19}F\{^{1}H\}$ NMR of authentic reference of [D_2]**7.**

Substrate Scope Limitations

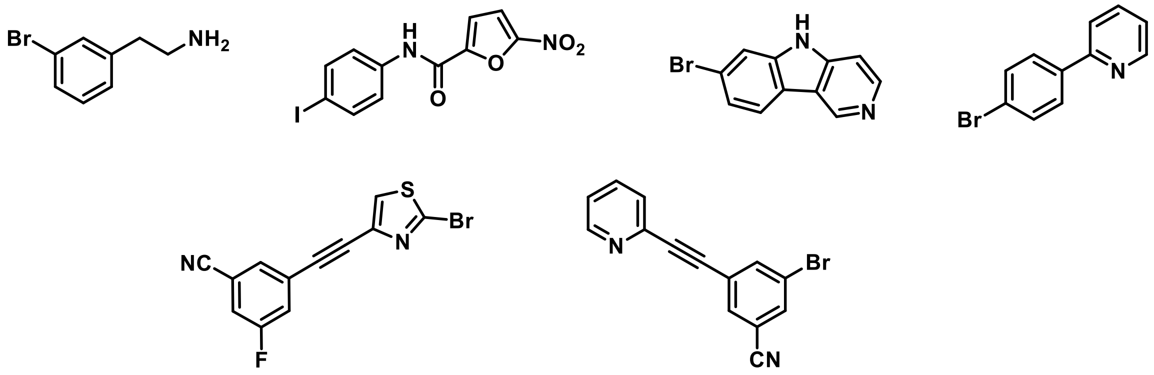

Figure S12. Unsuccessful substrates.

Various functional groups are known to be challenging in cross-electrophile couplings, e.g. internal alkynes and nitro groups.[57] As expected, substrates bearing these functional groups were not successful in this protocol. Furthermore, nitrogen-containing groups, such as pyridine and alkyl amines, were not well tolerated. This is likely due to the unproductive coordination of the nitrogen functionality to nickel, hindering the desired cross-coupling reaction.[58,59]

Semi-Automated Radiosynthesis of [^{18}F(D$_2$)]**39**

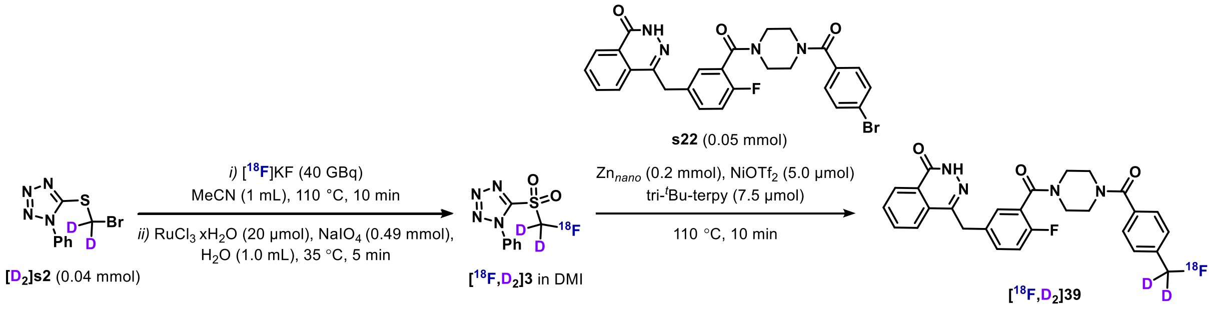

The radiosynthesis of [^{18}F,D$_2$]**39** was carried out using a Trasis AllinOne automated radiosynthesis platform, using a custom cassette set-up (Figure S13). A vial charged with K_2CO_3 (1.5 mg), Kryptofix® 222 (7.5 mg), MeCN (0.6 mL) and H_2O (0.15 mL) was inserted at position 2. A vial charged with 5-((bromomethyl-*d*$_2$)thio)-1-phenyl-1*H*-tetrazole [D$_2$]**s2** (10.9 mg, 0.04 mmol) in MeCN (1.0 mL) was inserted at position 9. A vial charged with $RuCl_3$ hydrate (4 mg), $NaIO_4$ (104 mg) and H_2O (1 mL) was inserted at position 10. Vials filled with dry MeCN (approximately 10 mL) were placed at positions 8 and 30. A vial was filled with dry DMI (approximately 5 mL) and placed at position 17. A water bag was placed in position 14. HPLC collection vials filled with H_2O (20 mL) were placed in positions 29 and 34. A Waters Sep-Pak AccellPlus QMA Carbonate Plus Light Cartridge was pre-conditioned with H_2O (10 mL) prior to use and placed in position 5. Two Waters Sep-Pak C18 Plus Short cartridges was activated with EtOH (10 mL) and then H_2O (10 mL) prior to use and was placed in positions 28 and 33.

A 1 mL V-vial equipped with a spin vane was charged with 4-(3-(4-(4-bromobenzoyl)piperazine-1-carbonyl)-4-fluorobenzyl)phthalazin-1(2*H*)-one (27.5 mg, 0.05 mmol), zinc nanoparticles (13.1 mg, 0.20 mmol), Ni(OTf)$_2$ (1.8 mg, 5.0 µmol) and tri-^{t}Bu-terpy (3.0 mg, 7.5 mmol) and fitted with a cap and vent needle. The reaction mixture was placed inside the hot cell in a lead pot in front of the TRASIS, connected to line 31 with a needle. An external hotplate inside the same hot cell was set to 110 °C and 650 rpm (Figure S14).

[^{18}F]Fluoride (40 GBq) in [^{18}O]water was received directly from the cyclotron, into the Trasis AllInOne (position 6). The [^{18}F]fluoride was separated from the water by trapping on the QMA cartridge (position 5), followed by elution with the solution from the vial in position 2 into the reactor. The [^{18}F]fluoride was then dried azeotropically with addition of MeCN, and once this was complete, the ^{18}F-fluorination of the precursor ([D$_2$]**s2**) proceeded in the same reactor at 110 °C for 10 minutes. The reactor was then cooled and the contents of the vial at position 10 were added to the same reactor. The oxidation reaction proceeded at 35 °C for 5 minutes. The crude reaction mixture was diluted with H_2O and MeCN, and transferred to the HPLC injection loop for semi-preparative reverse phase HPLC purification (conditions G, 45:55 MeCN:H_2O). The desired purified product was collected in the collection vial at position 34. This solution was diluted further with H_2O for reformulation using a C18 Plus cartridge. The cartridge was purged with air (10 mL) and flushed with DMI (0.5 mL) that was sent to the waste container. Then, [^{18}F,D$_2$]**3** was eluted from the C18 Plus cartridge with DMI (0.4 mL) and added to the reaction mixture *via* line 31. Once elution was complete, the line to position 31 and the vent needle were manually removed from the reaction vial using the telemanipulators, and the activity in the vial was measured in a dose calibrator. The vial was then moved using the telemanipulators onto the hotplate inside the hot cell

and the reaction mixture was stirred whilst heating at 110 °C for 15 minutes. The vial was moved using the telemanipulators to a lead pot inside the hot cell to cool for 5 minutes. A vent needle was added to the reaction mixture using the telemanipulators, and further purification of the reaction mixture was carried out following either method A or method B.

Method A: Inside the hot cell, the reaction mixture was taken up into a syringe using telemanipulators containing H_2O (4 mL). This liquid was passed through a Waters Sep-Pak C18 Plus Short cartridge (pre-activated with EtOH (10 mL) and H_2O (10 mL)). The cartridge was then purged with air (3 mL) and eluted with MeCN (1.5 mL) into an empty V-vial equipped with a vent needle. The needle from line 13 was then placed into the V-vial containing the eluate. The eluate was taken up *via* the 3 mL syringe at position 11, diluted with H_2O and MeCN and transferred to the HPLC injection loop for semi-preparative reverse phase HPLC purification (conditions H, 35:65 MeCN:H_2O). The desired purified product was collected in the collection vial at position 29. This solution was diluted further with H_2O for reformulation using a C18 Plus cartridge. [^{18}F]**39** was then eluted with MeCN (2.0 mL) into a vial *via* line 26. The radiosynthesis data is summarised below (Table S19).

***Note**: high backpressure was observed for manual filtration of the reaction mixture due to poor solubility through a C18 cartridge, hence an alternative method (method B) was also attempted.*

Method B: The reaction mixture was manually taken up into a syringe containing MeCN (2 mL). This syringe was moved onto a Waters Sep-Pak C18 Plus Short cartridge (pre-activated with EtOH (10 mL) and H_2O (10 mL)) and eluted into an empty V-vial equipped with a vent needle. The cartridge was then purged with air (3 mL) and the needle from line 13 was then placed into the V-vial containing the eluate. The eluate was taken up *via* the 3 mL syringe at position 11, diluted with H_2O and MeCN and transferred to the HPLC injection loop for semi-preparative reverse phase HPLC purification (conditions H, 32:68 MeCN:H_2O). The desired purified product was collected in the vial at position 29. The radiosynthesis data is summarised below (Table S19).

Table S20. Radiosynthesis of [^{18}F,D_2]**39**. RCP determined by integration of [^{18}F,D_2]**39** in radio-HPLC relative to all radioactive species.

Starting activity of [^{18}F,D_2]3 (GBq)	Purification method	AY (MBq, n.d.c.)	RCP (%)	Total synthesis time (min)	A_m (GBq µmol^{-1})
2.91	**A**	260	>99	145	64
2.10	**B**	174	>99	139	78

***Note:** all manipulations described in this procedure are carried out inside a 75 mm Pb-shielded hot cell that remains sealed at all times, using either a TRASIS AiO or externally mounted telepliers to minimize exposure to ionizing radiation (Figure S14).*

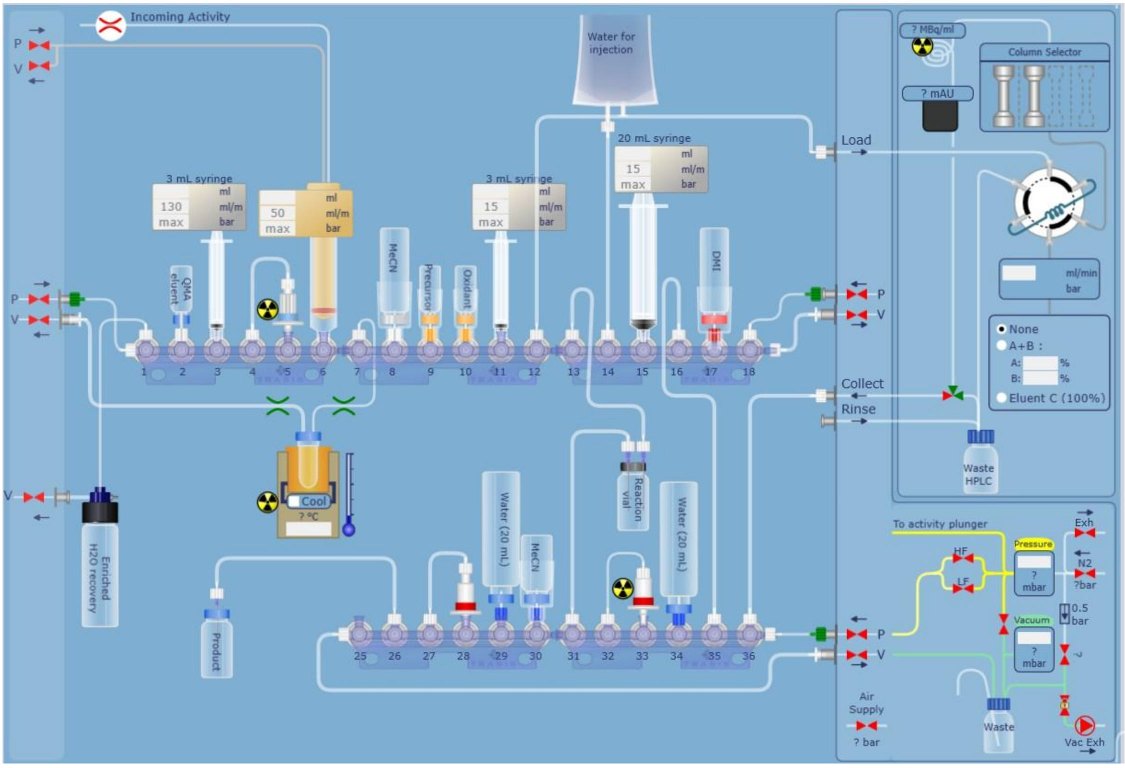

Figure S13. Cassette layout for the semi-automated synthesis of [^{18}F]**39** on the Trasis AllInOne platform.

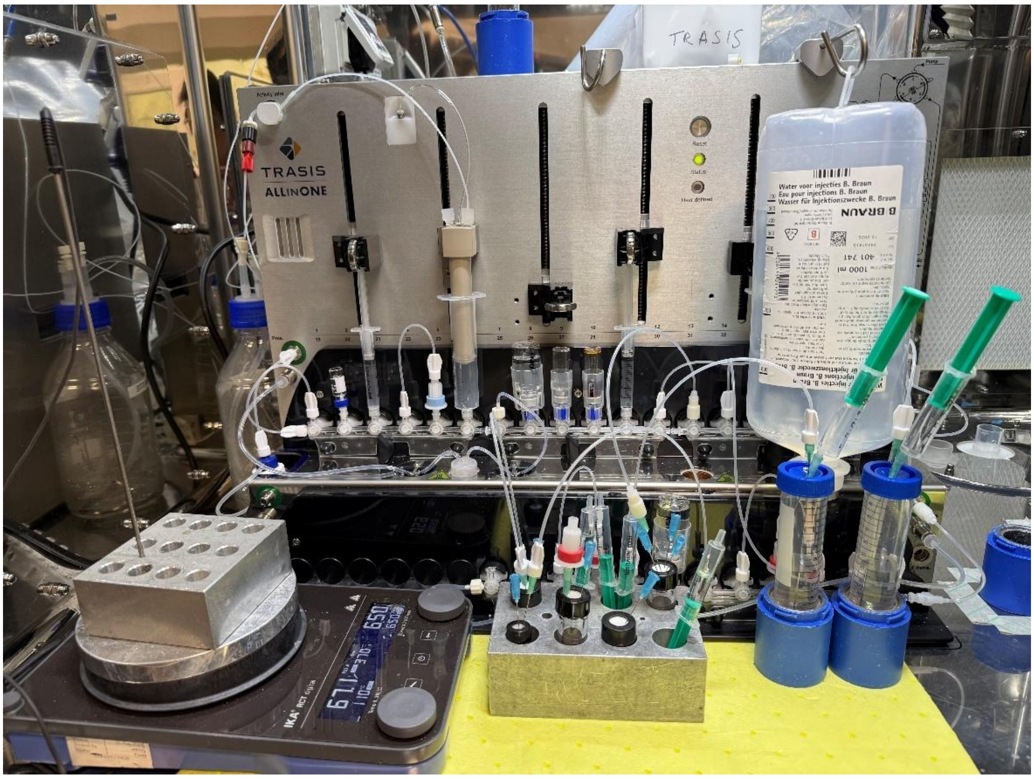

Figure S14. Trasis AllInOne set-up with external hotplate for semi-automated radiosynthesis.

Calculation of Molar Activity of [^{18}F,D_2]39

The molar activity of [^{18}F,D_2]**39** was measured on two samples, and each sample was injected multiple times. For each injection, the UV response corresponding to [^{18}F,D_2]**39** was integrated, and converted to an amount in µmol using a calibration curve (Figure S15). The amount of activity injected (in MBq) and the time of injection were also recorded, and the amount of activity injected was decay-corrected back to the end of synthesis.

Procedure for the calibration curve of **39**: A calibration curve for authentic reference **39** was recorded by preparing samples of a range of concentrations by serial dilution, starting with a solution of **39** (2.7 mg) in MeCN (1.0 mL). These were injected onto an HPLC (10 µL injection volume from a 1.0 mL stock, HPLC conditions F) and the UV response was measured by integrating the peak of interest.

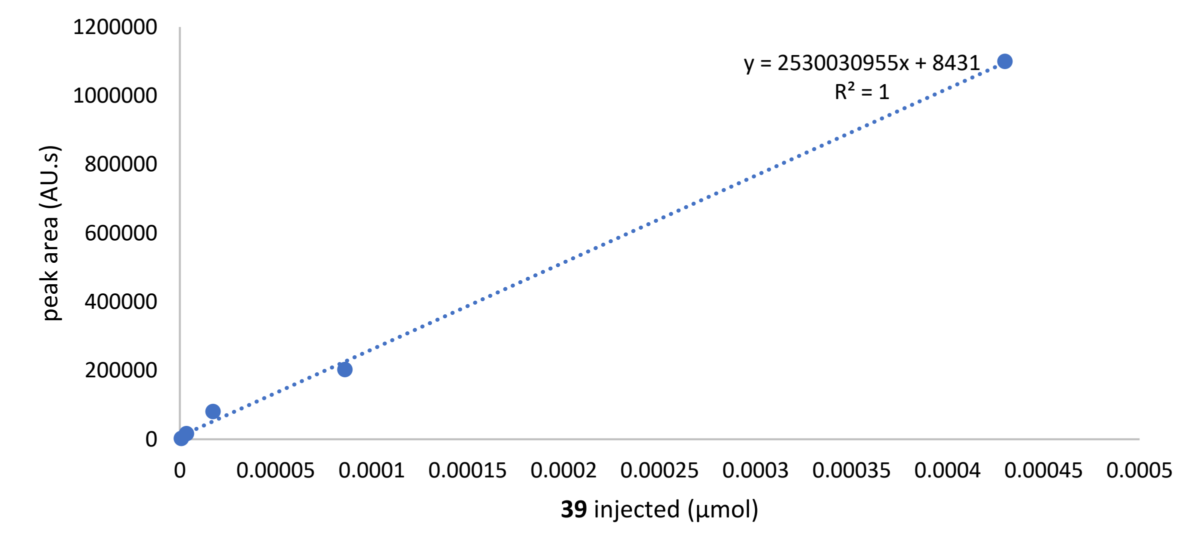

Figure S15. HPLC calibration curve for authentic reference **39**.

Table S21. Molar activity calculation of [^{18}F,D_2]**39**, using purification method A.

Measurement	Activity injected (MBq, d.c. EOS)	Peak area (mAU.s)	39 injected (µmol)	A_m (GBq µmol^{-1})
**1**	0.414	16059	6.35 x 10^{-6}	65.2
**2**	0.372	15191	6.00 x 10^{-6}	62.0
			**Average:** 64 ± 2 GBq µmol^{-1}	

Table S22. Molar activity calculation of [^{18}F,D$_2$]**39**, using purification method B.

Measurement	Activity injected (MBq, d.c. EOS)	Peak area (AU.s)	39 injected (µmol)	A_m (GBq µmol^{-1})
**1**	0.528	21298	8.42×10^{-6}	62.7
**2**	0.672	21017	8.31×10^{-6}	80.9
**3**	0.772	21368	8.45×10^{-6}	91.4

Average: 78 ± 12 GBq µmol^{-1}

Overall average: 71 ± 7 GBq µmol^{-1}$_{(n=2)}$

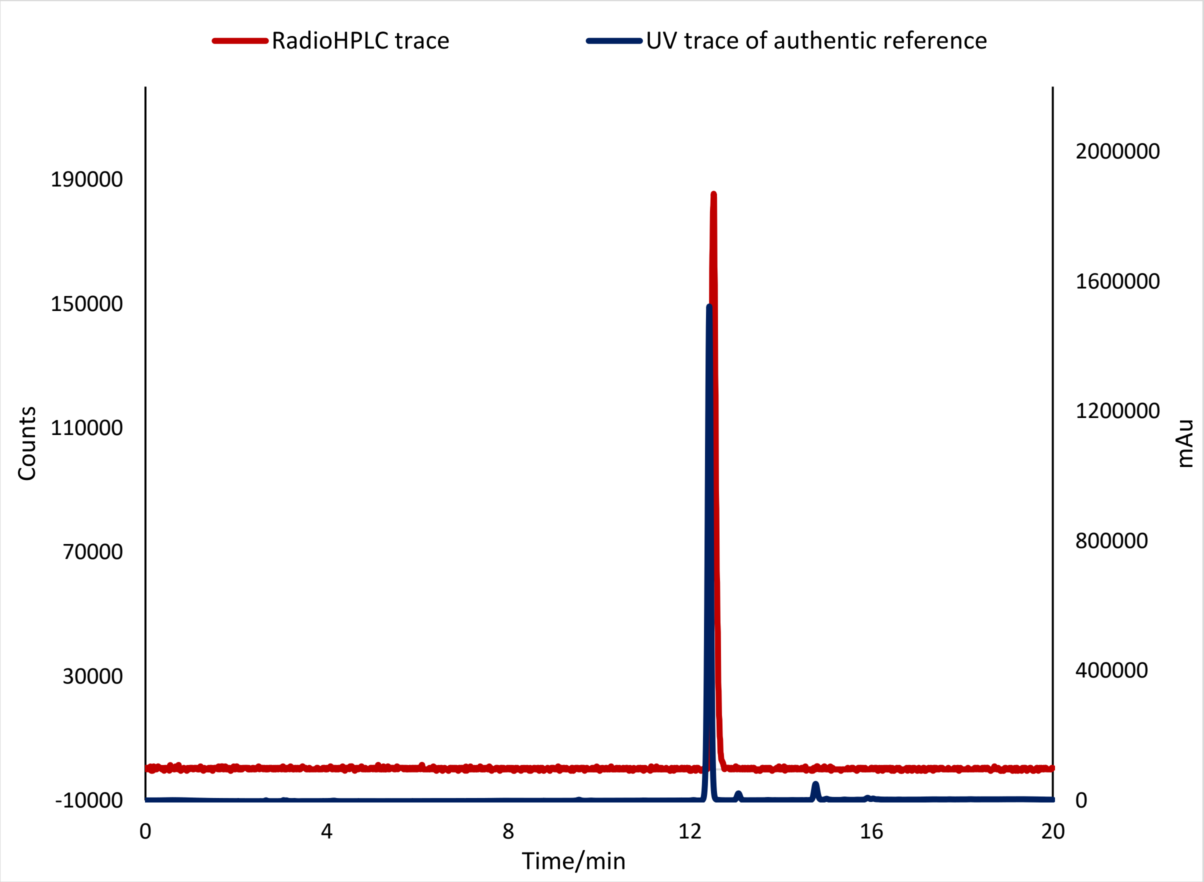

Figure S16. Radio and UV-HPLC trace overlays for isolated [^{18}F,D$_2$]**39**.

Determination of Residual Nickel Content in Isolated [^{18}F,D_2]39

HPLC-purified [^{18}F,D_2]**39** was left to decay, diluted with H_2O (1 mL) and then analyzed by Quantofix® semi-quantitative test strips for determination of residual Ni^{2+} content. No colour change was detected, indicative of a residual Ni^{2+} content of less than 10 mg/L (Figure S17).

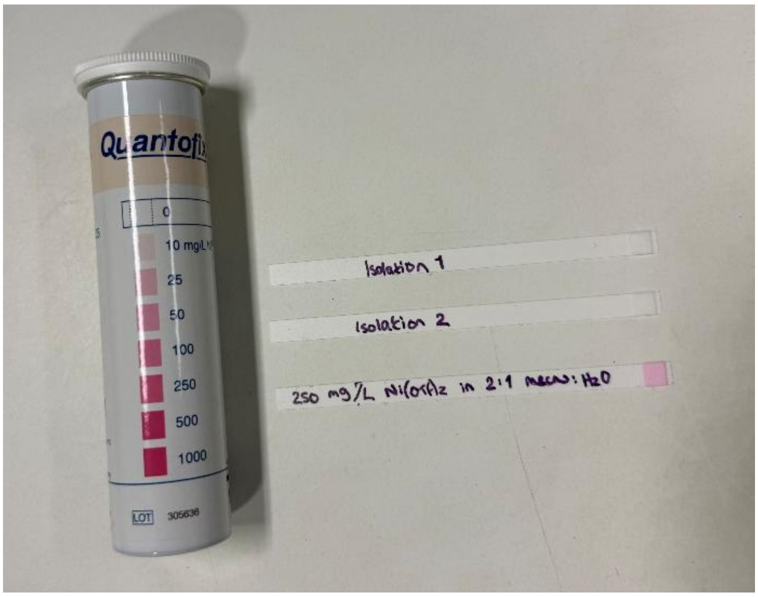

Figure S17. Quantofix® test strips for determination of residual nickel content. Sampled from final product vial of isolated [^{18}F,D_2]**39** in MeCN/H_2O after decay (top two strips), compared to 250 mg/L $Ni(OTf)_2$ stock solution in MeCN/H_2O (bottom strip).

Radio and UV HPLC Trace Overlays

[^{18}F]5-((Fluoromethyl)sulfonyl)-1-phenyl-1*H*-tetrazole ([^{18}F]3)

Prepared following the automated radiosynthesis of [^{18}F]**3** and analyzed by (radio)HPLC using conditions A or C-E.

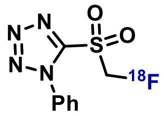

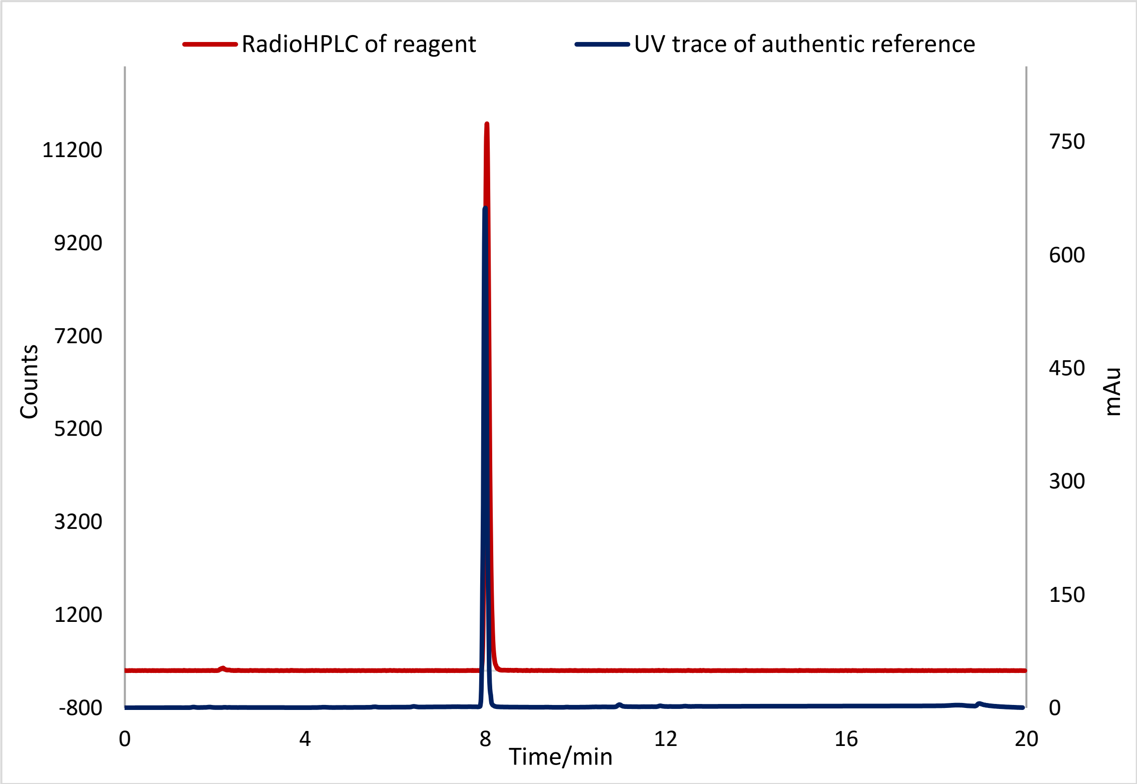

2-((Fluoromethyl)sulfonyl)benzo[*d*]thiazole ([^{18}F]4)

Prepared following the automated radiosynthesis of [^{18}F]**4** and analyzed by (radio)HPLC using conditions D.

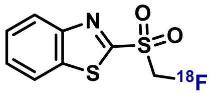

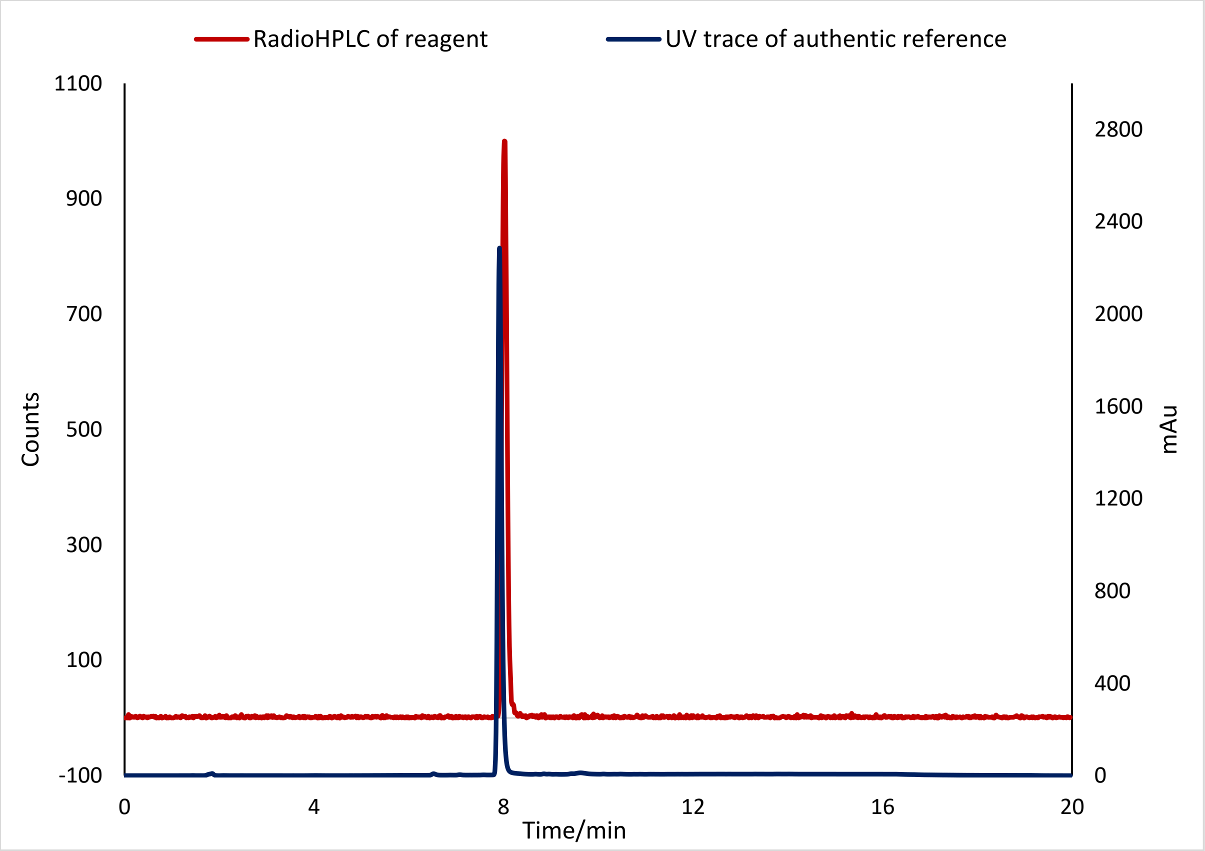

[^{18}F]4-(Fluoromethyl)-1,1'-biphenyl ([^{18}F]7)

Prepared following the general procedure for the cross-coupling of [^{18}F]**3** and analyzed by (radio)HPLC using conditions A or E.

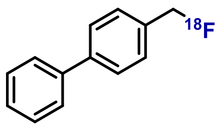

Run	RCC (%)
1	97
2	91
3	74
4	65
5	59
6	77
7	68
8	76
9	79
10	82
11	83
12	89
13	90
	**Average RCC:** 79 ± 11%*(n=13)*

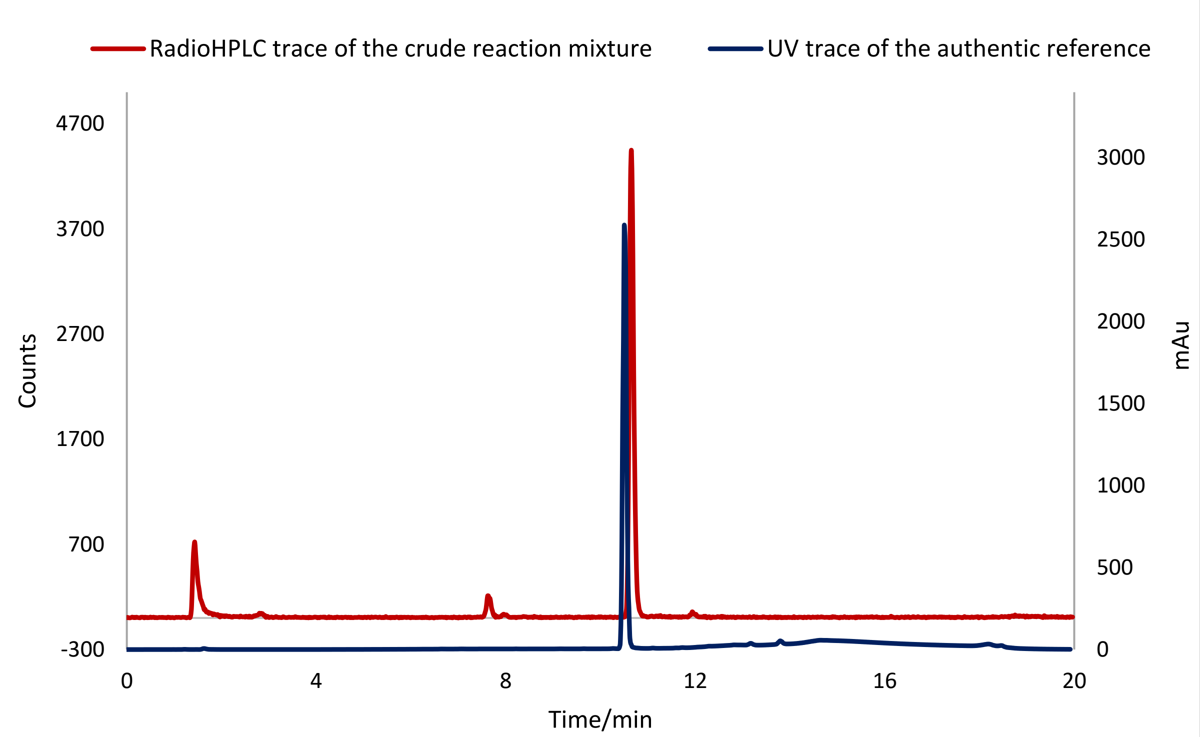

[^{18}F]Ethyl 4-(fluoromethyl)benzoate ([^{18}F]8)

Prepared following the general procedure for the cross-coupling of [^{18}F]**3** and analyzed by (radio)HPLC using conditions A or C.

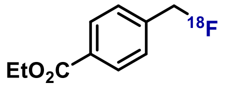

Run	RCC (%)
1	82
2	62
3	62
	**Average RCC:** 69 ± 9%$_{(n=3)}$

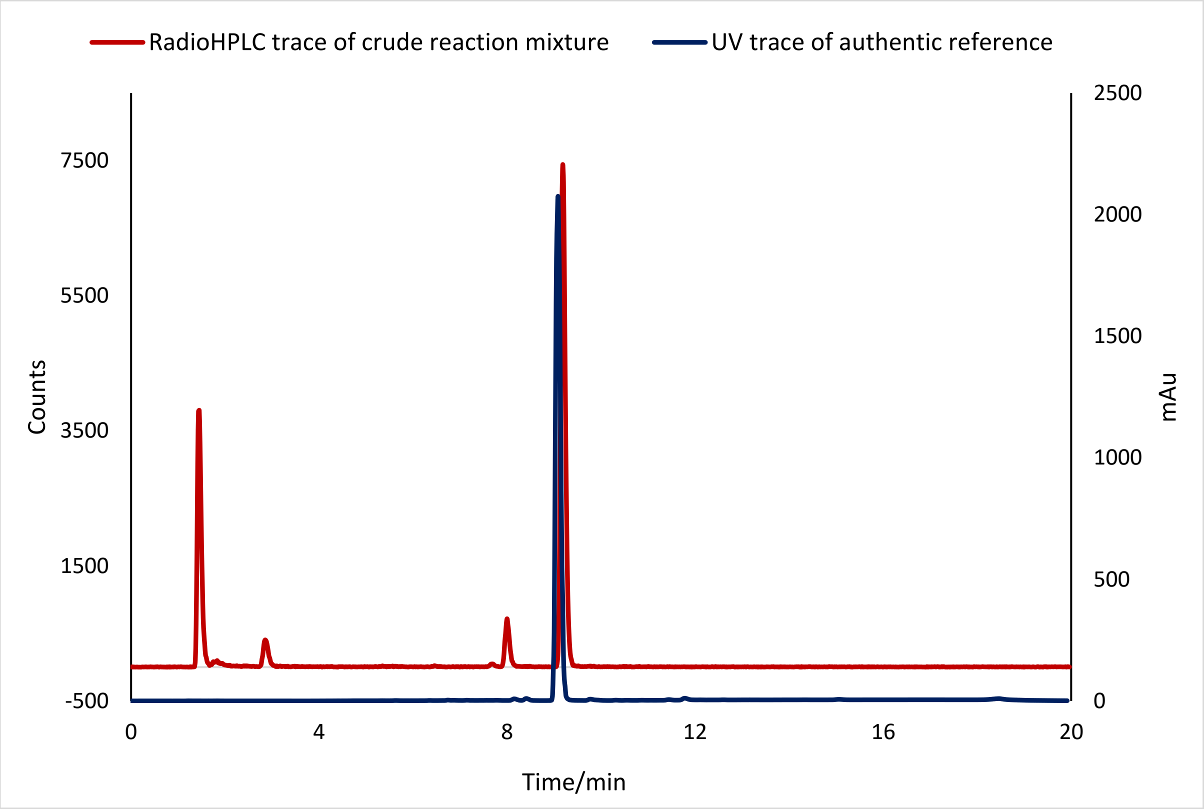

[^{18}F]4-(Fluoromethyl)benzonitrile ([^{18}F]9)

Prepared following the general procedure for the cross-coupling of [^{18}F]**3** and analyzed by (radio)HPLC using conditions A or D.

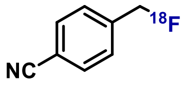

Run	RCC (%)
1	68
2	69
3	46
	**Average RCC:** 61 ± 11%$_{(n=3)}$

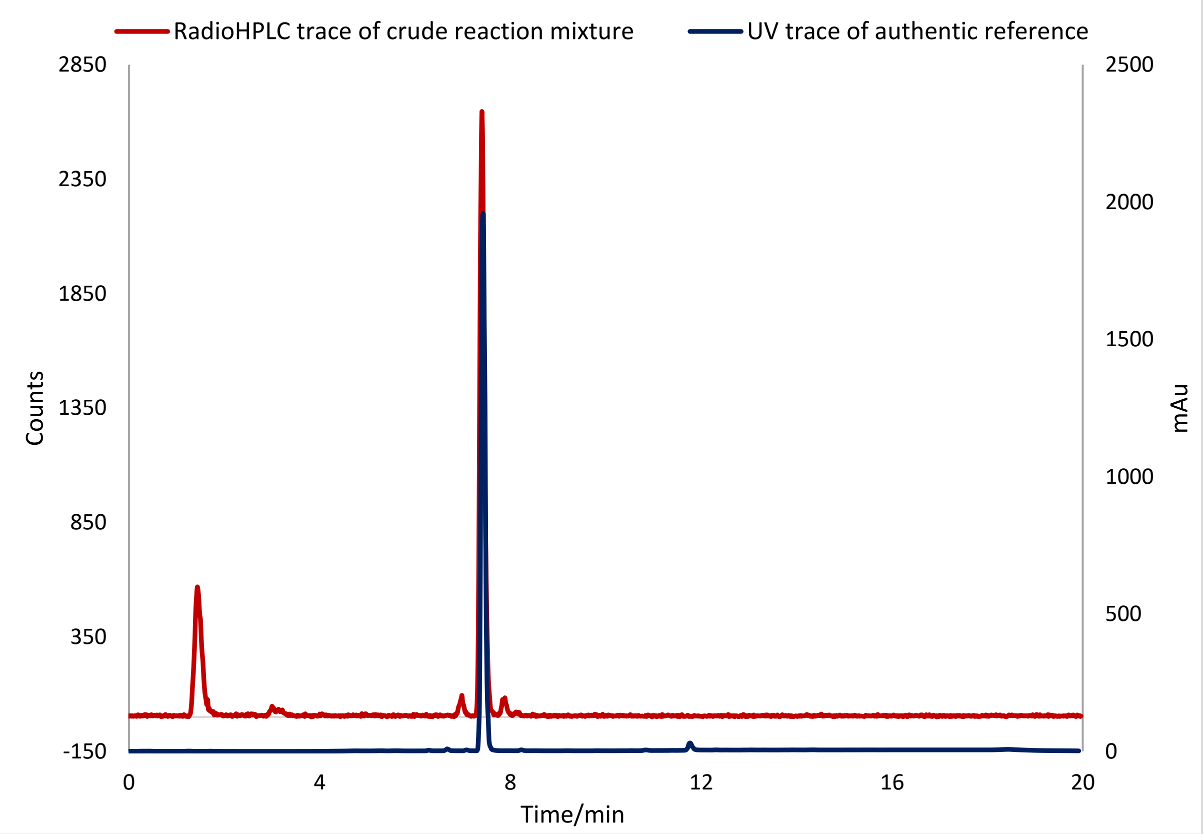

[^{18}F]1-(4-(Fluoromethyl)phenyl)ethan-1-one ([^{18}F]10)

Prepared following the general procedure for the cross-coupling of [^{18}F]**3** and analyzed by (radio)HPLC using conditions A or D.

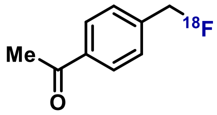

Run	RCC (%)
1	85
2	96
3	66
	**Average RCC:** 82 ± 12%$_{(n=3)}$

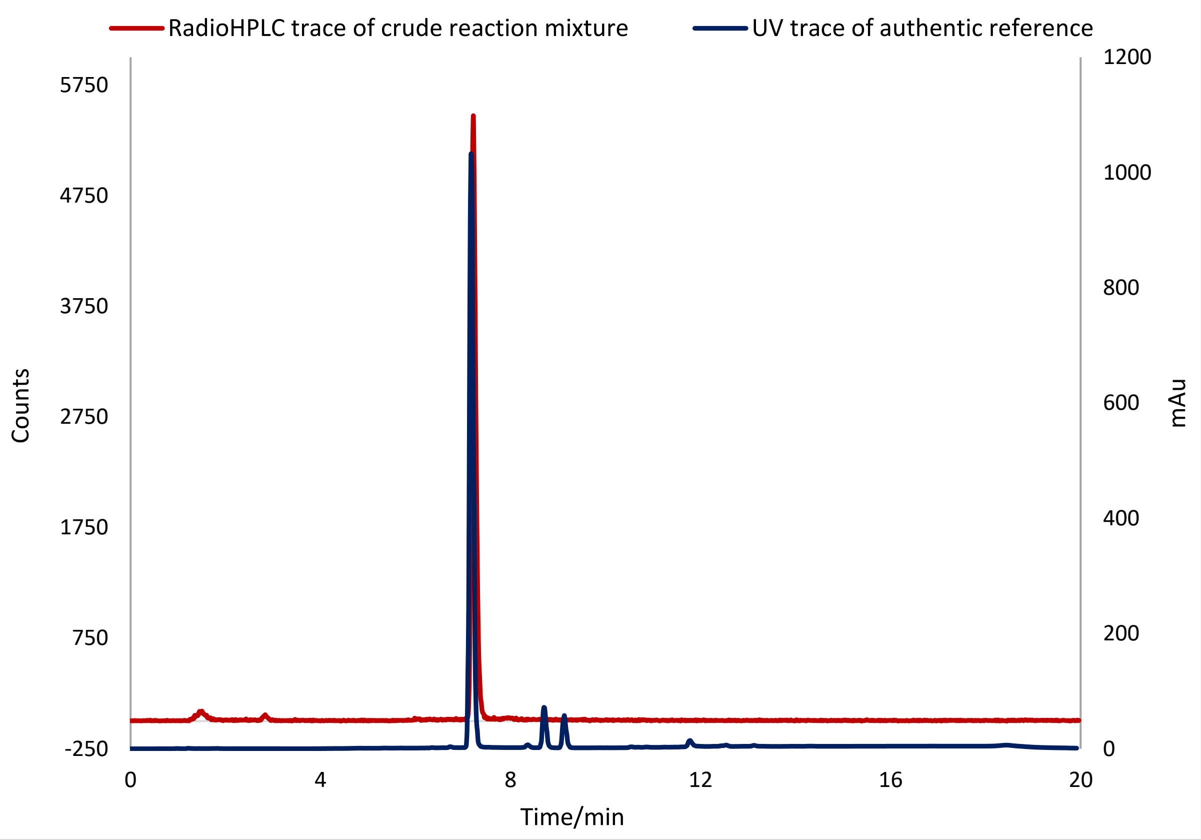

[^{18}F]4-(Fluoromethyl)-*N,N*-dimethylbenzamide ([^{18}F]11)

Prepared following the general procedure for the cross-coupling of [^{18}F]**3** and analyzed by (radio)HPLC using conditions A or C or D.

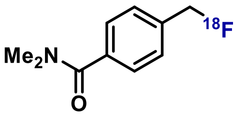

Run	RCC (%)
1	93
2	70
3	77
	**Average RCC:** 80 ± 10%$_{(n=3)}$

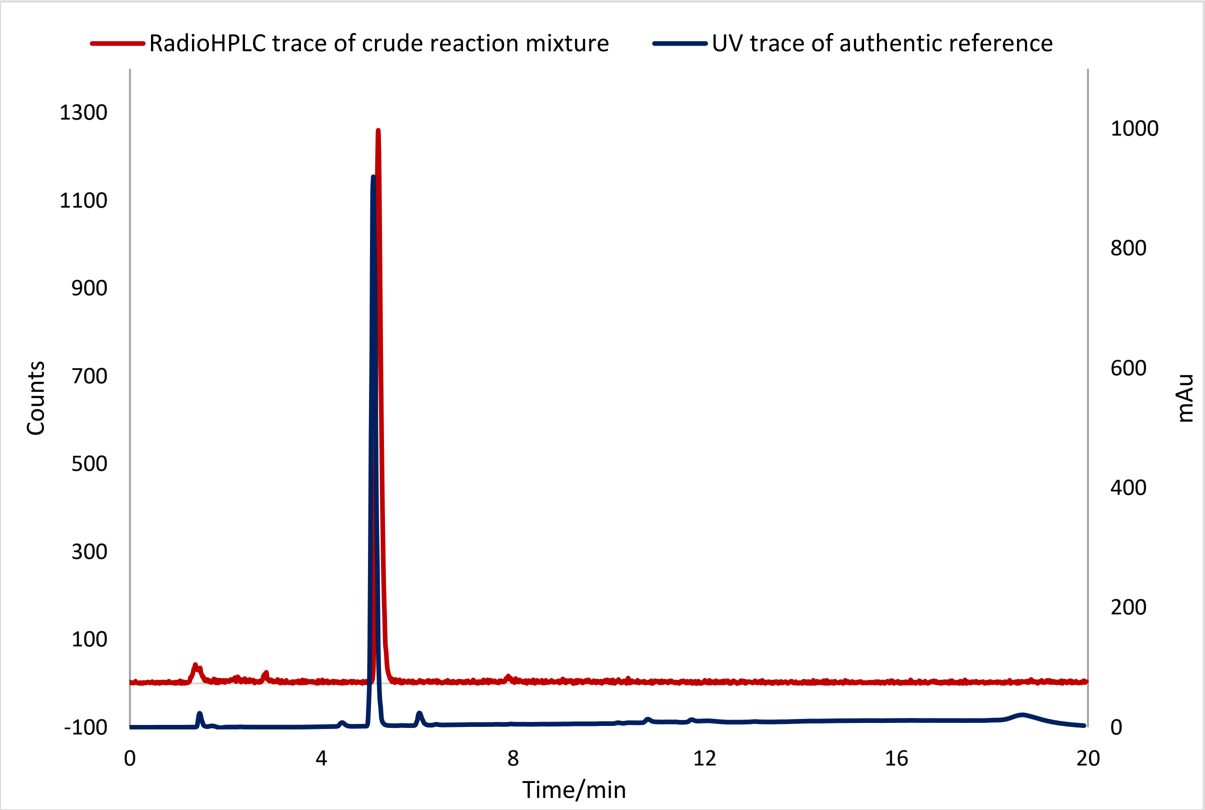

[^{18}F]1-(Fluoromethyl)-4-(trifluoromethyl)benzene ([^{18}F]12)

Prepared following the general procedure for the cross-coupling of [^{18}F]**3** and analyzed by (radio)HPLC using conditions D or E.

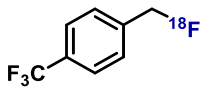

Run	RCC (%)
1	52
2	84
3	65
	**Average RCC:** 67 ± 13%$_{(n=3)}$

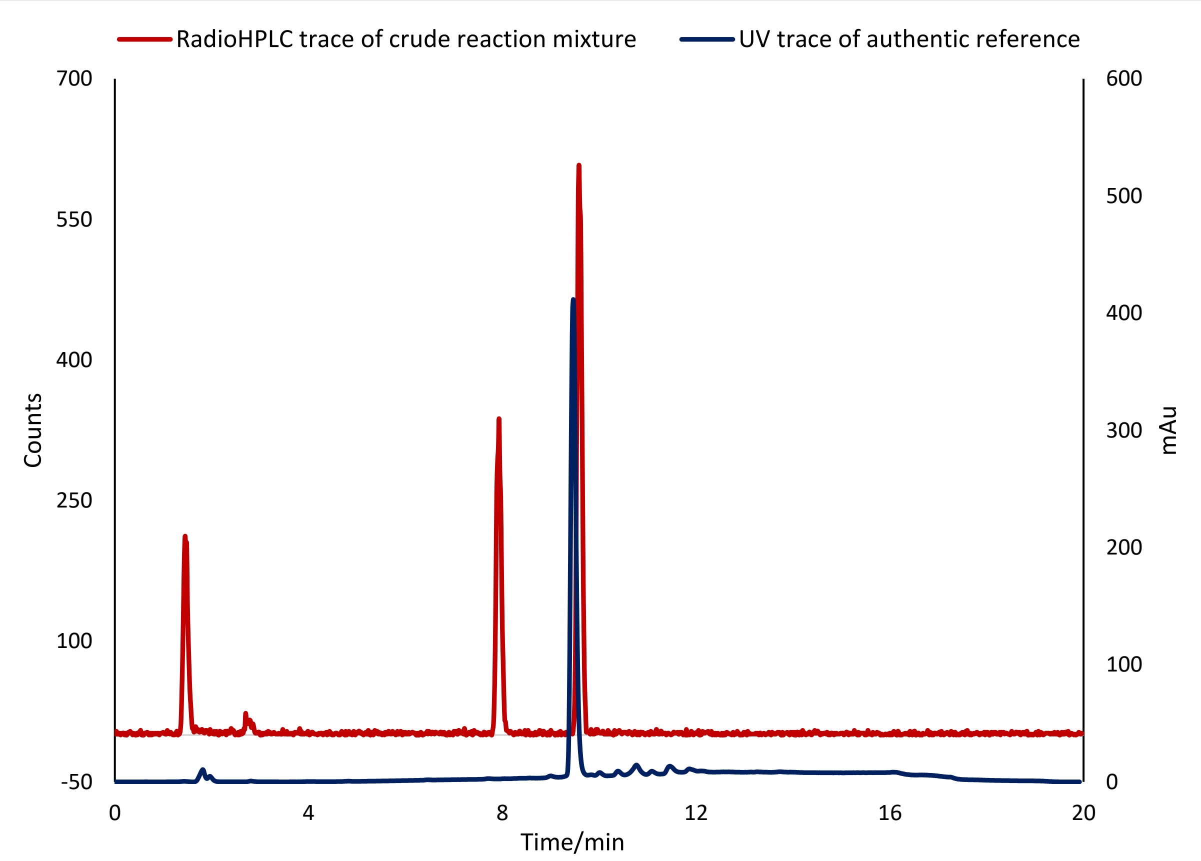

[^{18}F]1-(Fluoromethyl)-4-(methylsulfonyl)benzene ([^{18}F]13)

Prepared following the general procedure for the cross-coupling of [^{18}F]**3** and analyzed by (radio)HPLC using conditions D or E.

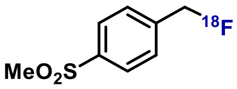

Run	RCC (%)
1	77
2	78
3	71
	**Average RCC:** 75 ± 3%$_{(n=3)}$

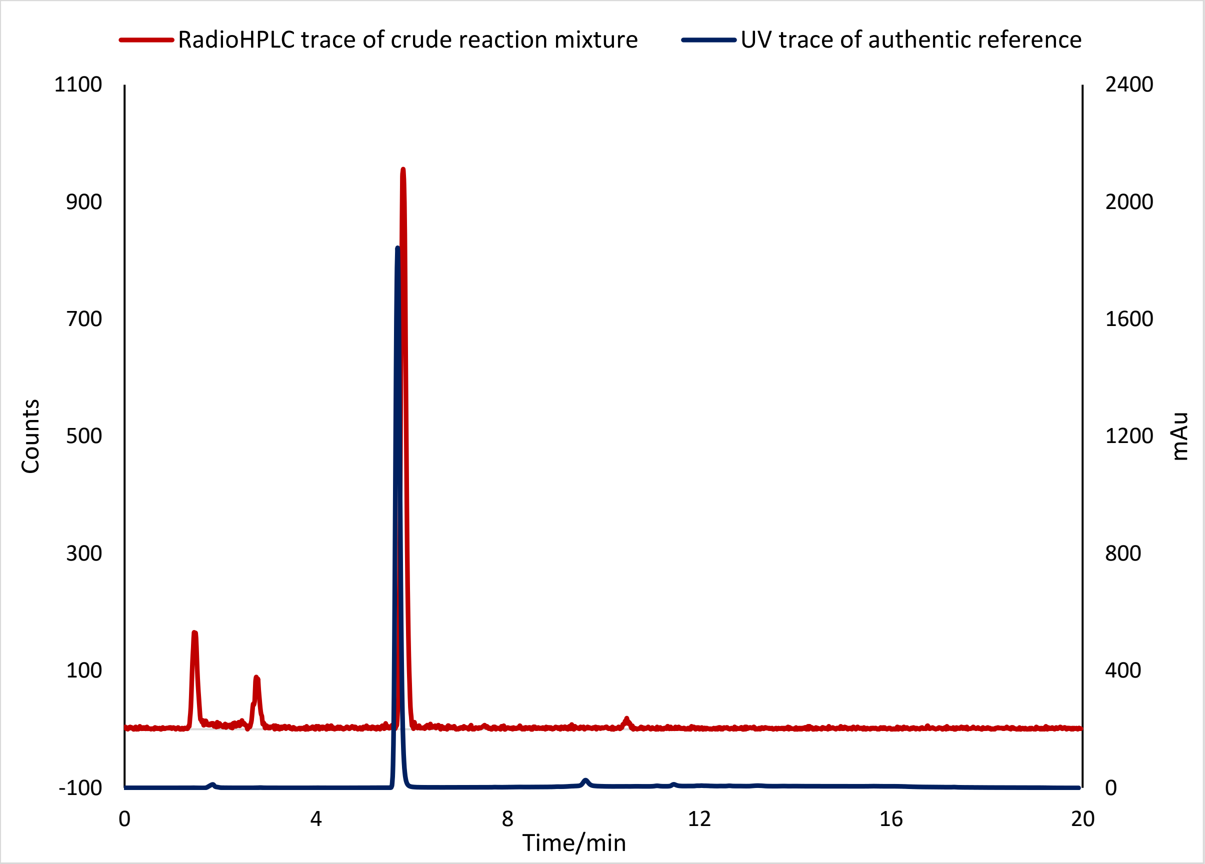

[^{18}F]2-(Fluoromethyl)-1,1'-biphenyl ([^{18}F]14)

Prepared following the general procedure for the cross-coupling of [^{18}F]**3** and analyzed by (radio)HPLC using conditions A or D.

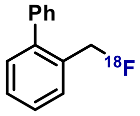

Run	RCC (%)
1	76
2	47
3	58
	**Average RCC:** 60 ± 12%$_{(n=3)}$

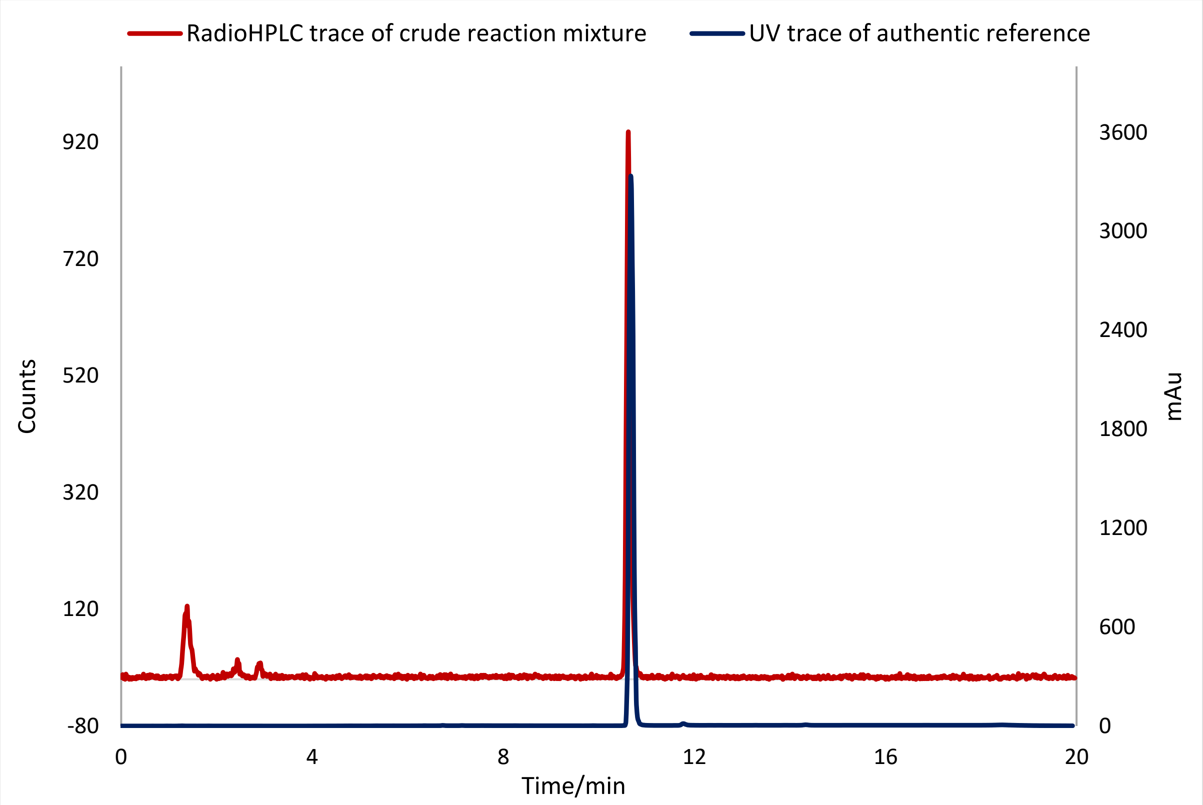

[18F]1-(Fluoromethyl)naphthalene ([18F]15)

Prepared following the general procedure for the cross-coupling of [18F]**1** and analyzed by (radio)HPLC using conditions D or E.

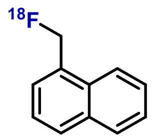

Run	RCC (%)
1	84
2	81
3	80
	**Average RCC:** 82 ± 2%*(n=3)*

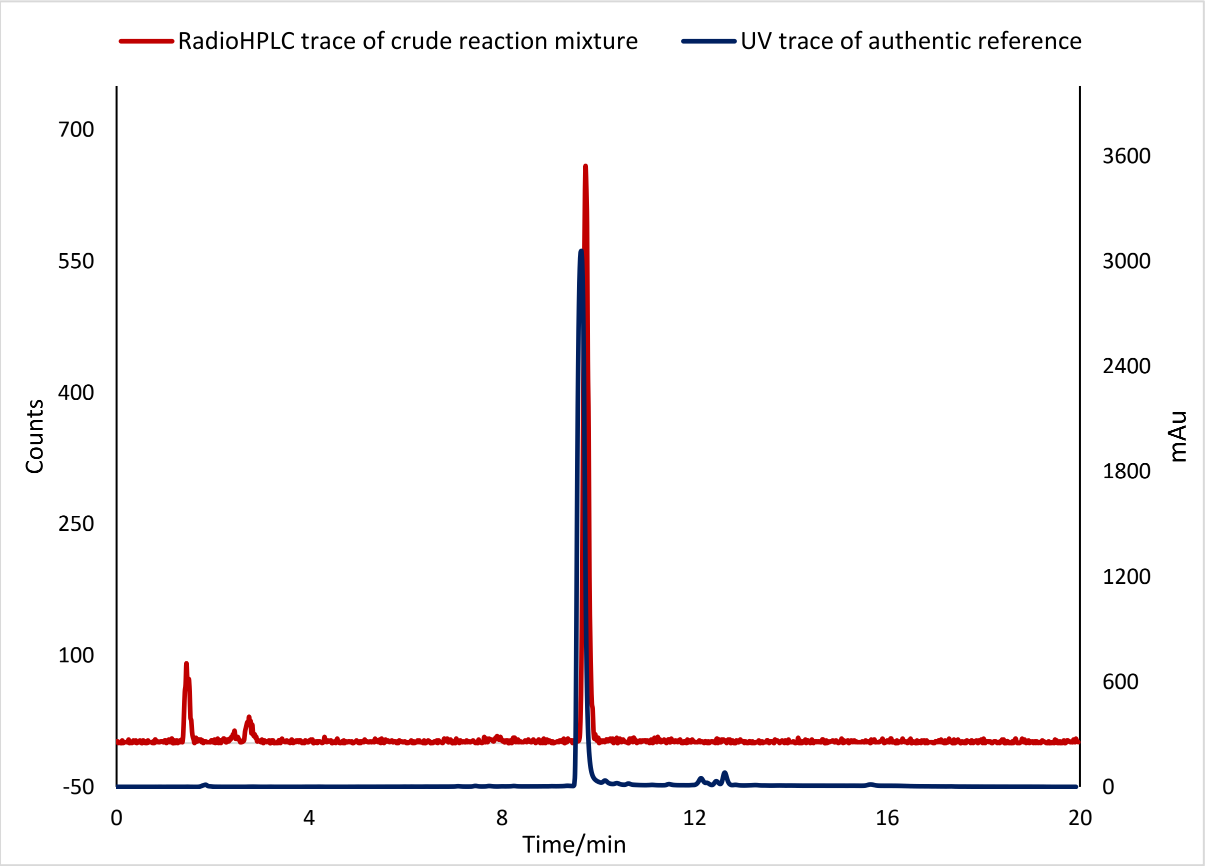

[^{18}F]2-(Fluoromethyl)naphthalene ([^{18}F]16)

Prepared following the general procedure for the cross-coupling of [^{18}F]**3** and analyzed by (radio)HPLC using conditions A or C.

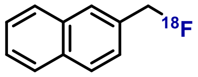

Run	RCC (%)
1	72
2	81
3	59
	**Average RCC:** 71 ± 9%$_{(n=3)}$

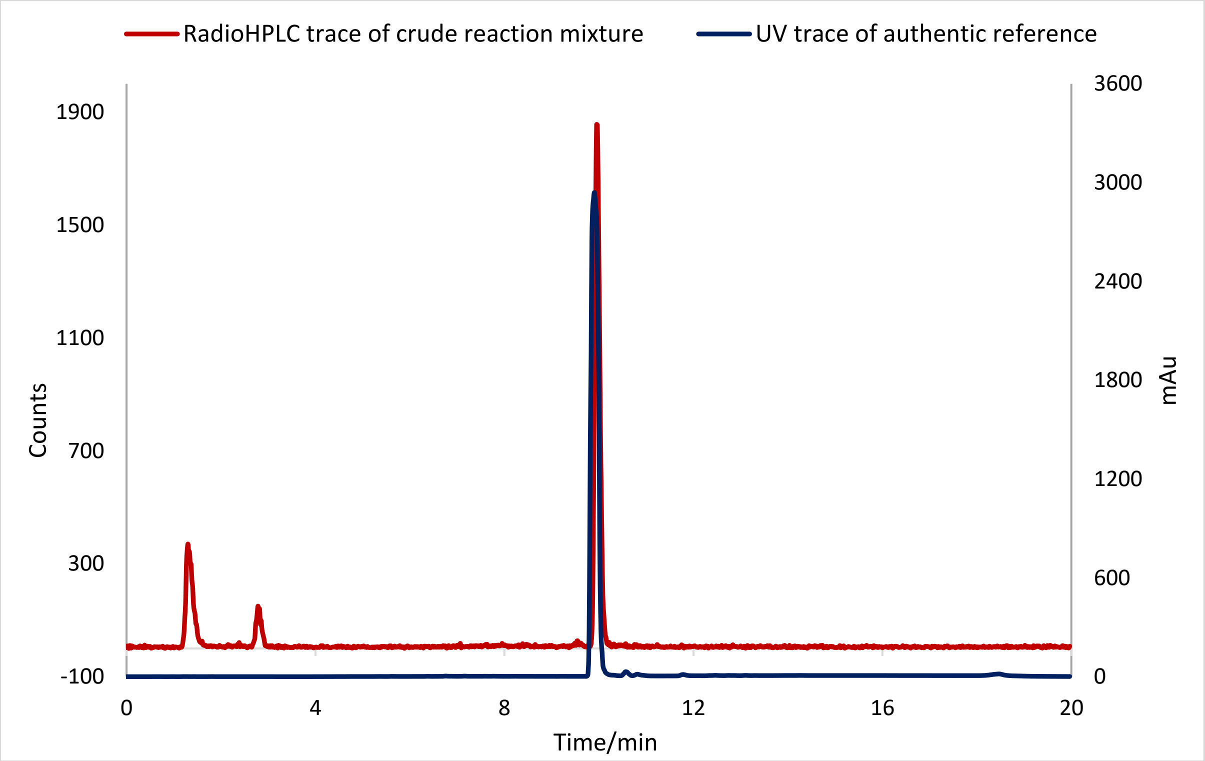

[^{18}F]Ethyl 3-(fluoromethyl)benzoate ([^{18}F]17)

Prepared following the general procedure for the cross-coupling of [^{18}F]**3** and analyzed by (radio)HPLC using conditions A or E.

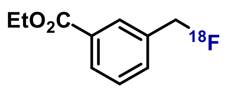

Run	RCC (%)
1	91
2	80
3	59
	**Average RCC:** 77 ± 13%$_{(n=3)}$

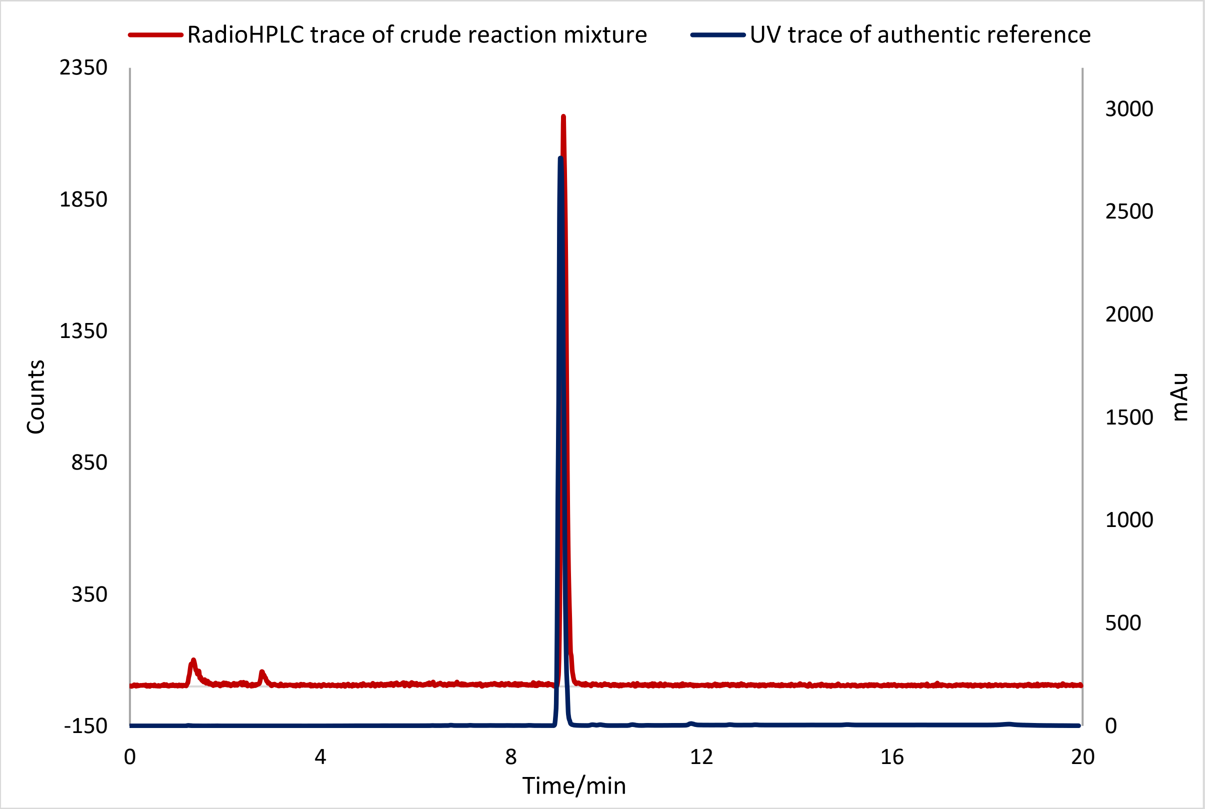

[^{18}F]1-(Fluoromethyl)-3-isopropoxybenzene ([^{18}F]18)

Prepared following the general procedure for the cross-coupling of [^{18}F]**3** and analyzed by (radio)HPLC using conditions C-E .

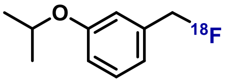

Run	RCC (%)
1	53
2	92
3	85
	**Average RCC:** 77 ± 17%$_{(n=3)}$

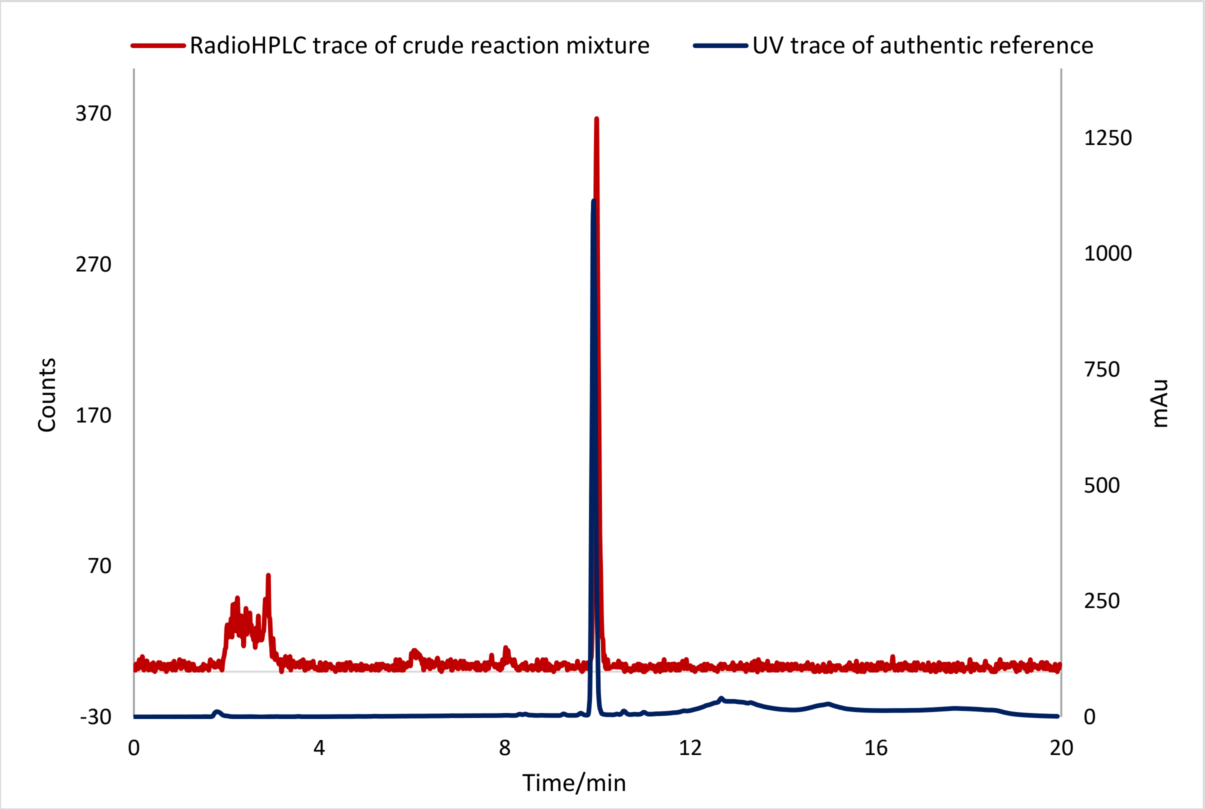

[^{18}F]4-(Fluoromethyl)phenethyl 4-methylbenzenesulfonate ([^{18}F]19)

Prepared following the general procedure for the cross-coupling of [^{18}F]**3** and analyzed by (radio)HPLC using conditions C.

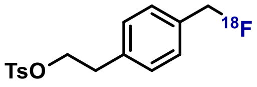

Run	RCC (%)
1	66
2	54
3	71
	**Average RCC:** 64 ± 7%$_{(n=3)}$

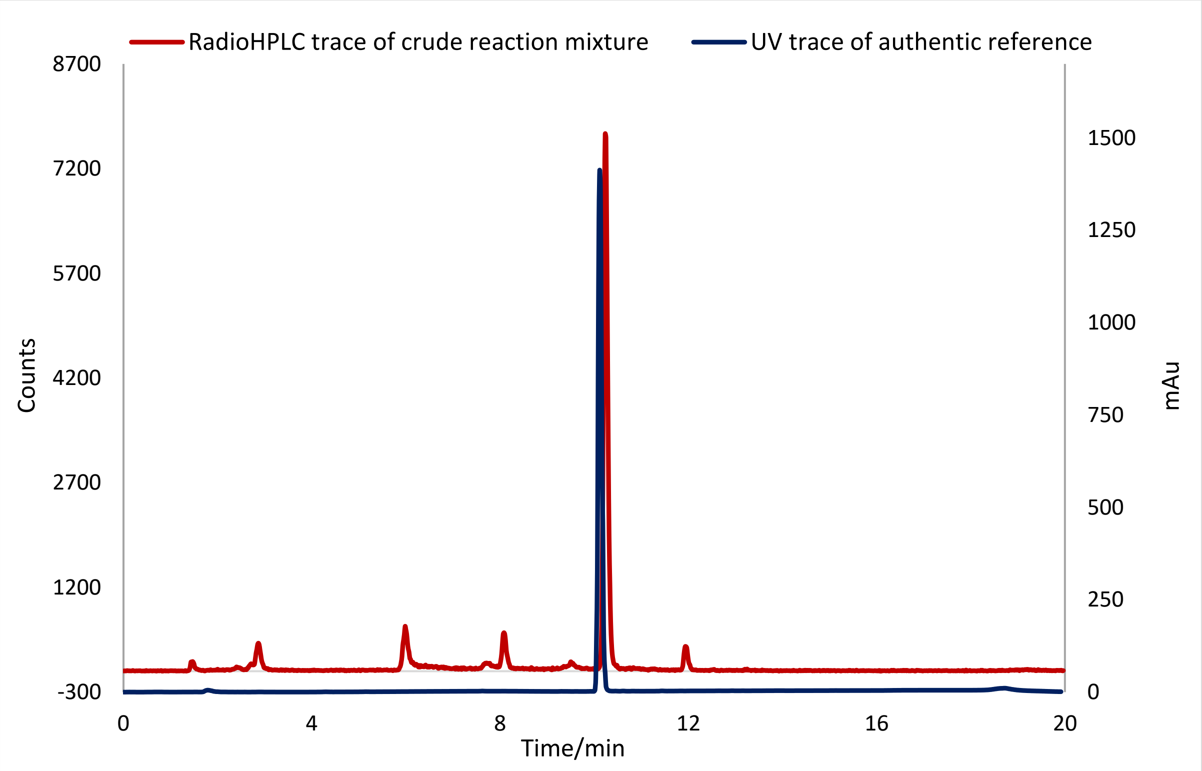

[18F](4-(Fluoromethyl)phenyl)(phenyl)methanone ([18F]20)

Prepared following the general procedure for the cross-coupling of [18F]**3** and analyzed by (radio)HPLC using conditions D or E.

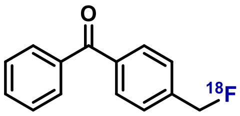

Run	RCC (%)
1	71
2	79
3	90
	**Average RCC:** 80 ± 8%*(n=3)*

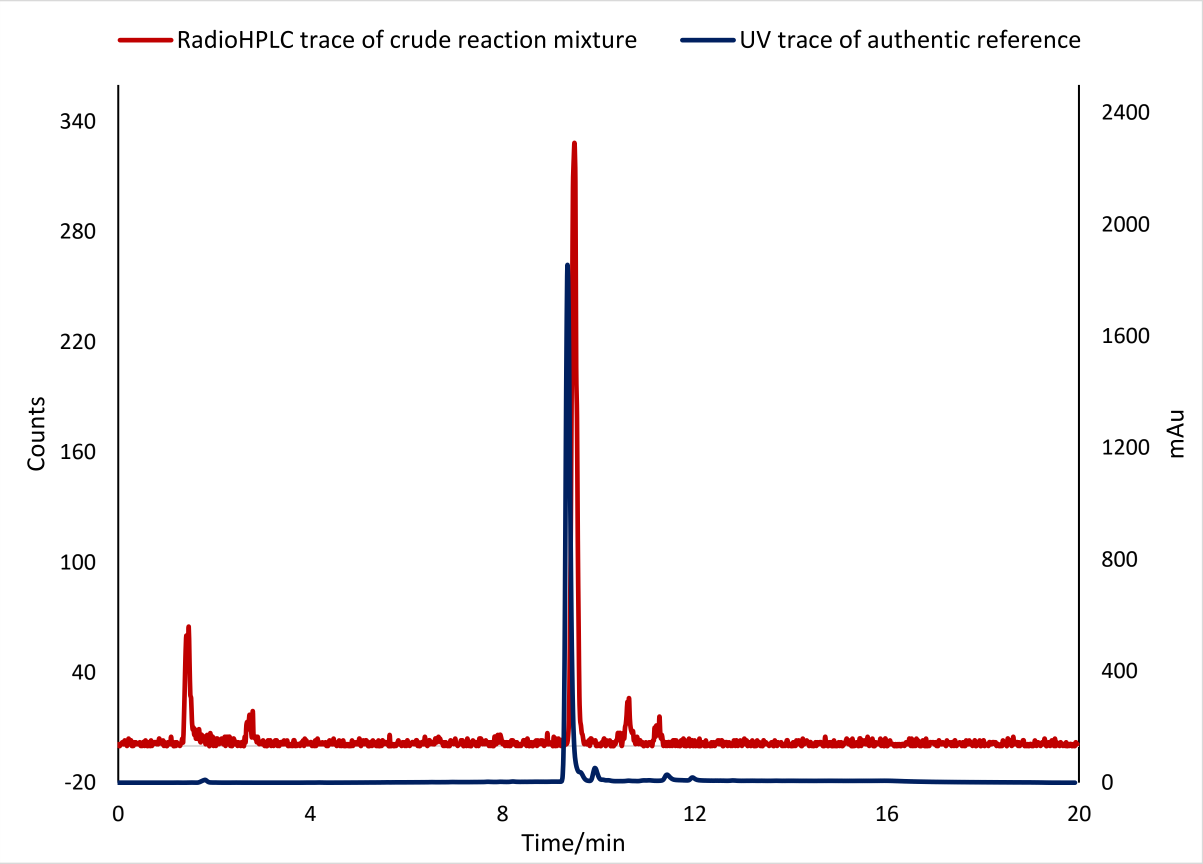

[^{18}F]2-(4-(Fluoromethyl)phenyl)thiophene ([^{18}F]21)

Prepared following the general procedure for the cross-coupling of [^{18}F]**3** and analyzed by (radio)HPLC using conditions A or C or E.

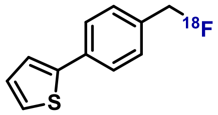

Run	RCC (%)
1	96
2	93
3	88
	**Average RCC:** 92 ± 3%$_{(n=3)}$

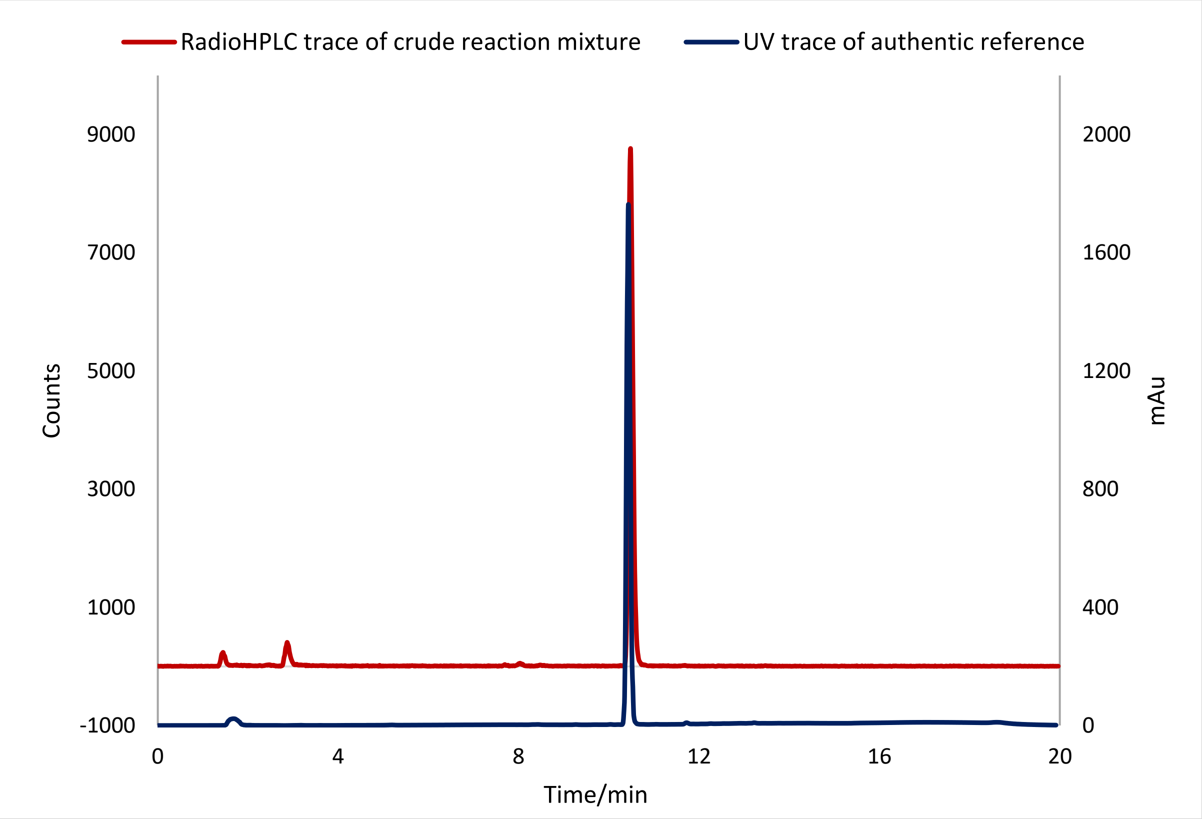

[^{18}F]4-((3-((Fluoromethyl)phenyl)sulfonyl)morpholine ([^{18}F]22)

Prepared following the general procedure for the cross-coupling of [^{18}F]**3** and analyzed by (radio)HPLC using conditions C or E.

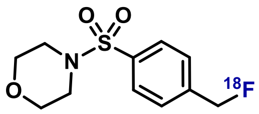

Run	RCC (%)
1	64
2	46
3	93
	**Average RCC:** 68 ± 19%$_{(n=3)}$

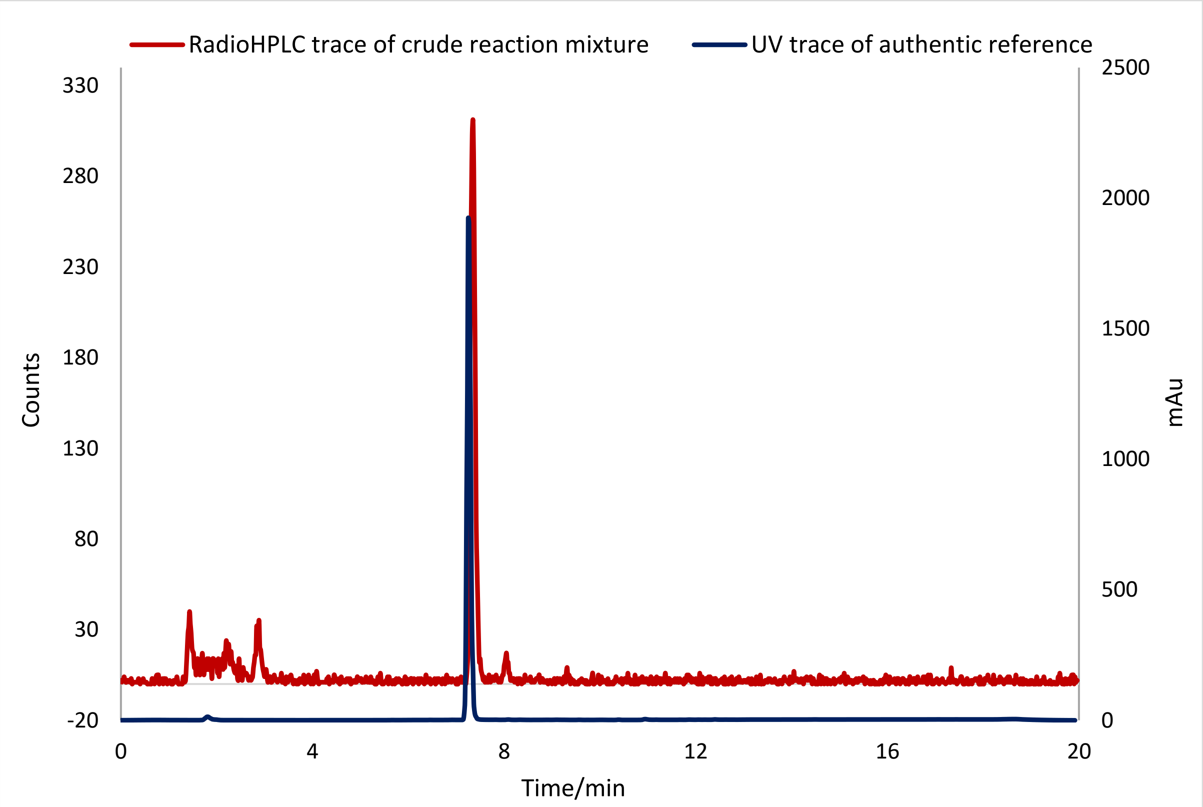

[^{18}F]methyl (*E*)-3-(4-(fluoromethyl)phenyl)acrylate ([^{18}F]23)

Prepared following the general procedure for the cross-coupling of [^{18}F]**3** and analyzed by (radio)HPLC using conditions E.

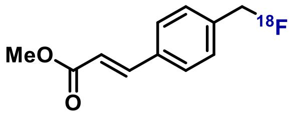

Run	RCC (%)
1	73
2	88
	**Average RCC:** 81 ± 8%$_{(n=2)}$

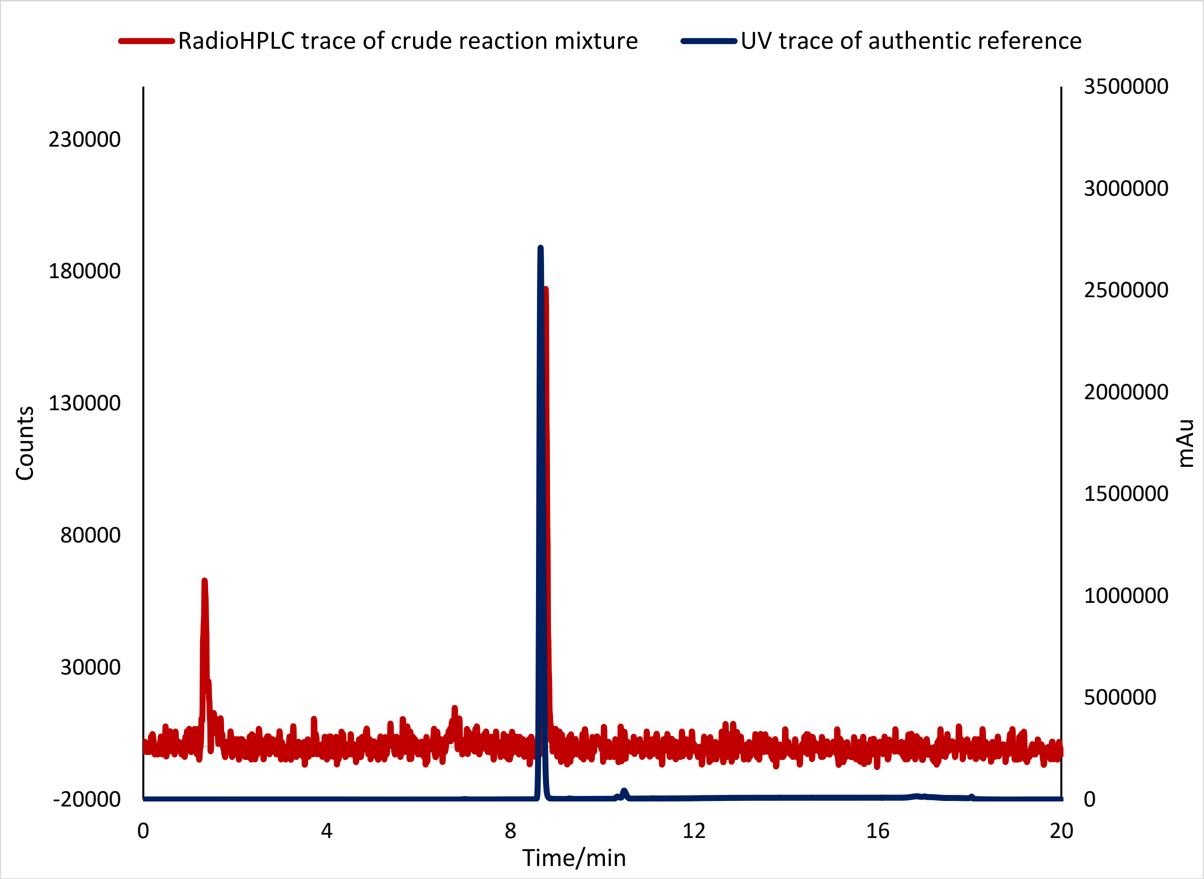

[^{18}F]6-(Fluoromethyl)-2-methylbenzothiazole ([^{18}F]24)

Prepared following the general procedure for the cross-coupling of [^{18}F]**3** and analyzed by (radio)HPLC using conditions A or C.

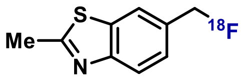

Run	RCC (%)
1	78
2	39
3	59
	**Average RCC:** 59 ± 16%$_{(n=3)}$

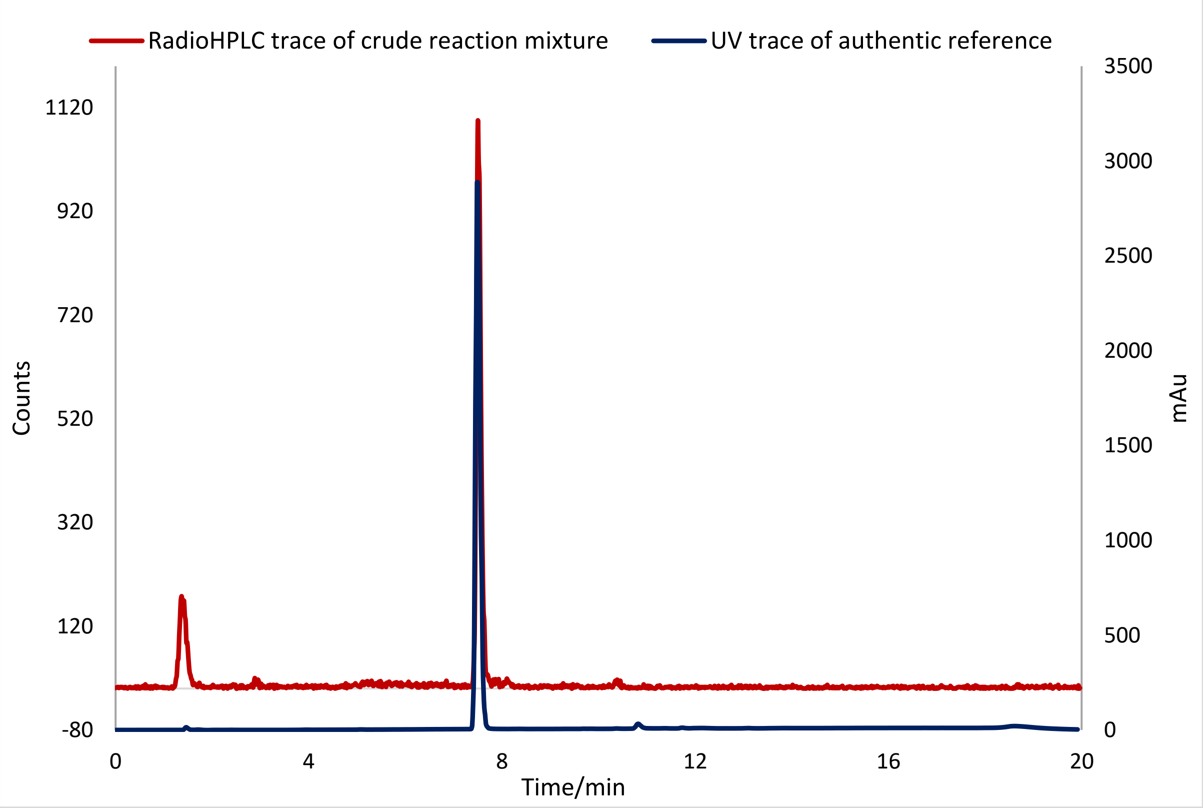

[^{18}F]2-(Fluoromethyl)benzothiazole ([^{18}F]25)

Prepared following the general procedure for the cross-coupling of [^{18}F]**3** and analyzed by (radio)HPLC using conditions B.

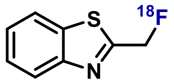

Run	RCC (%)
1	27
2	24
**Average RCC:** 26 ± 2%$_{(n=2)}$ with [^{18}F]**3**	
3	21
**RCC:** 21%$_{(n=1)}$ with [^{18}F,D_2]**3**	

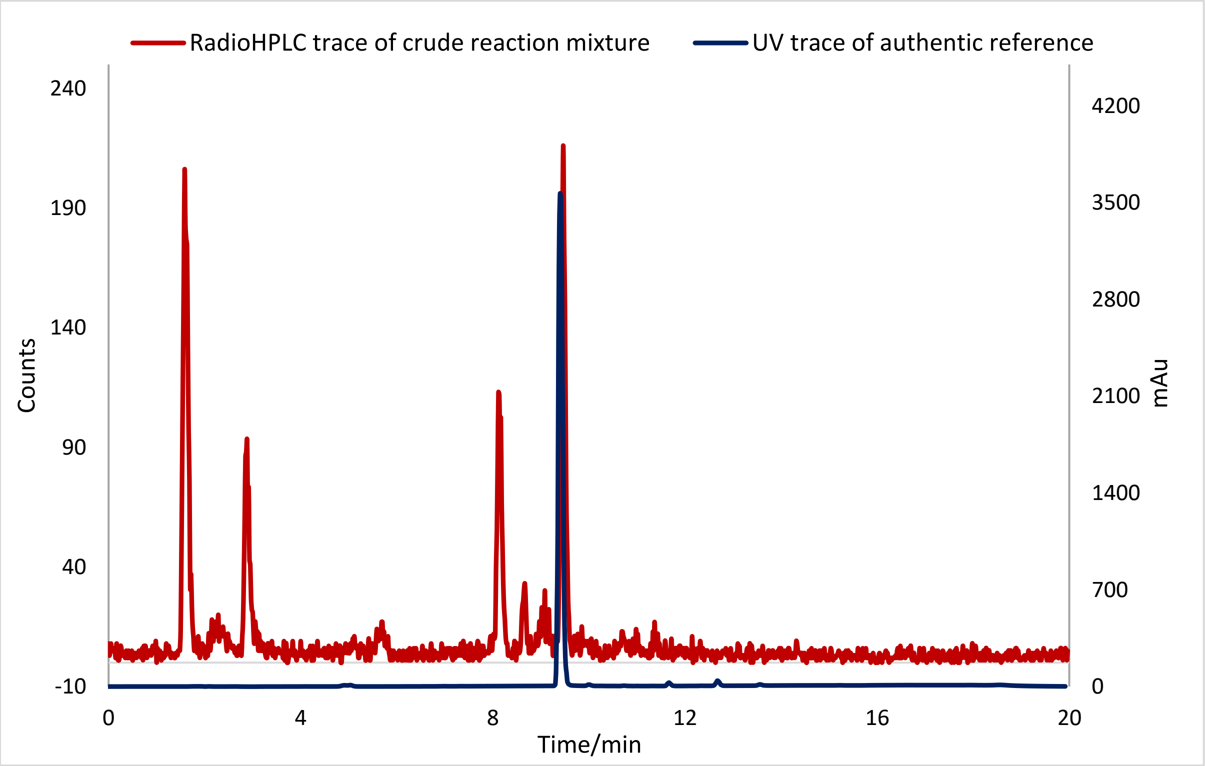

[^{18}F]3-((Fluoro)methyl)dibenzofuran ([^{18}F]26)

Prepared following the general procedure for the cross-coupling of [^{18}F]**3** and analyzed by (radio)HPLC using conditions C-E.

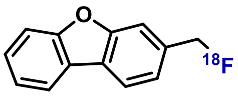

Run	RCC (%)
1	61
2	84
**Average RCC:** 73 ± 12%$_{(n=2)}$ with [^{18}F]**3**	
3	76
**RCC:** 76%$_{(n=1)}$ with [^{18}F,D_2]**3**	

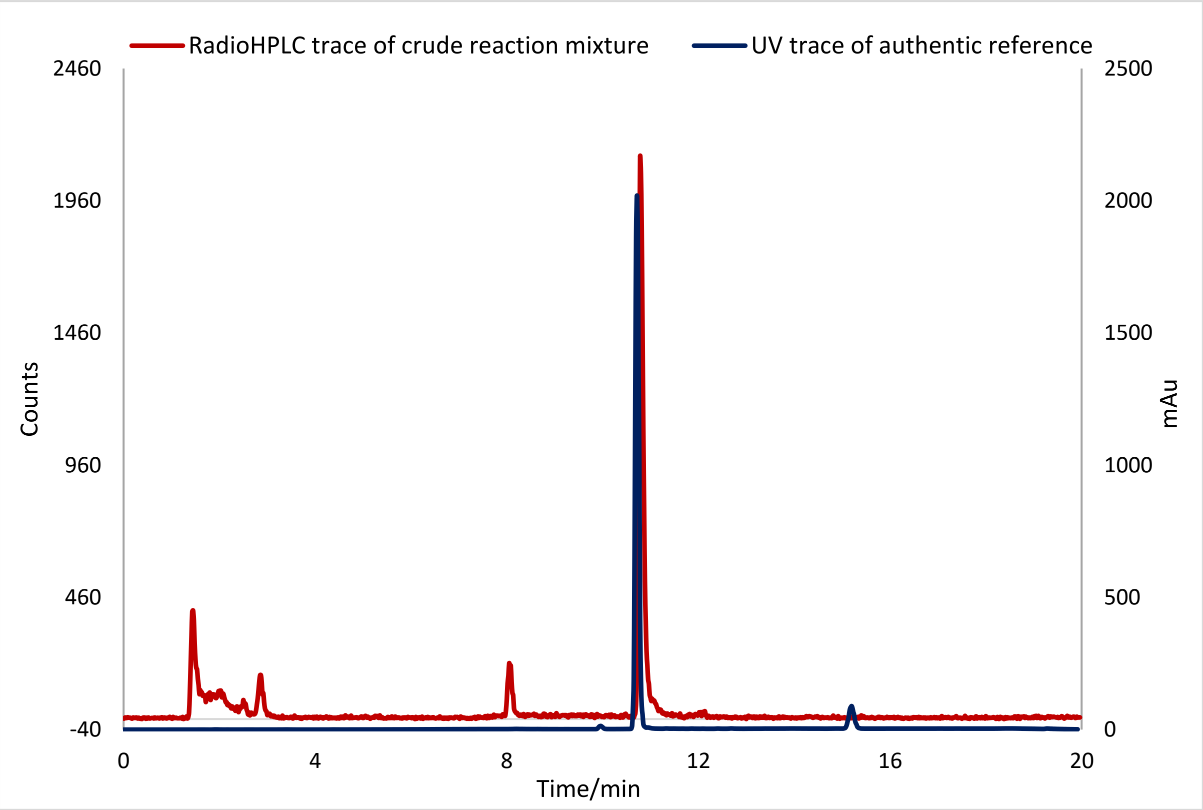

[^{18}F]6-((Fluoromethyl)quinoline ([^{18}F]27)

Prepared following the general procedure for the cross-coupling of [^{18}F]**3** and analyzed by (radio)HPLC using conditions C.

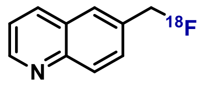

Run	RCC (%)
1	41
2	29
	**Average RCC:** 35 ± 6%$_{(n=2)}$

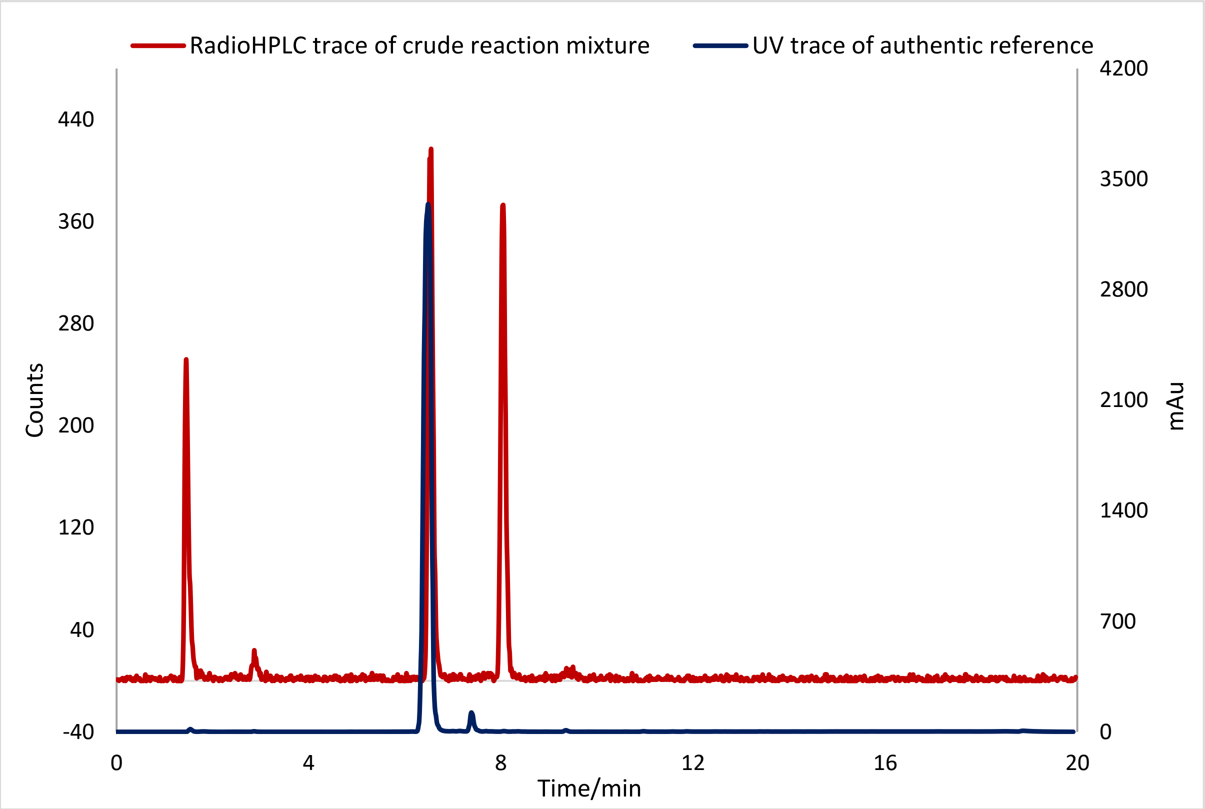

[^{18}F]5-(Fluoromethyl)-2-methoxypyridine ([^{18}F]28)

Prepared following the general procedure for the cross-coupling of [^{18}F]**3** and analyzed by (radio)HPLC using conditions D or E.

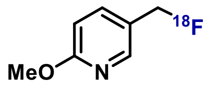

Run	RCC (%)
1	38
2	47
**Average RCC:** 43 ± 5%$_{(n=2)}$ with [^{18}F]**3**	
3	29
**RCC:** 29%$_{(n=1)}$ with [^{18}F,D_2]**3**	

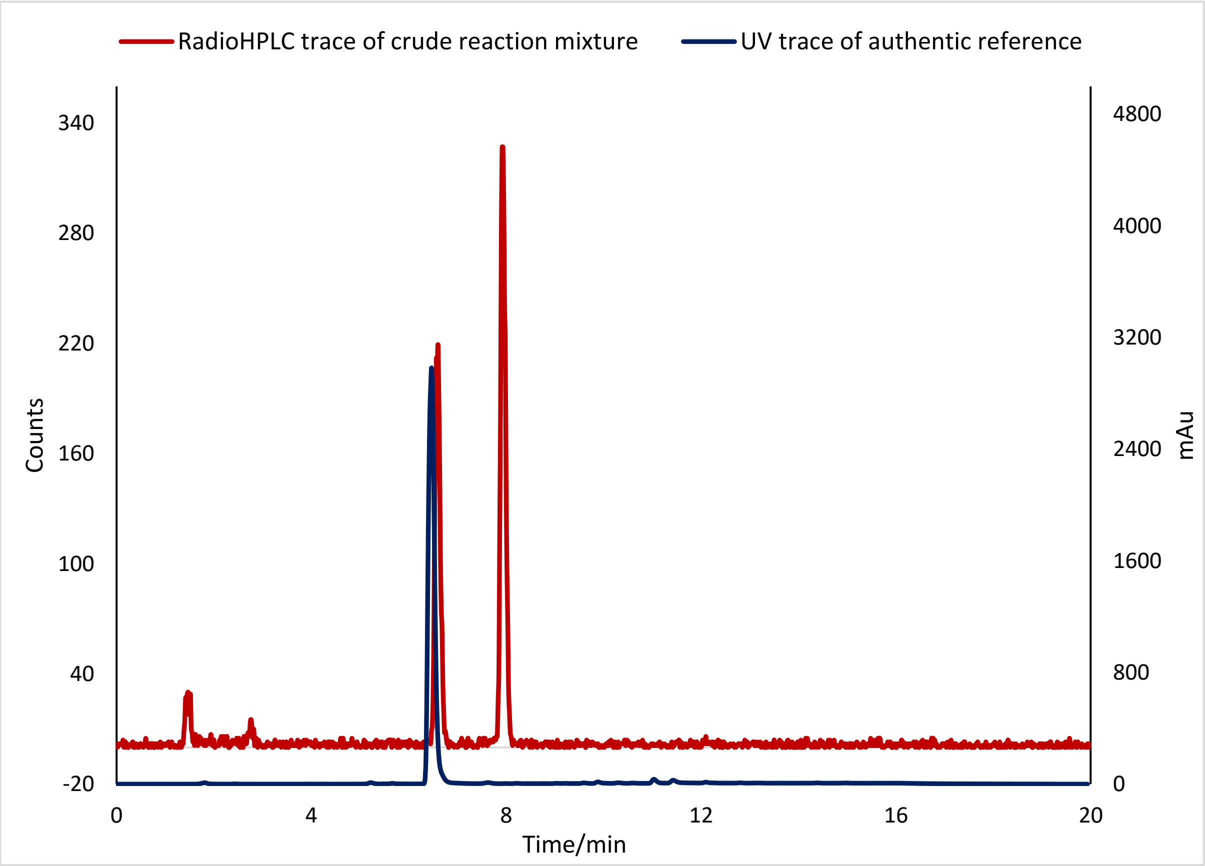

[^{18}F]3-(Fluoromethyl)-1-tosyl-1*H*-indole ([^{18}F]29)

Prepared following the general procedure for the cross-coupling of [^{18}F]**3** and analyzed by (radio)HPLC using conditions D or E.

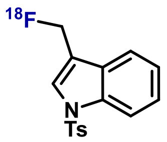

Run	RCC (%)
1	41
2	72
**Average RCC:** 57 ± 16%$_{(n=2)}$ with [^{18}F]**3**	
3	36
**RCC:** 36%$_{(n=1)}$ with [^{18}F,D_2]**3**	

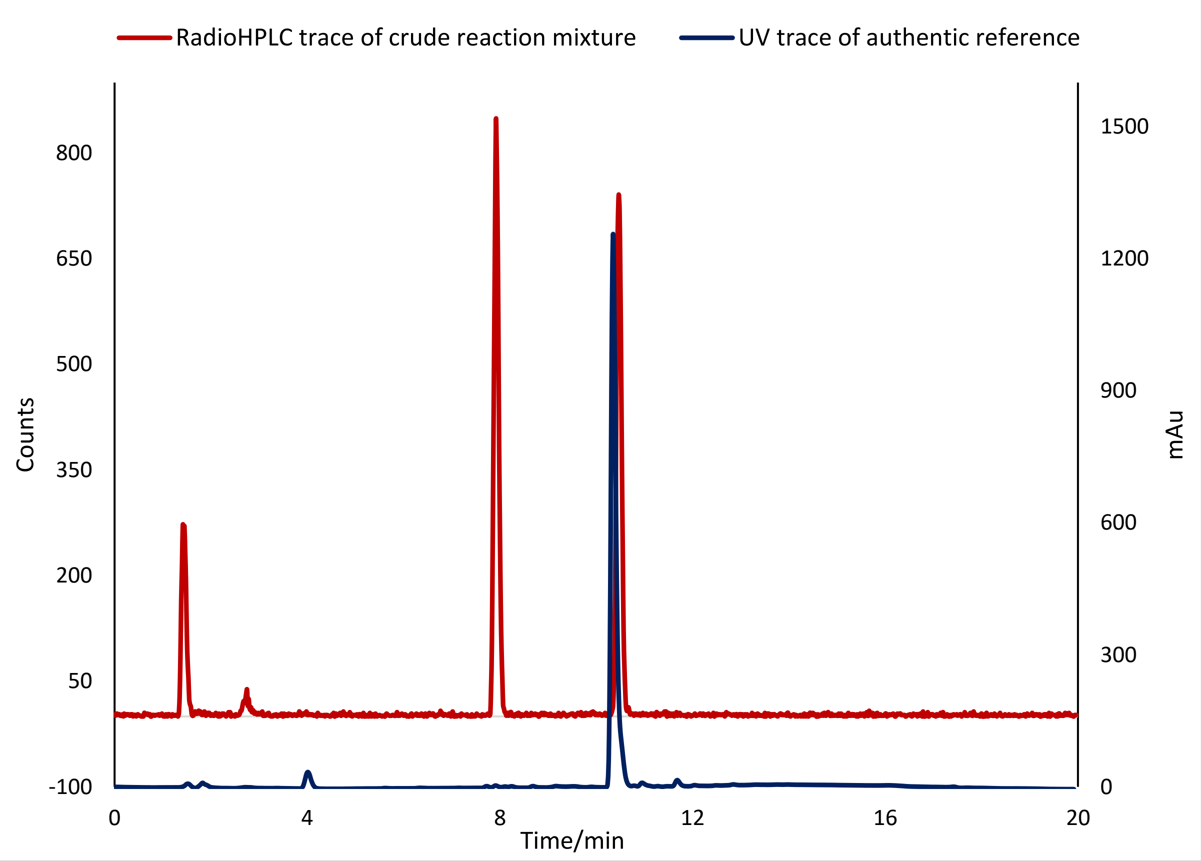

[¹⁸F]6-(Fluoromethyl)-1-methyl-1*H*-indazole ([¹⁸F]30)

Prepared following the general procedure for the cross-coupling of [^{18}F]**3** and analyzed by (radio)HPLC using conditions C-E.

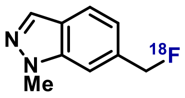

Run	RCC (%)
1	72
2	27
**Average RCC:** 50 ± 23%$_{(n=2)}$ with [^{18}F]**3**	
3	72
**RCC:** 72%$_{(n=1)}$ with [^{18}F,D_2]**3**	

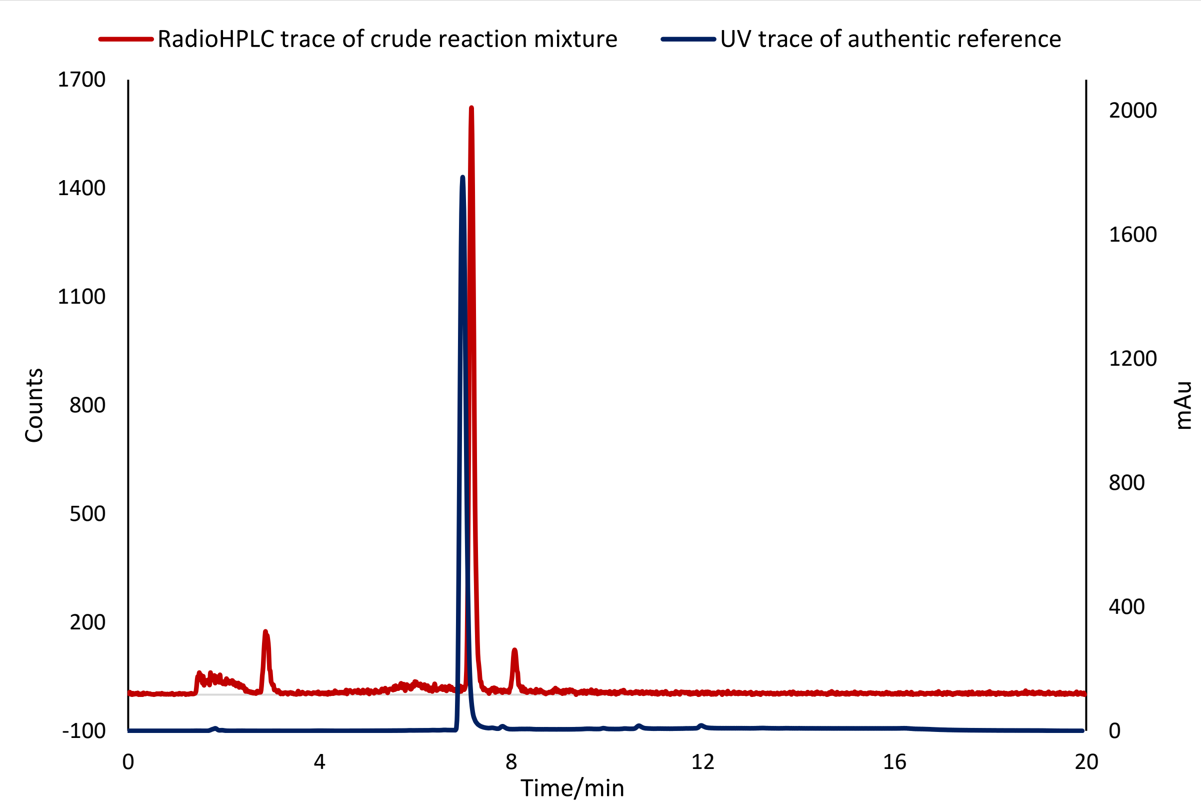

[^{18}F]*tert*-Butyl 5'-(fluoromethyl)-3'*H*-spiro[azetidine-3,1'-isobenzofuran]-1-carboxylate ([^{18}F]31)

Prepared following the general procedure for the cross-coupling of [^{18}F]**3** and [^{18}F,D_2]**3** and analyzed by (radio)HPLC using conditions A or C.

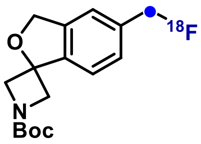

Run	RCC (%)
1	85
2	56
**Average RCC:** 71 ± 15%$_{(n=2)}$ with [^{18}F]**3**	
3	67
**RCC:** 67%$_{(n=1)}$ with [^{18}F,D_2]**3**	

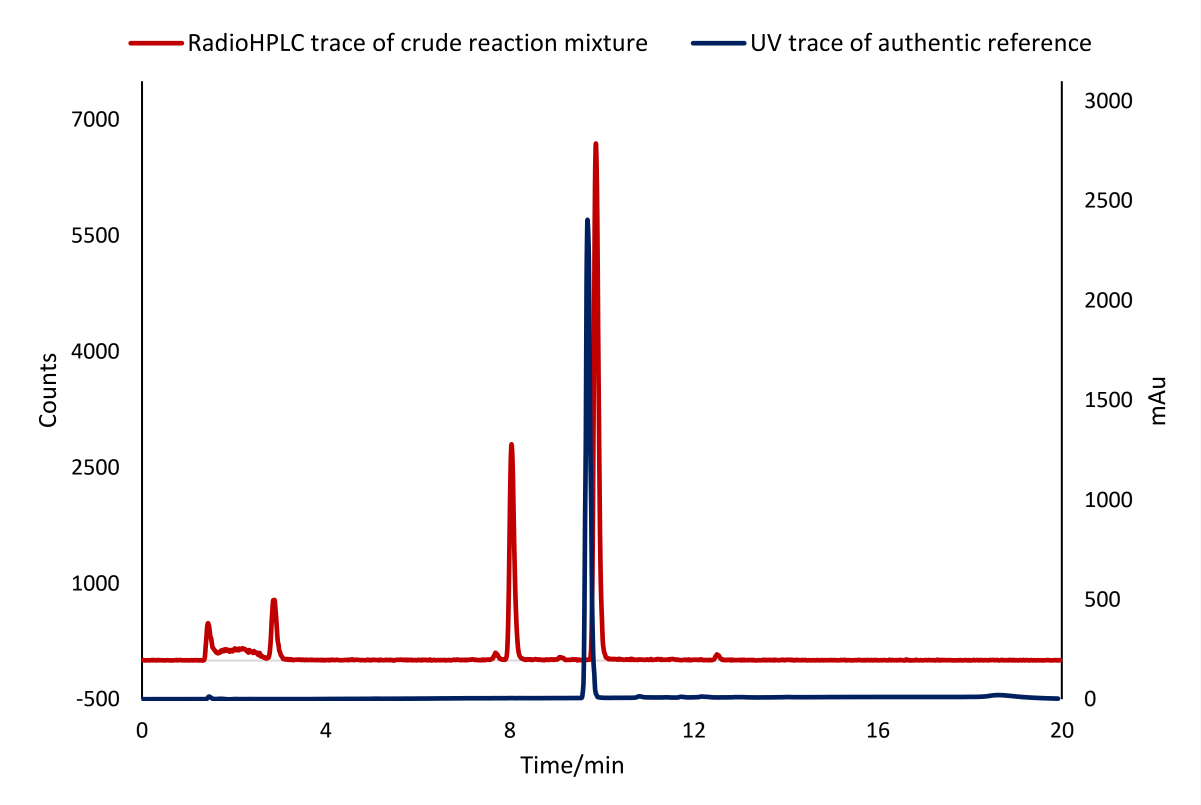

[^{18}F]3-((Fluoro)methyl)dibenzofuran ([^{18}F]32)

Prepared following the general procedure for the cross-coupling of [^{18}F]**3** and [^{18}F,D_2]**3** and analyzed by (radio)HPLC using conditions C or E.

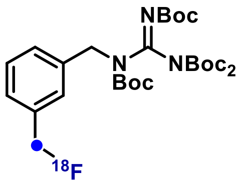

Run	RCC (%)
1	71
**RCC:** 71%$_{(n=1)}$ with [^{18}F]**3**	
2	49
3	80
**Average RCC:** 65 ± 16%$_{(n=2)}$ with [^{18}F,D_2]**3**	

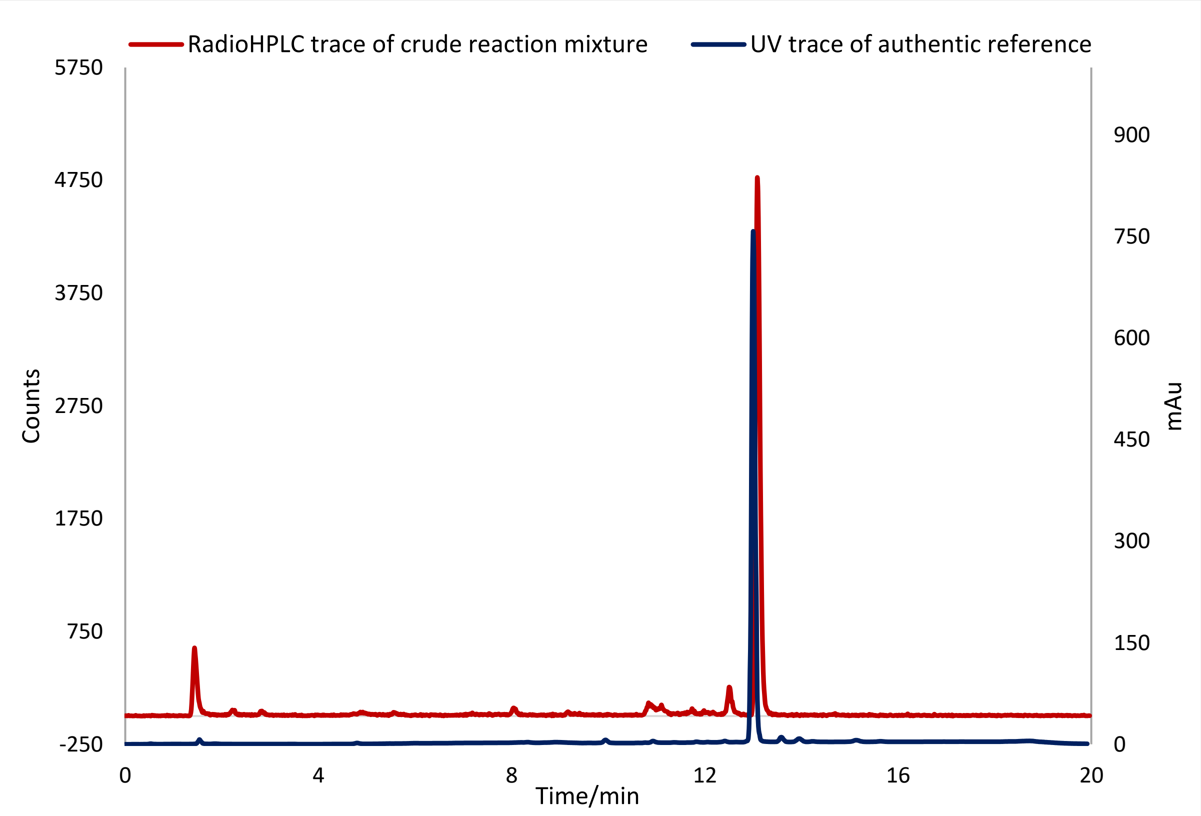

[^{18}F]2-(4-(Fluoromethyl)phenyl)-6-methoxybenzo[*d*]thiazole ([^{18}F]33)

Prepared following the general procedure for the cross-coupling of [^{18}F]**3** and [^{18}F,D_2]**3** and analyzed by (radio)HPLC using conditions D or E.

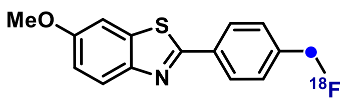

Run	RCC (%)
1	55
**RCC:** 55%$_{(n=1)}$ with [^{18}F]**3**	
2	63
3	62
**Average RCC:** 63 ± 1%$_{(n=2)}$ with [^{18}F,D_2]**3**	

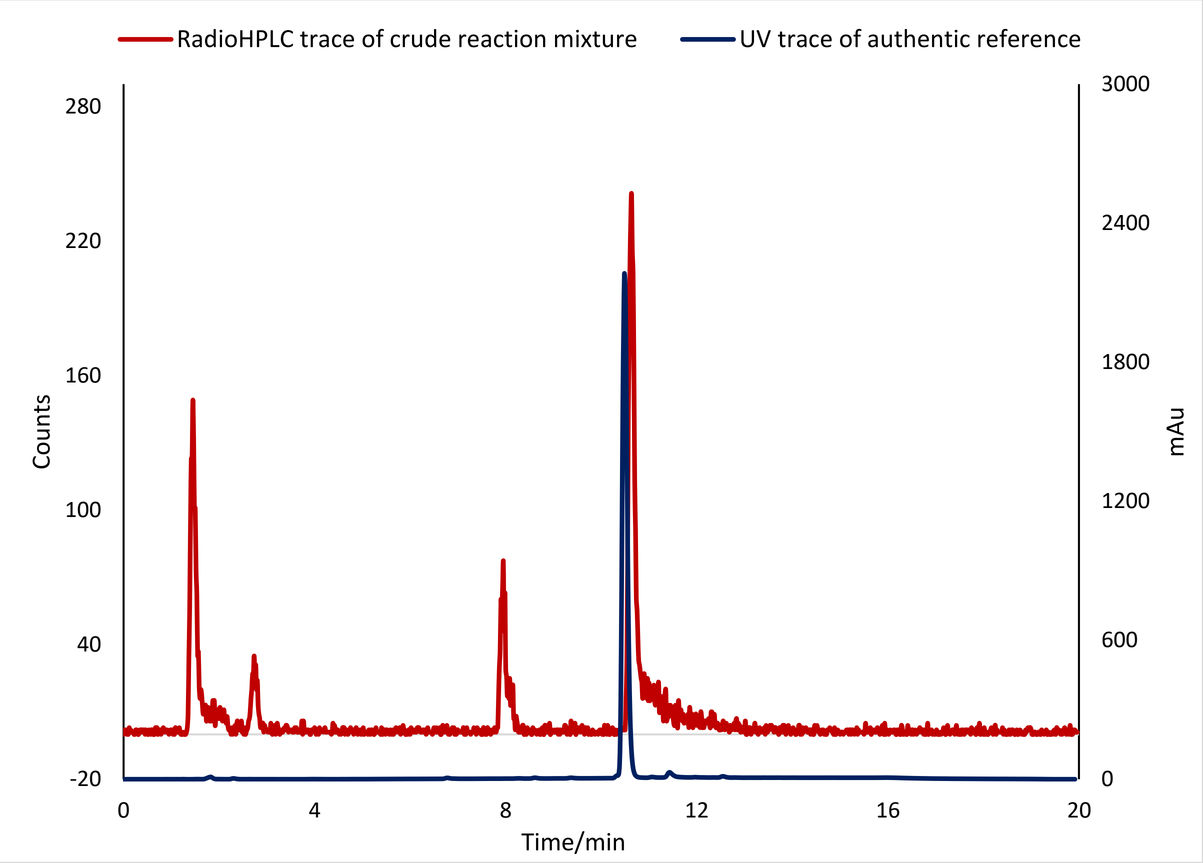

[^{18}F]Methyl (R)-2-(bis(*tert*-butoxycarbonyl)amino)-3-(4-(fluoromethyl)phenyl)propanoate ([^{18}F]34)

Prepared following the general procedure for the cross-coupling of [^{18}F,D_2]**3** and analyzed by (radio)HPLC using conditions E, at 195 nm.

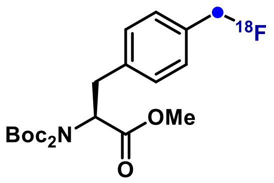

Run	RCC (%)
1	54
2	58
3	57
**Average RCC:** 56 ± 2%$_{(n=3)}$ with [^{18}F,D_2]**3**	

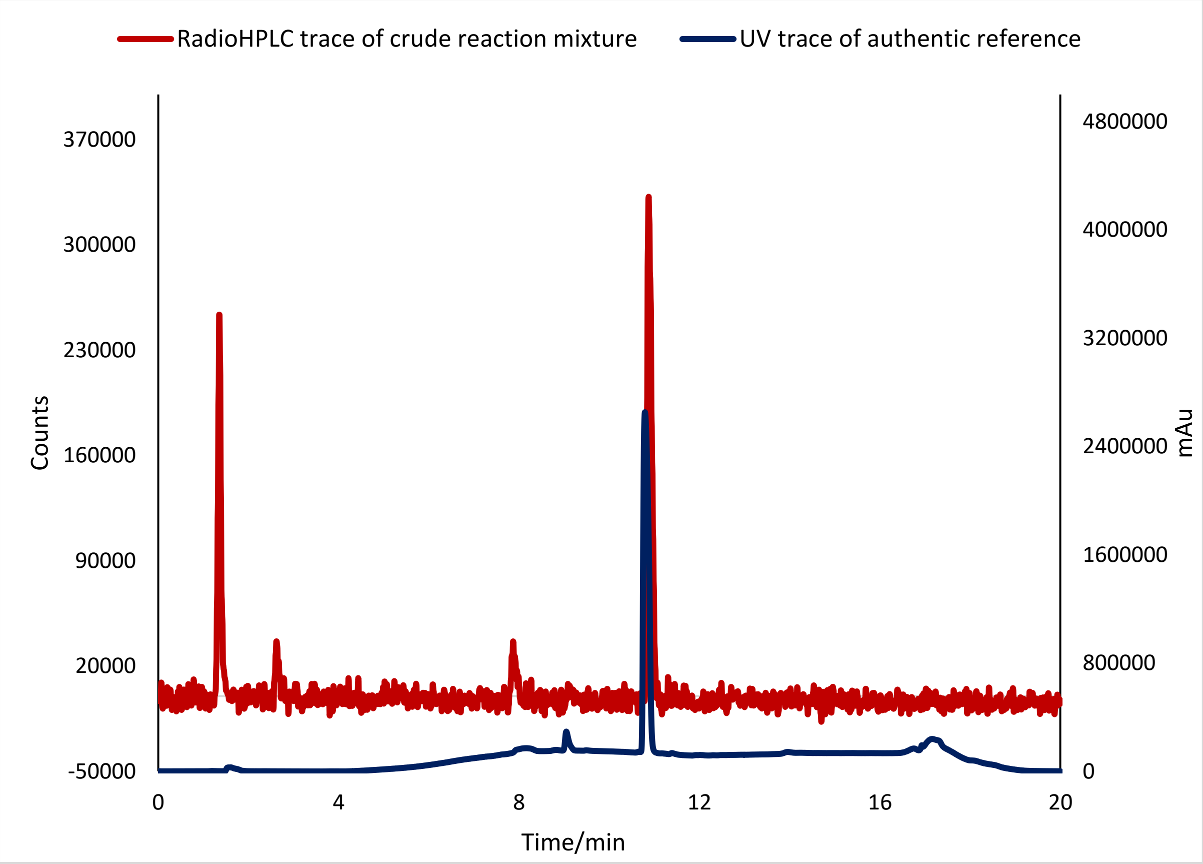

[^{18}F](8*R*,9*S*,13*S*,14*S*)-3-(Fluoromethyl)-13-methyl-6,7,8,9,11,12,13,14,15,16-decahydro-17*H* cyclopenta[*a*]phenanthren-17-one ([^{18}F]35)

Prepared following the general procedure for the cross-coupling of [^{18}F]**3** and analyzed by (radio)HPLC using conditions E, at 202 nm.

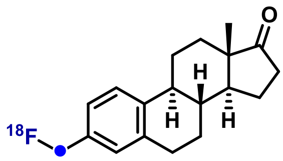

Run	RCC (%)
1	52
2	31
**Average RCC:** 42 ± 11%$_{(n=2)}$ with [^{18}F]**3**	
3	58
**RCC:** 58%$_{(n=1)}$ with [^{18}F,D_2]**3**	

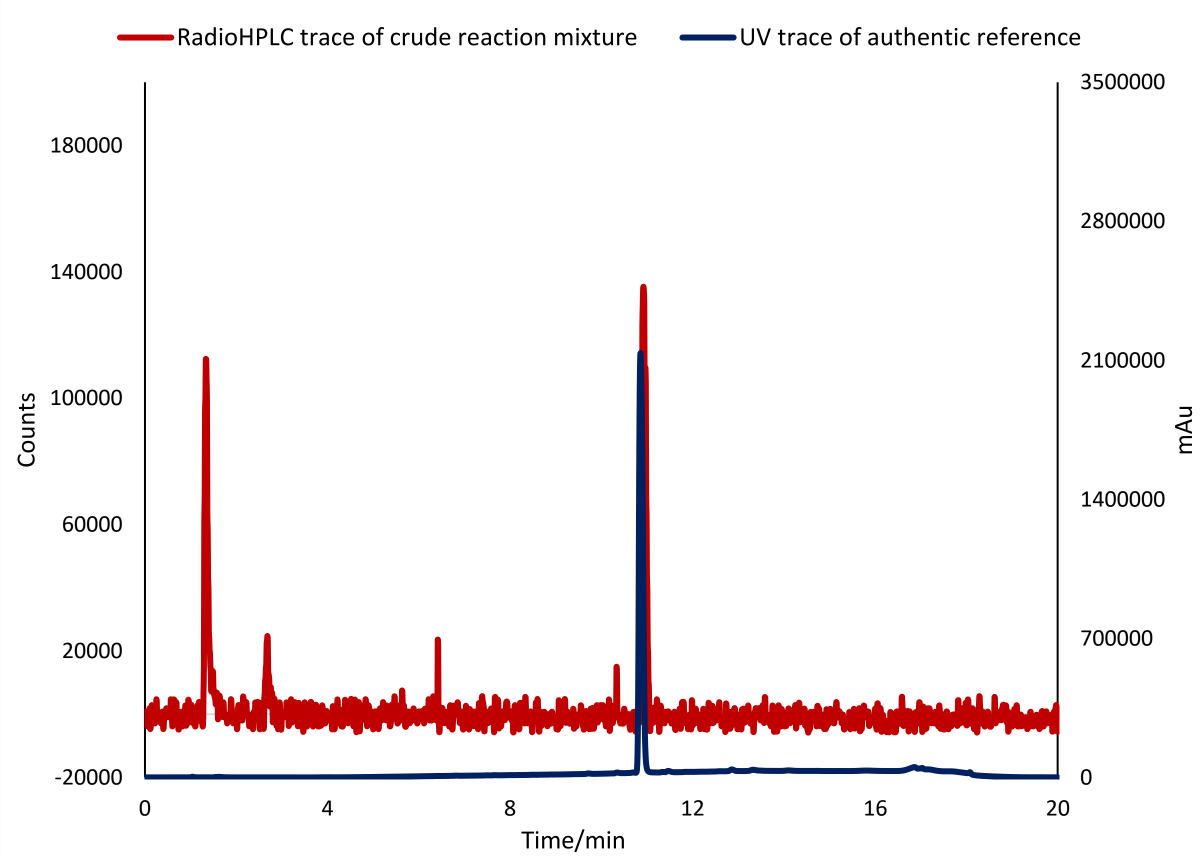

[^{18}F]Isopropyl 2-(4-(4-((fluoro-^{18}F)methyl)benzoyl)phenoxy)-2-methylpropanoate ([^{18}F]36)

Prepared following the general procedure for the cross-coupling of [^{18}F]**3** and [^{18}F,D_2]**3** and analyzed by (radio)HPLC using conditions C or E.

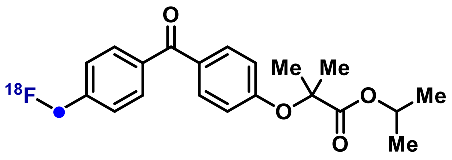

Run	RCC (%)
1	61
2	65
**Average RCC:** 63 ± 2%$_{(n=2)}$ with [^{18}F]**3**	
3	62
**RCC:** 62%$_{(n=1)}$ with [^{18}F,D_2]**3**	

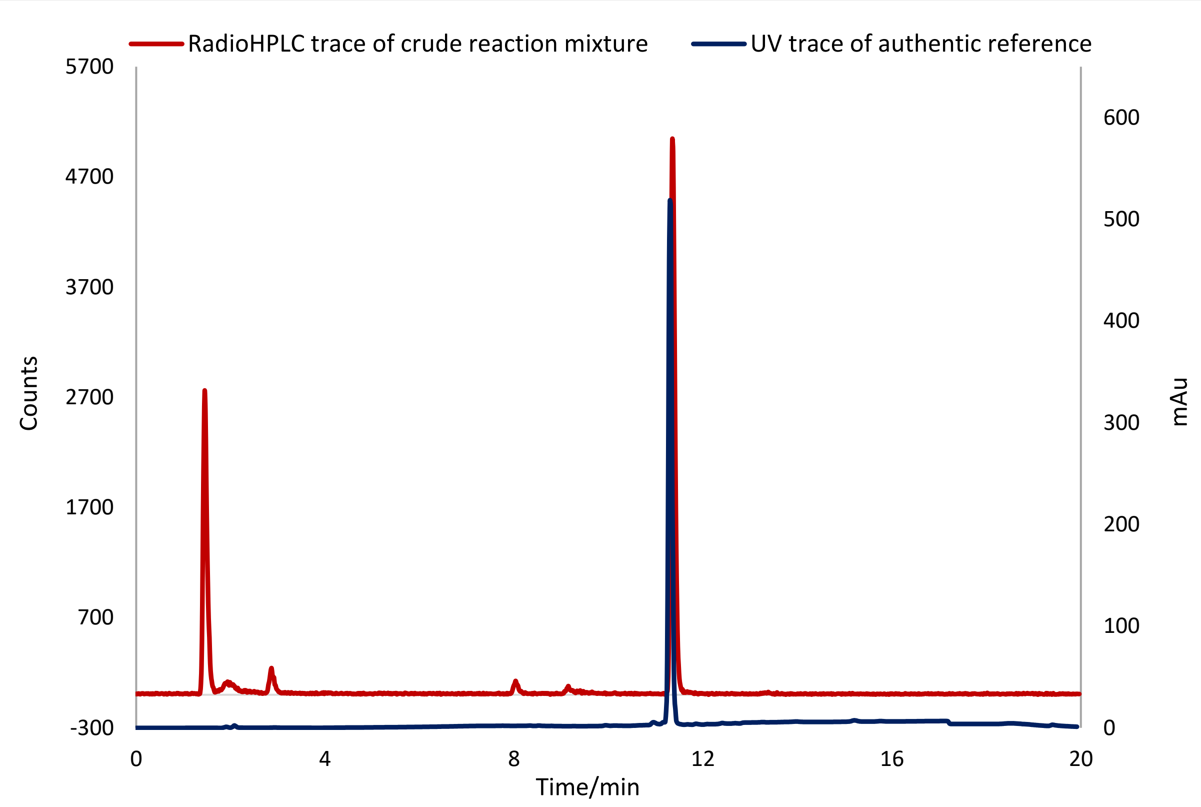

[^{18}F]4-(5-(4-((Fluoro)methyl)phenyl)-3-(trifluoromethyl)-1*H*-pyrazol-1-yl)benzenesulfonamide ([^{18}F]37)

Prepared following the general procedure for the cross-coupling of [^{18}F]**3** and [^{18}F,D_2]**3** and analyzed by (radio)HPLC using conditions C.

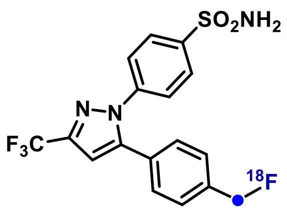

Run	RCC (%)
1	73
2	57
**Average RCC:** 65 ± 8%$_{(n=2)}$ with [^{18}F]**3**	
3	57
4	77
**Average RCC:** 67 ± 10%$_{(n=2)}$ with [^{18}F,D_2]**3**	

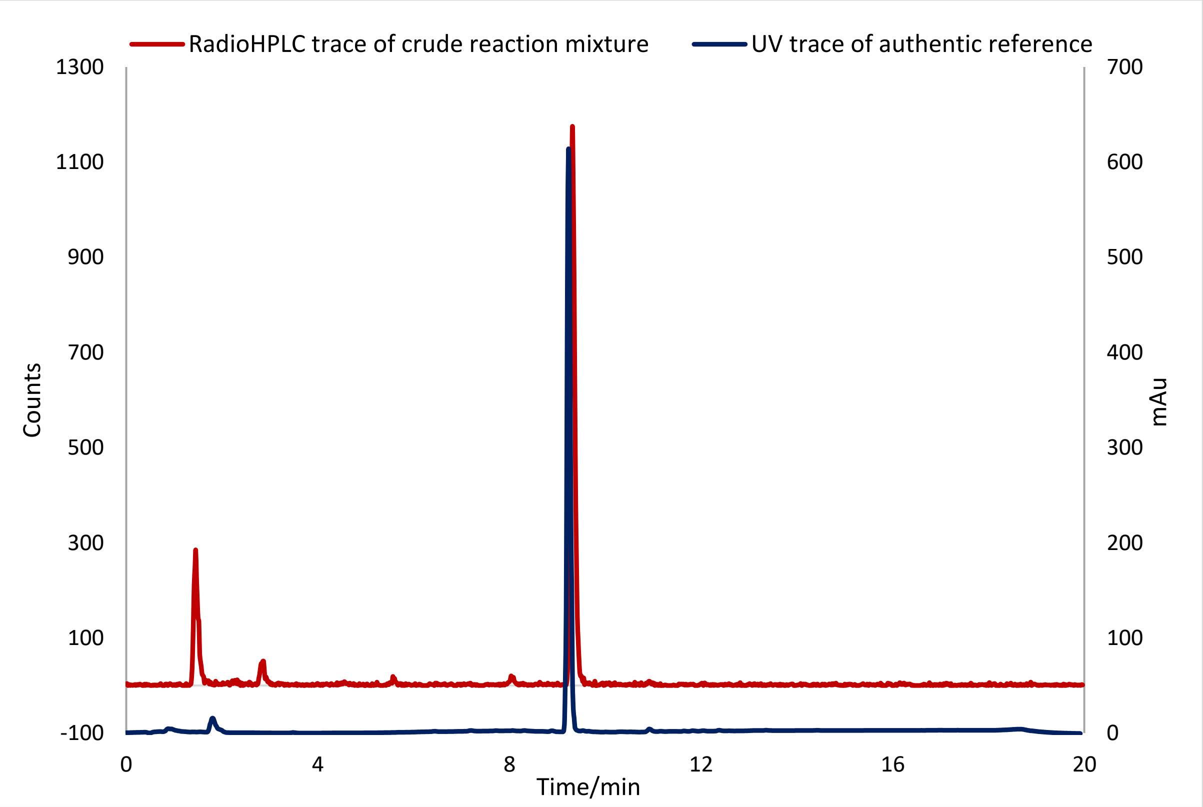

[^{18}F]3-Cyclopropyl-1-(2-fluoro-4-((fluoro)methyl)phenyl)-5-hydroxy-6,8-dimethylpyrido[2,3-*d*]pyrimidine-2,4,7(1*H*,3*H*,8*H*)-trione ([^{18}F]38)

Prepared following the general procedure for the cross-coupling of [^{18}F]**3** and [^{18}F,D_2]**3** and analyzed by (radio)HPLC using conditions C.

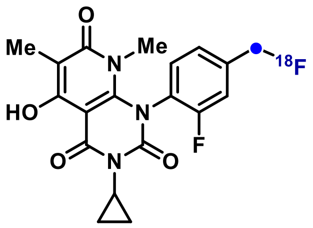

Run	RCC (%)
1	67
**RCC:** 67%$_{(n=1)}$ with [^{18}F]**3**	
2	62
3	40
**Average RCC:** 51 ± 11%$_{(n=2)}$ with [^{18}F,D_2]**3**	

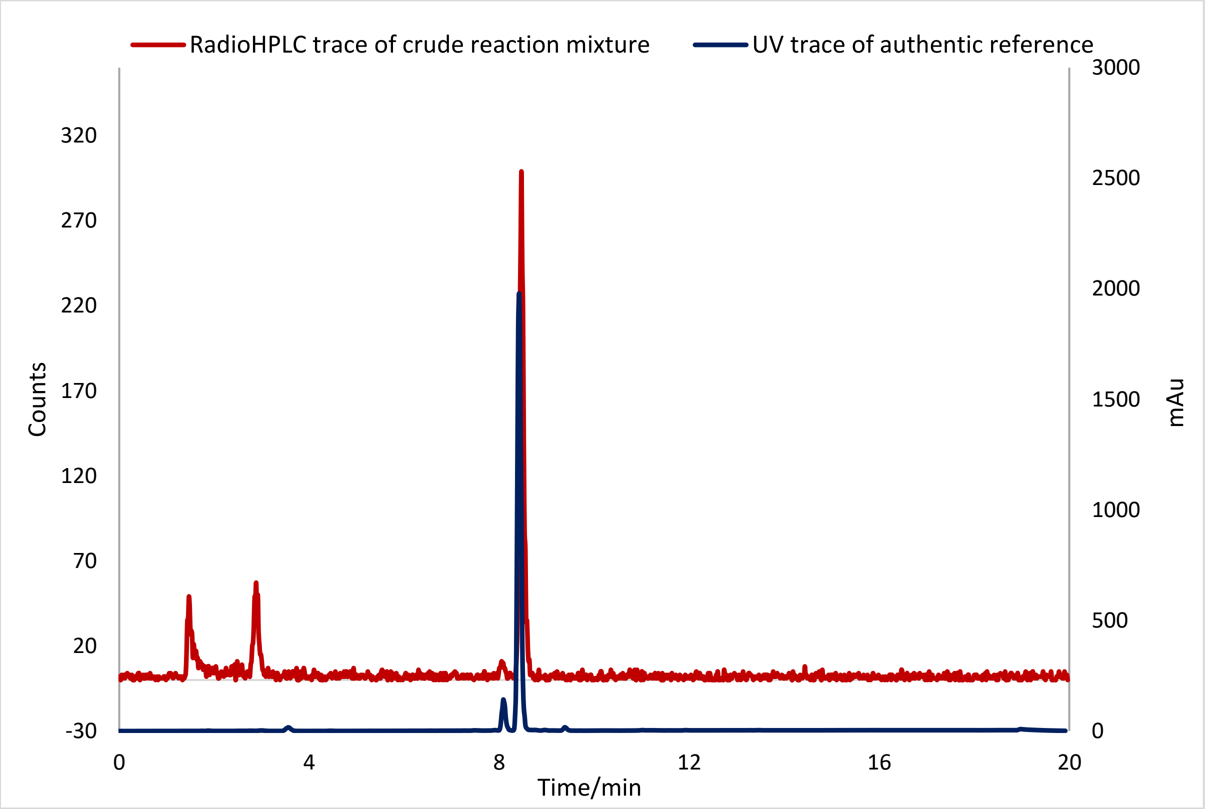

[^{18}F]4-(4-Fluoro-3-(4-(4-(fluoromethyl)benzoyl)piperazine-1-carbonyl)benzyl)phthalazin-1(2*H*)-one ([^{18}F]39)

Prepared following the general procedure for the cross-coupling of [^{18}F]**3** and [^{18}F,D_2]**3** and analyzed by (radio)HPLC using conditions C or D.

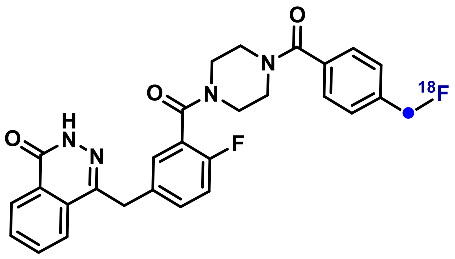

Run	RCC (%)
1	40
2	50
**Average RCC:** 45 ± 5%$_{(n=2)}$ with [^{18}F]**3**	
3	63
**RCC:** 63%$_{(n=1)}$ with [^{18}F,D_2]**3**	

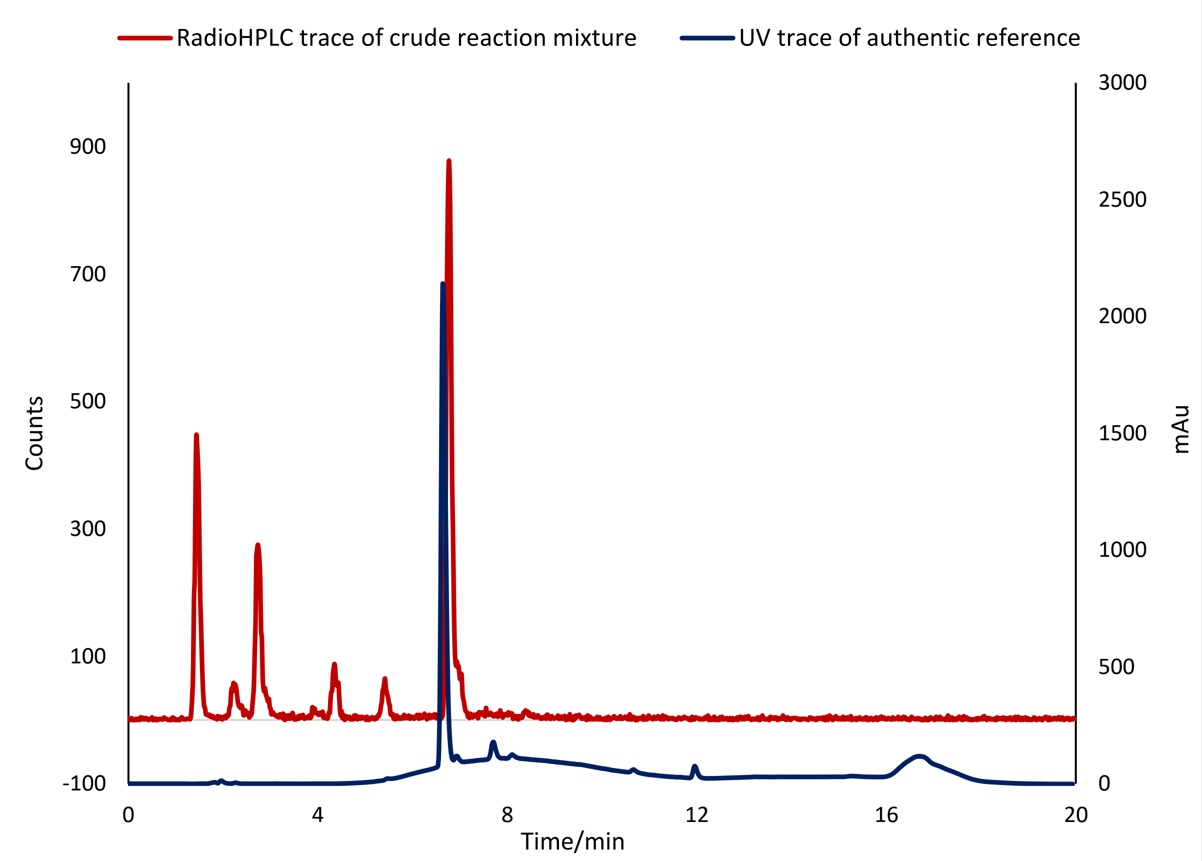

References

(1) Senatore, R.; Malik, M.; Spreitzer, M.; Holzer, W.; Pace, V. Direct and Chemoselective Electrophilic Monofluoromethylation of Heteroatoms (*O*-, *S*-, *N*-, *P*-, *Se*-) with Fluoroiodomethane. *Org. Lett.* **2020**, *22*, 1345–1349.

(2) Zhao, Y.; Gao, B.; Hu, J. From Olefination to Alkylation: In-Situ Halogenation of Julia–Kocienski Intermediates Leading to Formal Nucleophilic Iodo- and Bromodifluoromethylation of Carbonyl Compounds. *J. Am. Chem. Soc.* **2012**, *134*, 5790–5793.

(3) Zhu, L.; Ni, C.; Zhao, Y.; Hu, J. 1-*tert*-Butyl-1*H*-tetrazol-5-yl fluoromethyl sulfone ($TBTSO_2CH_2F$): a versatile fluoromethylidene synthon and its use in the synthesis of monofluorinated alkenes via Julia–Kocienski olefination. *Tetrahedron* **2010**, *66*, 5089–5100.

(4) Xing, B.; Ni, C.; Hu, J. Copper-Mediated Di- and Monofluoromethanesulfonylation of Arenediazonium Tetrafluoroborates: Probing the Fluorine Effect. *Chin. J. Chem.* **2018**, *36*, 206–212.

(5) Singh, G.; Kumar, R.; Swett, J.; Zajc, B. Modular Synthesis of N-Vinyl Benzotriazoles. *Org. Lett.* **2013**, *15*, 4086–4089.

(6) Mamat, C.; Franke, M.; Peppel, T.; Köckerling, M.; Steinbach, J. Synthesis, Structure Determination, and (Radio-)Fluorination of Novel Functionalized Phosphanes Suitable for the Traceless Staudinger Ligation. *Tetrahedron* **2011**, *67*, 4521–4529.

(7) Rosas Vargas, D.; Cook, S. P. Palladium Nanoparticles: Chemoselective Control for Reductive Heck with Aryl Triflates and 2,3-Dihydrofuran. *Tetrahedron* **2018**, *74*, 3314–3317.

(8) Scheepstra, M.; Doyle, L.; Fabritius, C.-H.; Mindich, A. Heterocyclic Pla2g15 Inhibitors and Their Use in Therapy, in the Treatment of Diseases Characterized by Lysosomal Dysregulation. WO2025153715A1, 2025.

(9) Wang, L.; Jacobson, O.; Avdic, D.; Rotstein, B. H.; Weiss, I. D.; Collier, L.; Chen, X.; Vasdev, N.; Liang, S. H. Ortho-Stabilized ^{18}F-Azido Click Agents and Their Application in PET Imaging with Single-Stranded DNA Aptamers. *Angew. Chem. Int. Ed.* **2015**, *54*, 12777–12781.

(10) Švec, P.; Nový, Z.; Kučka, J.; Petřík, M.; Sedláček, O.; Kuchař, M.; Lišková, B.; Medvedíková, M.; Kolouchová, K.; Groborz, O.; Loukotová, L.; Konefał, R. Ł.; Hajdúch, M.; Hrubý, M. Iodinated Choline Transport-Targeted Tracers. *J. Med. Chem.* **2020**, *63*, 15960–15978.

(11) Tolnai, G. L.; Nilsson, U. J.; Olofsson, B. Efficient O-Functionalization of Carbohydrates with Electrophilic Reagents. *Angew. Chem. Int. Ed.* **2016**, *55*, 11226–11230.

(12) Wilson, T. C.; McSweeney, G.; Preshlock, S.; Verhoog, S.; Tredwell, M.; Cailly, T.; Gouverneur, V. Radiosynthesis of SPECT Tracers via a Copper Mediated ^{123}I Iodination of (Hetero)Aryl Boron Reagents. *Chem. Commun.* **2016**, *52*, 13277–13280.

(13) Hu, J.; Gao, B.; Li, L.; Ni, C.; Hu, J. Palladium-Catalyzed Monofluoromethylation of Arylboronic Esters with Fluoromethyl Iodide. *Org. Lett.* **2015**, *17*, 3086–3089.

(14) Zhang, D.; Le, L.; Qiu, R.; Wong, W.-Y.; Kambe, N. Nickel- and Palladium-Catalyzed Cross-Coupling Reactions of Organostibines with Organoboronic Acids. *Angew. Chem. Int. Ed.* **2021**, *60*, 3104–3114.

(15) Lee, E.; Hooker, J. M.; Ritter, T. Nickel-Mediated Oxidative Fluorination for PET with Aqueous [^{18}F] Fluoride. *J. Am. Chem. Soc.* **2012**, *134*, 17456–17458.

(16) Chen, X.-Y.; Li, Y.-N.; Wu, Y.; Bai, J.; Guo, Y.; Wang, P. Cu-Mediated Thianthrenation and Phenoxathiination of Arylborons. *J. Am. Chem. Soc.* **2023**, *145*, 10431–10440.

(17) Zhao, Q.; Lu, L.; Shen, Q. Direct Monofluoromethylthiolation with *S*-(Fluoromethyl) Benzenesulfonothioate. *Angew. Chem. Int. Ed.* **2017**, *56*, 11575–11578.

(18) Xiao, Y.-L.; Min, Q.-Q.; Xu, C.; Wang, R.-W.; Zhang, X. Nickel-Catalyzed Difluoroalkylation of (Hetero)Arylborons with Unactivated 1-Bromo-1,1-Difluoroalkanes. *Angew. Chem. Int. Ed.* **2016**, *55*, 5837–5841.

(19) Sheng, J.; Ni, H.-Q.; Zhang, H.-R.; Zhang, K.-F.; Wang, Y.-N.; Wang, X.-S. Nickel-Catalyzed Reductive Cross-Coupling of Aryl Halides with Monofluoroalkyl Halides for Late-Stage Monofluoroalkylation. *Angew. Chem. Int. Ed.* **2018**, *57*, 7634–7639.
(20) Heijnen, D.; Tosi, F.; Vila, C.; Stuart, M. C. A.; Elsinga, P. H.; Szymanski, W.; Feringa, B. L. Oxygen Activated, Palladium Nanoparticle Catalyzed, Ultrafast Cross-Coupling of Organolithium Reagents. *Angew. Chem. Int. Ed.* **2017**, *56*, 3354–3359.
(21) Kim, J. Y.; Park, J. A.; Lee, K. C.; Lee, Y. J.; Kang, C. M. Novel Benzothiazole Derivatives and Use Thereof in Boron Neutron Capture Therapy. KR20220165359A, 2022.
(22) Wang, A.; Li, Y.; Lv, K.; Gao, R.; Wang, A.; Yan, H.; Qin, X.; Xu, S.; Ma, C.; Jiang, J.; Wei, Z.; Zhang, K.; Liu, M. Optimization and SAR Research at the Piperazine and Phenyl Rings of JNJ4796 as New Anti-Influenza A Virus Agents, Part 1. *Eur. J. Med. Chem.* **2021**, *222*, 113591.
(23) Rerkrachaneekorn, T.; Tankam, T.; Sukwattanasinitt, M.; Wacharasindhu, S. NaI-Mediated Oxidative Amidation of Benzyl Alcohols/Aromatic Aldehydes to Benzamides via Electrochemical Reaction. *Tetrahedron Lett.* **2021**, *70*, 153017.
(24) Battisti, V.; Moesslacher, J.; Abdelnabi, R.; Leyssen, P.; Rosales Rosas, A. L.; Langendries, L.; Aufy, M.; Studenik, C.; Kratz, J. M.; Rollinger, J. M.; Puerstinger, G.; Neyts, J.; Delang, L.; Urban, E.; Langer, T. Design, Synthesis, and Lead Optimization of Piperazinyl-Pyrimidine Analogues as Potent Small Molecules Targeting the Viral Capping Machinery of Chikungunya Virus. *Eur. J. Med. Chem.* **2024**, *264*, 116010.
(25) Zmuda, F.; Malviya, G.; Blair, A.; Boyd, M.; Chalmers, A. J.; Sutherland, A.; Pimlott, S. L. Synthesis and Evaluation of a Radioiodinated Tracer with Specificity for Poly(ADP-Ribose) Polymerase-1 (PARP-1) in Vivo. *J. Med. Chem.* **2015**, *58*, 8683–8693.
(26) Karaj, E.; Sindi, S. H.; Kuganesan, N.; Perera, L.; Taylor, W.; Tillekeratne, L. M. V. Tunable Cysteine-Targeting Electrophilic Heteroaromatic Warheads Induce Ferroptosis. *J. Med. Chem.* **2022**, *65*, 11788–11817.
(27) Ye, J.; Song, Q.; Pan, D.; Zeng, Z.; Gao, H.; Xu, H.; Yi, W.; Zhou, Z. Enantioselective Synthesis of Indole-Derivated Axially Chiral Frameworks via CpXRh(III)-Catalyzed Domino Cyclization/C3-Arylation of 2-Alkynylanilines with 1-Diazonaphthoquinones. *Adv. Synth. Catal.* **2024**, *366*, 1064–1069.
(28) Siméon, F. G.; Wendahl, M. T.; Pike, V. W. Syntheses of 2-Amino and 2-Halothiazole Derivatives as High-Affinity Metabotropic Glutamate Receptor Subtype 5 Ligands and Potential Radioligands for in Vivo Imaging. *J. Med. Chem.* **2011**, *54*, 901–908.
(29) Garg, A.; Gerwien, N. J.; Fasting, C.; Charlton, A.; Hopkinson, M. N. Formal Insertion of Alkenes into C(sp)−F Bonds Mediated by Fluorine-Hydrogen Bonding. *Angew. Chem. Int. Ed.* **2023**, *62*, e202302860.
(30) Feofanov, M.; Förtsch, A.; Amsharov, K.; Akhmetov, V. Solid-State Construction of Zigzag Periphery via Intramolecular C–H Insertion Induced by Alumina-Mediated C–F Activation. *Chem. Commun.* **2021**, *57*, 12325–12328.
(31) Zhang, W.; Zhu, L.; Hu, J. Electrophilic Monofluoromethylation of *O*-, *S*-, and *N*-Nucleophiles with Chlorofluoromethane. *Tetrahedron* **2007**, *63*, 10569–10575.
(32) Blessley, G.; Holden, P.; Walker, M.; Brown, J. M.; Gouverneur, V. Palladium-Catalyzed Substitution and Cross-Coupling of Benzylic Fluorides. *Org. Lett.* **2012**, *14*, 2754–2757.
(33) Zheng, Y.; Xie, Z.-Z.; He, X.-C.; Chen, Y.-S.; Cheng, W.-S.; Chen, K.; Xiang, H.-Y.; Chen, X.-Q.; Yang, H. Phosphonium Ylide-Mediated Programmable Fluorination to Access Mono- and Difluoromethylarenes. *Org. Lett.* **2021**, *23*, 2538–2542.
(34) An, L.; Xiao, Y.-L.; Min, Q.-Q.; Zhang, X. Facile Access to Fluoromethylated Arenes by Nickel-Catalyzed Cross-Coupling between Arylboronic Acids and Fluoromethyl Bromide. *Angew. Chem. Int. Ed.* **2015**, *54*, 9079–9083.
(35) Min, S.-Y.; Song, H.-X.; Yan, S.-S.; Yuan, R.; Ye, J.-H.; Wang, B.-Q.; Gui, Y.-Y.; Yu, D.-G. Photocatalytic Defluorocarboxylation Using Formate Salts as Both a Reductant and a Carbon Dioxide Source. *Green Chem.* **2023**, *25*, 6194–6199.

(36) Yu, Q.; Zhou, D.; Ma, J.; Song, C. Decarboxylative Nucleophilic Fluorination of Aliphatic Carboxylic Acids. *Org. Lett.* **2024**, *26*, 4257–4261.
(37) Blackaby, W. P.; De, K. M.; Enthoven, M.; Hinchliffe, P. S.; Paulie, C.; Timmers, C. M.; Verkaik, S. Fsh Receptor Antagonists. WO2013041461A1, 2013.
(38) Shapiro, G. 3,3-Difluoro-Piperidine Derivatives as Nr2b Nmda Receptor Antagonists. WO2016126869A1, 2016.
(39) Deligny, M.; Crosignani, S.; Houthuys, E. J. K. H.; Rowley, M. Heterocyclic Compounds as Ent Inhibitors and Compounds for Use in the Treatment of Cancers. WO2024134541A1, 2024.
(40) Ma, J.; Yi, W.; Lu, G.; Cai, C. Transition-Metal-Free C–H Oxidative Activation: Persulfate-Promoted Selective Benzylic Mono- and Difluorination. *Org. Biomol. Chem.* **2015**, *13*, 2890–2894.
(41) Yuan, D.; Liu, S.; Li, S.; Liu, R.; Zhu, X. Design, Synthesis and Biological Evaluation of 7-Substituted-1,3-Diaminopyrrol[3,2-*f*]Quinazolines as Potential Antibacterial Agents. *ChemMedChem* **2023**, *18*, e202300078.
(42) Yin, H.; Sheng, J.; Zhang, K.-F.; Zhang, Z.-Q.; Bian, K.-J.; Wang, X.-S. Nickel-Catalyzed Monofluoromethylation of (Hetero)Aryl Bromides via Reductive Cross-Coupling. *Chem. Commun.* **2019**, *55*, 7635–7638.
(43) Sirvinskaite, G.; Nardo, C. S.; Müller, P.; Gasser, A. C.; Morandi, B. Direct Synthesis of Unprotected Indolines through Intramolecular sp^3 C–H Amination Using Nitroarenes as Aryl Nitrene Precursors. *Chem. Eur. J.* **2023**, *29*, e202301978.
(44) Dong, C.; Bai, J.; Lv, X.-L.; Wu, W.; Lv, J.; Li, J.-R. Fixing Flexible Arms of Core-Shared Ligands to Enhance the Stability of Metal–Organic Frameworks. *Inorg. Chem.* **2019**, *58*, 15909–15916.
(45) Ramkumar, N.; Plantus, K.; Ozola, M.; Mishnev, A.; Nikolajeva, V.; Senkovs, M.; Ošeka, M.; Veliks, J. Photoredox-Catalyzed Direct C–H Monofluoromethylation of Heteroarenes. *New J. Chem.* **2023**, *47*, 20642–20652.
(46) Zhao, S.; Guo, Y.; Su, Z.; Cao, W.; Wu, C.; Chen, Q.-Y. A Series of Deoxyfluorination Reagents Featuring OCF_2 Functional Groups. *Org. Lett.* **2020**, *22*, 8634–8637.
(47) Palkowitz, M. D.; Laudadio, G.; Kolb, S.; Choi, J.; Oderinde, M. S.; Ewing, T. E.-H.; Bolduc, P. N.; Chen, T.; Zhang, H.; Cheng, P. T. W.; Zhang, B.; Mandler, M. D.; Blasczak, V. D.; Richter, J. M.; Collins, M. R.; Schioldager, R. L.; Bravo, M.; Dhar, T. G. M.; Vokits, B.; Zhu, Y.; Echeverria, P.-G.; Poss, M. A.; Shaw, S. A.; Clementson, S.; Petersen, N. N.; Mykhailiuk, P. K.; Baran, P. S. Overcoming Limitations in Decarboxylative Arylation via Ag–Ni Electrocatalysis. *J. Am. Chem. Soc.* **2022**, *144*, 17709–17720.
(48) Braun, M.-G.; Katcher, M. H.; Doyle, A. G. Carbofluorination via a Palladium-Catalyzed Cascade Reaction. *Chem. Sci.* **2013**, *4*, 1216–1220.
(49) Dimagno, S. G. Stabilization of Radioiodinated and Astatinated Pharmaceuticals and Imaging Agents. US2014275539A1, 2014.
(50) Kim, D. J.; Kang, Y. K.; Keum, G. C.; Kim, Y. K.; Ryu, H.; Lim, S.; Kim, D.; Lee, D. S.; Lee, Y. S.; Choi, Y.; Lee, J. H. Compounds with High Selectivity to Tau Aggregates Tau-Targeting Probe Comprising the Same and Preparation Method Thereof. KR20190090448A, 2019.
(51) Li, G.; Qin, Z.; Radosevich, A. T. P(III)/P(V)-Catalyzed Methylamination of Arylboronic Acids and Esters: Reductive C–N Coupling with Nitromethane as a Methylamine Surrogate. *J. Am. Chem. Soc.* **2020**, *142*, 16205–16210.
(52) Wangngae, S.; Siriwibool, S.; Chansaenpak, K.; Wet-osot, S.; Lai, R.-Y.; Kamkaew, A. Near-Infrared Fluorescent Heptamethine Cyanine Dyes for COX-2 Targeted Photodynamic Cancer Therapy. *ChemMedChem* **2022**, *17*, e202100780.
(53) Scholtz, C.; Riley, D. L. Improved Batch and Flow Syntheses of the Nonsteroidal Anti-Inflammatory COX-2 Inhibitor Celecoxib. *React. Chem. Eng.* **2021**, *6*, 138–146.
(54) Zmuda, F.; Blair, A.; Liuzzi, M. C.; Malviya, G.; Chalmers, A. J.; Lewis, D.; Sutherland, A.; Pimlott, S. L. An ^{18}F-Labeled Poly(ADP-Ribose) Polymerase Positron Emission Tomography Imaging Agent. *J. Med. Chem.* **2018**, *61*, 4103–4114.

(55) Falcone, N. A.; He, S.; Hoskin, J. F.; Mangat, S.; Sorensen, E. J. N-Oxide-to-Carbon Transmutations of Azaarene N-Oxides. *Org. Lett.* **2024**, *26*, 4280–4285.
(56) Liu, Q.; Shen, X.; Ni, C.; Hu, J. Stereoselective Carbonyl Olefination with Fluorosulfoximines: Facile Access to Z or E Terminal Monofluoroalkenes. *Angew. Chem. Int. Ed.* **2017**, *56*, 619–623.
(57) Ehehalt, L. E.; Beleh, O. M.; Priest, I. C.; Mouat, J. M.; Olszewski, A. K.; Ahern, B. N.; Cruz, A. R.; Chi, B. K.; Castro, A. J.; Kang, K.; Wang, J.; Weix, D. J. Cross-Electrophile Coupling: Principles, Methods, and Applications in Synthesis. *Chem. Rev.* **2024**, *124*, 13397–13569.
(58) Biswas, S.; Weix, D. J. Mechanism and Selectivity in Nickel-Catalyzed Cross-Electrophile Coupling of Aryl Halides with Alkyl Halides. *J. Am. Chem. Soc.* **2013**, *135*, 16192–16197.
(59) Dong, W.; Badir, S. O.; Zhang, X.; Molander, G. A. Accessing Aliphatic Amines in C–C Cross-Couplings by Visible Light/Nickel Dual Catalysis. *Org. Lett.* **2021**, *23*, 4250–4255.

NMR Spectra

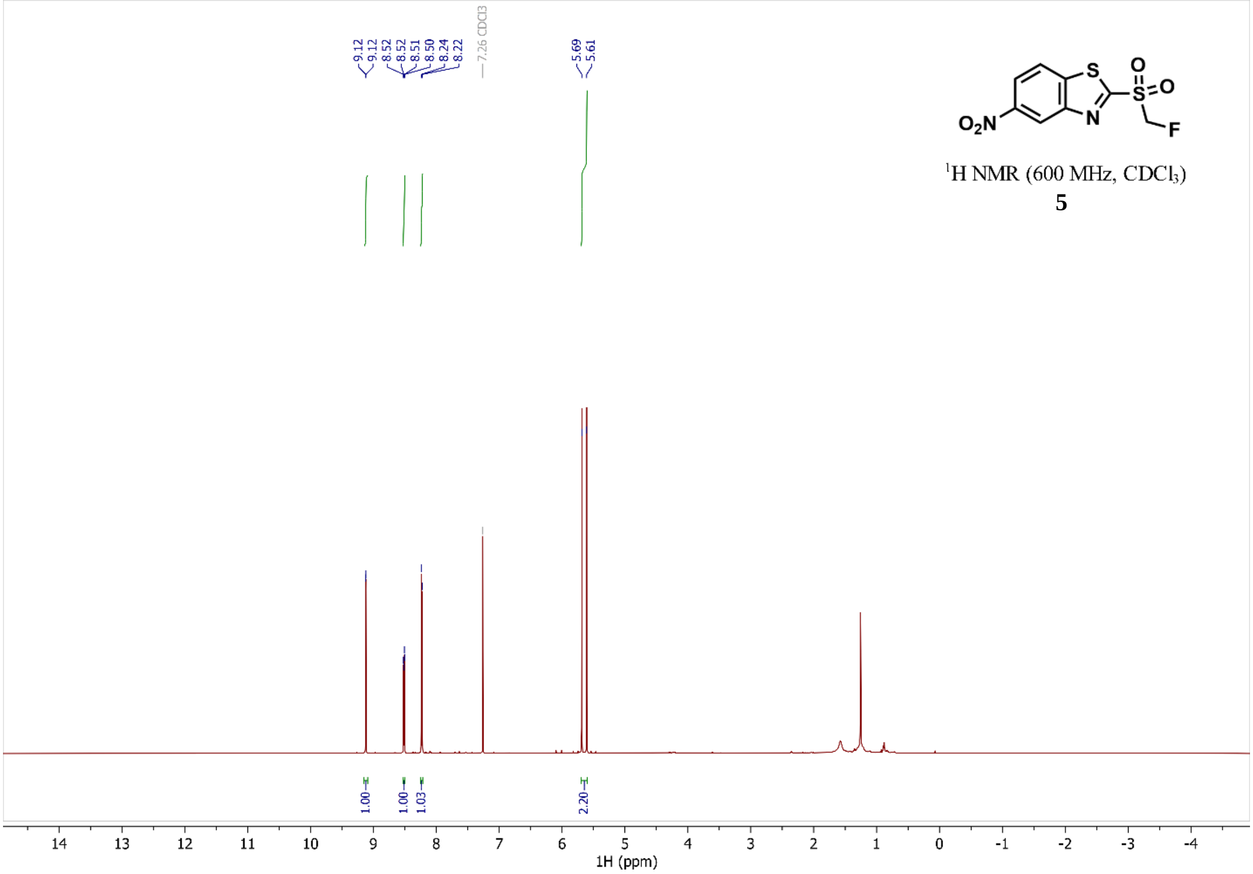
9.12
9.12
8.52
8.52
8.51
8.50
8.24
8.22
7.26 CDCl3
5.69
5.61
O2N
S
N
O
S
O
F
1H NMR (600 MHz, CDCl3)
5
1.00
1.00
1.03
2.20
1H (ppm)

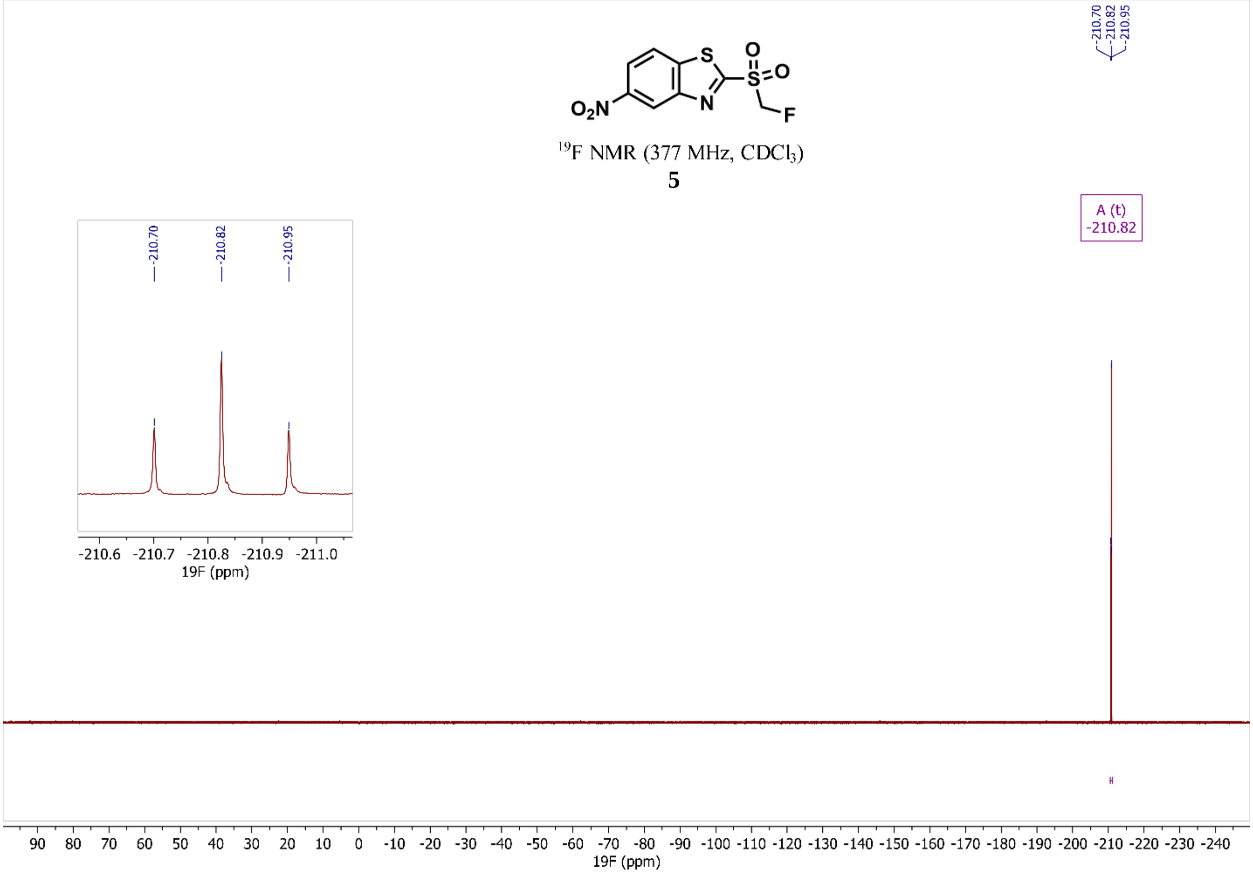
-210.70
-210.82
-210.95
O2N
S
N
O
S
O
F
19F NMR (377 MHz, CDCl3)
5
A (t)
-210.82
19F (ppm)

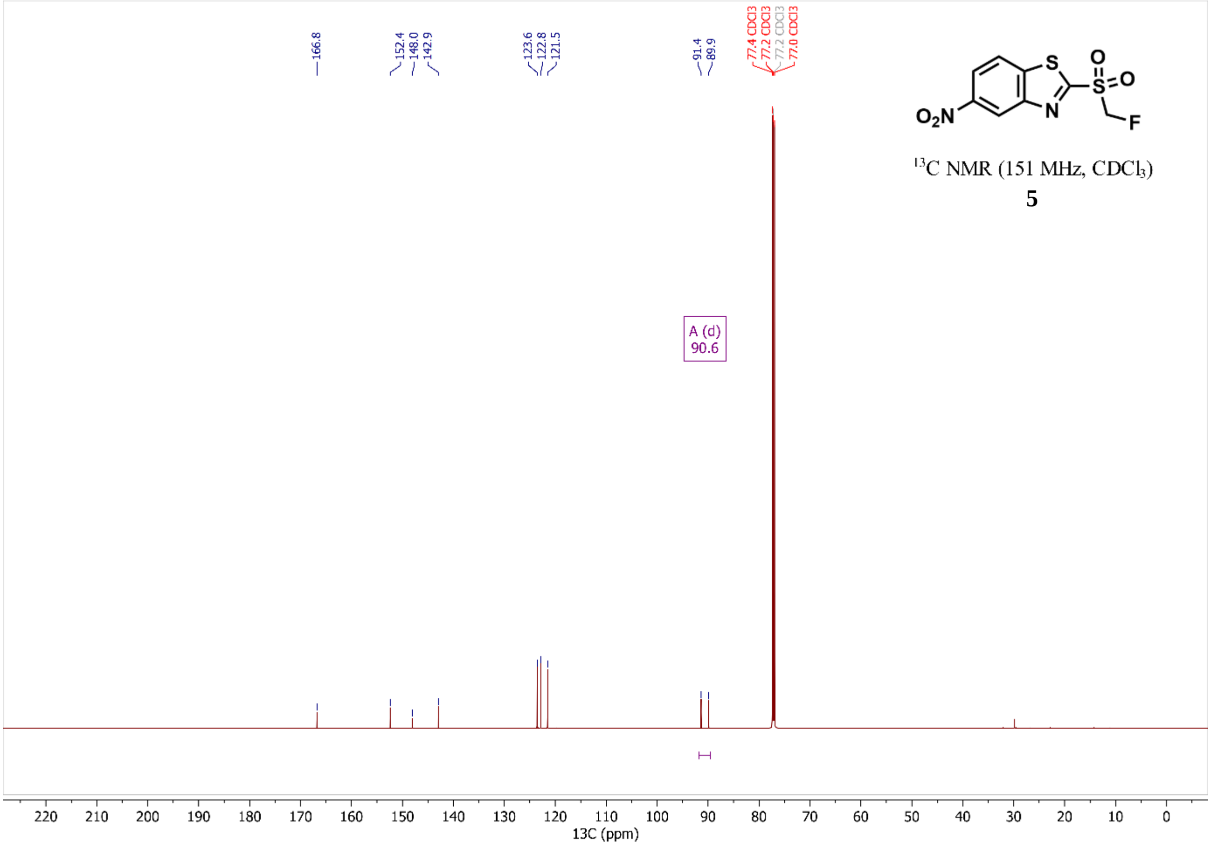

^{13}C NMR (151 MHz, $CDCl_3$)

5

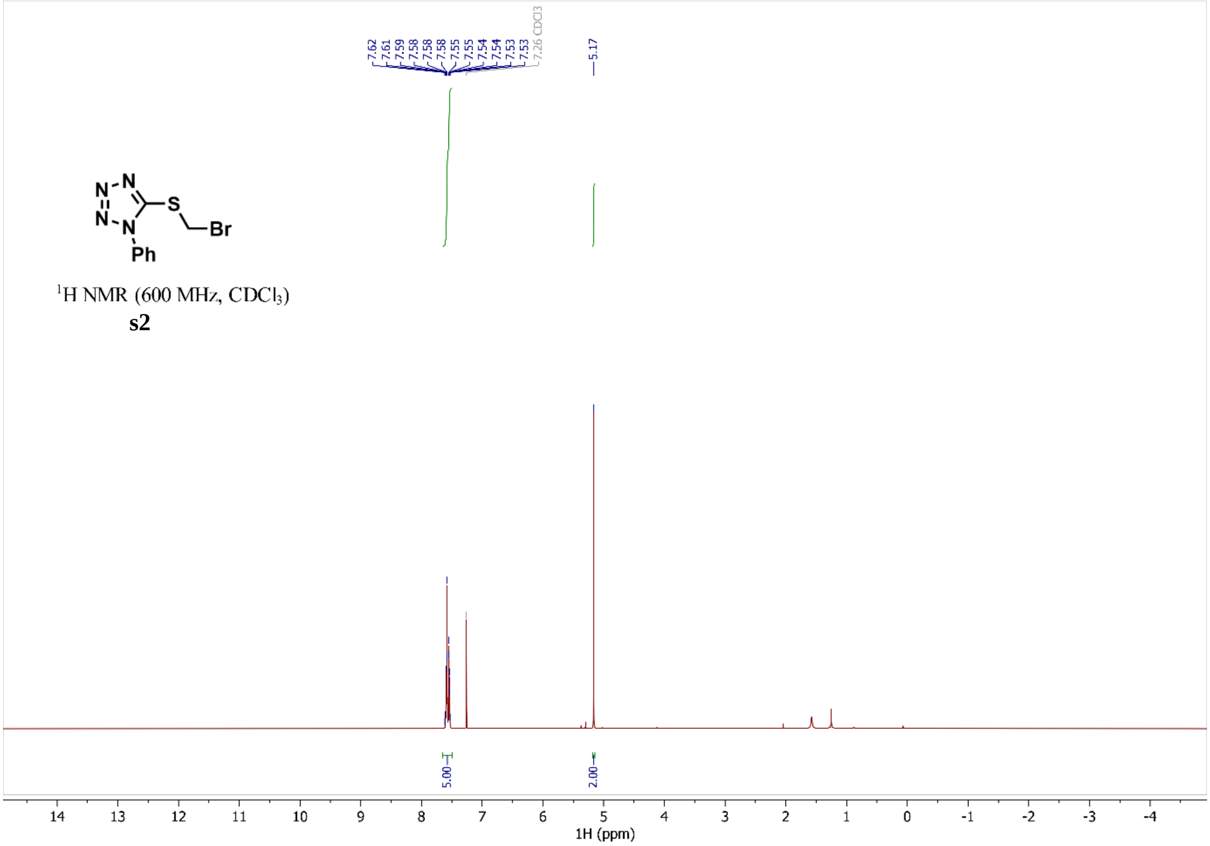

^{1}H NMR (600 MHz, $CDCl_3$)

s2

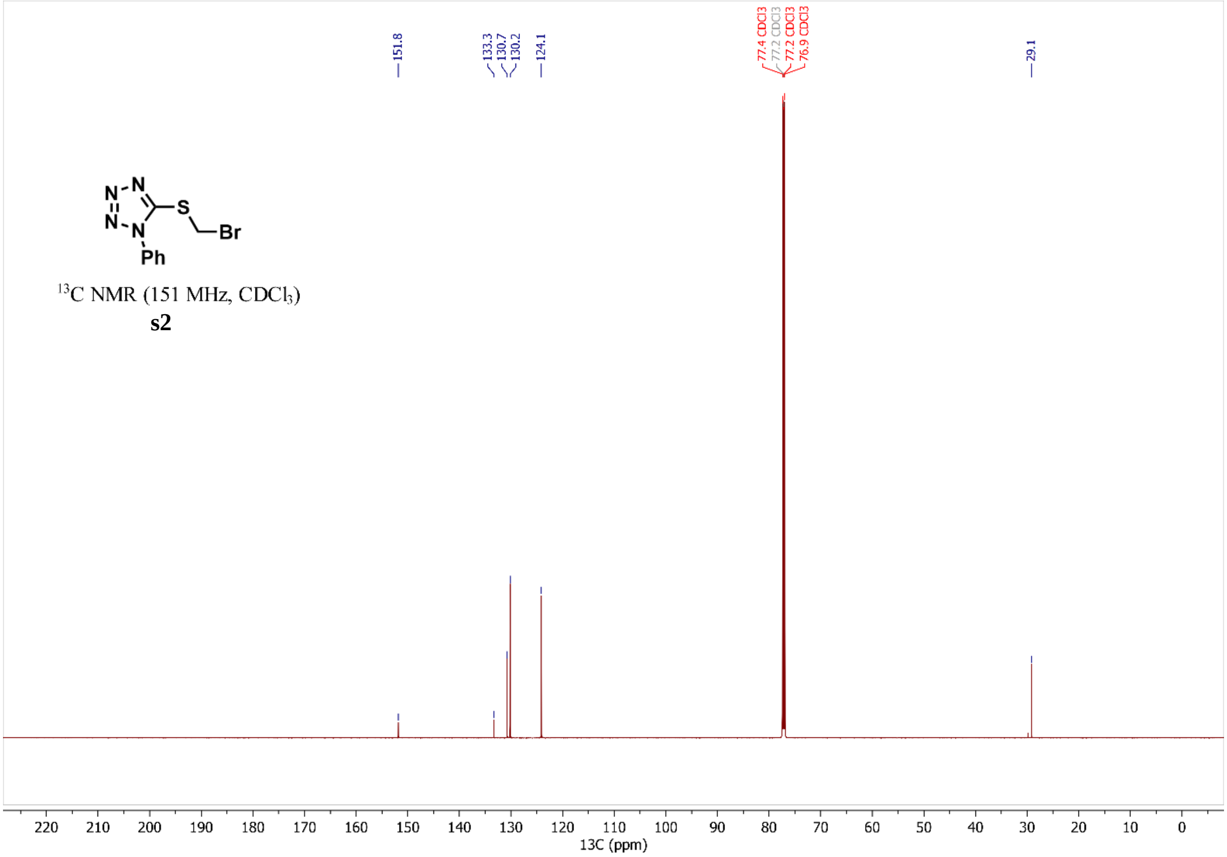

^{13}C NMR (151 MHz, $CDCl_3$)

s2

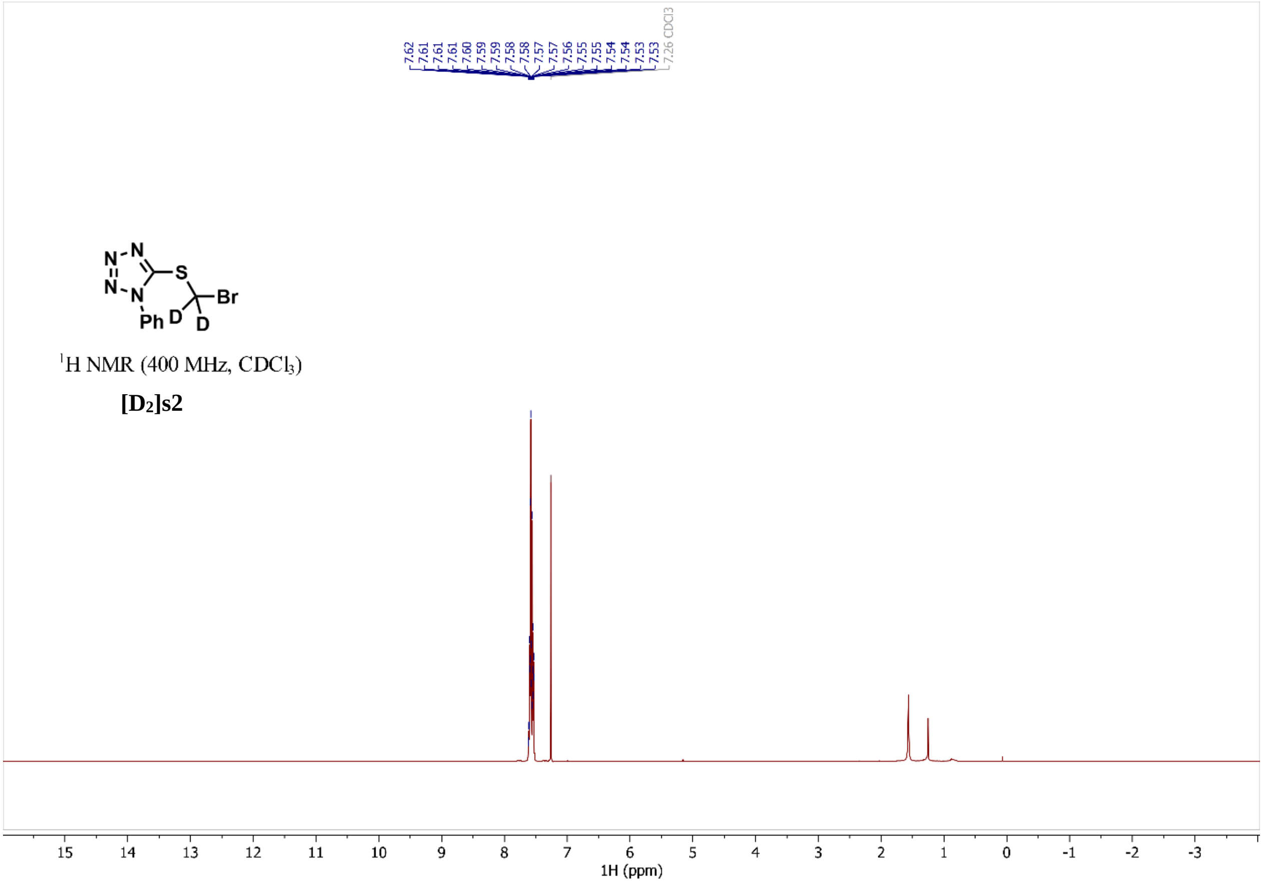

^{1}H NMR (400 MHz, $CDCl_3$)

[D_2]s2

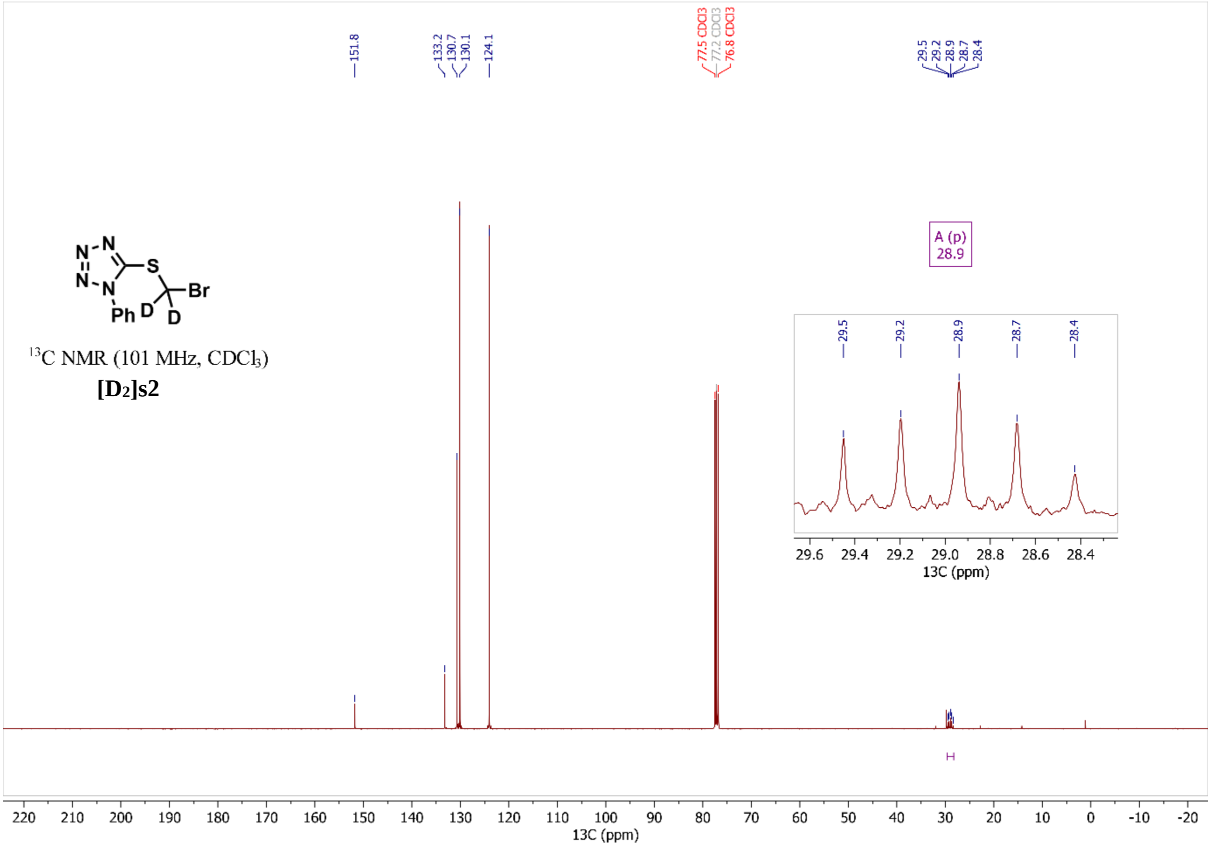

¹³C NMR (101 MHz, CDCl₃)
[D₂]s2
151.8
133.2
130.7
130.1
124.1
77.5 CDCl3
77.2 CDCl3
76.8 CDCl3
29.5
29.2
28.9
28.7
28.4
A (p)
28.9
13C (ppm)

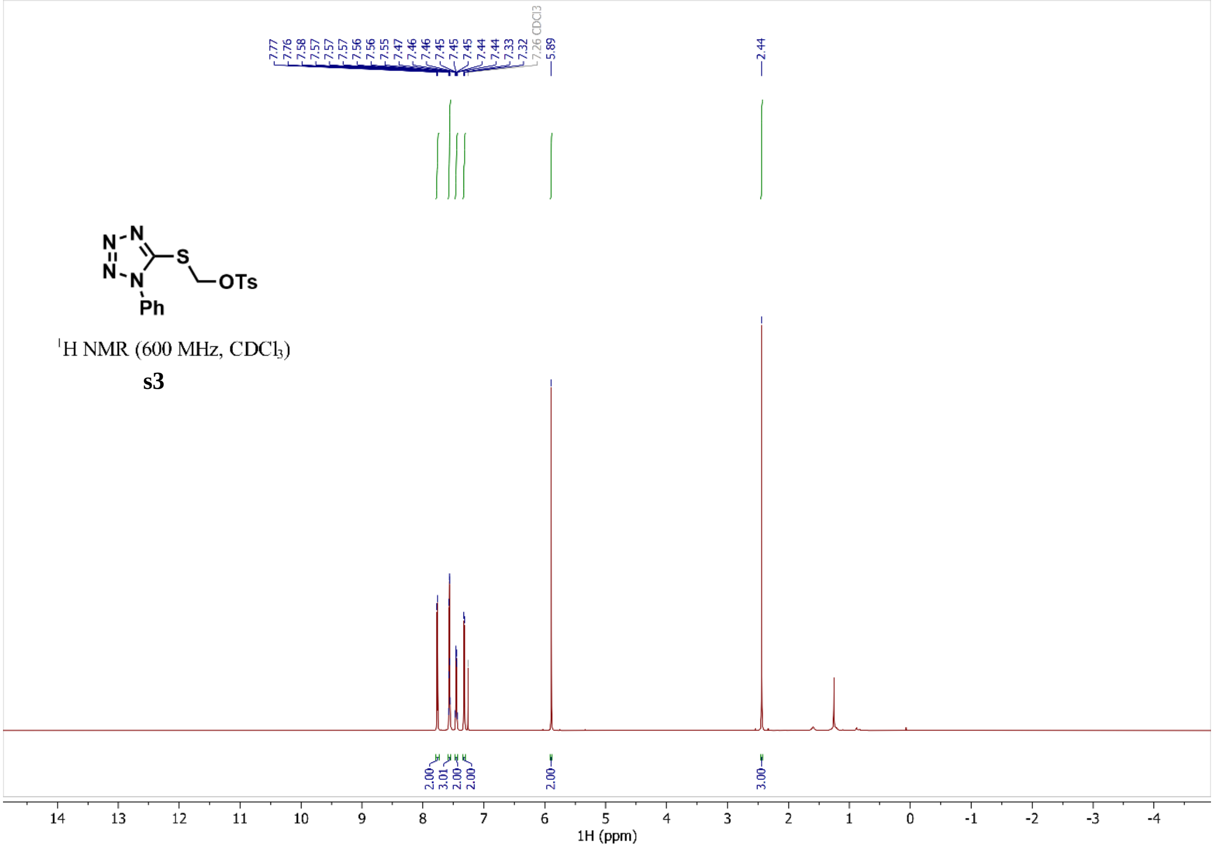

¹H NMR (600 MHz, CDCl₃)
s3
7.77
7.76
7.58
7.57
7.57
7.56
7.56
7.55
7.47
7.46
7.46
7.45
7.45
7.45
7.44
7.44
7.33
7.32
7.26 CDCl3
5.89
2.44
2.00
3.01
2.00
2.00
2.00
3.00
1H (ppm)

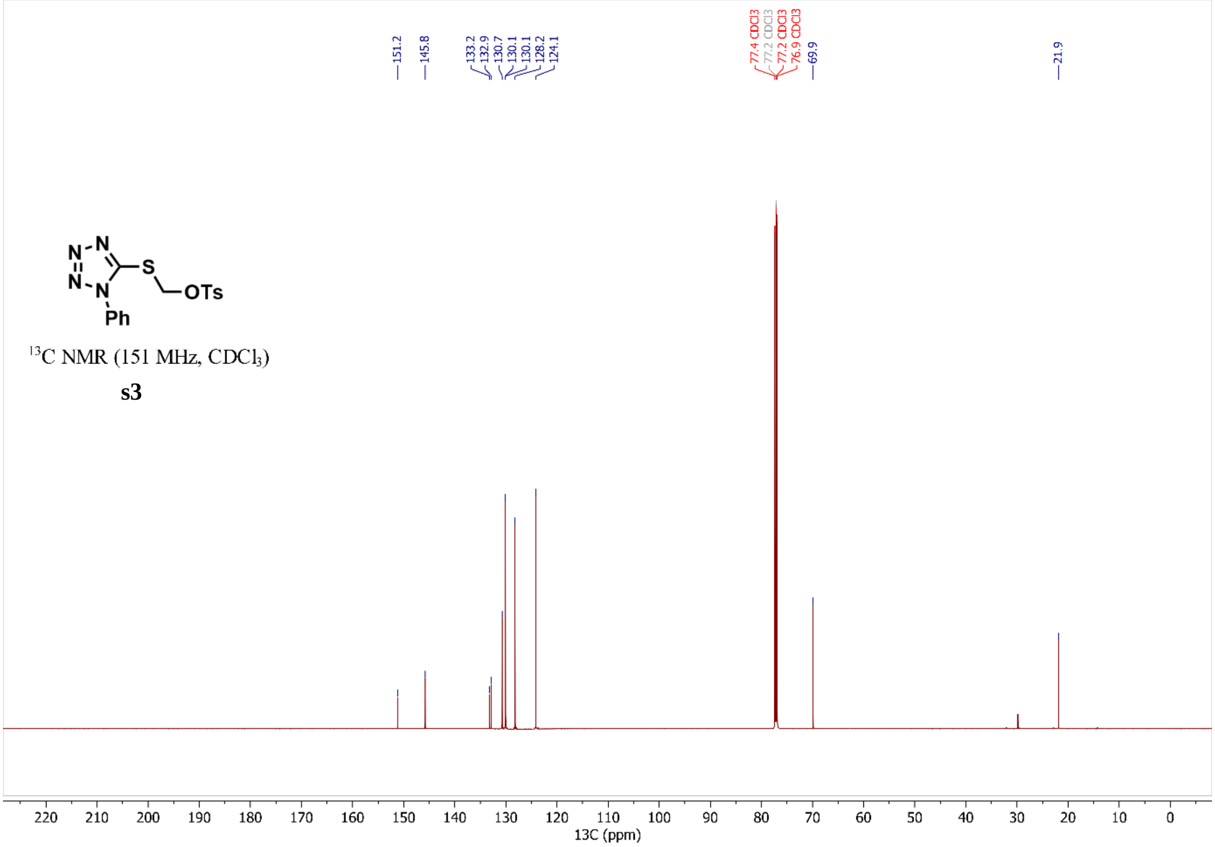

^{13}C NMR (151 MHz, $CDCl_3$)

s3

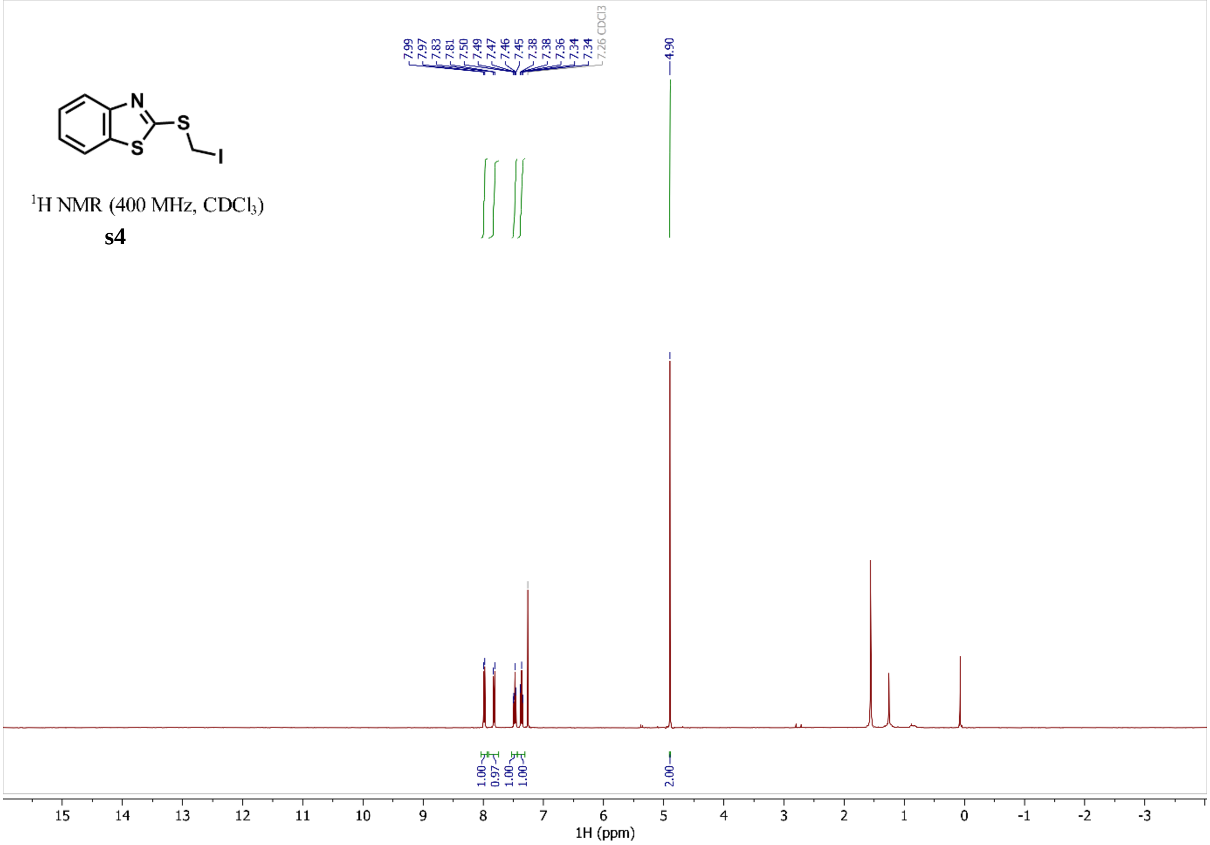

^{1}H NMR (400 MHz, $CDCl_3$)

s4

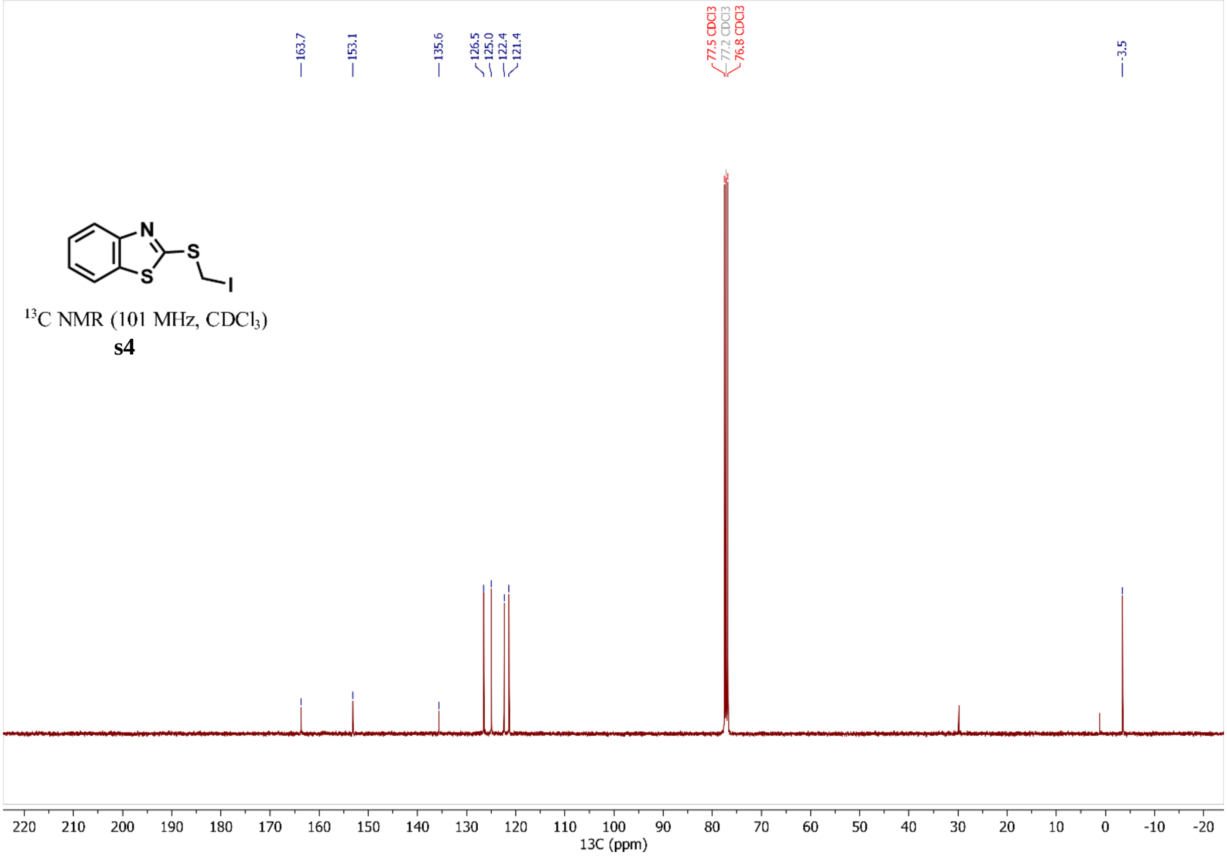
163.7
153.1
135.6
126.5
125.0
122.4
121.4
77.5 CDCl3
77.2 CDCl3
76.8 CDCl3
-3.5
N
S
S
I
13C NMR (101 MHz, CDCl3)
s4
220 210 200 190 180 170 160 150 140 130 120 110 100 90 80 70 60 50 40 30 20 10 0 -10 -20
13C (ppm)

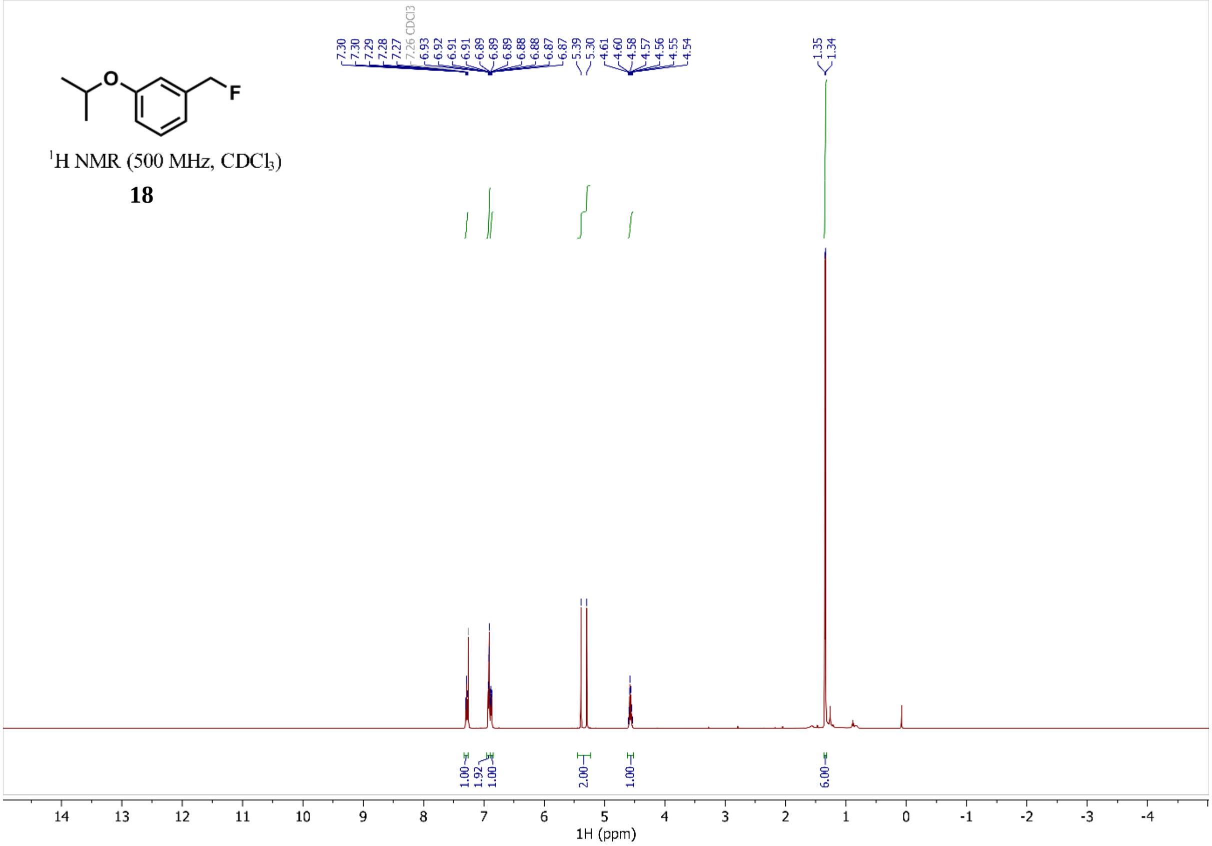
7.30
7.30
7.29
7.28
7.27
7.26 CDCl3
6.93
6.92
6.91
6.91
6.89
6.89
6.89
6.88
6.88
6.87
6.87
5.39
5.30
4.61
4.60
4.58
4.57
4.56
4.55
4.54
1.35
1.34
O
F
1H NMR (500 MHz, CDCl3)
18
1.00
1.92
1.00
2.00
1.00
6.00
14 13 12 11 10 9 8 7 6 5 4 3 2 1 0 -1 -2 -3 -4
1H (ppm)

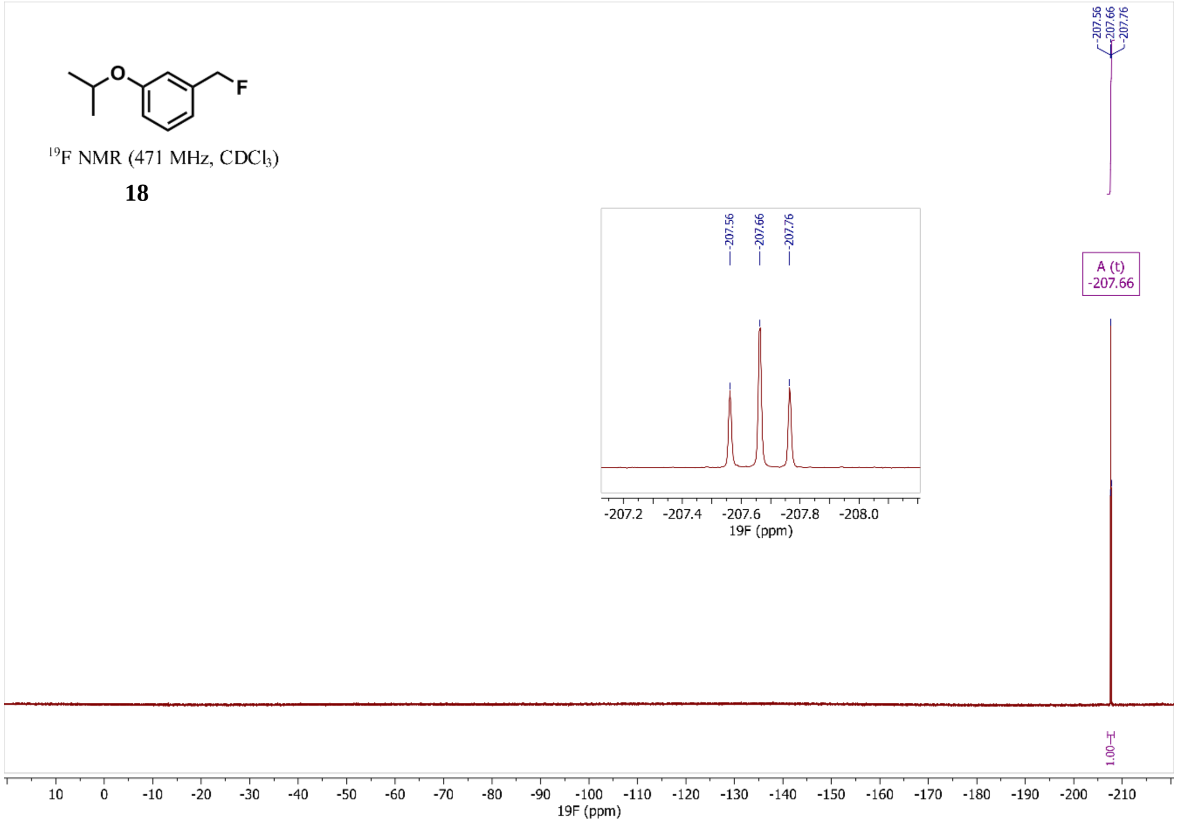

^{19}F NMR (471 MHz, CDCl$_3$)
18
F
O
-207.56
-207.66
-207.76
A (t)
-207.66
1.00
-207.2
-207.4
-207.6
-207.8
-208.0
19F (ppm)
10
0
-10
-20
-30
-40
-50
-60
-70
-80
-90
-100
-110
-120
-130
-140
-150
-160
-170
-180
-190
-200
-210
19F (ppm)

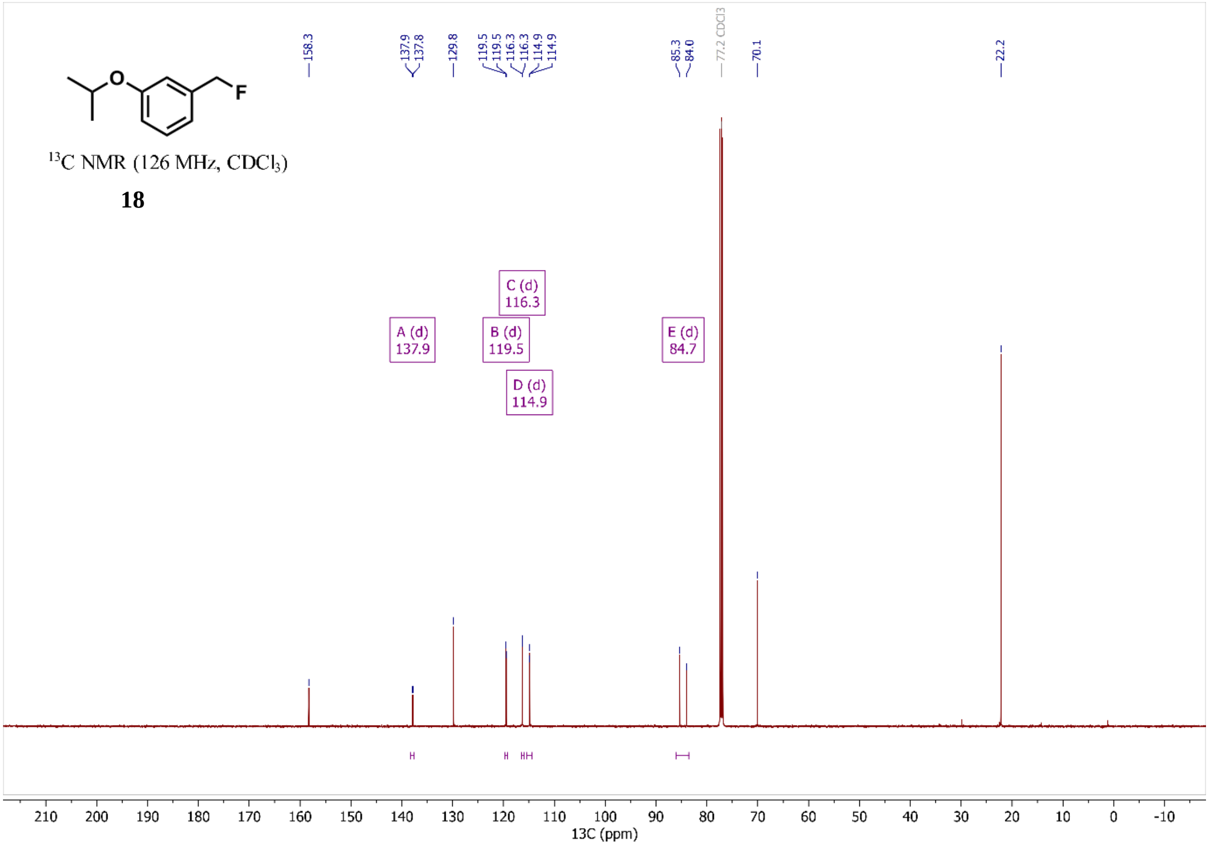

^{13}C NMR (126 MHz, CDCl$_3$)
18
F
O
158.3
137.9
137.8
129.8
119.5
119.5
116.3
116.3
114.9
114.9
85.3
84.0
77.2 CDCl3
70.1
22.2
A (d)
137.9
B (d)
119.5
C (d)
116.3
D (d)
114.9
E (d)
84.7
210
200
190
180
170
160
150
140
130
120
110
100
90
80
70
60
50
40
30
20
10
0
-10
13C (ppm)

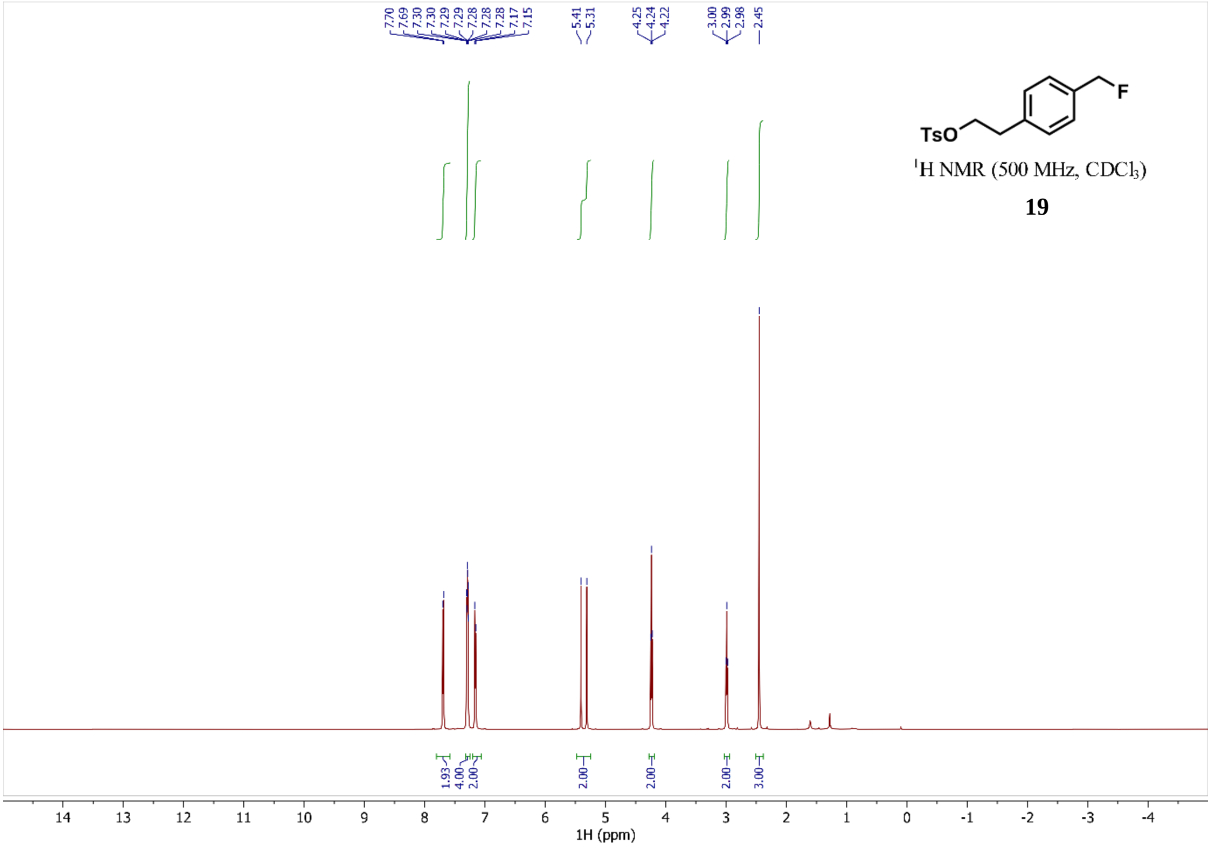
TsO
F
¹H NMR (500 MHz, CDCl₃)
19
1H (ppm)

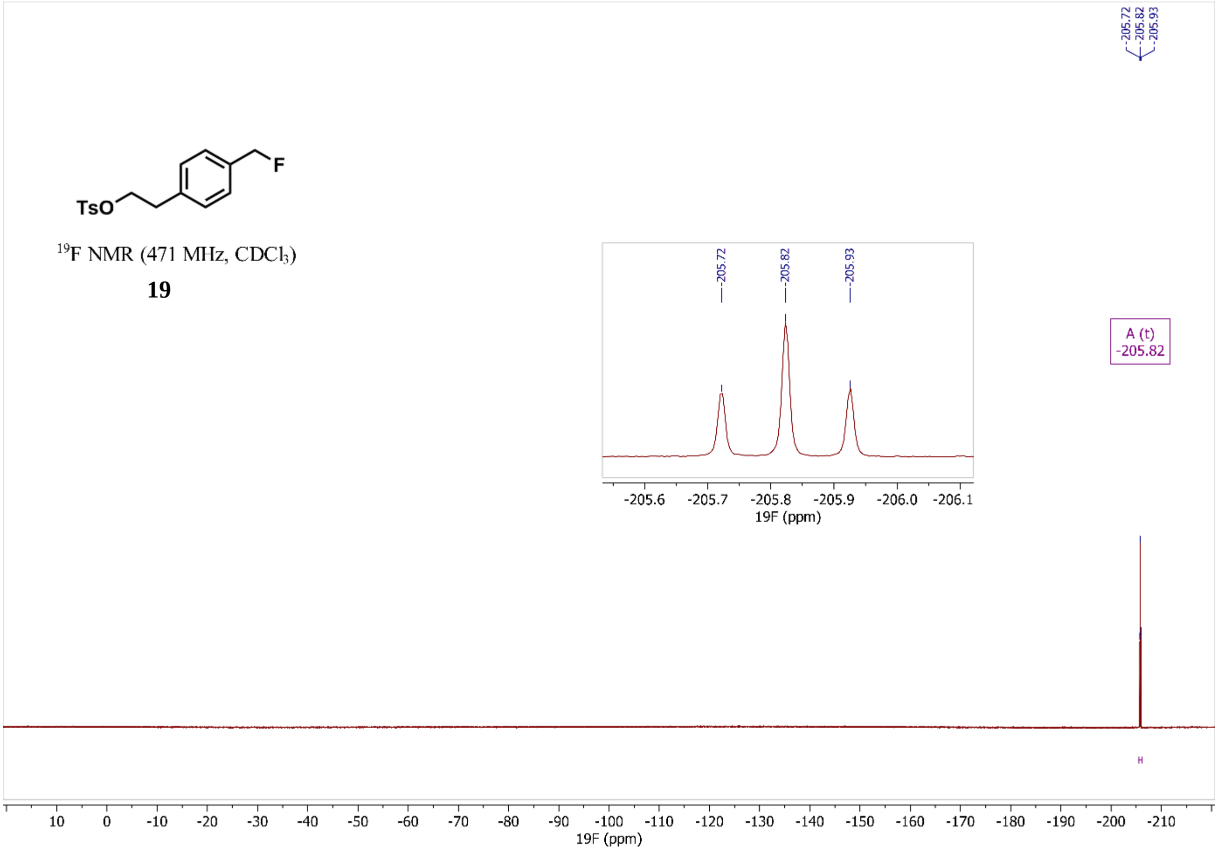
TsO
F
¹⁹F NMR (471 MHz, CDCl₃)
19
A (t)
-205.82
19F (ppm)

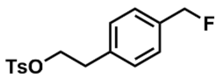

^{13}C NMR (126 MHz, $CDCl_3$)

19

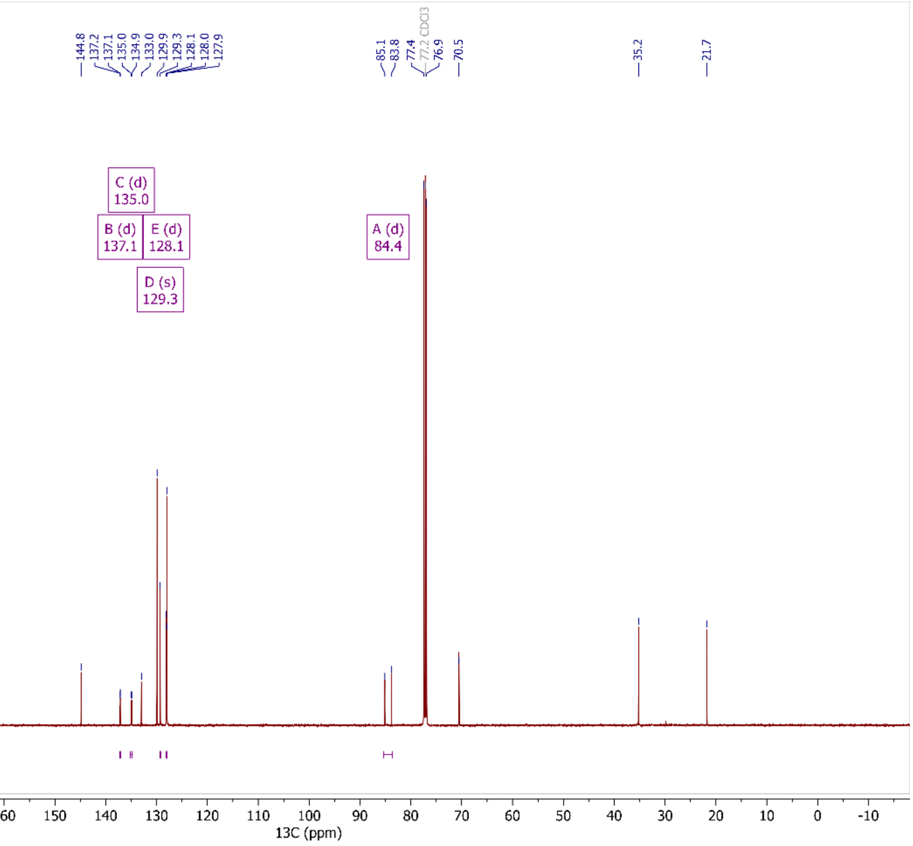

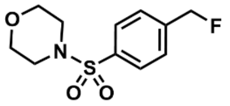

^{1}H NMR (500 MHz, $CDCl_3$)

22

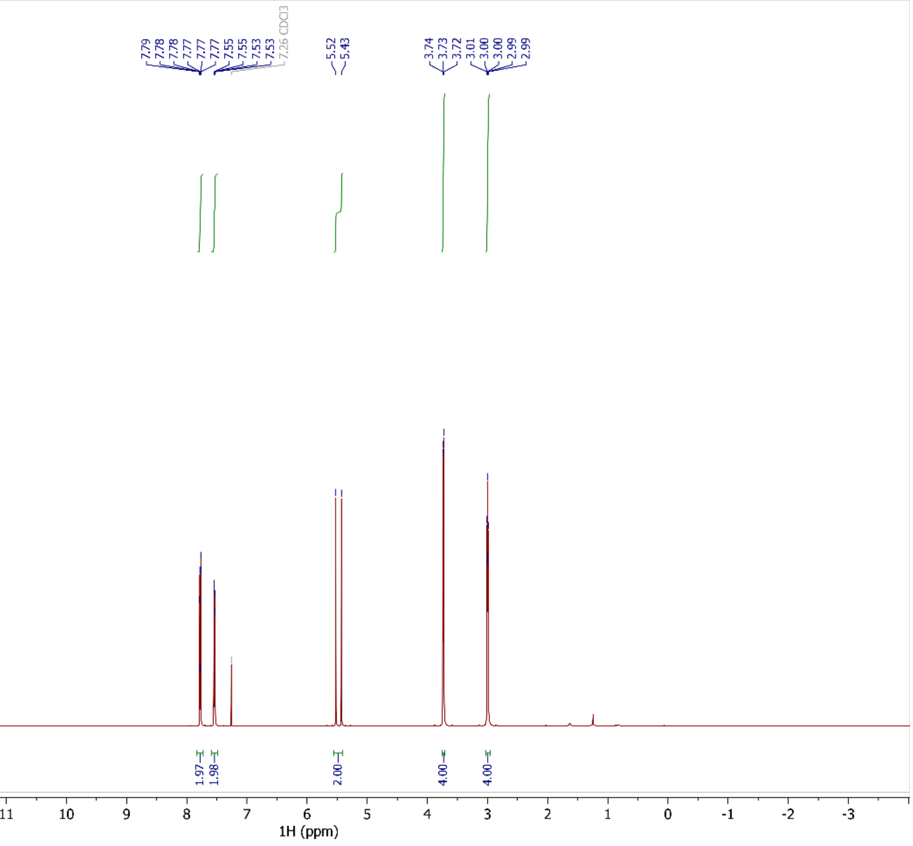

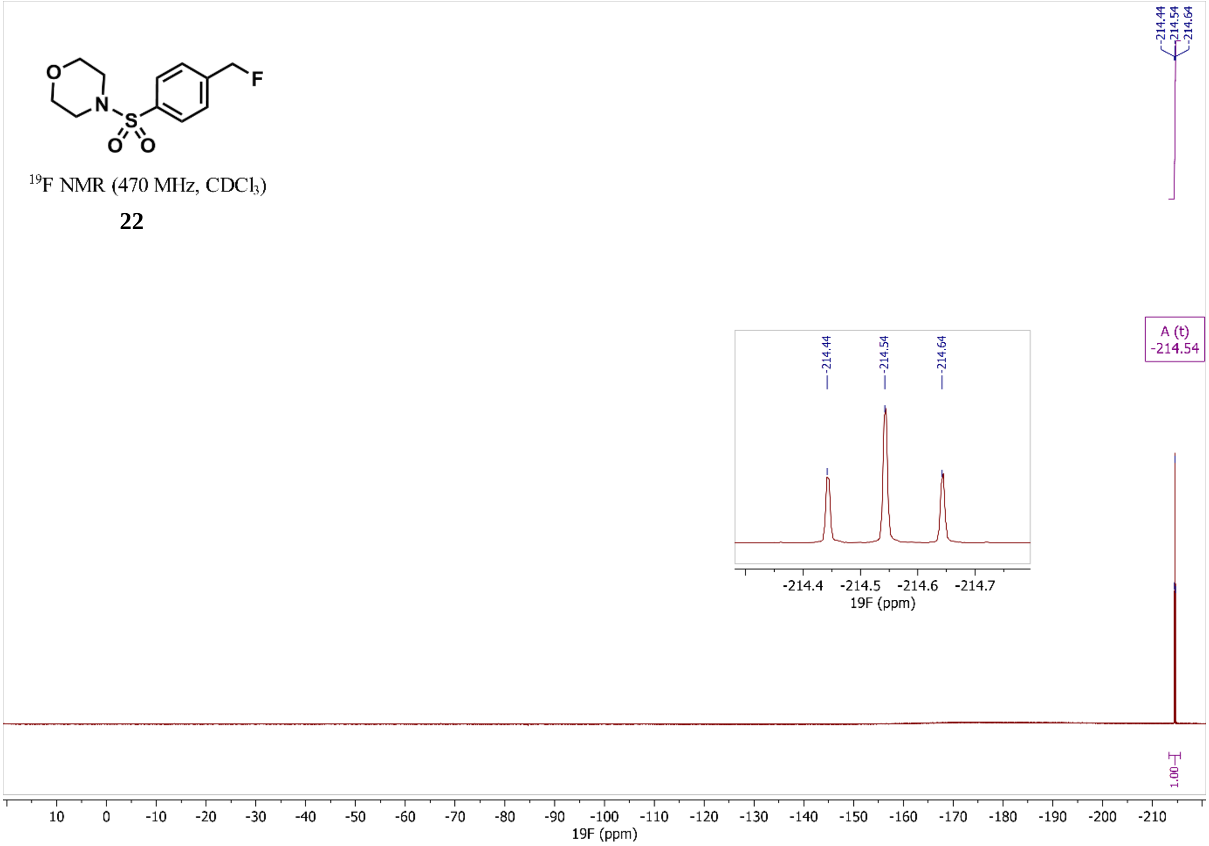

^{19}F NMR (470 MHz, CDCl$_3$)
22
-214.44
-214.54
-214.64
A (t)
-214.54
1.00
19F (ppm)

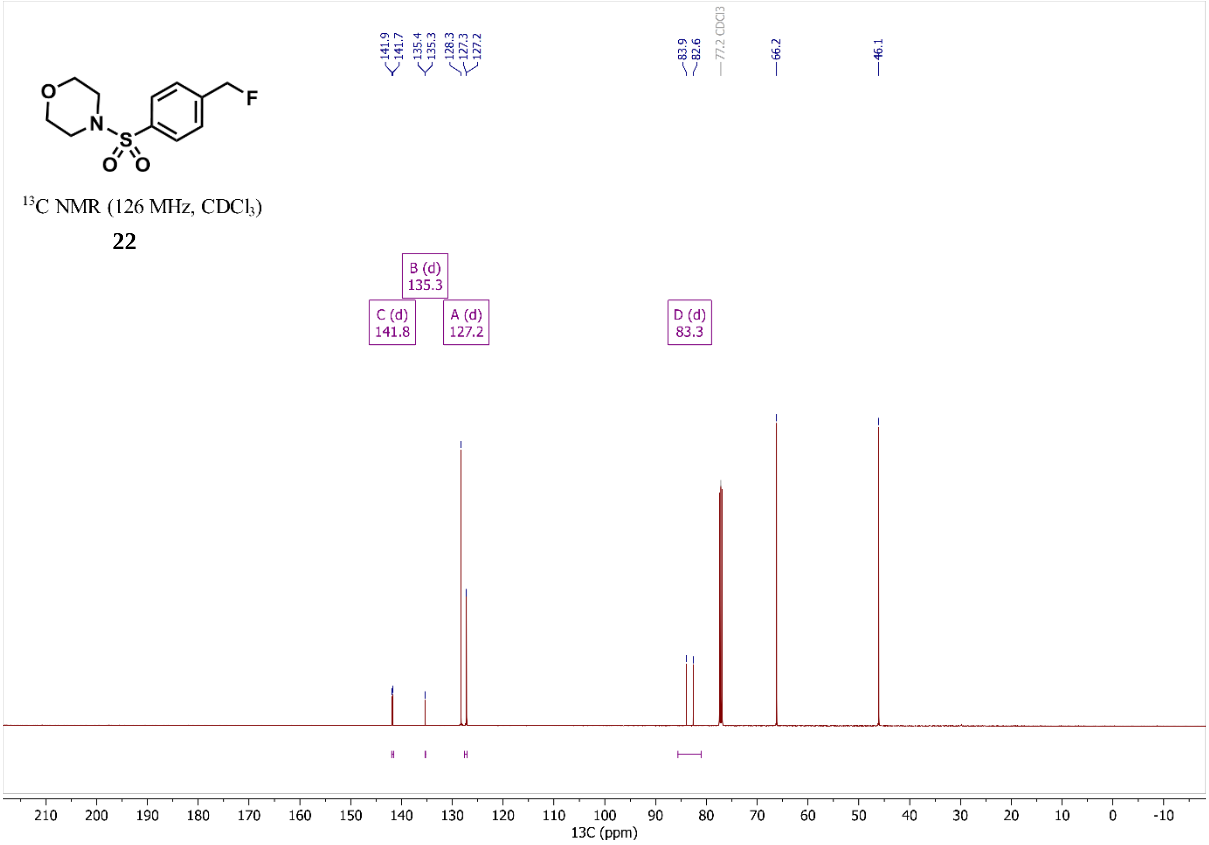

^{13}C NMR (126 MHz, CDCl$_3$)
22
141.9
141.7
135.4
135.3
128.3
127.3
127.2
83.9
82.6
77.2 CDCl3
66.2
46.1
B (d)
135.3
C (d)
141.8
A (d)
127.2
D (d)
83.3
13C (ppm)

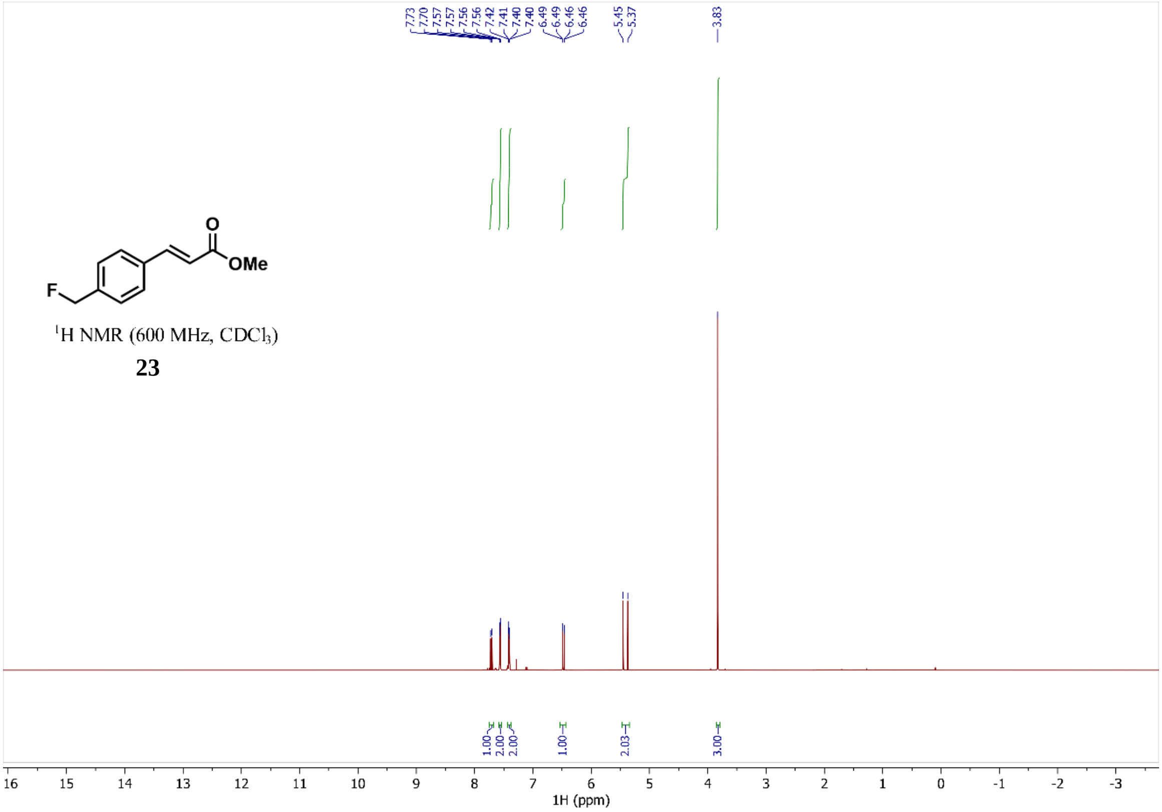

^{1}H NMR (600 MHz, $CDCl_3$)

23

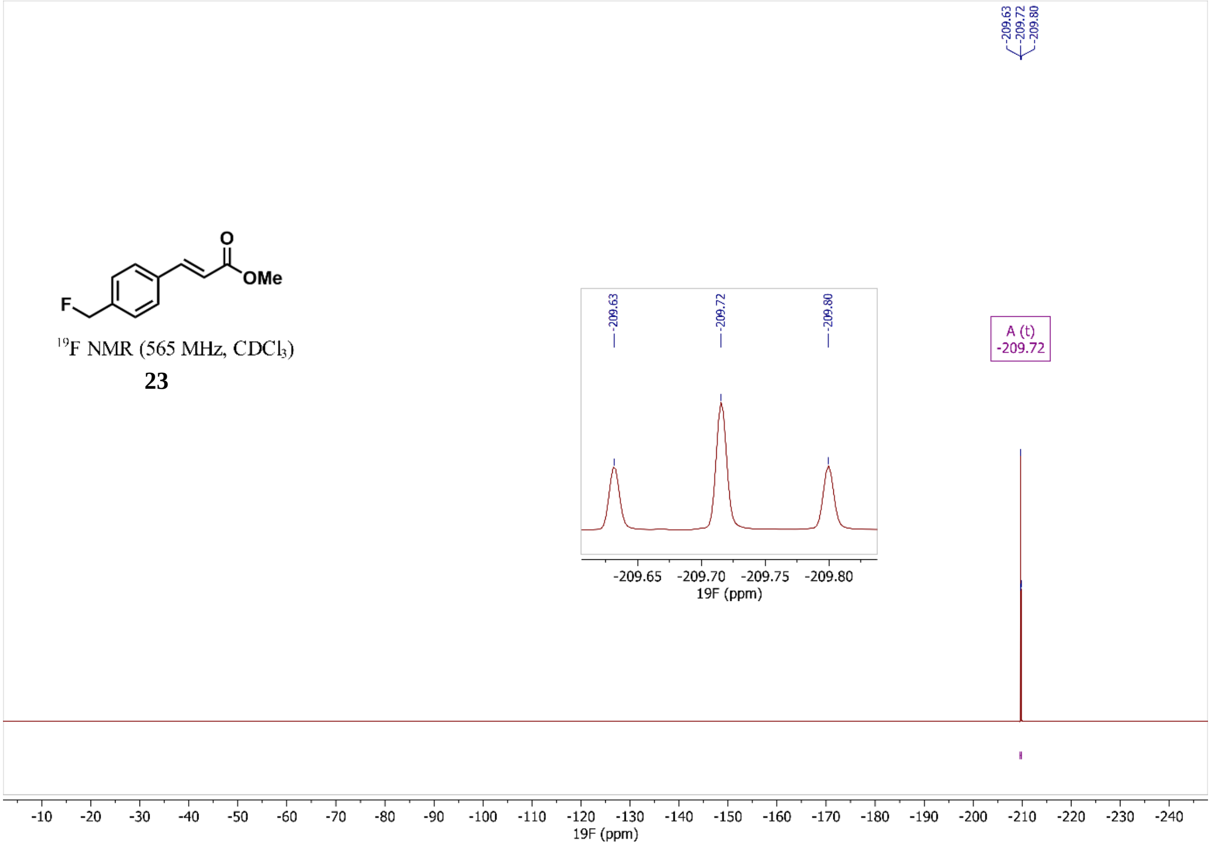

^{19}F NMR (565 MHz, $CDCl_3$)

23

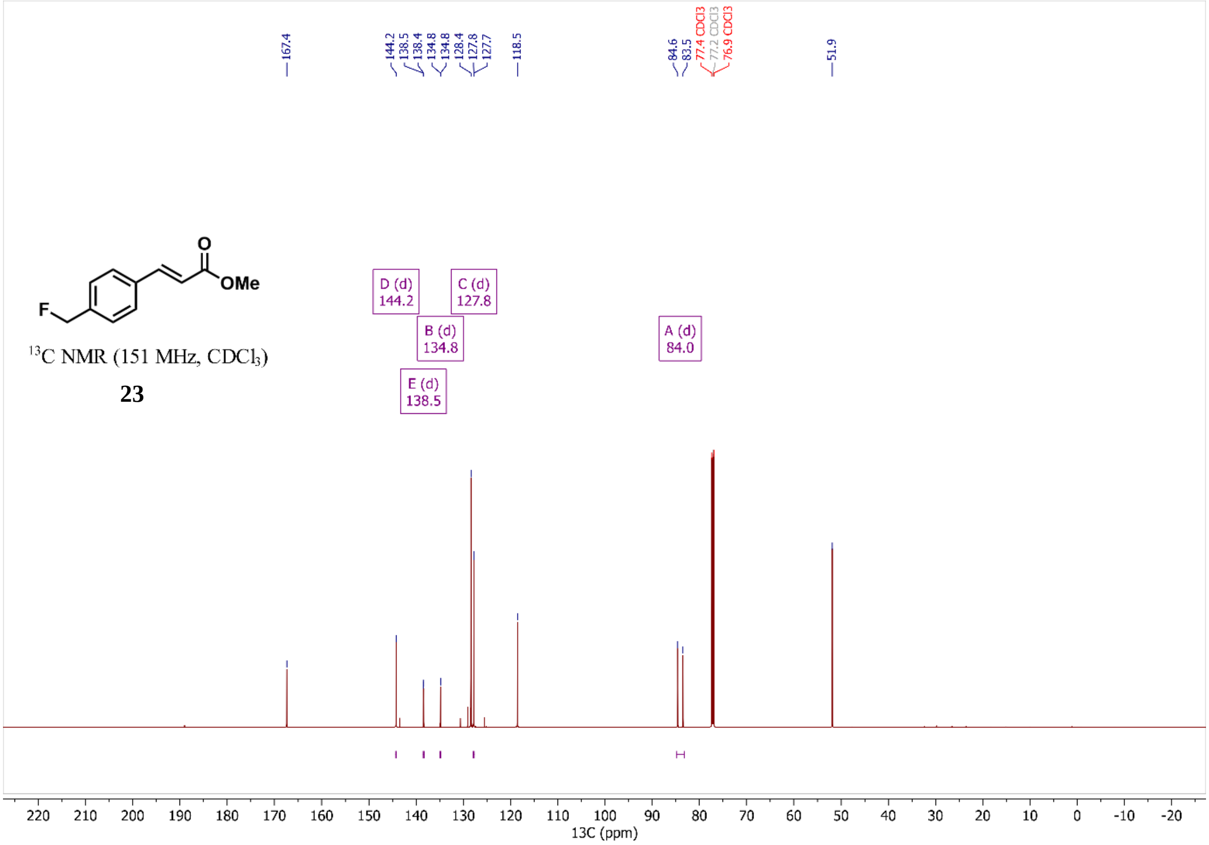

^{13}C NMR (151 MHz, $CDCl_3$)

23

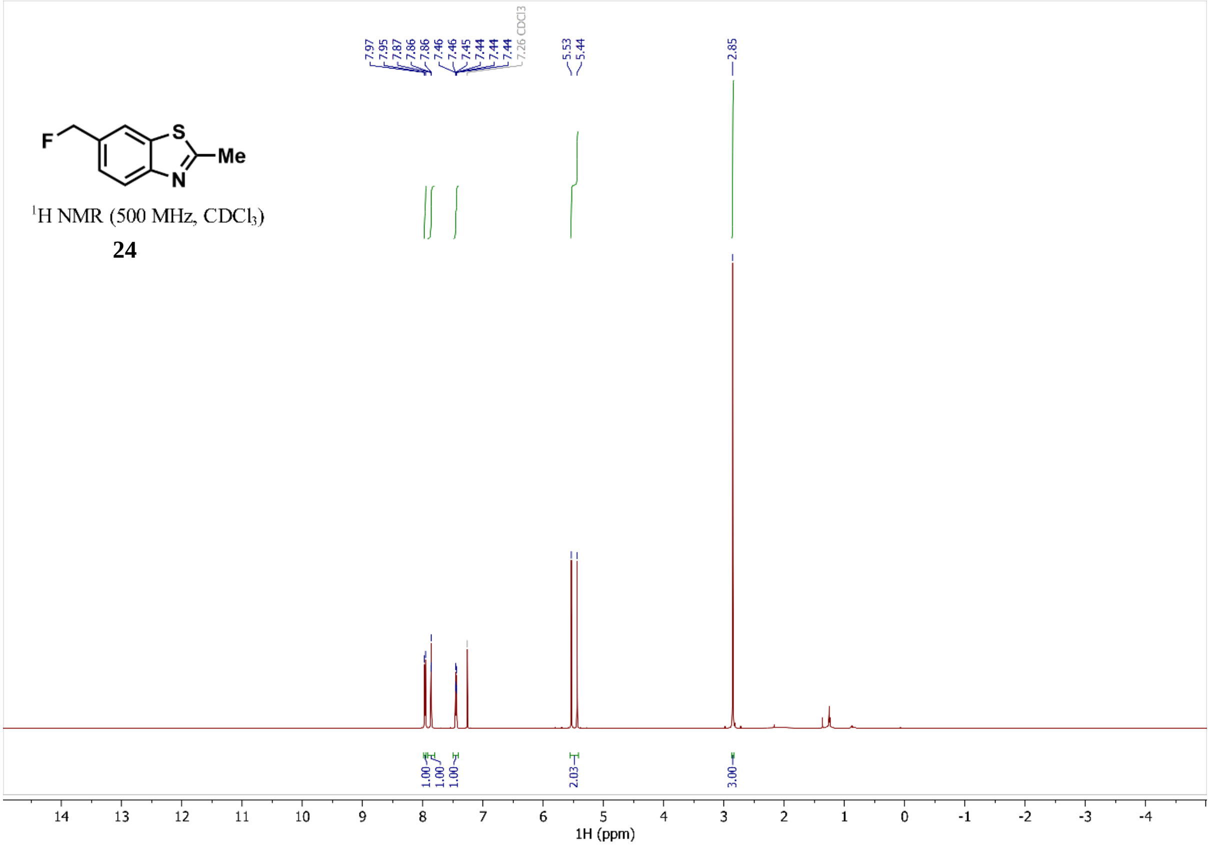

^{1}H NMR (500 MHz, $CDCl_3$)

24

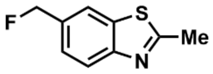

^{19}F NMR (471 MHz, $CDCl_3$)
24

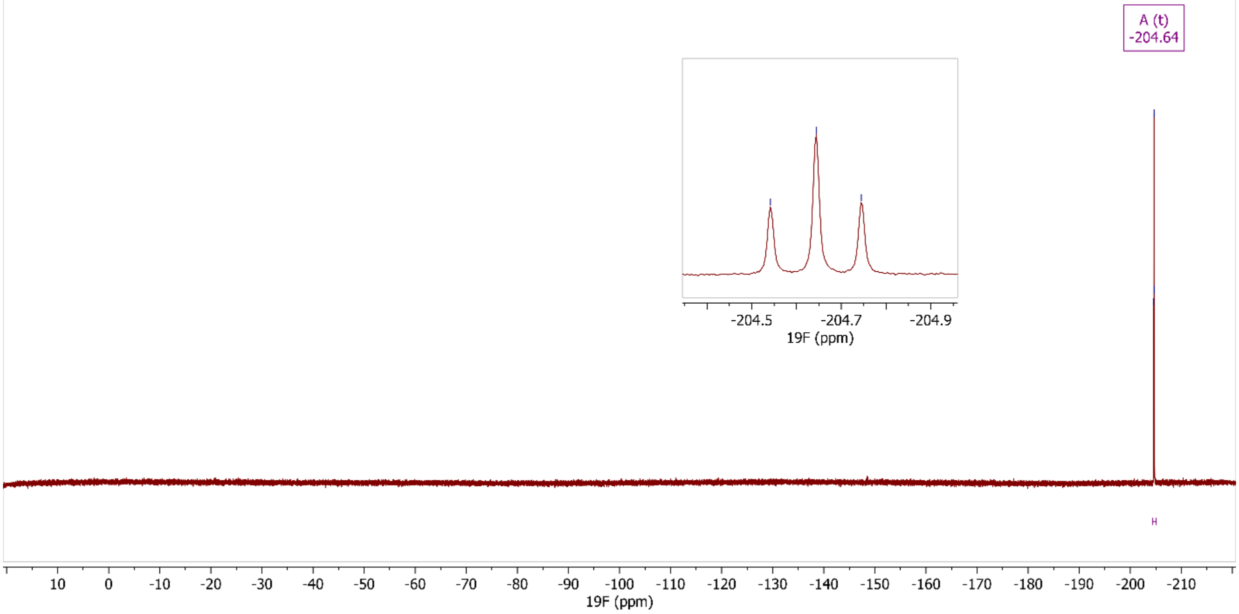

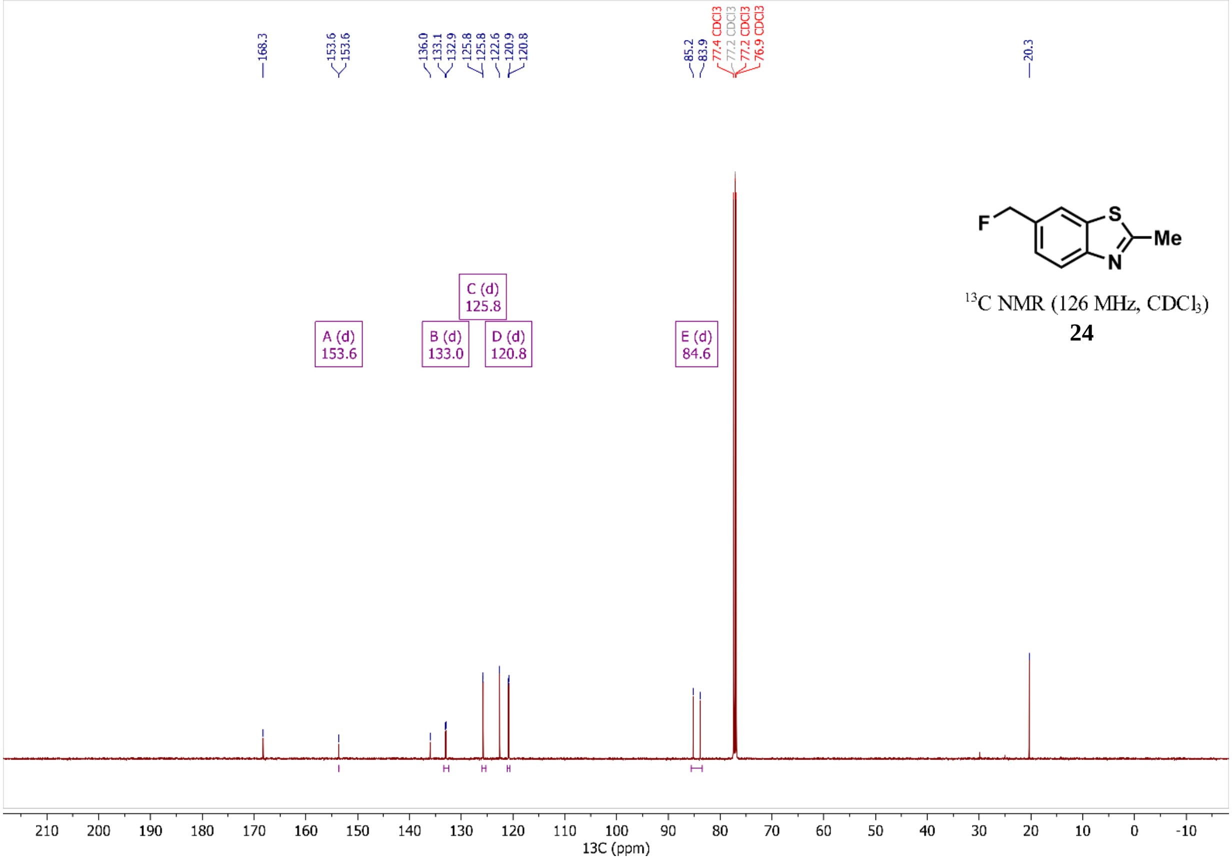

^{13}C NMR (126 MHz, $CDCl_3$)
24

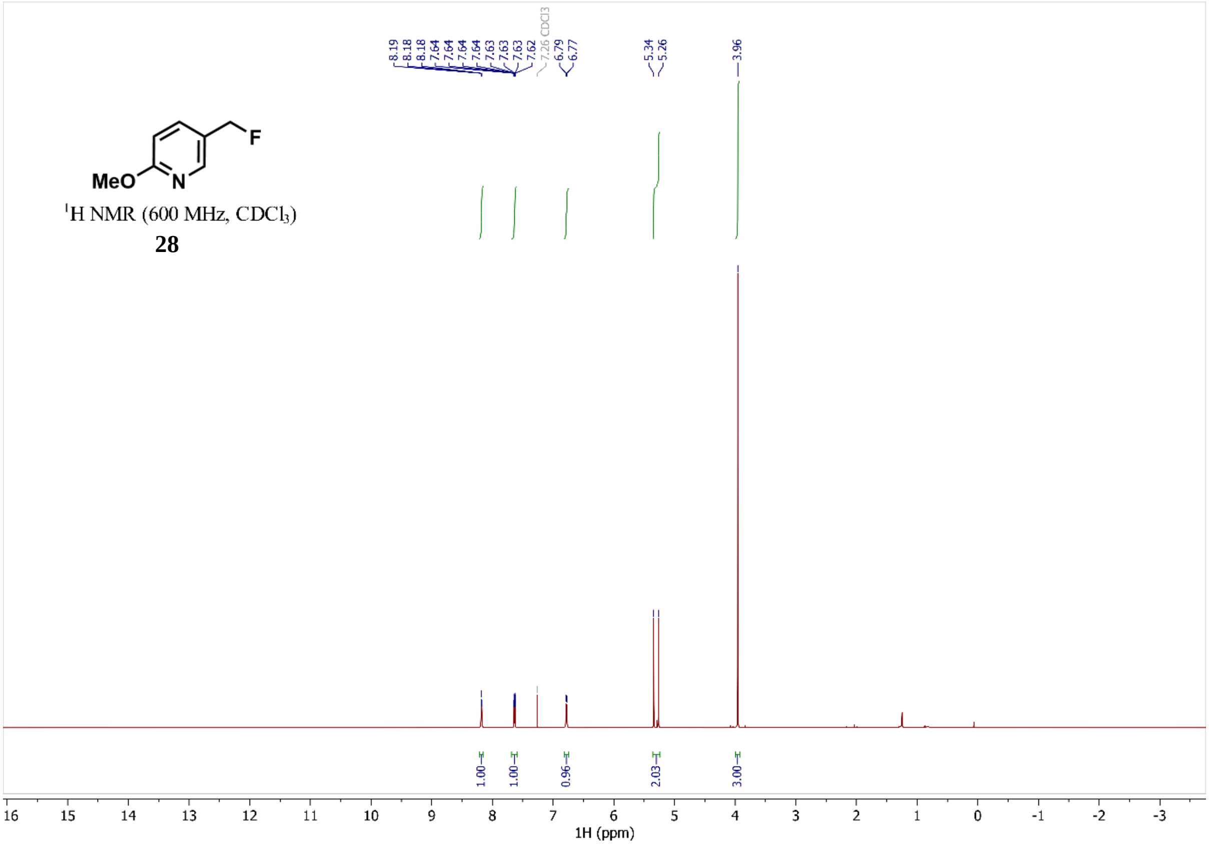

MeO
N
F
^{1}H NMR (600 MHz, $CDCl_3$)
28
8.19
8.18
8.18
7.64
7.64
7.64
7.64
7.63
7.63
7.63
7.62
7.26 CDCl3
6.79
6.77
5.34
5.26
3.96
1.00
1.00
0.96
2.03
3.00
1H (ppm)

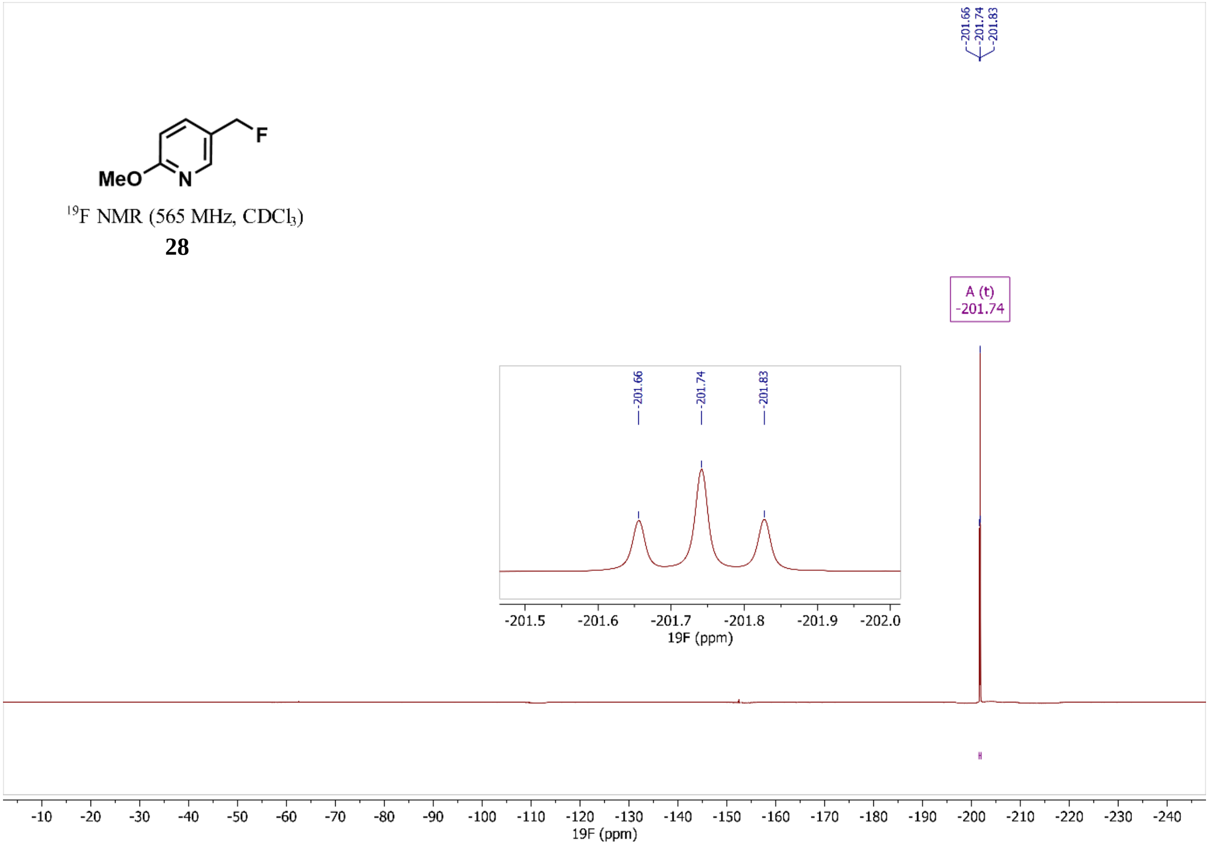

MeO
N
F
^{19}F NMR (565 MHz, $CDCl_3$)
28
-201.66
-201.74
-201.83
A (t)
-201.74
19F (ppm)

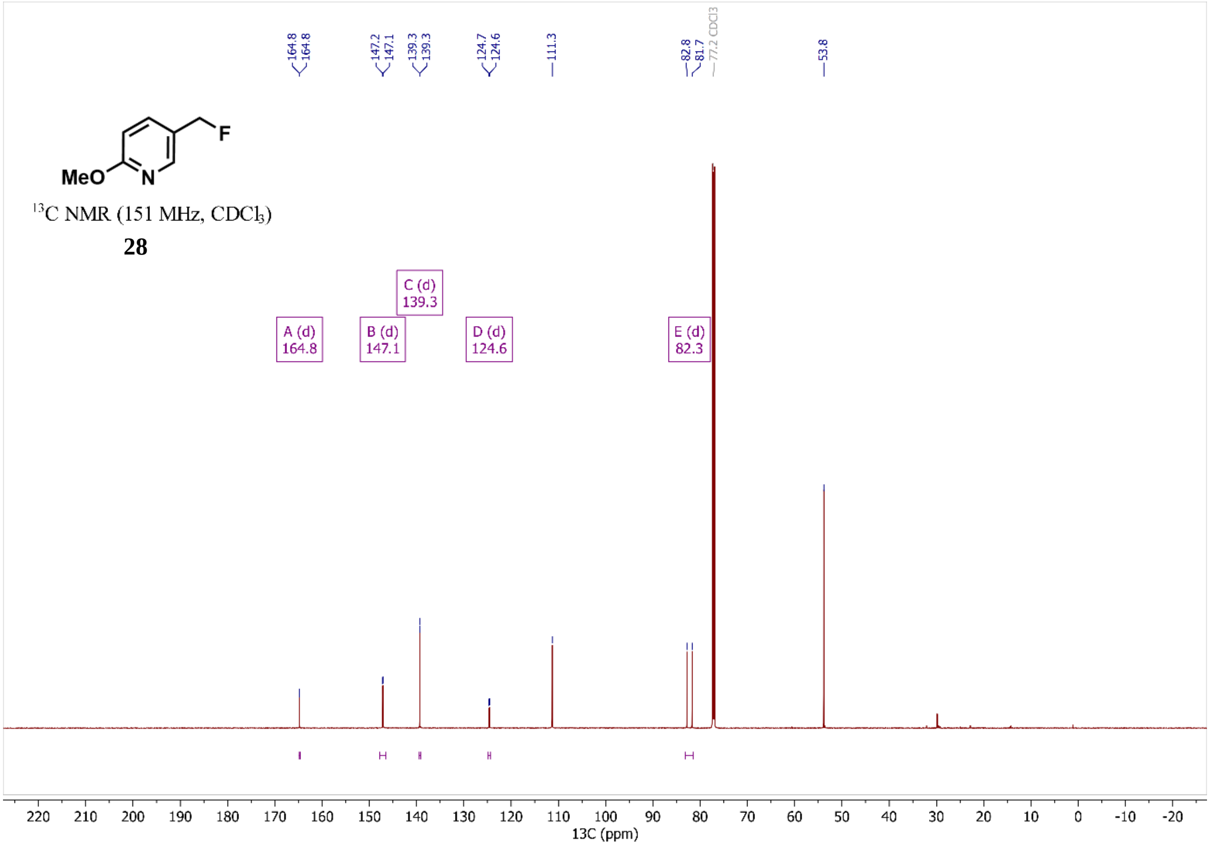
164.8
164.8
147.2
147.1
139.3
139.3
124.7
124.6
111.3
82.8
81.7
77.2 CDCl3
53.8
MeO
N
F
13C NMR (151 MHz, CDCl3)
28
C (d)
139.3
A (d)
164.8
B (d)
147.1
D (d)
124.6
E (d)
82.3
13C (ppm)

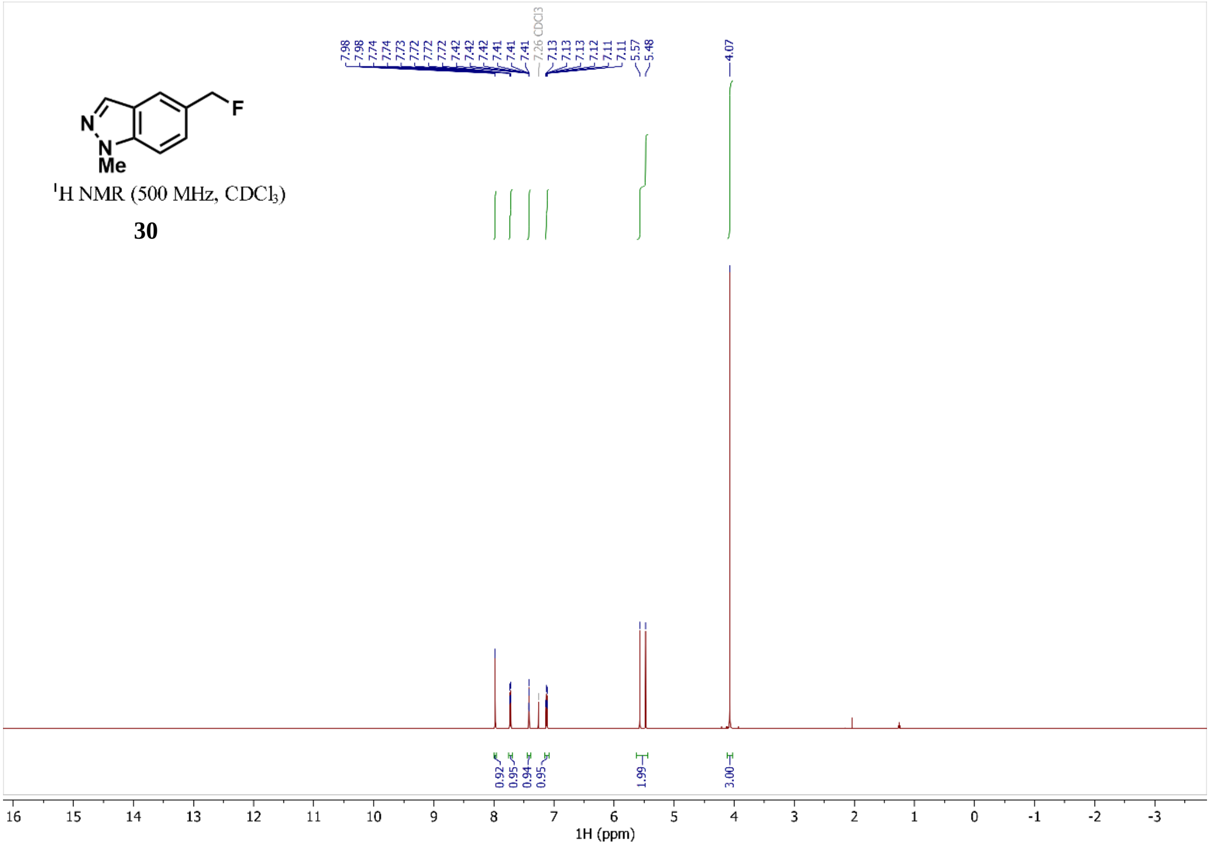
7.98
7.98
7.74
7.74
7.73
7.72
7.72
7.72
7.42
7.42
7.42
7.41
7.41
7.41
7.26 CDCl3
7.13
7.13
7.13
7.12
7.11
7.11
5.57
5.48
4.07
N
N
Me
F
1H NMR (500 MHz, CDCl3)
30
0.92
0.95
0.94
0.95
1.99
3.00
1H (ppm)

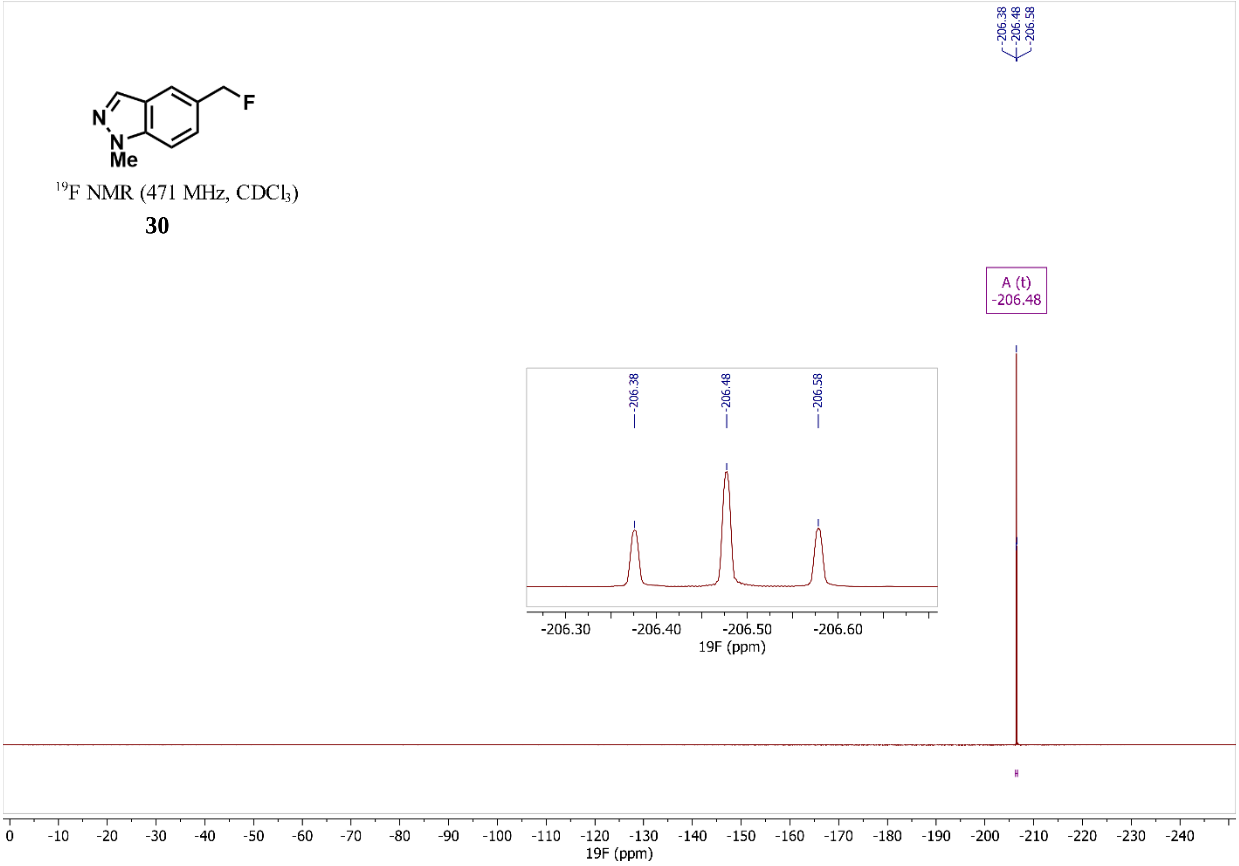

N
N
Me
F
19F NMR (471 MHz, CDCl3)
30
-206.38
-206.48
-206.58
A (t)
-206.48
-206.30
-206.40
-206.50
-206.60
19F (ppm)
0 -10 -20 -30 -40 -50 -60 -70 -80 -90 -100 -110 -120 -130 -140 -150 -160 -170 -180 -190 -200 -210 -220 -230 -240
19F (ppm)

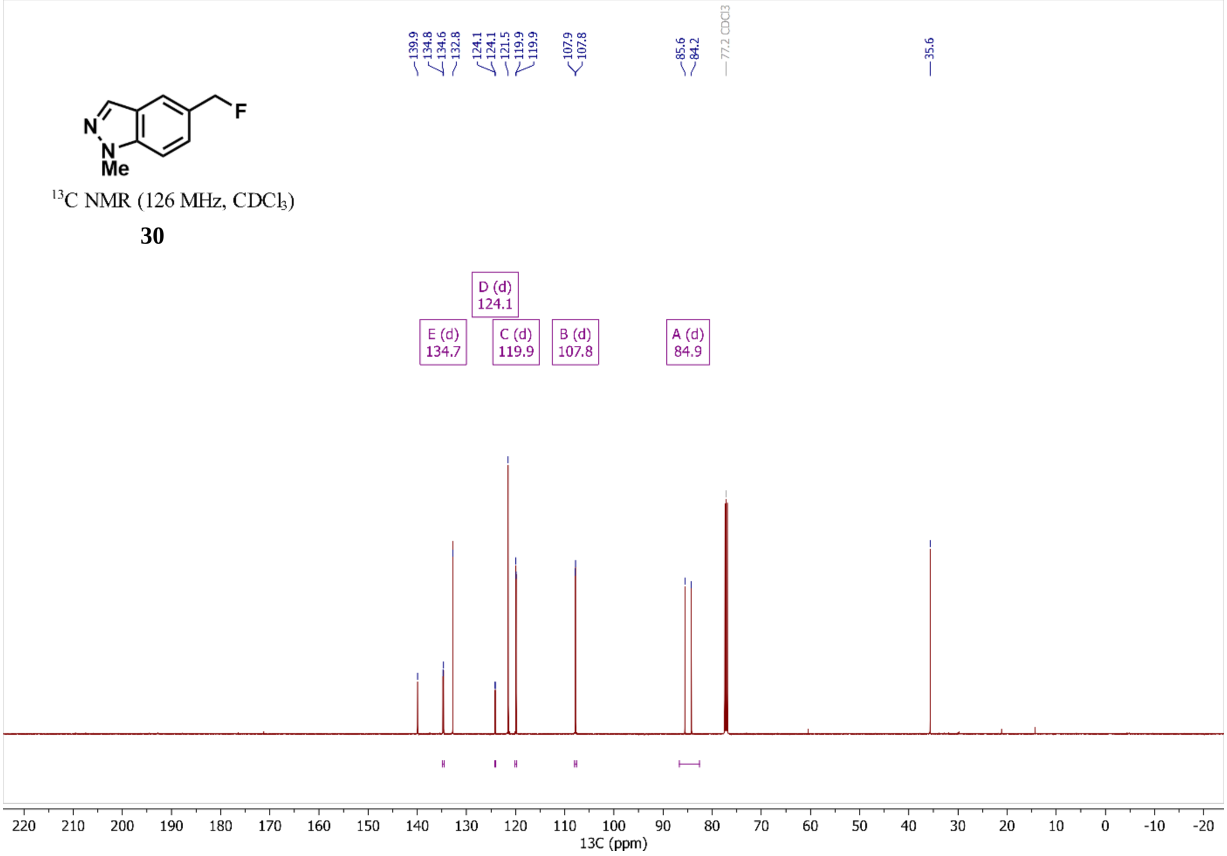

N
N
Me
F
13C NMR (126 MHz, CDCl3)
30
139.9
134.8
134.6
132.8
124.1
124.1
121.5
119.9
119.9
107.9
107.8
85.6
84.2
77.2 CDCl3
35.6
D (d)
124.1
E (d)
134.7
C (d)
119.9
B (d)
107.8
A (d)
84.9
220 210 200 190 180 170 160 150 140 130 120 110 100 90 80 70 60 50 40 30 20 10 0 -10 -20
13C (ppm)

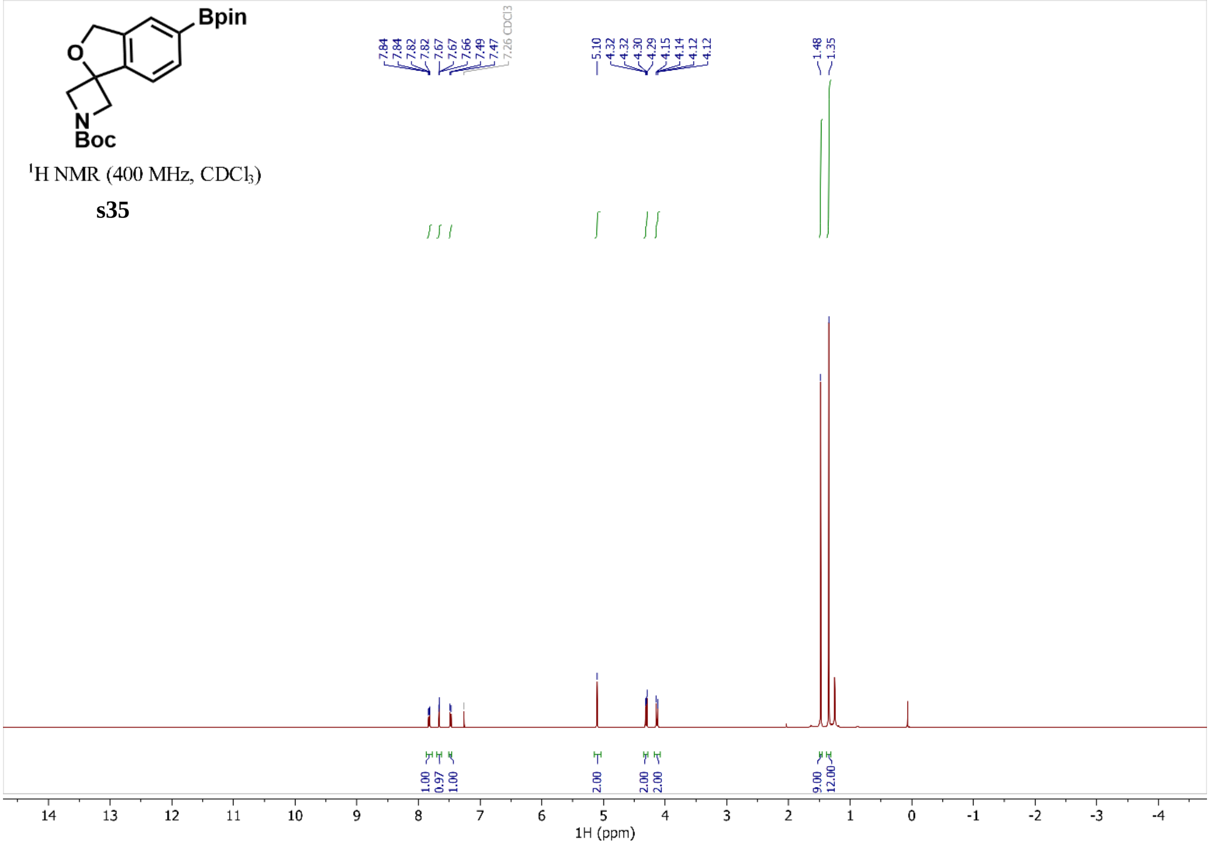
Bpin
O
N
Boc
^{1}H NMR (400 MHz, CDCl$_3$)
s35
7.84 7.84 7.82 7.82 7.67 7.67 7.66 7.49 7.47 7.26 CDCl3
5.10 4.32 4.32 4.30 4.29 4.15 4.14 4.12 4.12
1.48 1.35
1.00 0.97 1.00 2.00 2.00 2.00 9.00 12.00
14 13 12 11 10 9 8 7 6 5 4 3 2 1 0 -1 -2 -3 -4
1H (ppm)

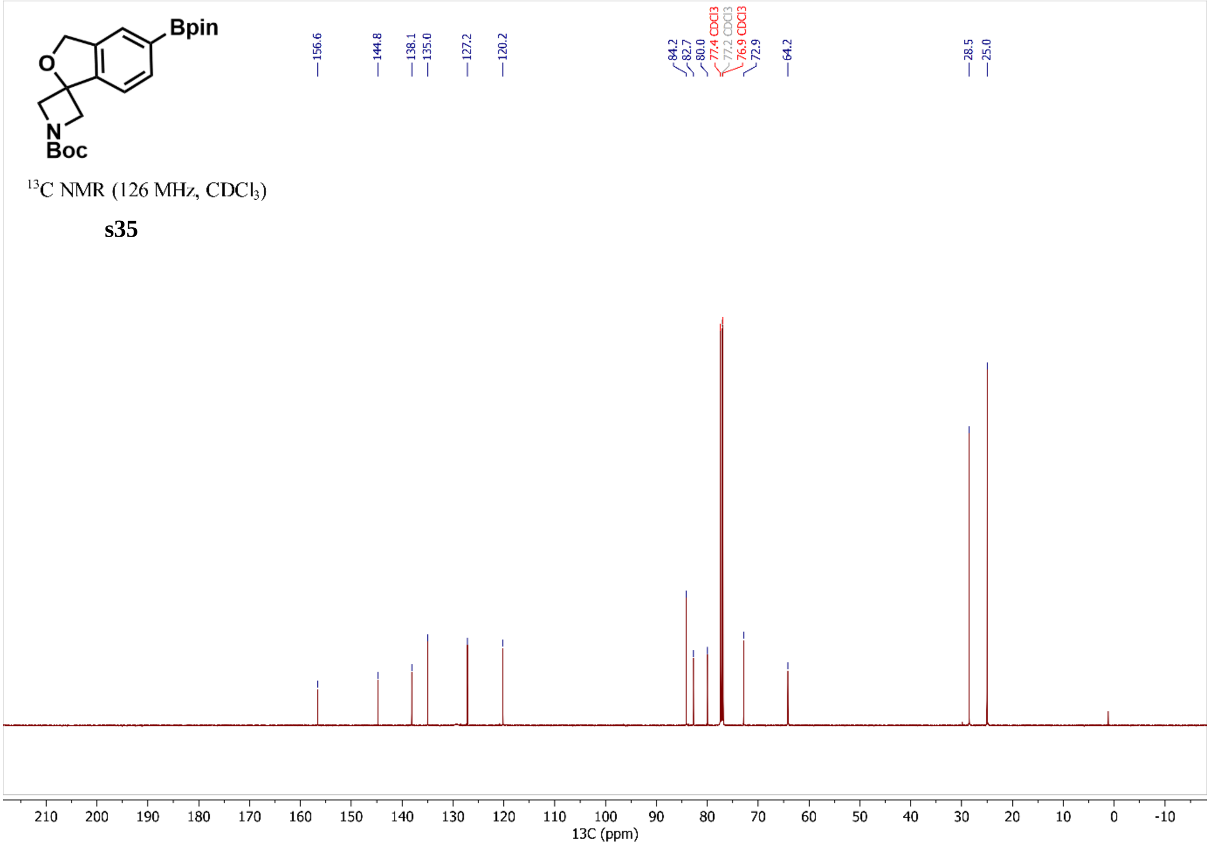
Bpin
O
N
Boc
^{13}C NMR (126 MHz, CDCl$_3$)
s35
156.6 144.8 138.1 135.0 127.2 120.2
84.2 82.7 80.0 77.4 CDCl3 77.2 CDCl3 76.9 CDCl3 72.9 64.2
28.5 25.0
210 200 190 180 170 160 150 140 130 120 110 100 90 80 70 60 50 40 30 20 10 0 -10
13C (ppm)

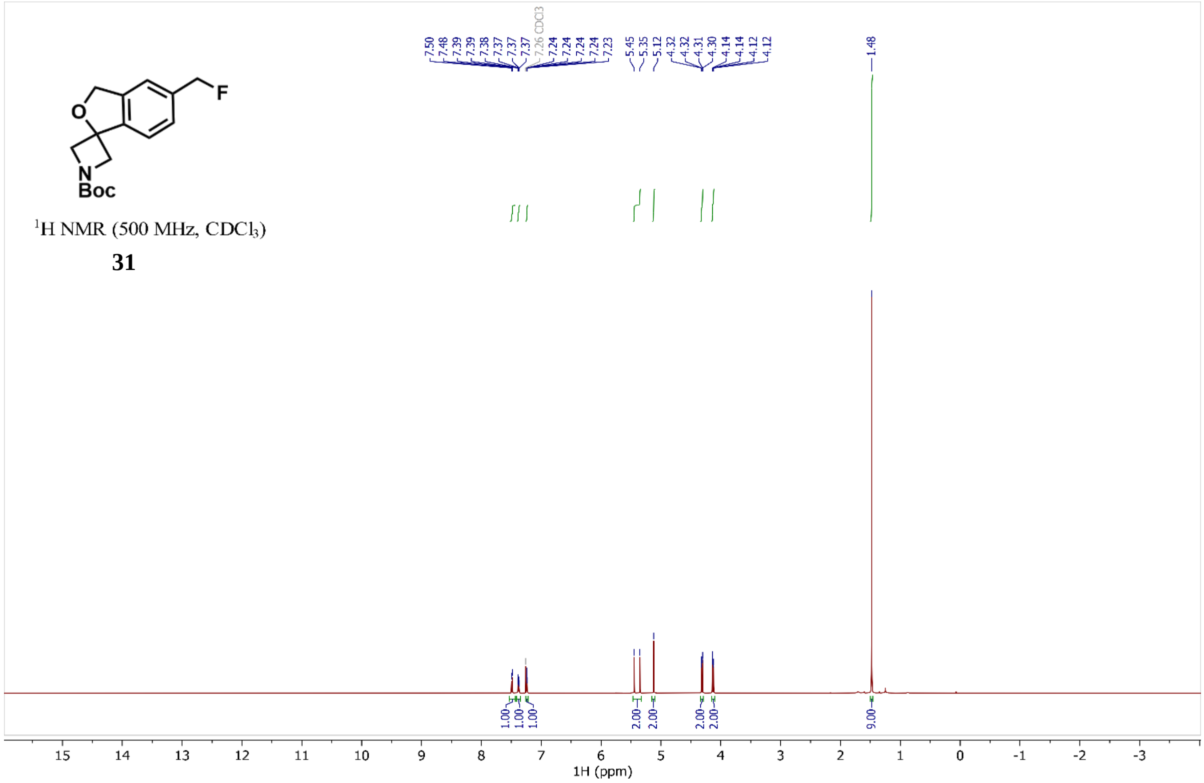

^{1}H NMR (500 MHz, $CDCl_3$)

31

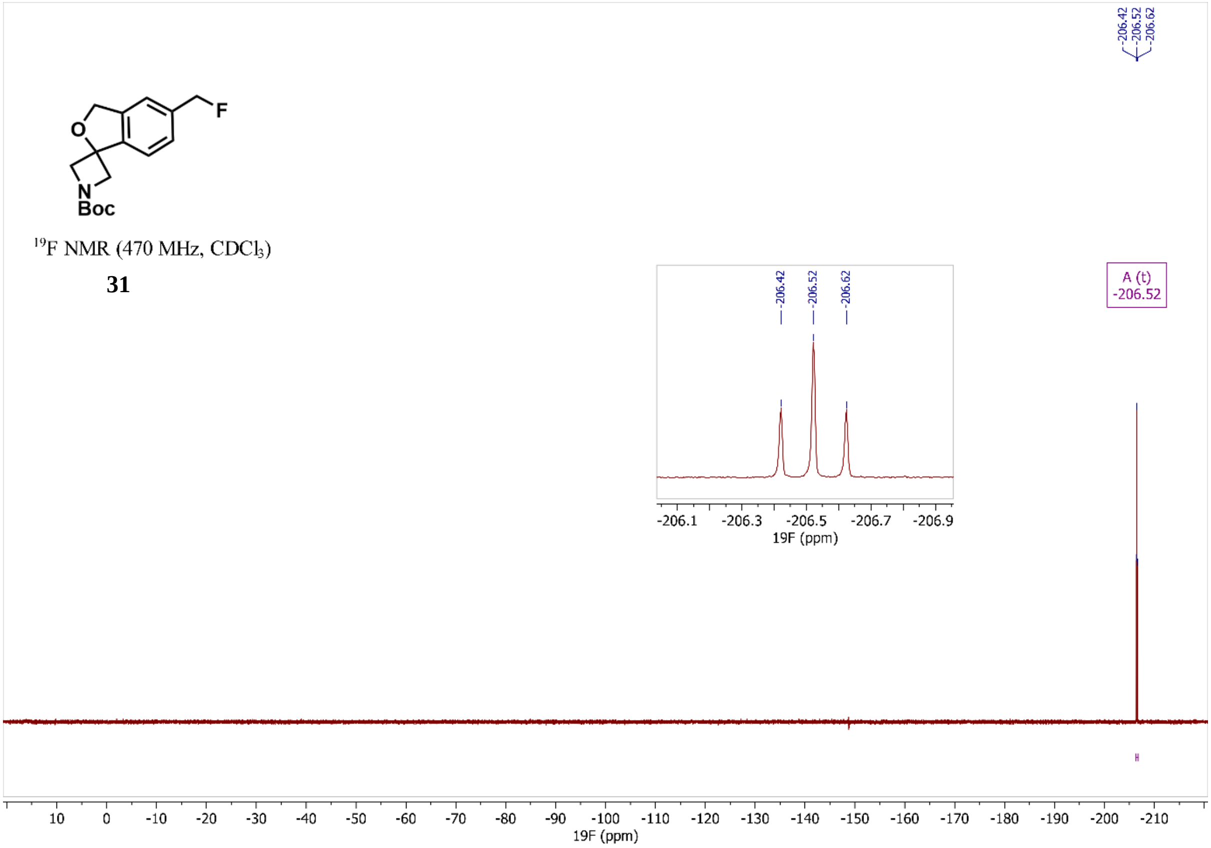

^{19}F NMR (470 MHz, $CDCl_3$)

31

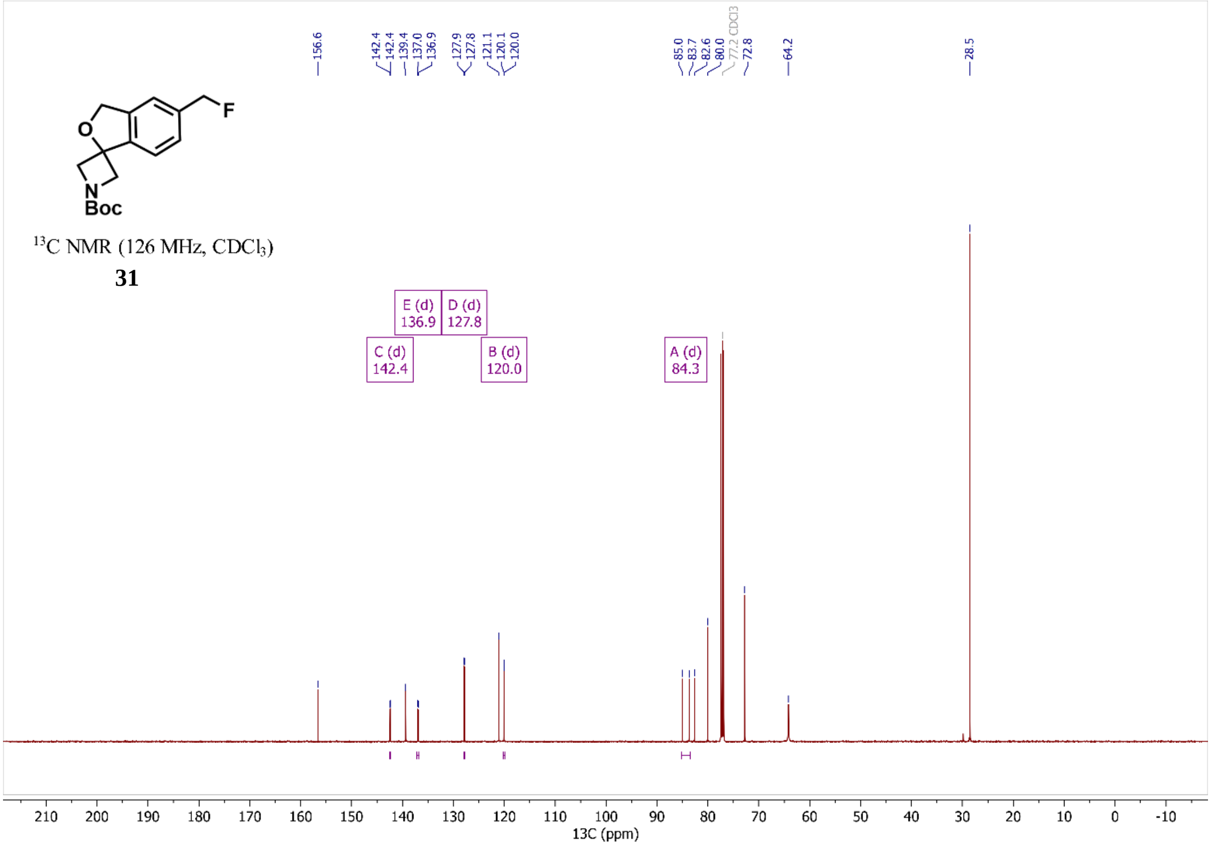

^{13}C NMR (126 MHz, $CDCl_3$)

31

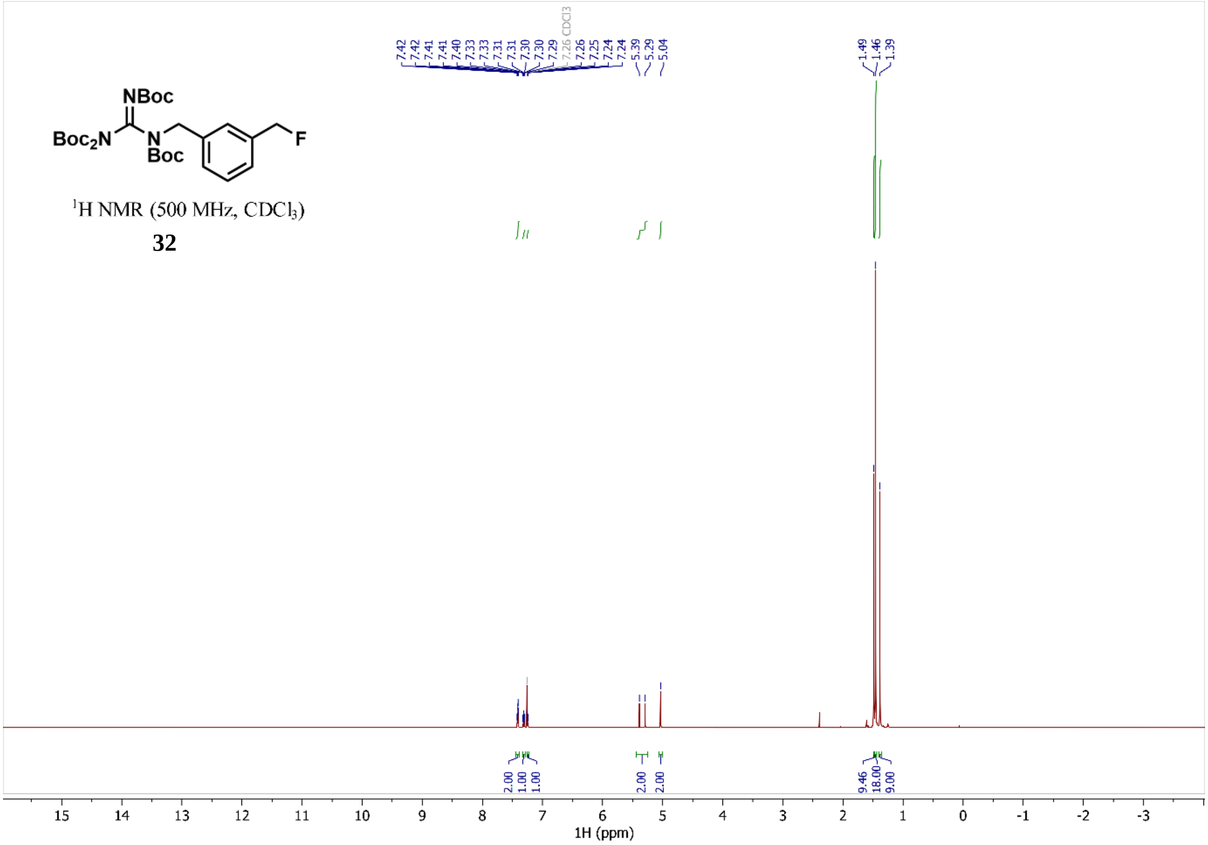

^{1}H NMR (500 MHz, $CDCl_3$)

32

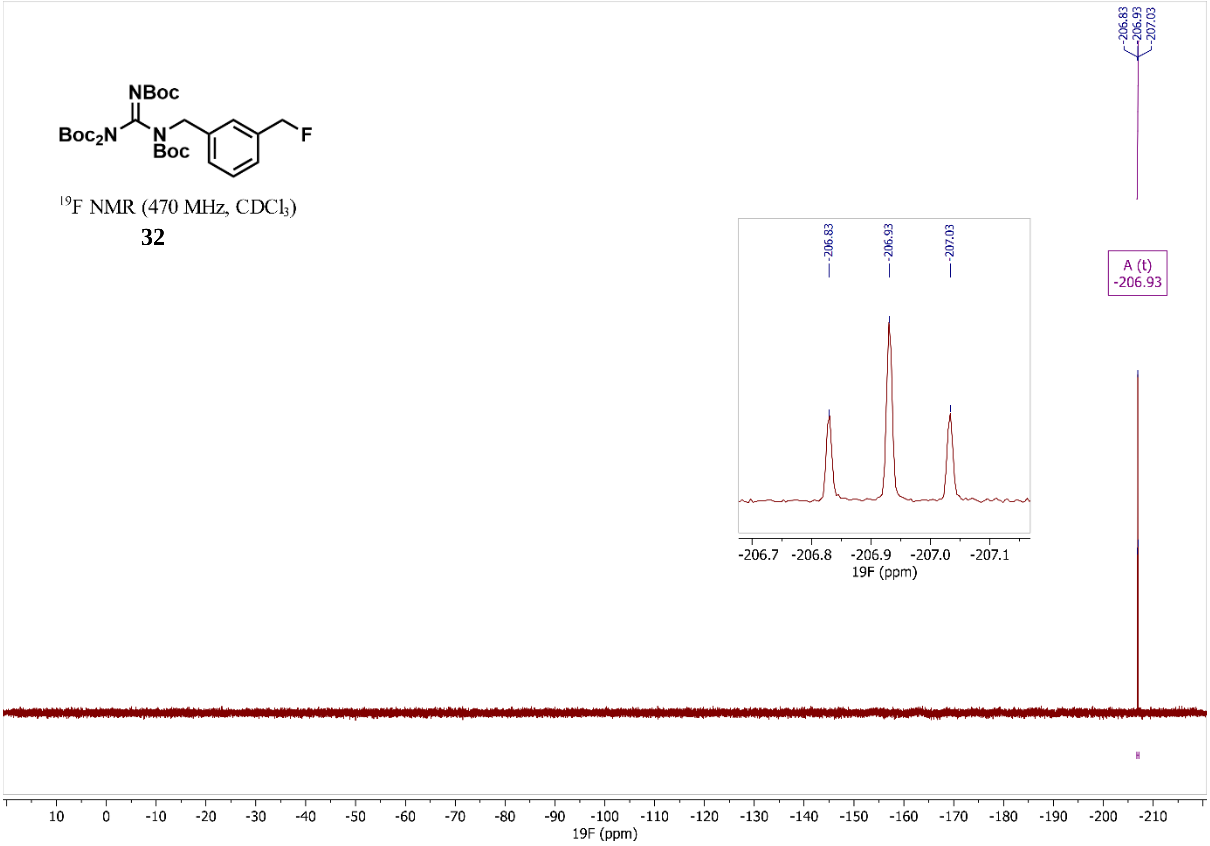
NBoc
Boc2N
N
Boc
F
19F NMR (470 MHz, CDCl3)
32
-206.83
-206.93
-207.03
A (t)
-206.93
-206.7 -206.8 -206.9 -207.0 -207.1
19F (ppm)
10 0 -10 -20 -30 -40 -50 -60 -70 -80 -90 -100 -110 -120 -130 -140 -150 -160 -170 -180 -190 -200 -210
19F (ppm)

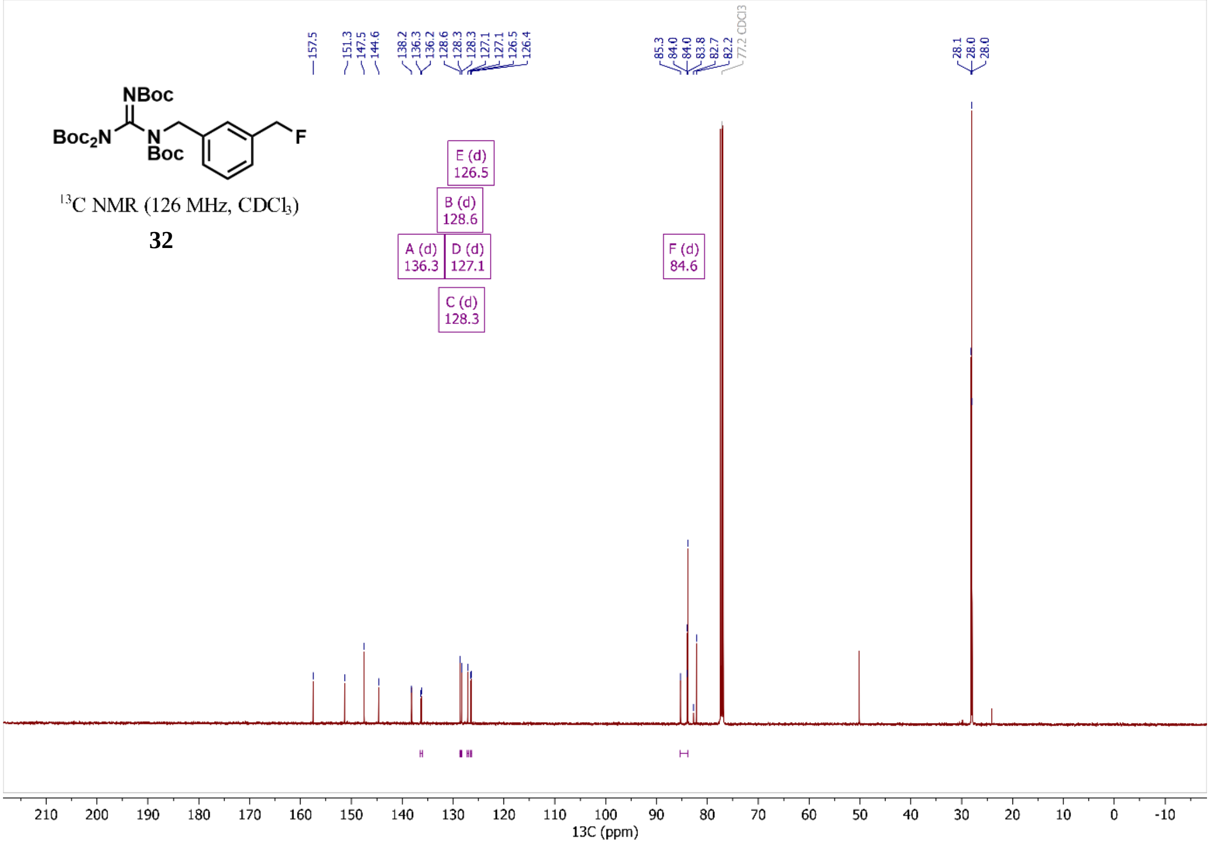
NBoc
Boc2N
N
Boc
F
13C NMR (126 MHz, CDCl3)
32
157.5
151.3
147.5
144.6
138.2
136.3
136.2
128.6
128.3
128.3
127.1
127.1
126.5
126.4
85.3
84.0
84.0
83.8
82.7
82.2
77.2 CDCl3
28.1
28.0
28.0
E (d)
126.5
B (d)
128.6
A (d)
136.3
D (d)
127.1
C (d)
128.3
F (d)
84.6
210 200 190 180 170 160 150 140 130 120 110 100 90 80 70 60 50 40 30 20 10 0 -10
13C (ppm)

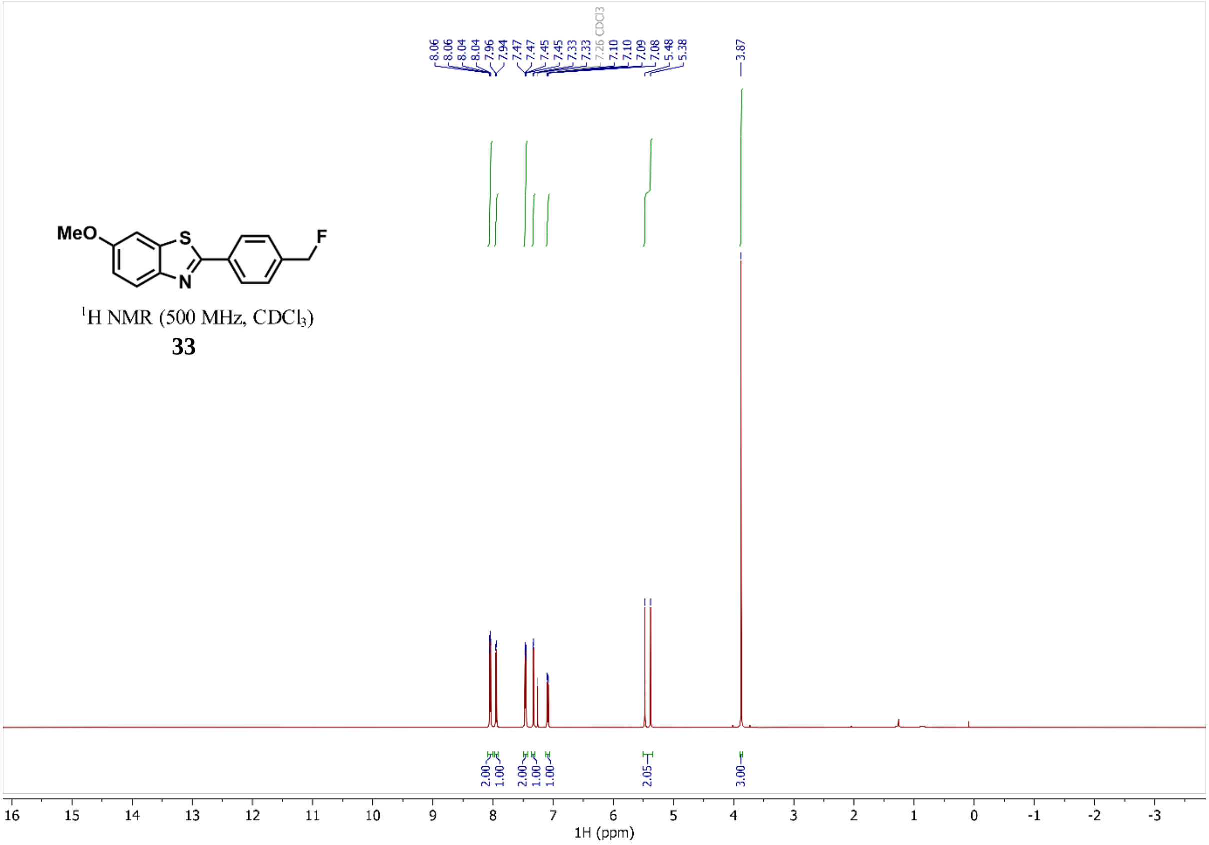
MeO
S
N
F
^{1}H NMR (500 MHz, $CDCl_3$)
33
8.06
8.06
8.04
8.04
7.96
7.94
7.47
7.47
7.45
7.45
7.33
7.33
7.26 CDCl3
7.10
7.10
7.09
7.08
5.48
5.38
3.87
2.00
1.00
2.00
1.00
1.00
2.05
3.00
1H (ppm)

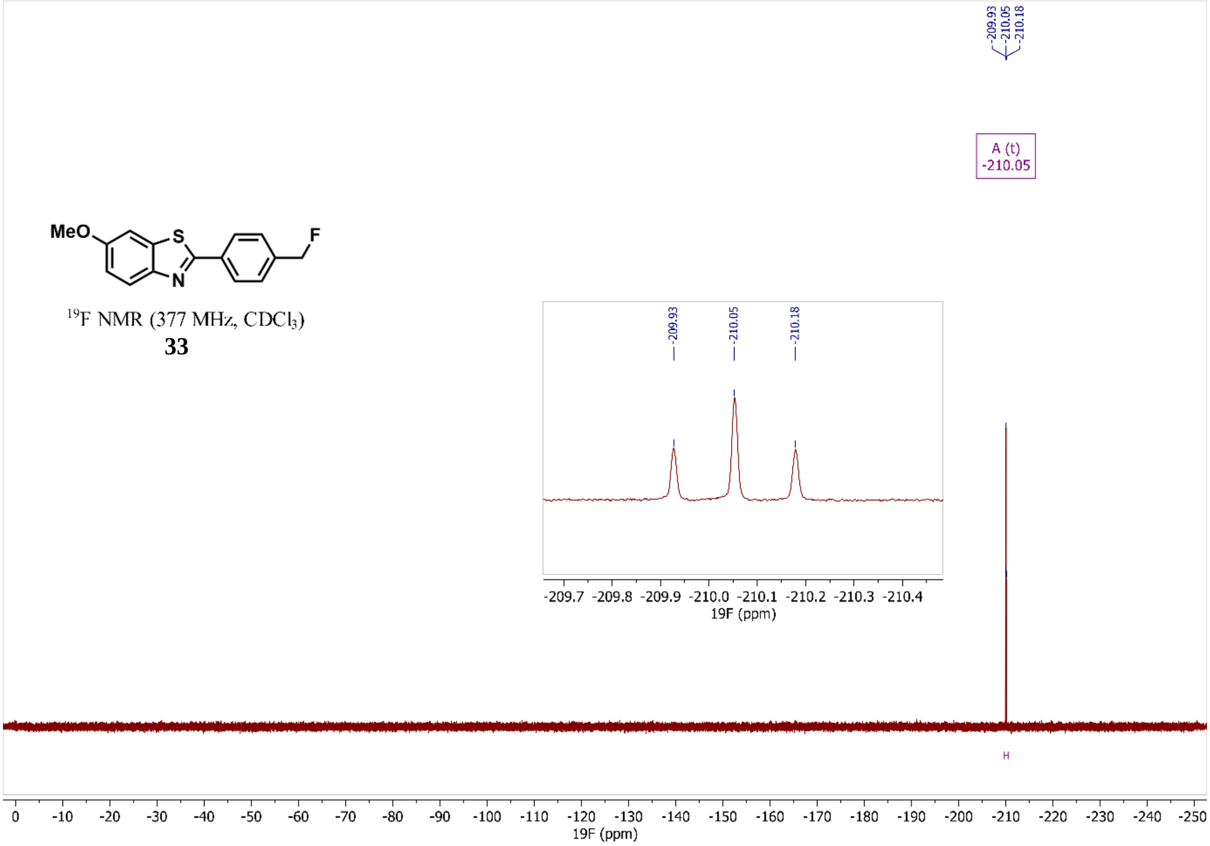
MeO
S
N
F
^{19}F NMR (377 MHz, $CDCl_3$)
33
-209.93
-210.05
-210.18
A (t)
-210.05
19F (ppm)

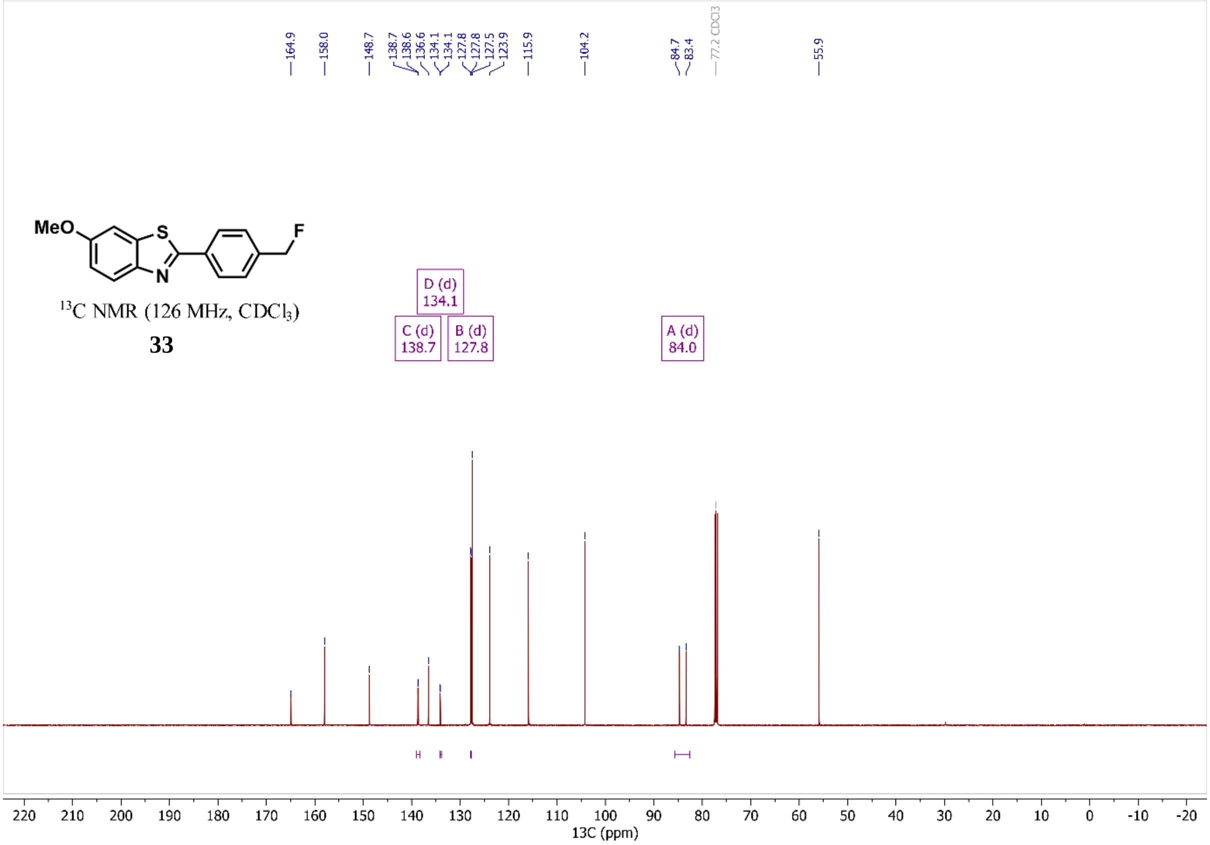

^{13}C NMR (126 MHz, $CDCl_3$)

33

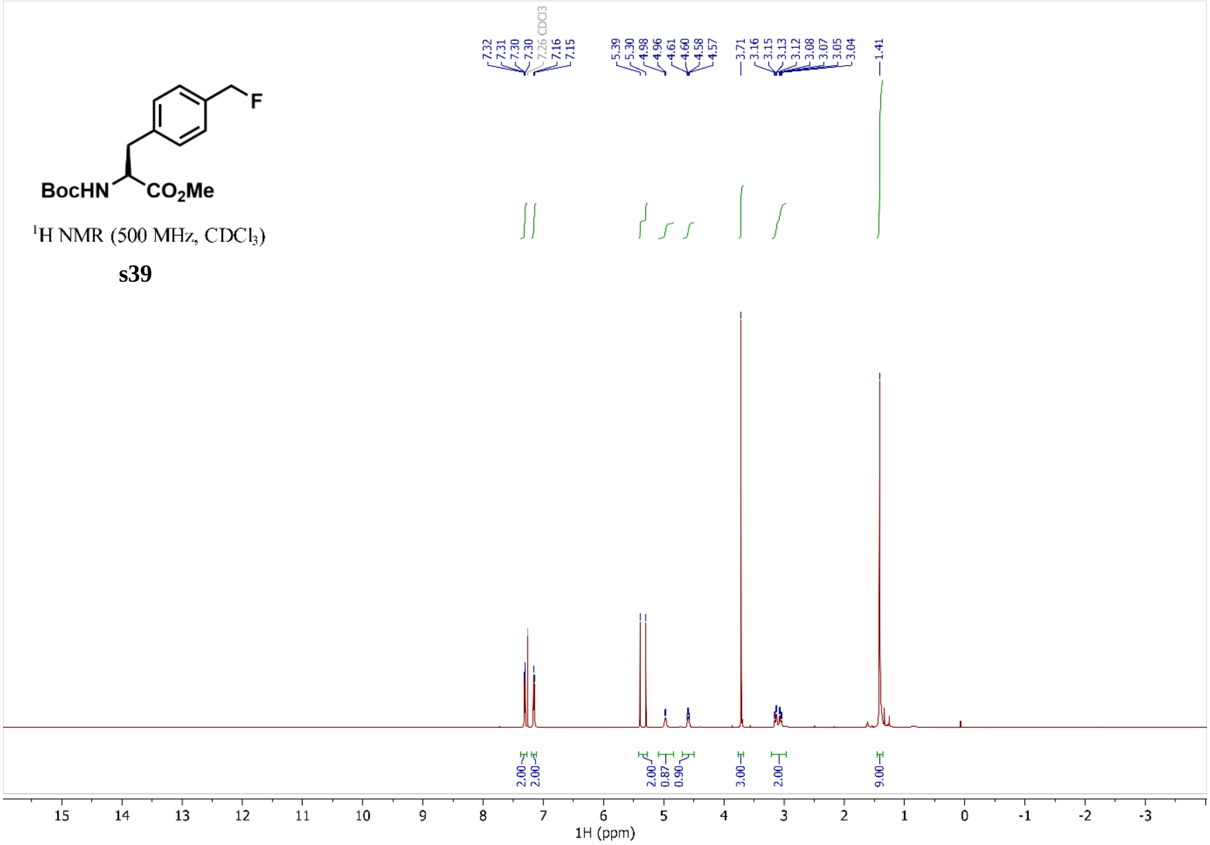

^{1}H NMR (500 MHz, $CDCl_3$)

s39

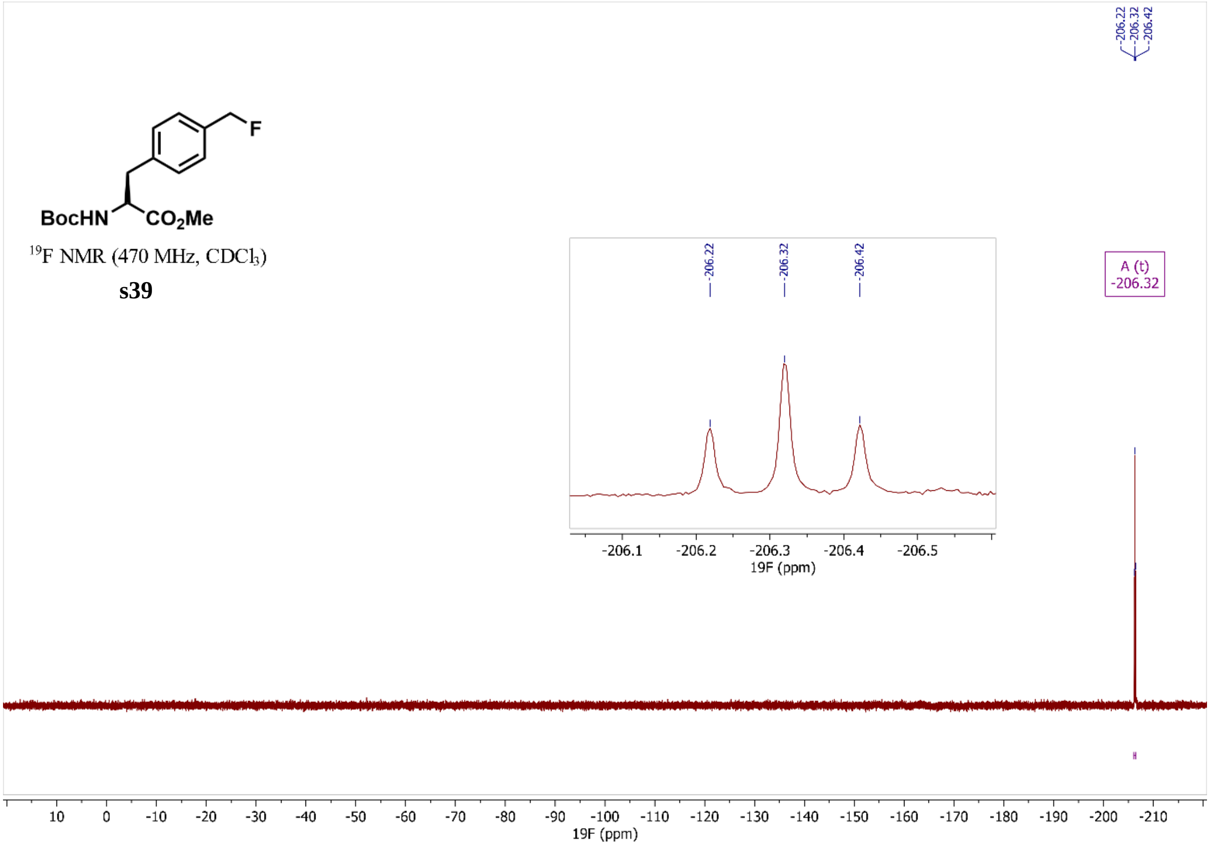

BocHN
CO2Me
F
19F NMR (470 MHz, CDCl3)
s39
-206.22
-206.32
-206.42
A (t)
-206.32
19F (ppm)

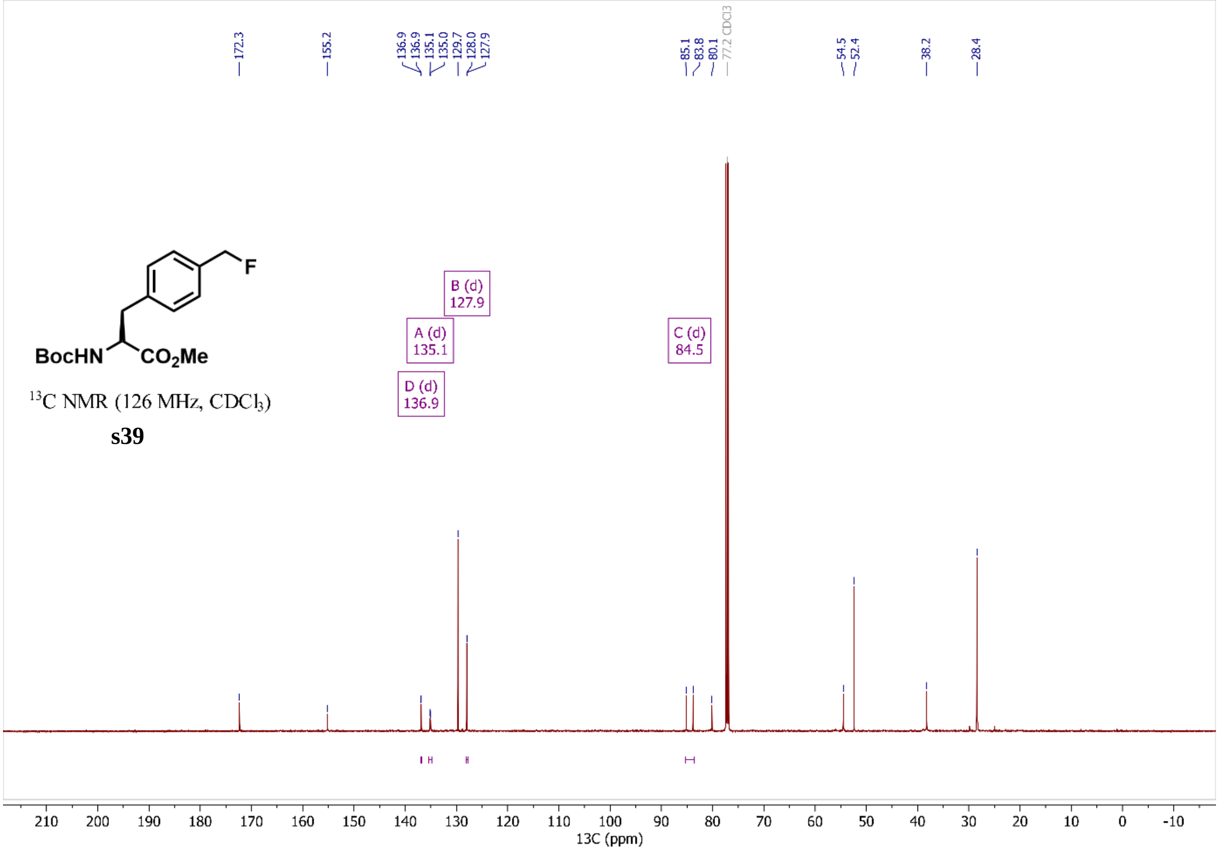

BocHN
CO2Me
F
13C NMR (126 MHz, CDCl3)
s39
172.3
155.2
136.9
136.9
135.1
135.0
129.7
128.0
127.9
85.1
83.8
80.1
77.2 CDCl3
54.5
52.4
38.2
28.4
B (d)
127.9
A (d)
135.1
D (d)
136.9
C (d)
84.5
13C (ppm)

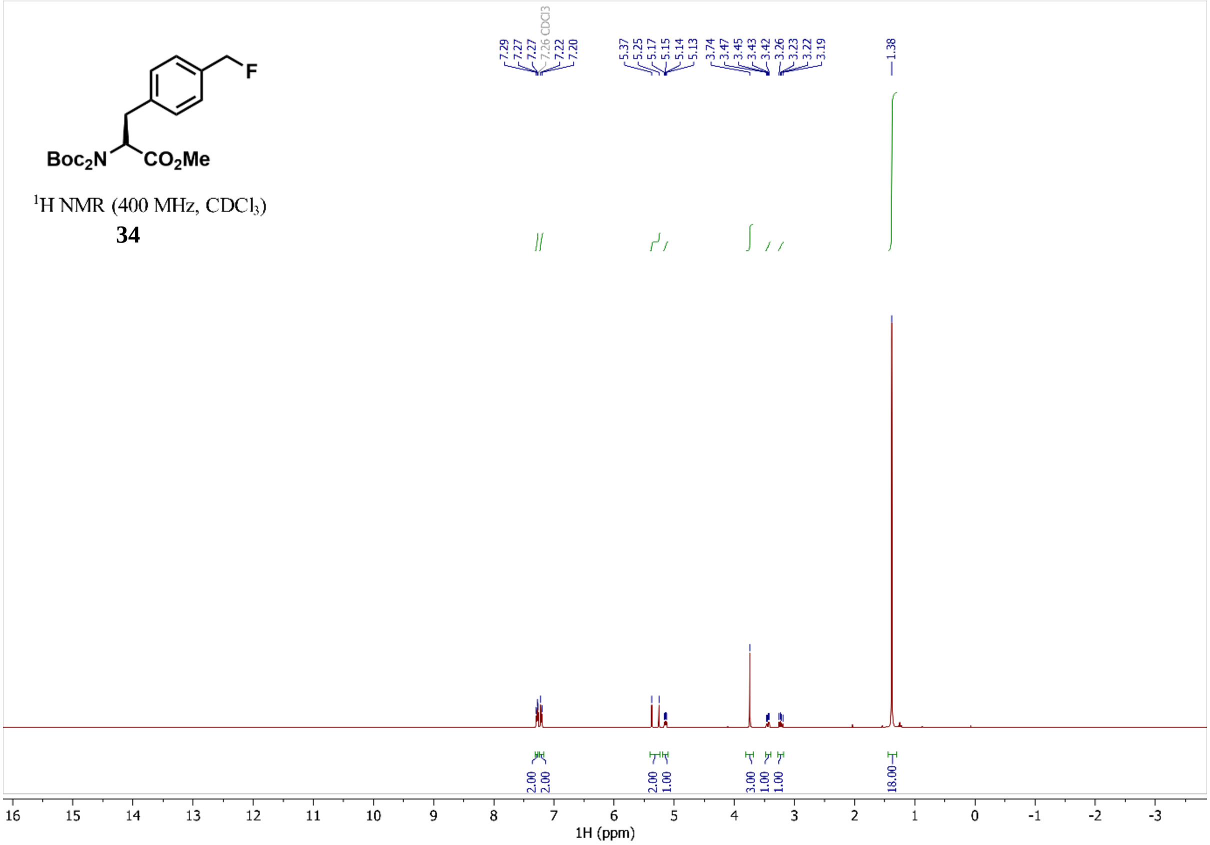
Boc2N
CO2Me
F
^{1}H NMR (400 MHz, CDCl3)
34
7.29
7.27
7.27
7.26 CDCl3
7.22
7.20
5.37
5.25
5.17
5.15
5.14
5.13
3.74
3.47
3.45
3.43
3.42
3.26
3.23
3.22
3.19
1.38
2.00
2.00
2.00
1.00
3.00
1.00
1.00
18.00
1H (ppm)

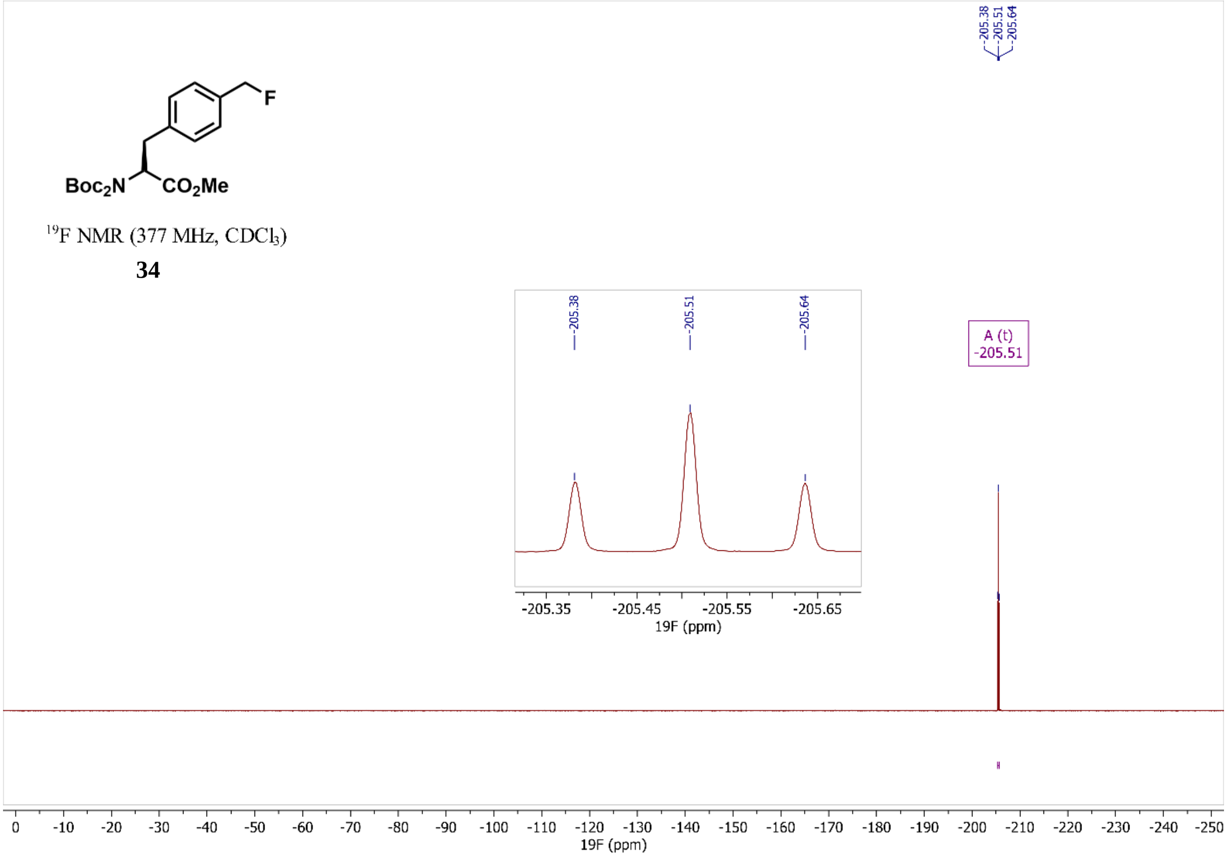
Boc2N
CO2Me
F
^{19}F NMR (377 MHz, CDCl3)
34
-205.38
-205.51
-205.64
A (t)
-205.51
19F (ppm)

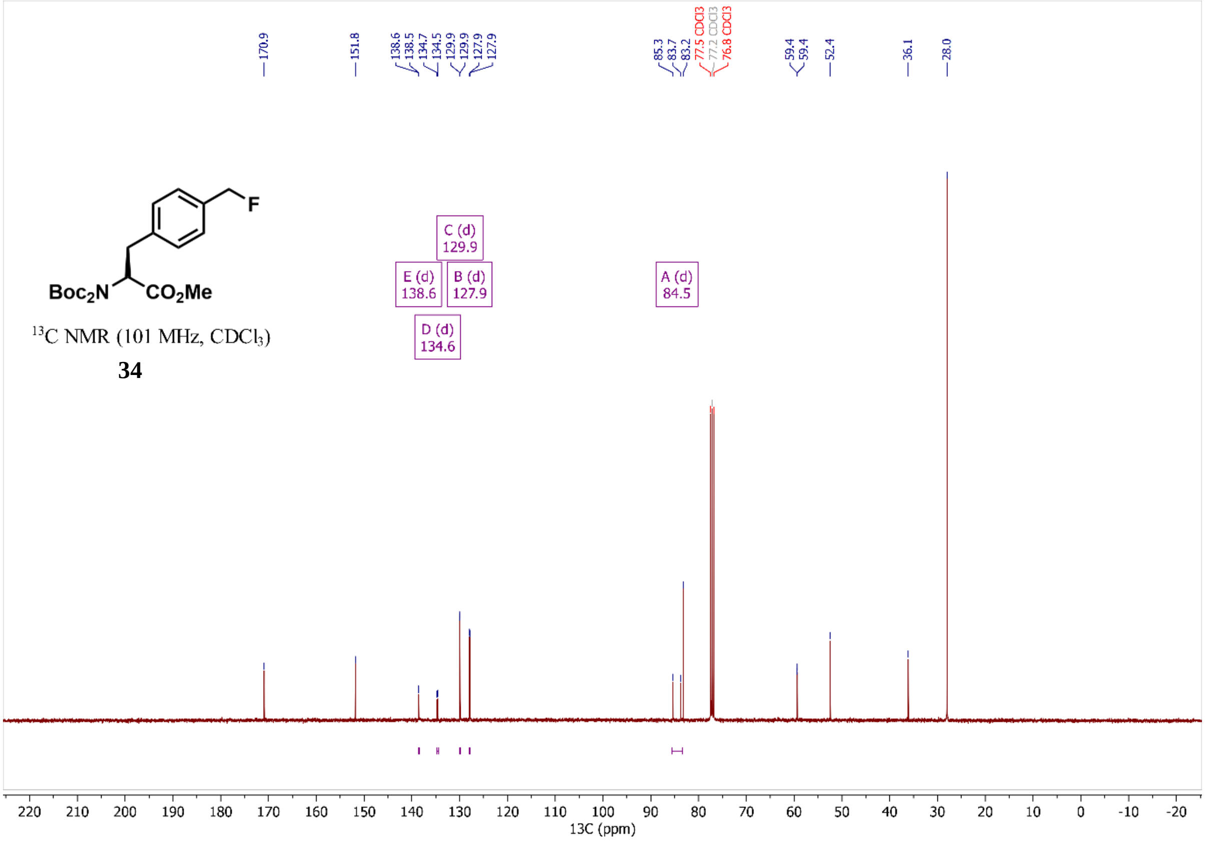

^{13}C NMR (101 MHz, $CDCl_3$)

34

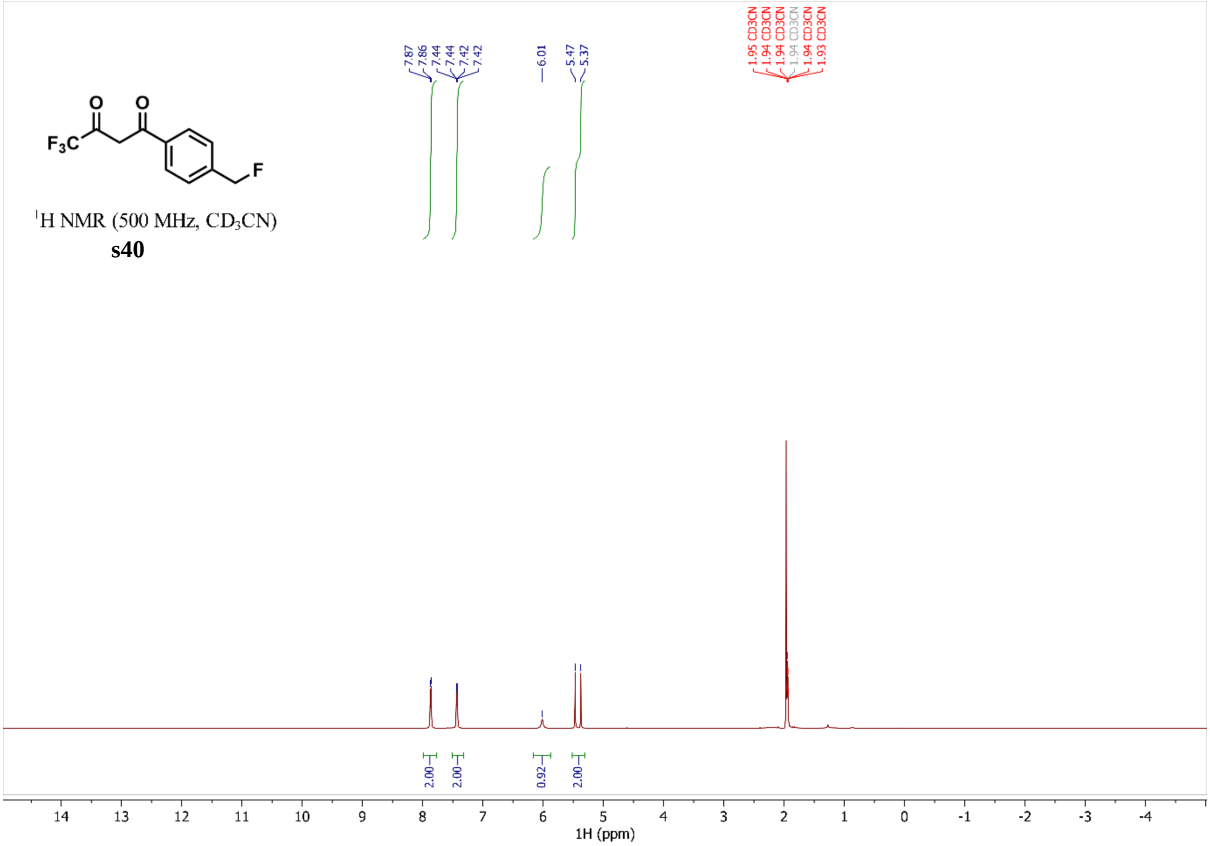

^{1}H NMR (500 MHz, CD_3CN)

s40

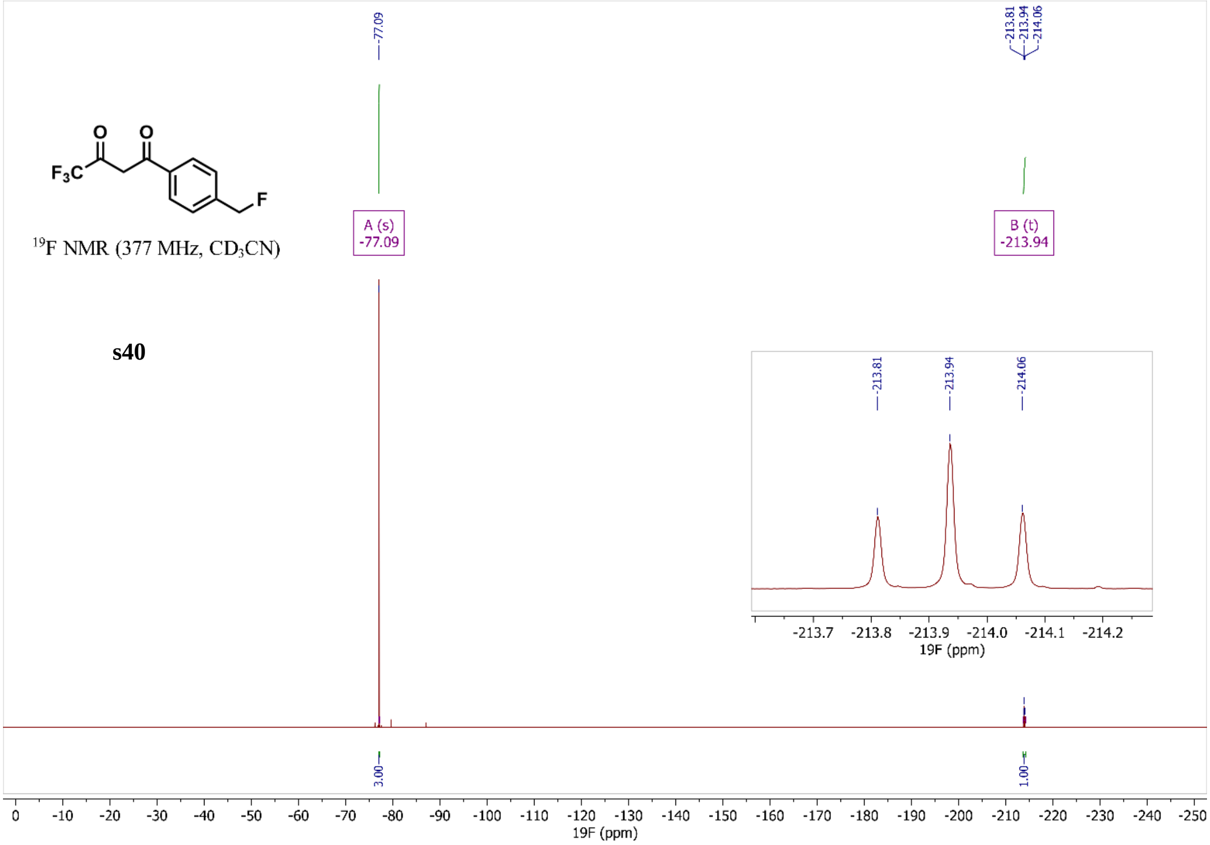
-77.09
-213.81
-213.94
-214.06
O
O
F3C
F
19F NMR (377 MHz, CD3CN)
s40
A (s)
-77.09
B (t)
-213.94
-213.7
-213.8
-213.9
-214.0
-214.1
-214.2
19F (ppm)
3.00
1.00
0
-10
-20
-30
-40
-50
-60
-70
-80
-90
-100
-110
-120
-130
-140
-150
-160
-170
-180
-190
-200
-210
-220
-230
-240
-250
19F (ppm)

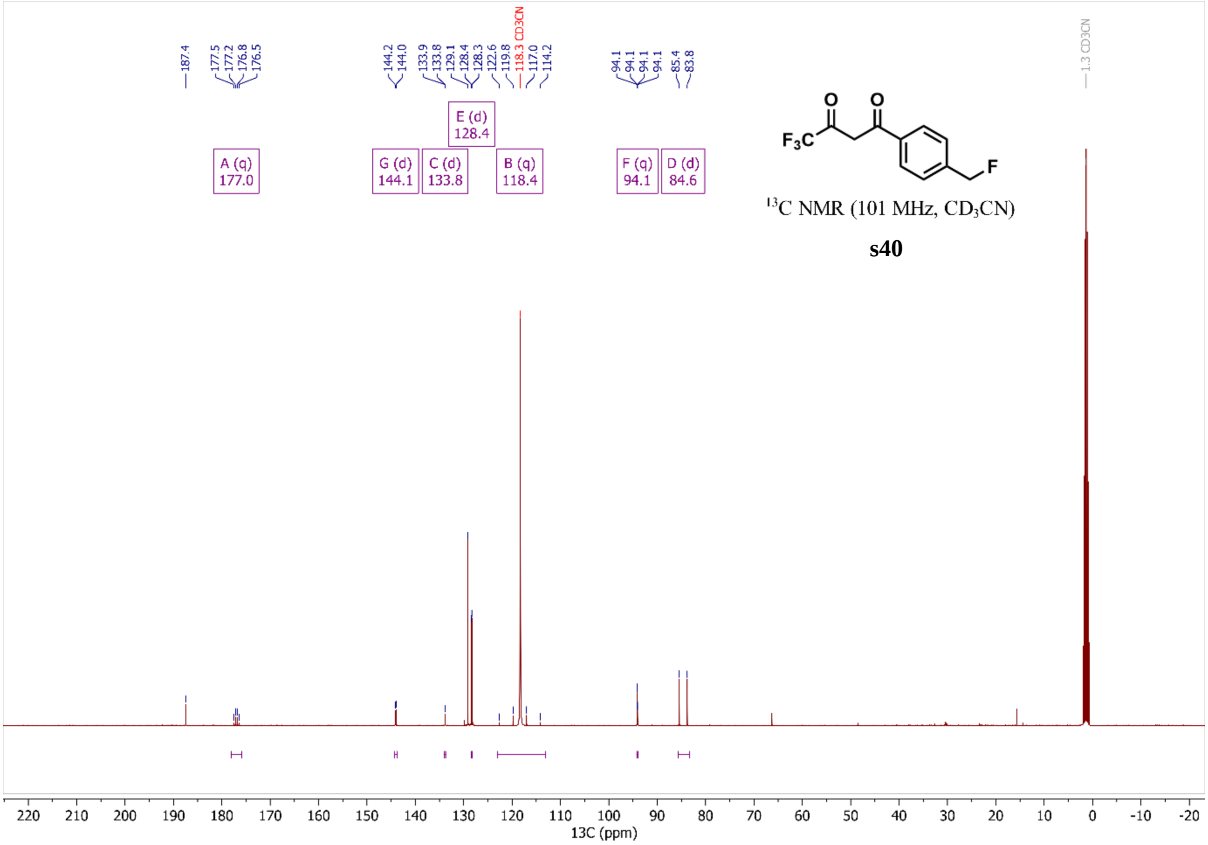
187.4
177.5
177.2
176.8
176.5
144.2
144.0
133.9
133.8
129.1
128.4
128.3
122.6
119.8
118.3 CD3CN
117.0
114.2
94.1
94.1
94.1
94.1
85.4
83.8
1.3 CD3CN
E (d)
128.4
A (q)
177.0
G (d)
144.1
C (d)
133.8
B (q)
118.4
F (q)
94.1
D (d)
84.6
O
O
F3C
F
13C NMR (101 MHz, CD3CN)
s40
220
210
200
190
180
170
160
150
140
130
120
110
100
90
80
70
60
50
40
30
20
10
0
-10
-20
13C (ppm)

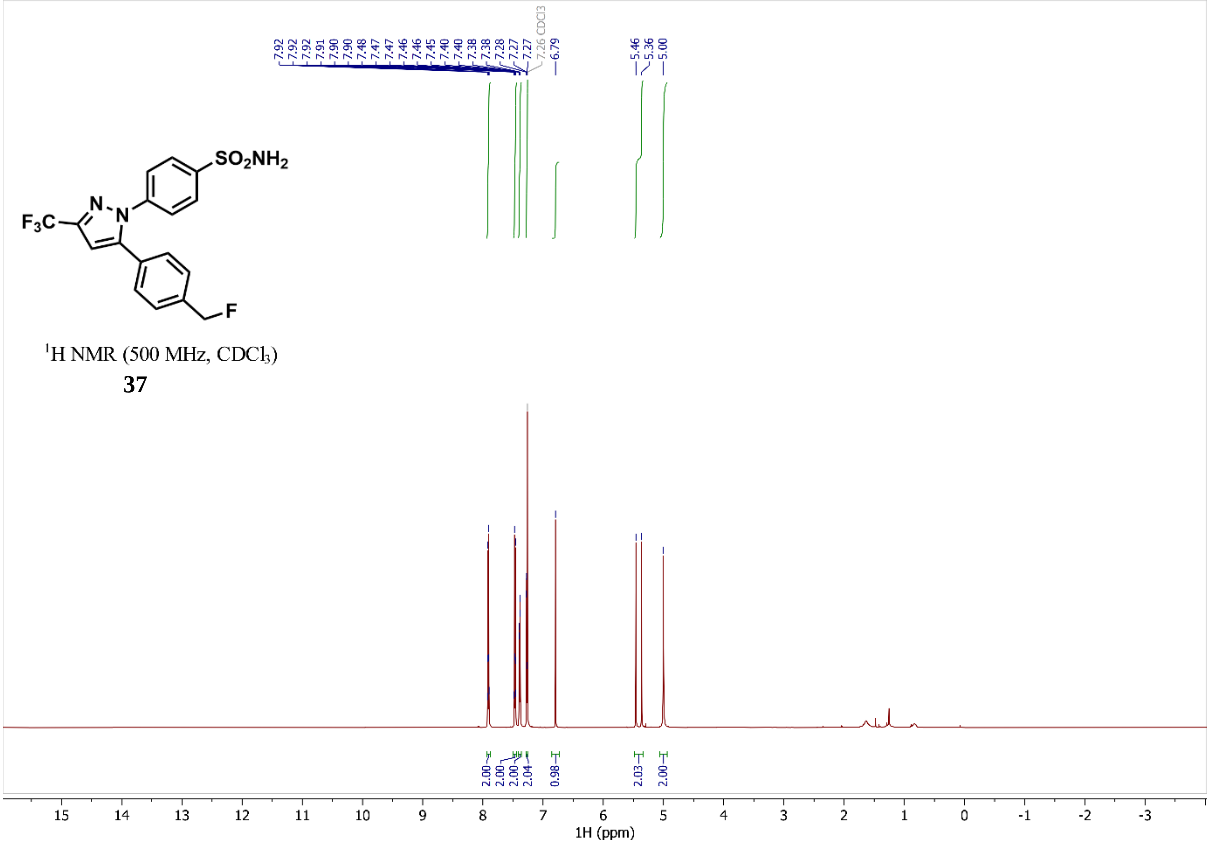

SO2NH2
F3C
N
N
F
1H NMR (500 MHz, CDCl3)
37
7.92
7.92
7.92
7.91
7.90
7.90
7.48
7.47
7.47
7.46
7.46
7.45
7.40
7.40
7.38
7.38
7.28
7.27
7.27
7.26 CDCl3
6.79
5.46
5.36
5.00
2.00
2.00
2.00
2.04
0.98
2.03
2.00
1H (ppm)

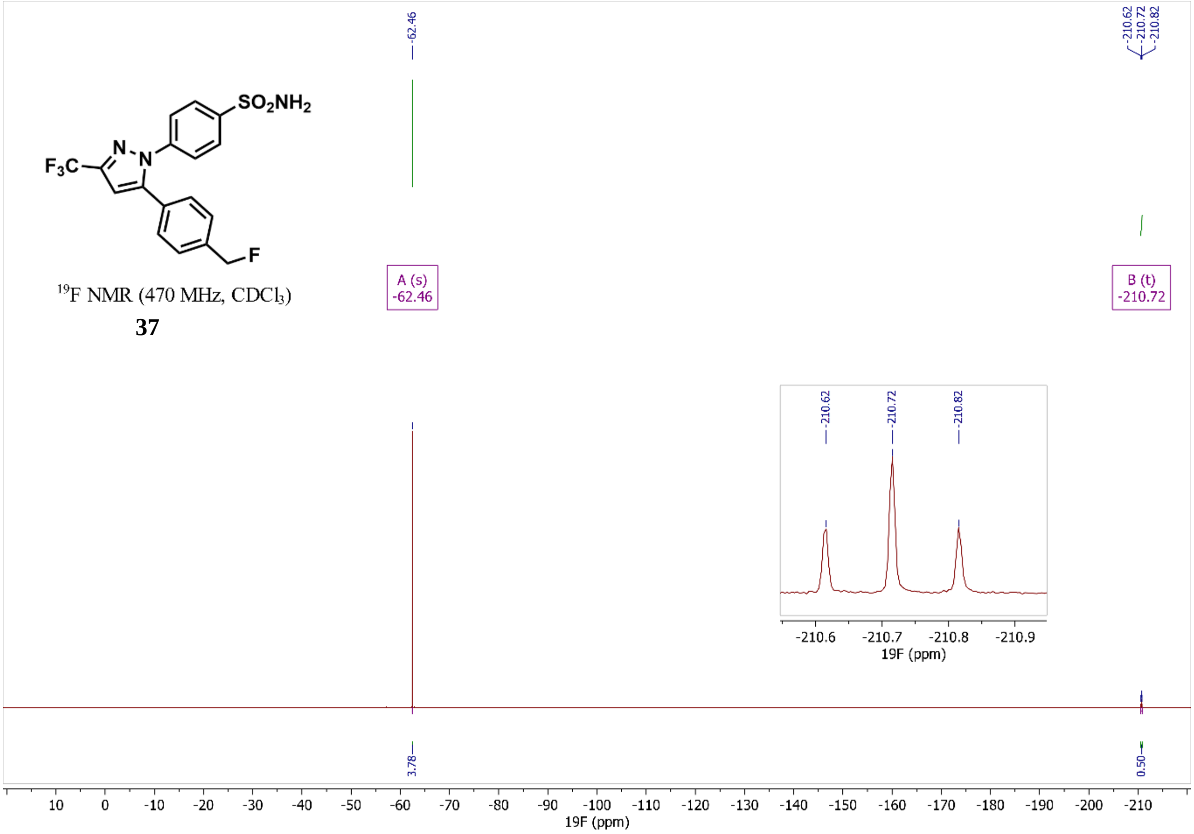

SO2NH2
F3C
N
N
F
19F NMR (470 MHz, CDCl3)
37
-62.46
-210.62
-210.72
-210.82
A (s)
-62.46
B (t)
-210.72
3.78
0.50
19F (ppm)

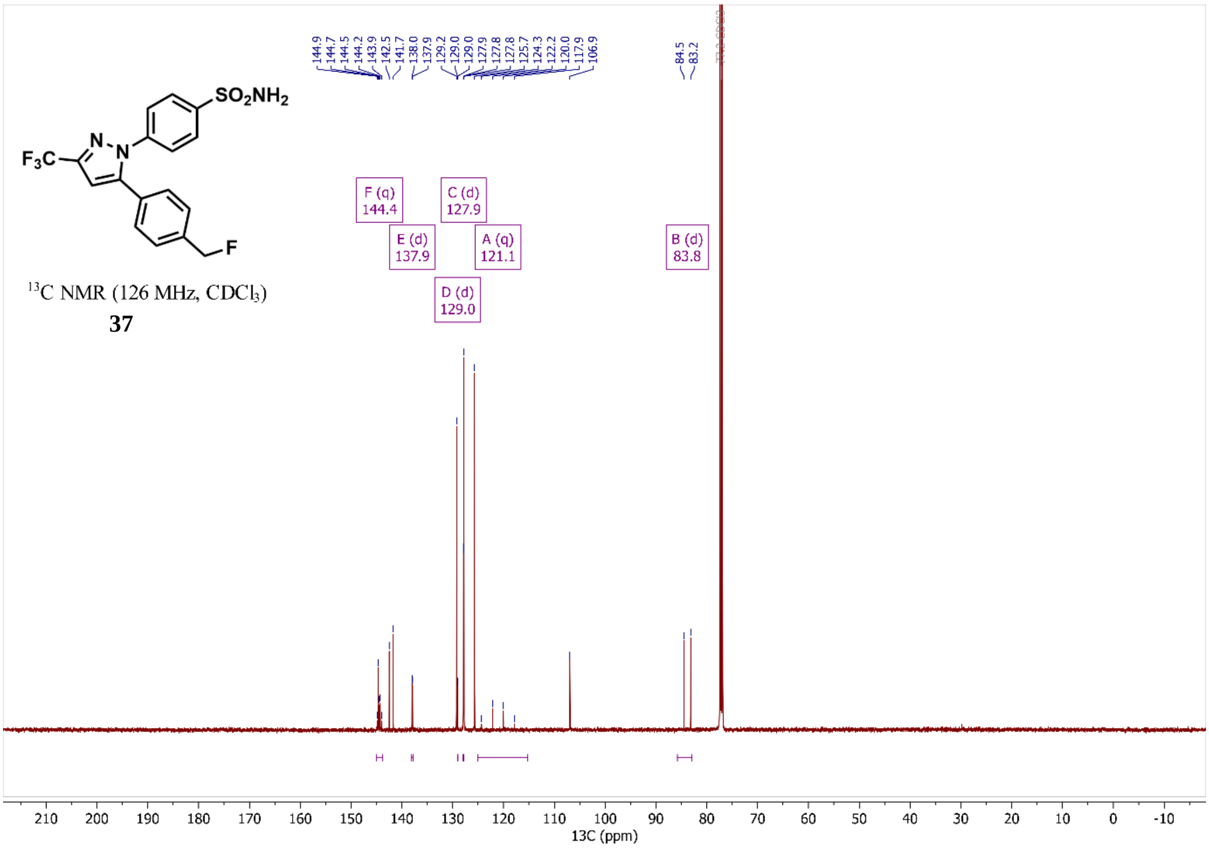

^{13}C NMR (126 MHz, $CDCl_3$)

37

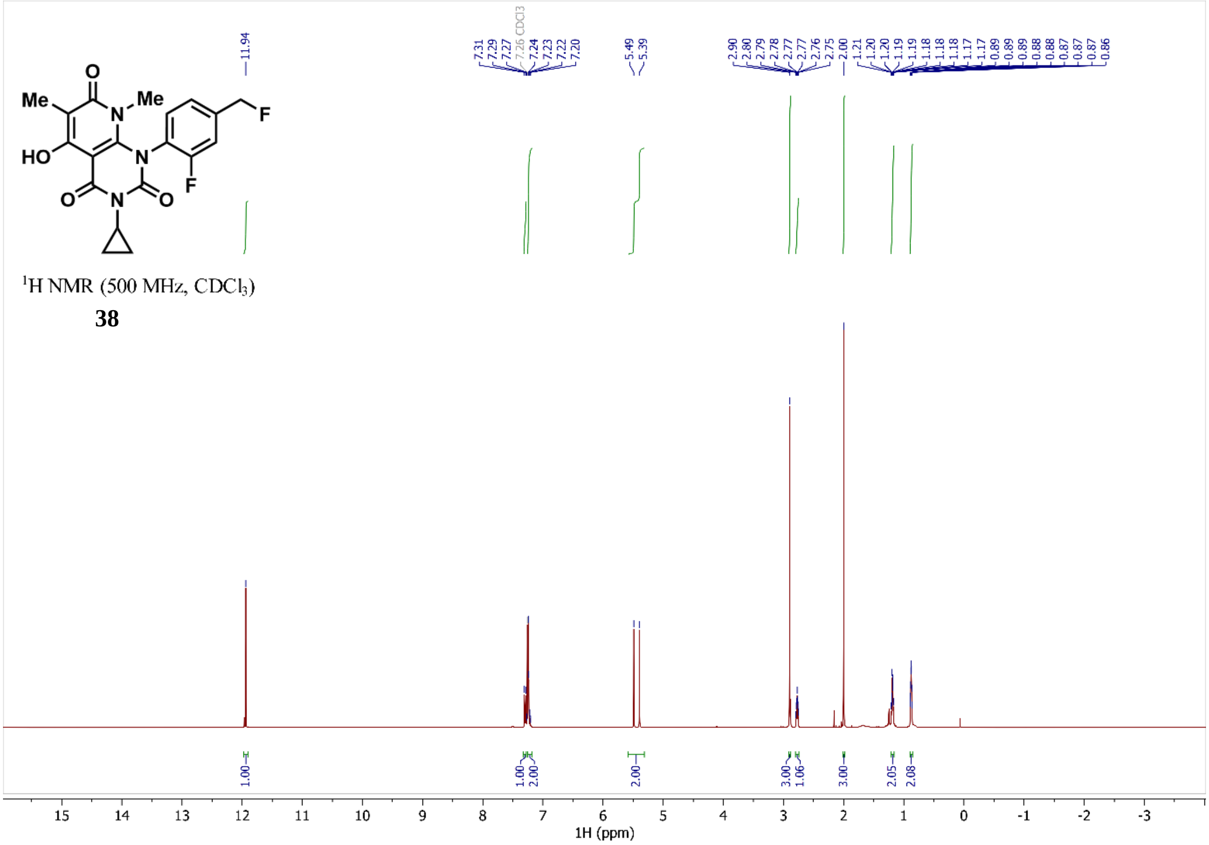

^{1}H NMR (500 MHz, $CDCl_3$)

38

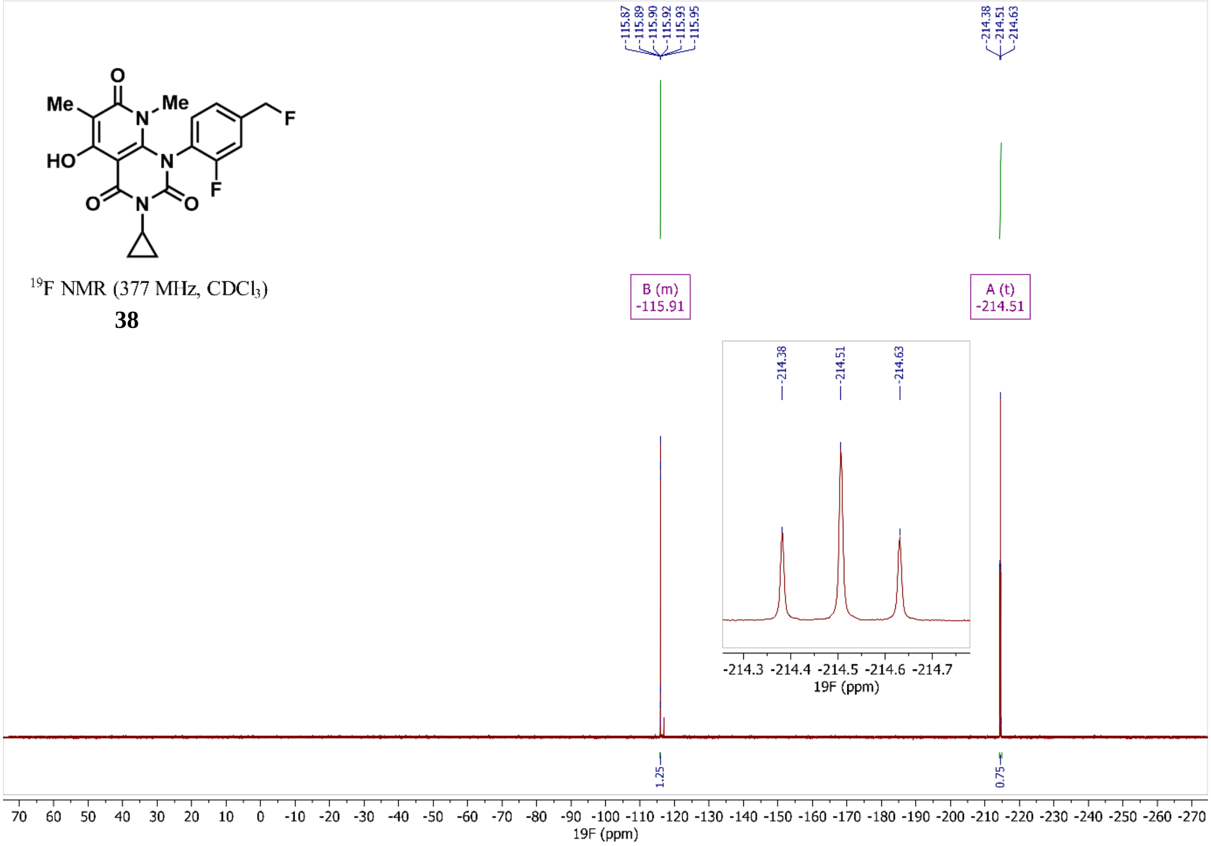

^{19}F NMR (377 MHz, CDCl$_3$)
38
B (m)
-115.91
A (t)
-214.51
19F (ppm)

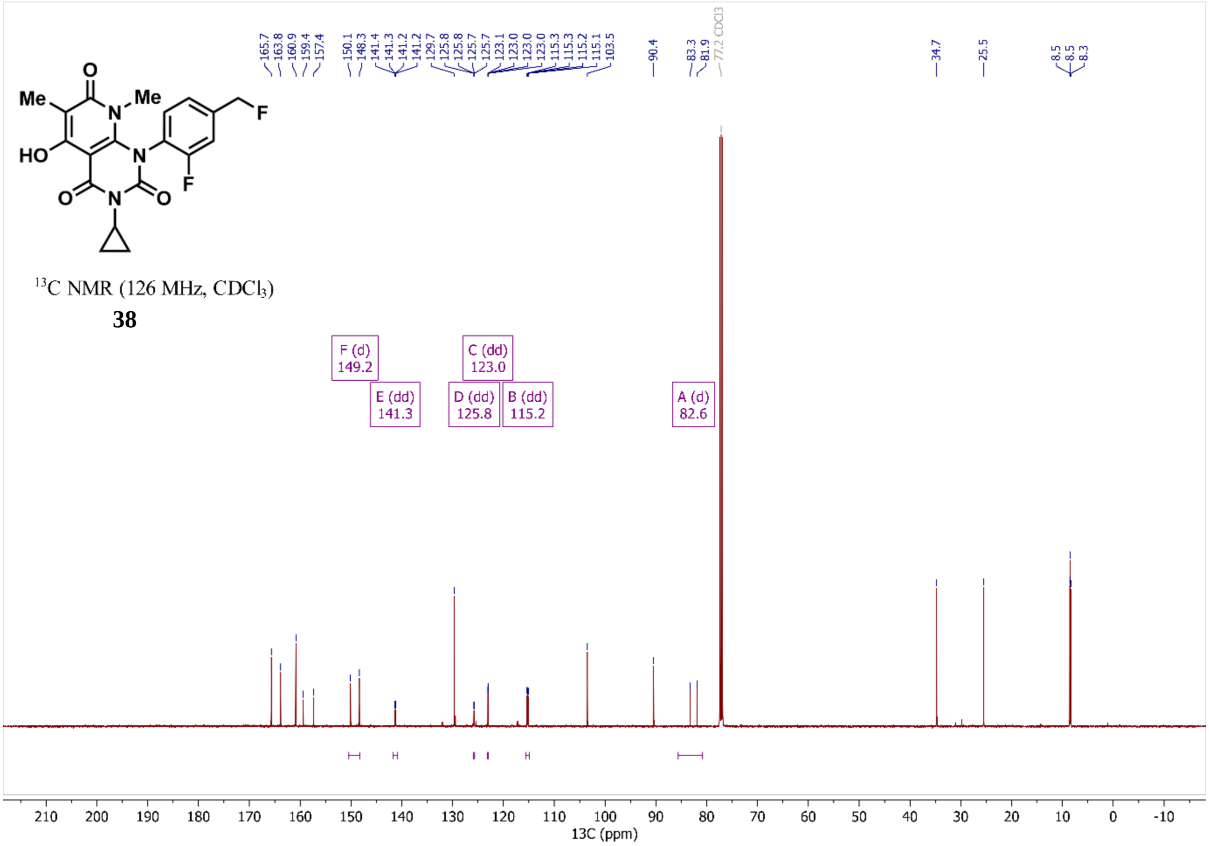

^{13}C NMR (126 MHz, CDCl$_3$)
38
F (d)
149.2
E (dd)
141.3
C (dd)
123.0
D (dd)
125.8
B (dd)
115.2
A (d)
82.6
13C (ppm)

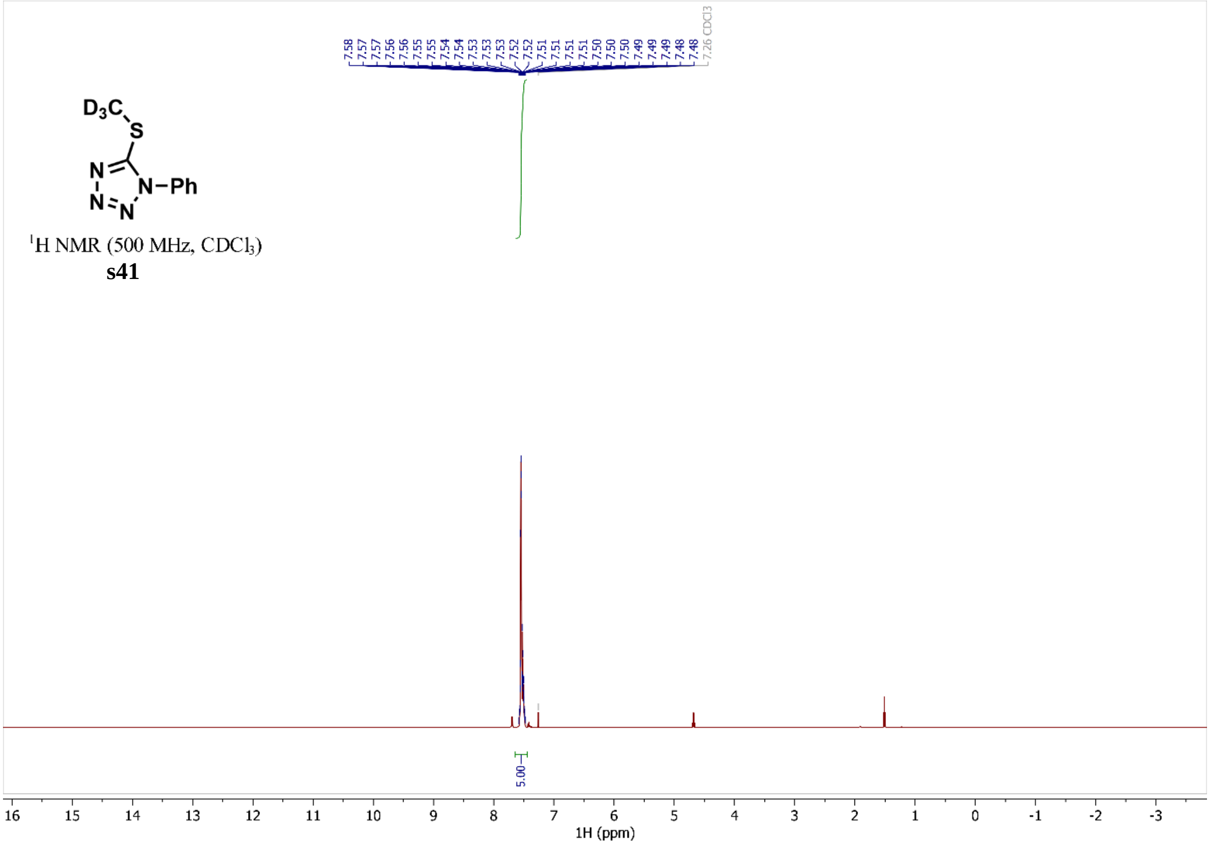

^{1}H NMR (500 MHz, $CDCl_3$)

s41

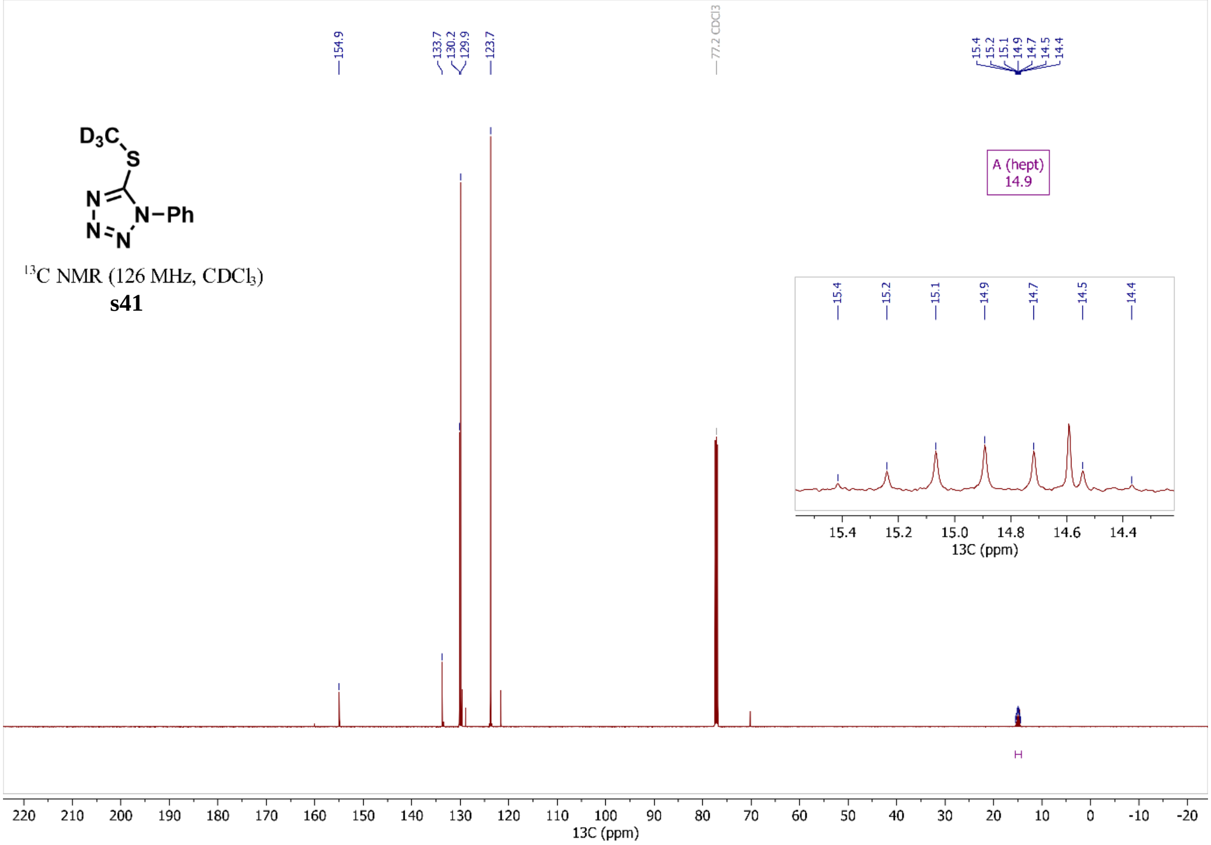

^{13}C NMR (126 MHz, $CDCl_3$)

s41

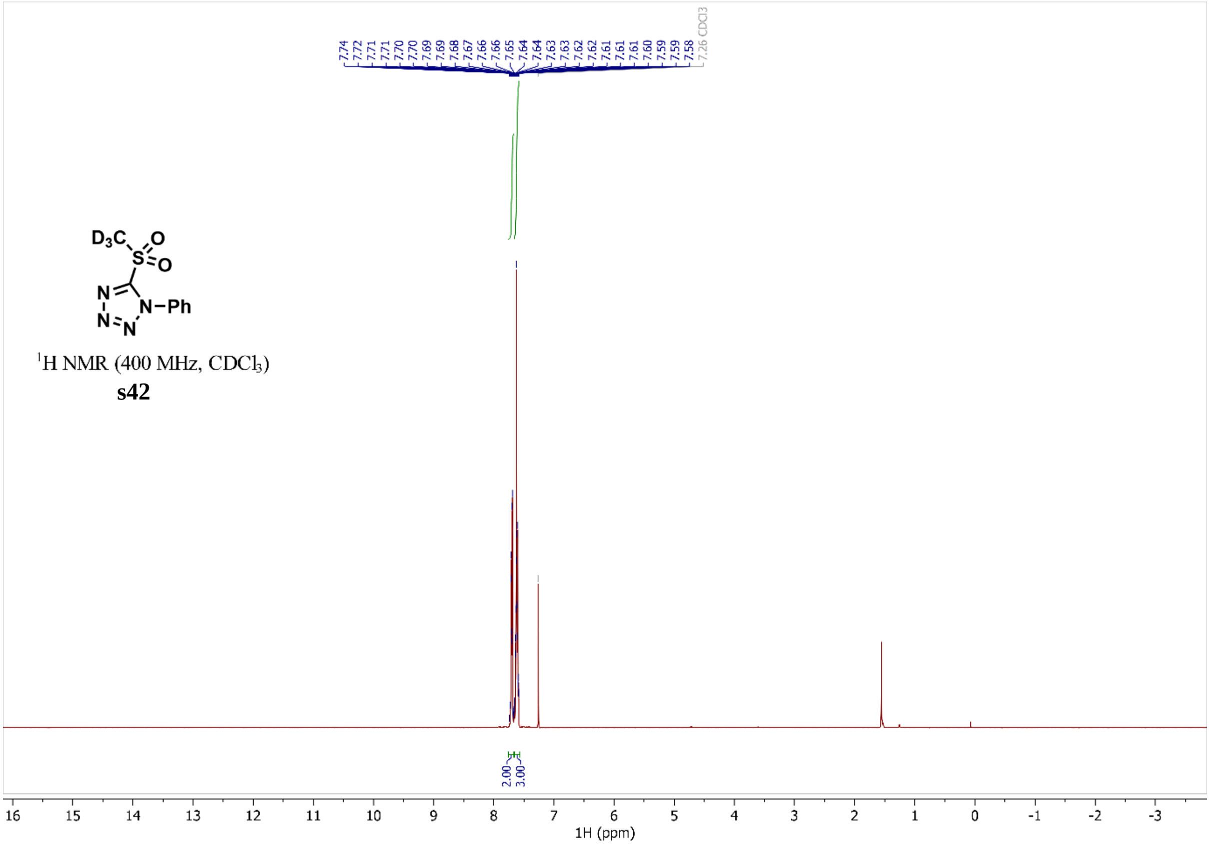

^1H NMR (400 MHz, $CDCl_3$)

s42

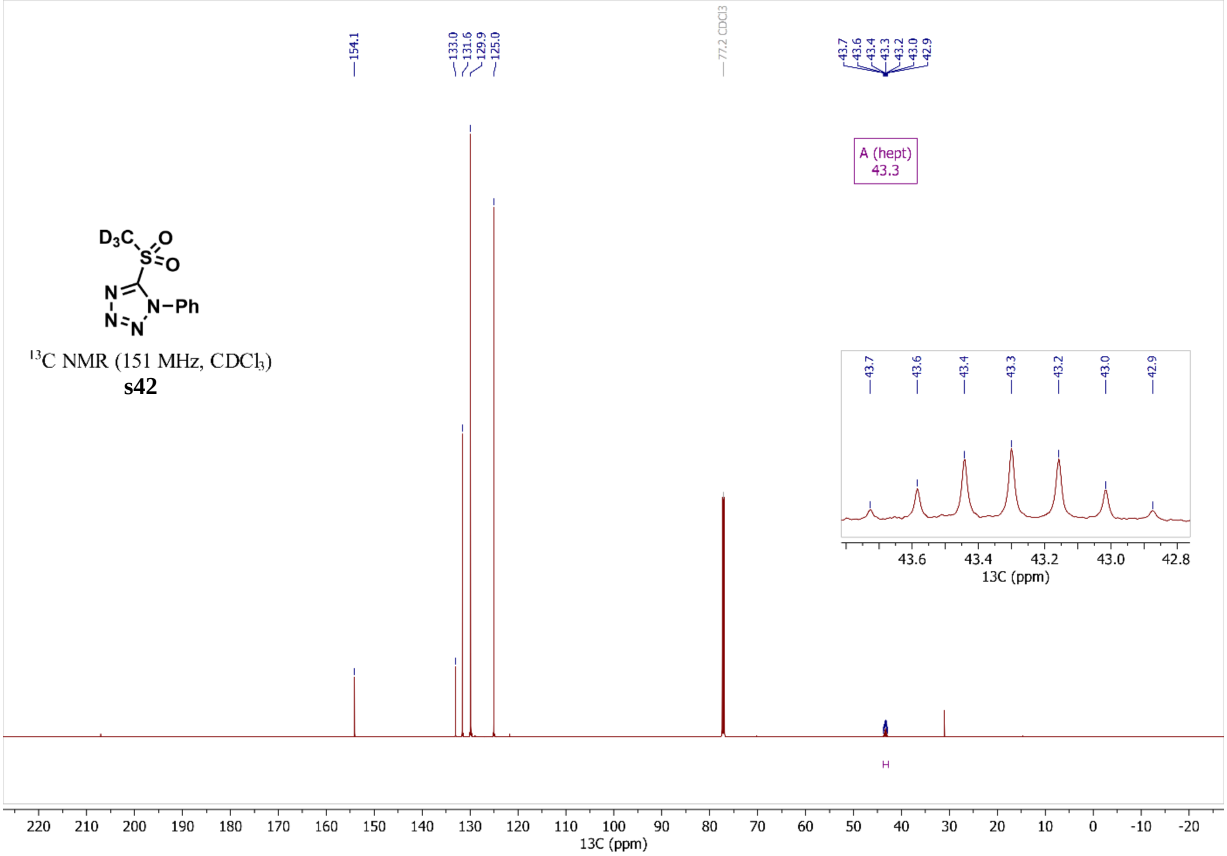

^{13}C NMR (151 MHz, $CDCl_3$)

s42

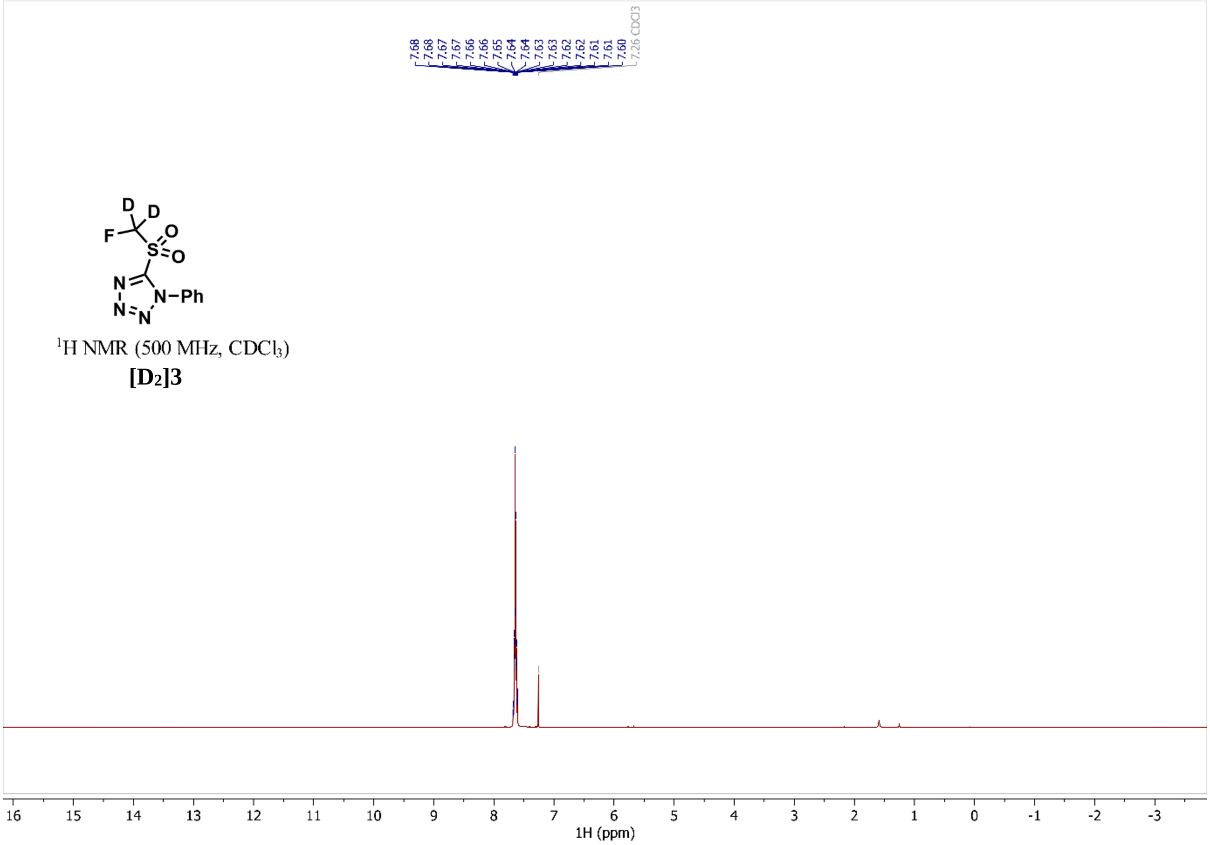

^{1}H NMR (500 MHz, $CDCl_3$)

[D2]3

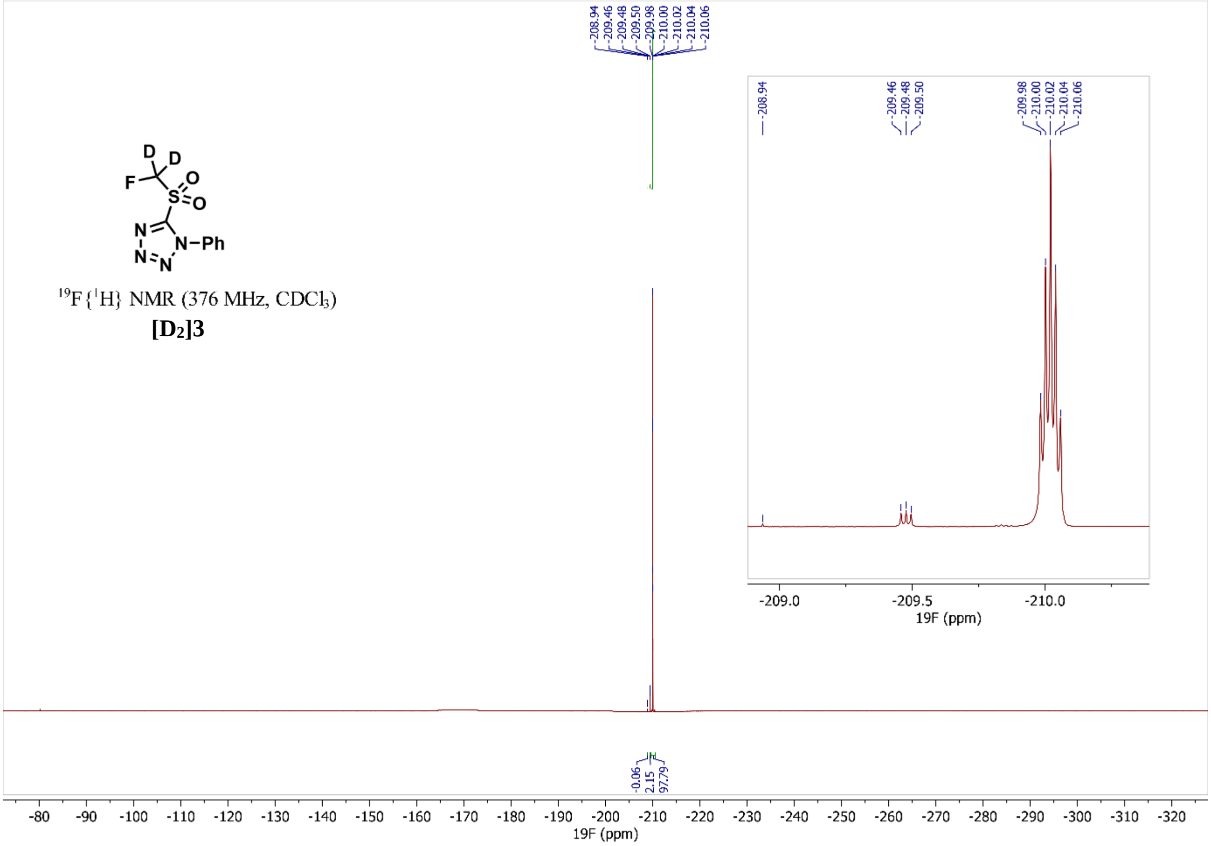

$^{19}F\{^{1}H\}$ NMR (376 MHz, $CDCl_3$)

[D2]3

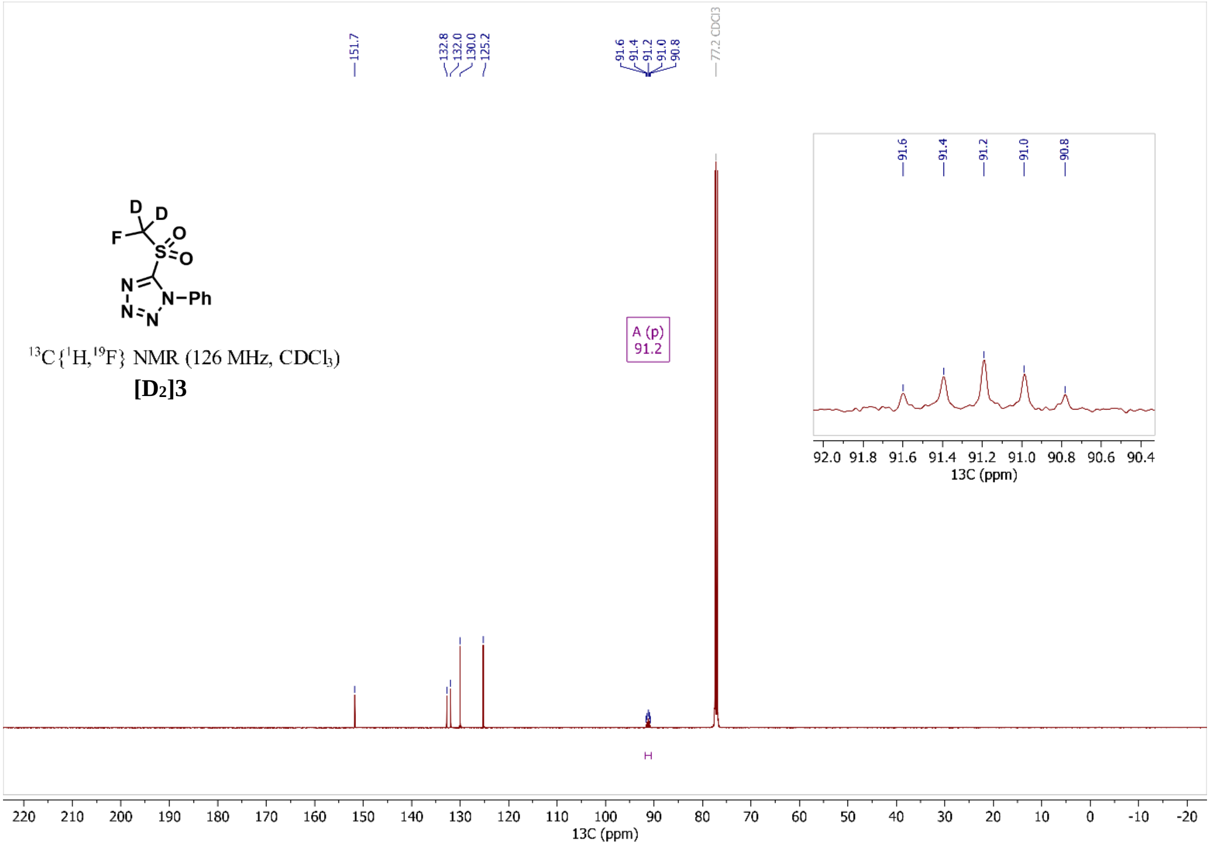

151.7
132.8
132.0
130.0
125.2
91.6
91.4
91.2
91.0
90.8
77.2 CDCl3
D
D
F
S
O
O
N
N
N
N
N−Ph
13C{1H,19F} NMR (126 MHz, CDCl3)
[D2]3
A (p)
91.2
92.0 91.8 91.6 91.4 91.2 91.0 90.8 90.6 90.4
13C (ppm)
220 210 200 190 180 170 160 150 140 130 120 110 100 90 80 70 60 50 40 30 20 10 0 -10 -20
13C (ppm)
